# Identification of miRNAs and their target genes in developing maize ears by combined small RNA and degradome sequencing

**DOI:** 10.1186/1471-2164-15-25

**Published:** 2014-01-14

**Authors:** Hongjun Liu, Cheng Qin, Zhe Chen, Tao Zuo, Xuerong Yang, Huangkai Zhou, Meng Xu, Shiliang Cao, Yaou Shen, Haijian Lin, Xiujing He, Yinchao Zhang, Lujiang Li, Haiping Ding, Thomas Lübberstedt, Zhiming Zhang, Guangtang Pan

**Affiliations:** 1 Maize Research Institute of Sichuan Agricultural University/Key Laboratory of Biology and Genetic Improvement of Maize in Southwest Region, Ministry of Agriculture, Chengdu 611130, China; 2 Zunyi Institute of Agricultural Sciences, Zunyi 563102, China; 3 Interdepartmental Genetics Graduate Program, Iowa State University, Ames 50011, USA; 4 Animal Nutrition Institute, Sichuan Agricultural University, Ya’an 625014, China; 5 BGI-Shenzhen, Shenzhen 518083, China; 6 Maize Research Institute of Heilongjiang, Academy of Agricultural Sciences, Harbin 150086, China; 7 Sichuan Agricultural University, Chengdu 611130, China; 8 Department of Agronomy, Iowa State University, Ames 50011, USA

## Abstract

**Background:**

In plants, microRNAs (miRNAs) are endogenous ~22 nt RNAs that play important regulatory roles in many aspects of plant biology, including metabolism, hormone response, epigenetic control of transposable elements, and stress response. Extensive studies of miRNAs have been performed in model plants such as rice and *Arabidopsis thaliana*. In maize, most miRNAs and their target genes were analyzed and identified by clearly different treatments, such as response to low nitrate, salt and drought stress. However, little is known about miRNAs involved in maize ear development. The objective of this study is to identify conserved and novel miRNAs and their target genes by combined small RNA and degradome sequencing at four inflorescence developmental stages.

**Results:**

We used deep-sequencing, miRNA microarray assays and computational methods to identify, profile, and describe conserved and non-conserved miRNAs at four ear developmental stages, which resulted in identification of 22 conserved and 21-maize-specific miRNA families together with their corresponding miRNA*. Comparison of miRNA expression in these developmental stages revealed 18 differentially expressed miRNA families. Finally, a total of 141 genes (251 transcripts) targeted by 102 small RNAs including 98 miRNAs and 4 ta-siRNAs were identified by genomic-scale high-throughput sequencing of miRNA cleaved mRNAs. Moreover, the differentially expressed miRNAs-mediated pathways that regulate the development of ears were discussed.

**Conclusions:**

This study confirmed 22 conserved miRNA families and discovered 26 novel miRNAs in maize. Moreover, we identified 141 target genes of known and new miRNAs and ta-siRNAs. Of these, 72 genes (117 transcripts) targeted by 62 differentially expressed miRNAs may attribute to the development of maize ears. Identification and characterization of these important classes of regulatory genes in maize may improve our understanding of molecular mechanisms controlling ear development.

## Background

Maize is one of the most productive crops worldwide, and is widely used as a model plant in genetics research [1]. Maize produces two distinct inflorescences, commonly referred to as the tassel and the ear. In this respect, it differs from other grasses such as rice and wheat. The tassel arises from the apex of the mature plant, while ears originate from axillary bud apices [2]. One obvious difference in morphology between the two inflorescences is the presence (tassel) or absence (ear) of a variable number of long branches originating at the base. In previous studies, the wide range of natural variation among different inbred lines was used to identify quantitative trait loci (QTL) underlying a variety of phenotypes by association mapping [3,4]. Several genes associated with maize ear development have been identified in genetic and molecular studies [2]. However, knowledge about maize ear development is still limited, and most of the genes involved in this process are still unknown.

In plants, small RNA-guided post-transcriptional regulatory mechanisms play important roles in many aspects of plant biology, including metabolism [5], hormone responses [6], epigenetic control of transposable elements [7], and responses to biotic stress [8] and abiotic stress [9]. The two main types of small RNAs are microRNAs (miRNAs) and small interfering RNAs (siRNAs) [10]. Over recent decades, many miRNA families have been discovered in plants, and have been shown to regulate more aspects of plant biology than siRNAs [5,11,12]. Published reports as well as publicly accessible miRNA datasets (miRBase, version 20, http://www.mirbase.org/) [13], mainly based on model plants, suggest that miRNAs in plants are complex and abundant [14-17]. Therefore, identification of miRNAs and their targets in diverse species has been a major focus in recent years.

So far, conserved miRNAs in maize have been identified by sequence homology analyses [18-20], and new miRNA sequences have been identified by traditional [21-23] or high-throughput [17,24-30] sequencing methods. These miRNA sequences can be found in miRBase databases [13]. Functional analysis has been carried out for only a few maize miRNAs, mainly in their role in flower development [21,31-36].

There are three main objectives of this study. The first objective is to identify conserved and novel miRNAs in maize ears at four different developmental stages. The second objective is to combine publically available *Arabidopsis thaliana*, *Oryza sativa*, *Sorghum bicolor*, and *Zea mays* miRNAs data with the new *Zea mays* miRNAs data to generate a miRNA microarray platform to analyze the dynamics of miRNA expression. Finally, to discover the targets of conserved and non-conserved miRNAs, we aimed to identify the remnants of small RNA-directed target cleavage by sequencing the 5′ ends of uncapped RNAs using a degradome sequencing approach.

## Results

### Overview over small RNA library sequencing

**Figure 1:**
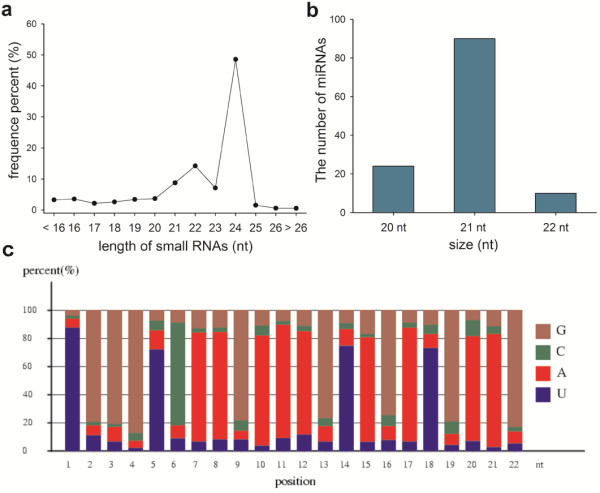
**Size distribution of small RNAs by deep sequencing and characterizations of maize miRNA. (a)** Size distribution of sequenced sRNAs in total RNA; **(b)** The composition of maize miRNAs of various lengths; **(c)** The relative nucleotide bias at each position of the miRNAs in total RNA.

To study the involvement of regulatory miRNAs in the complex process of ear development, we profiled miRNA accumulation during ear development in the maize inbred line B73. We constructed a maize small RNA library using mixed RNAs obtained from ears at four different developmental stages. Sequencing was conducted on the Illumina platform. We obtained more than 10.67 million raw clean reads, ranging from 18 nt to 30 nt in length. After trimming adaptor sequences and removing contaminated reads, clean reads were aligned against the Maize B73 RefGen v2 working gene set using SOAP2 software [37]. We found that 7,981,459 (3,436,342 distinct) reads matched perfectly to the maize genome, representing 74.85% of total reads (66.64% of distinct reads). Of the distinct reads, 5.22% matched with non-coding RNAs in Rfam and NCBI Genbank databases; these non-coding RNAs included snoRNAs (0.01%), snRNAs (0.03%), tRNAs (0.15%), rRNAs (1.69%), and siRNAs (3.34%) (Additional file 1). The remaining reads were then used to identify conserved and new miRNAs. The length of these small RNAs ranged from 20 nt to 24 nt. Of these, the 24 nt category was the most abundant small RNA (48.55%) (Figure 1a), followed by 22 nt (14.18%) and 21 nt (8.78%). These were consistent with the typical lengths of plant mature small RNAs reported in other studies [14,27,38,39].

### Computational identification of genuine miRNAs during maize ear development

To date, research on identifying conserved and novel miRNAs has used several standard methods and databases, including Rfam, GenBank, and miRBase. Because of their low expression levels and sequence depths, it is always difficult to predict miRNAs. Hence, we used a strict strategy with eight steps to predict and identify known and novel miRNAs based on the characteristic features of miRNAs specifically processed by Dicer-like proteins from canonical stem-loop regions of longer RNA precursors [40,41]. We used an integrated strategy combining high-throughput sequencing with bioinformatics analyses to identify miRNAs meeting all reported previously criteria (see “Methods”) [42].

As shown in the schematic diagram of the strategy (Additional file 2), our computational analysis generated 508 loci folded within typical stem-loop structures (Additional file 3). After excluding 38 loci that overlapped with protein-coding gene exons, 76 loci overlapping transposable elements and other repetitive elements, and 9 loci with free energy lower than −20 kcal/mol (Additional file 4), the remaining 385 loci were considered to be candidate miRNA genes. We used miRAlign to identify paralogs or orthologs of these 385 candidate miRNA genes by comparing their sequences with those of known miRNAs, as described previously [43]. From this analysis, we detected 99 known miRNA genes encoding 96 mature miRNAs and three miRNA star (miRNA*) (Additional file 4). We also detected 64 novel miRNA* sequences (Additional file 5).

In plants, it is difficult to identify new miRNAs, even when they have the characteristic hairpin feature, because of abundant inverted repeats that can also fold into dysfunctional hairpins [44,45]. Thus, we used additional strategies that were not based on phylogenetic conservation to identify non-conserved pre-miRNAs. We used MiPred (http://www.bioinf.seu.edu.cn/miRNA/) to distinguish pre-miRNAs from other similar segments in the maize genome. Among the remaining 286 candidate pre-miRNA-like hairpins, 52 were classified as pseudo-pre-miRNAs and 198 were not pre-miRNA-like hairpins. The other 36 loci, which encoded 26 non-redundant mature miRNAs (Table 1), were identified as maize-specific miRNA genes. Of these 26 miRNAs, 25 belonged to new families that have not been reported in plants (Additional file 4, Additional file 5). Here, we have designated them in the form of their zma-miR-specific-number, e.g., zma-miRs2. When several maize-specific miRNAs belonged to the same family, we named them in a similar manner to that used to name known mature miRNAs [15] (e.g. zma-miRs6a and zma-miRs6b). All of the new miRNA precursors had regular stem-loop structures. We also detected four miRNA* (Additional file 3, Additional file 4, Additional file 5, Additional file 6), providing further evidence for the existence of this class of miRNAs in maize.

**Table 1:** Newly identified miRNAs

**miRNA**	**Sequence (5'-3′)**	**Ori**	**Size (nt)**	**Loci**	**miRNA abundance**	Sequence of cloned putative miRNA* (5′-3′)	miRNA* abundance	**Precursor location**
zma-miRs1a	GGUGAACCACCGGACAUCGCAC	5′	22	1	13	NO	0	Chr10:170455..170536:-
zma-miRs1b	GGUGAACCACCGGACAUCGCAC	3′	22	1	21	NO	0	Chr1:261835708..261835791:-
zma-miRs2	UUUGACCAAGUUUGUAGAAAA	5′	21	1	10	NO	0	Chr10:7112184..7112314:-
zma-miRs3	UAUACACUGUGGUUGUGGAUG	5′	21	1	64	NO	0	Chr10:140401501..140401631:-
zma-miRs4	UAGCCAAGCAUGAUUUGCCCGU	5′	22	1	7	NO	0	Chr1:297173995..297174083:+
zma-miRs5	UGAUGCCAUUCAUUAAUCUC	5′	20	3	16	NO	0	Chr1:16908651..16908718:-
zma-miRs6a	UGGGUCAAGAAAGUAGAUGAAG	5′	22	6	4439	UCAUGUAUUUCUUCAUCCAGG	6	Chr1:145766064..145766151:-
zma-miRs6b	UGGGUCAAGAAAGUAGAUGAAG	5′	22	2	4314	UCAUGUAUUUCUUCAUCCAGG	6	Chr8:65126597..65126681:+
zma-miRs7	UUGGAGGGGAUUGAGGGGGCUA	5′	22	1	13	NO	0	Chr2:160587100..160587178:-
zma-miRs8	UUGGAUUGGUUUAGAGUGGUU	5′	21	3	9	NO	0	Chr2:193765038..193765121:-
zma-miRs9	UUAGGCUCGGGGACUAUGGUG	5′	21	1	56	CCGUGGCUCCUGCUCCUGAUG	1	Chr3:37277918..37278069:+
zma-miRs10	AUCGGCUGAUCGUUUGGCCUG	5′	21	1	12	NO	0	Chr3:95960134..95960203:+
zma-miRs11	CGUGGAACUUCUUCGGCGUAG	5′	21	1	59	GUGCCGAAGAAGAACUUCCUGCA	2	Chr4:6987996..6988075:-
zma-miRs12	UGGCUGUGAUGACAAAAAGGU	5′	21	1	14	NO	0	Chr5:13726506..13726621:+
zma-miRs13	UGAGUUUAGGGACUGGGAUGG	3′	21	1	5	NO	0	Chr5:33732172..33732274:+
zma-miRs14	UGAAACUGUCACAGCAUGAUC	5′	21	1	6	NO	0	Chr5:7648838..7648949:-
zma-miRs15	UGUUCGGUUGCUCAGGAACGGU	5′	22	1	9	NO	0	Chr6:102874227..102874366:+
zma-miRs16	UUGCCAGGAGGAGGAUGGAGC	3′	21	1	65	NO	0	Chr9:7189032..7189100:+
zma-miRs17	UGAAAAGCUAGAACGAUUUAC	3′	21	1	9	NO	0	Chr9:33858898..33859002:-
zma-miRs18	GUACUACGGGUACUGCGAGC	3′	20	1	5	NO	0	Chr1:140132808..140132921:+
zma-miRs19a	UGGUUGACAUAUGGACCCCAC	5′	21	1	5	NO	0	Chr2:196666638..196666791:+
zma-miRs19b	UGGUUGACAUAUGGACCCCAC	5′	21	1	5	NO	0	Chr2:196666638..196666790:-
zma-miRs19c	UGGUUGACAUAUGGACCCCAC	5′	21	1	5	NO	0	Chr5:182646213..182646347:+
zma-miRs19d	UGGUUGACAUAUGGACCCCAC	5′	21	1	5	NO	0	Chr9:114976246..114976391:-
zma-miRs20	AGGGCUUGUUCGUUUUGGAGU	5′	21	1	35	NO	0	Chr4:41972777..41972854:-
zma-miRs21	GGUGUUGGUGCCUGUAGCGG	5′	20	1	7	NO	0	Chr7:6041852..6041939:+

^*^indicated miRNA star (miRNA*).

### Characterization of newly identified miRNAs in maize

As expected, approximately all 22 the conserved miRNA families in the small RNA library were identified in this study. However, we detected miRNA* sequences of zma-miR171h/k and zma-miR408b instead of their corresponding mature miRNA sequences (Additional file 5). We also identified five mature miRNAs (zma-miR160e, zma-miR166e/f, zma-miR169g, and zma-miR172e) previously predicted by similarity searches [18,20] and unexpectedly found their corresponding miRNA* sequences (zma-miR160e*, zma-miR166e*/f*, and zma-miR169g*), which were not available in miRBase. Besides the known miRNAs, we also identified 26 new miRNA candidates (Table 1, Additional file 6), and nine (zma-miRs1a/b, zma-miRs2, zma-miRs4, zma-miRs7, zma-miRs9, zma-miRs12, zma-miRs14, and zma-miRs17) were previously reported [27] (Additional file 5). The sequence of miRs4 was similar to that of members of the miR169 family (Additional file 7), indicating that miRs4 may be a member of that family. Most of the new miRNAs could only be produced from one locus. However, zma-miRs6b and four other new miRNA genes (zma-miRs5, zma-miRs6a, zma-miRs8, and zma-miRs23) could be produced from two or more loci (Additional file 4). Among the newly identified miRNAs, 21-nt miRNAs were the most abundant category (52.6%) (Figure 1b). Analysis of the nucleotide sequences of these miRNAs revealed that uridine (U) was the most common nucleotide at the 5′ end (>80%) (Figure 1c), and the 10th and 11th nucleotides, which match to the cleavage site of targets, were usually adenine (A). Also, U was the most common nucleotide at positions 21 and 22 in these miRNAs.

Next, we conducted microarray assays to analyze expressions of the known and newly identified miRNAs during maize ear development. We detected transcripts of all of the conserved miRNAs and 20 out of 26 (76%) maize-specific miRNAs in the microarray experiment. Those that were undetected either had a low affinity to the chip probes or very low transcript levels (sequencing frequency <50) (Additional file 8). These results suggest that Solexa sequencing is a more specific and efficient tool than the miRNA microarray assay for identifying mature miRNAs. In our study, we detected six miRNA families (miR408/482/827/397/398/2118) in the microarray assay that were not present in the Solexa sequencing data (Additional file 8, Additional file 9). These miRNAs need to be further validated.

Although we identified 122 miRNAs (96 conserved + 26 non-conserved) and 64 miRNA*s, they showed a diverse range of abundance, and only a few miRNA families dominated in the miRNA library and microarray assay data. The six most abundantly expressed miRNA families were miR166, miR168, miR167, miR156, miR159, and miRs6. There were extremely low frequencies of miR395, miR399, miR2275, miRs12, and miRs19, possibly because these families are expressed in a tissue-specific manner. Most of the miRNA*s showed very low transcript levels (sequencing frequency <30), much lower than those of their homoplastic miRNAs, consistent with previous findings [12]. The transcript level of zma-miR408b was lower than that of zma-miR408b*, and the mature product from the 3′ arm of the hairpin suggested that the 3′ arm may be functional.

### Expression profiles of known and newly identified miRNAs

**Figure 2:**
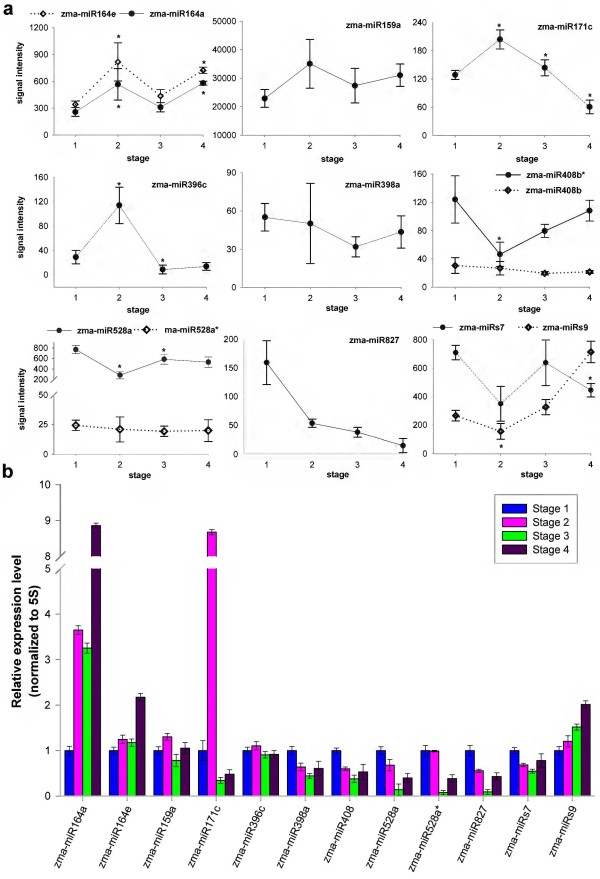
**Expression of 12 miRNAs in developmental ears in maize.** The expression pattern of 12 miRNAs detected by microarray experiment **(a)** and stem-loop quantitative Real-Time PCR (qRT-PCR) **(b)**. Stages 1, 2, 3, and 4 represent stage I, stage II, stage III, and stage IV, respectively. *indicated the significant difference in adjacent two developmental stages (one-way ANOVA and Fisher′s LSD, p < 0.01, n = 4). The blue, pink, green, and purple bars in graph b depicted the stem-loop qRT-PCR relative expression level ± standard error of three replicates for each miRNA in the four developmental stages of maize ears, respectively.

To analyze miRNA expression during maize ear development, we analyzed the miRNA expression profiles of ear samples collected at four different developmental stages using microarray assays. Conserved mature miRNAs are generally conserved among plant species and are stably expressed in diverse tissues. However, when microarray technology is used to analyze expression, members of the same miRNA family with 1–3 nt sequence differences need to be normalized for further analyses because hybridization can occur between members of the same miRNA family across different species [46]. Hence, a total of 53 miRNAs, about 8.4% (53/632) of the probes on the microarray, were identified as putative differentially expressed miRNAs (*P = 0.01*) (Table 2). Of these, 45 miRNAs aligned with 59 members of 21 maize miRNA families, while the others corresponded to members of miRNA families from three other plant species, including rice (osa-miR156/162/164/168/396/529) *Arabidopsis* (ath-miR156/164/167) and sorghum (sbi-miR396). The results shown in Additional file 10: Figure S3 indicated that the differentially expressed miRNAs may be specially regulated in diverse pathways during ear development. A sample of 12 expressed miRNAs was randomly selected for validation by stem-loop qRT-PCR. The trends in the expression of these miRNAs detected by microarray experiments were consistent (7 miRNAs) or partially (5 miRNAs) consistent with those determined in stem-loop qRT-PCR analyses (Figure 2).

**Table 2:** The differentially expressed miRNA families and members

**miR family**	**Members of identified miRNAs**	**miR family members**	**signal (> 500)**
zma-miR156	zma-miR156a/j/k	zma-miR156a/b/c/d/e/f/g/h/i/l	no
	osa-miR156l	zma-miR156j	no
	ath-miR156g	zma-miR156k	no
zma-miR160	zma-miR160a	zma-miR160a/b/c/d/g/h	no
zma-miR162	zma-miR162	zma-miR162	no
	osa-miR162a/b		
zma-miR164	zma-miR164a/e/f/h	zma-miR164a/b/c/d/g	yes
	osa-miR164c/e	zma-miR164e	yes
	ath-miR164c	zma-miR164f	yes
		zma-miR164h	no
zma-miR167	zma-miR167a/e	zma-miR167a/b/c/d	yes
	ath-miR167c/d	zma-miR167e/f/g/h/i/j	yes
zma-miR168	zma-miR168a	zma-miR168a/b	yes
	osa-miR168a/b		
zma-miR171	zma-miR171b/c/d/g	zma-miR171b/d/e/i/j	no
		zma-miR171c	no
		zma-miR171g	no
zma-miR172	zma-miR172e	zma-miR172e	no
zma-miR319	zma-miR319a	zma-miR319a/b/c/d	yes
zma-miR390	zma-miR390a	zma-miR390a/b	no
zma-miR396	zma-miR396c	zma-miR396c/d	no
	osa-miR396f/g		
	sbi-miR396d		
zma-miR408	zma-miR408b*	zma-miR408b*	no
zma-miR528	zma-miR528a	zma-miR528a/b	yes
zma-miR529	zma-miR529	zma-miR529	no
	osa-miR529b		
zma-miR827	zma-miR827	zma-miR827	no
zma-miRs7	zma-miRs7	zma-miRs7	yes
zma-miRs9	zma-miRs9	zma-miRs9	no
zma-miRs16	zma-miRs16	zma-miRs16	no

^*^indicated miRNA star (miRNA*).

### Target prediction of conserved and non-conserved miRNAs by degradome sequencing

To identify small RNA targets at a global level in maize, we used the recently developed degradome library sequencing technology [47,48]. We generated four libraries from maize ears at different developmental stages (I-IV) as described above. High-throughput sequencing yielded 13,638,690 (1,706,149 distinct), 18,257,616 (2,887,536 distinct), 9,477,595 (682,164 distinct), and 8,393,209 (2,490,241 distinct) 20-nt sequences representing the 5′ ends of uncapped, poly-adenylated RNAs for stages I to IV, respectively. The total number of signatures matching to the genome was 10,596,420 for stage I, 14,571,419 for stage II, 7,415,394 for stage III, and 6,524,350 for stage IV. The number of distinct sequences in the four libraries matching to the genome was 1,123,608 (66%) for stage I, 1,995,882 (69%) for stage II, 423,065 (62%) for stage III, and 1,746,858 (70%) for stage IV. The number of signatures that matched to only one location in the genome was relatively high: 825,904 (74%) for stage I, 1,521,543 (76%) for stage II, 317,671 (75%) for stage III, and 1,318,724 (70%) for stage IV, suggesting that 20-nt signatures are sufficient to identify their origin in the maize genome. Of these, 973,186 (87%), 1,816,631 (91%), 382,792 (90%) and 1,580,297 (90%) distinct signatures for stage I, II, III, and IV, respectively, could be mapped to annotated maize gene models (B73 RefGen_v2). A small proportion of the distinct signatures (0.8%, 0.7%, 0.9%, and 0.7% for stage I, II, III, and IV, respectively) could also be mapped to the maize chloroplast or mitochondrial genomes. The number of distinct signatures matching to rRNAs, tRNAs, small nucleolar RNAs (snRNAs) or small nuclear RNAs was 10,101 (0.9%) for stage I, 9,596 (0.5%) for stage II, 4,521 (1.1%) for stage III, and 11,572 (0.7%) for stage IV. These were removed before subsequent analyses (Additional file 10). Similarly, we removed the sequences matching to repeats/transposons that were revealed by searches against the repeat database (http://www.girinst.org/server/RepBase/). Interestingly, a significant proportion of distinct signatures from the four libraries matched to introns and intergenic regions, similar to the findings of previous transcript profiling analyses [47,48].

Based on previous studies, a characteristic scenario of miRNA-guided slicing is that the cleavage takes place precisely between the 10th and 11th nt from the 5′ end of miRNA in the complementary region of the target transcript [47-51]. We used CleaveLand pipeline [52] to identify sliced miRNA targets in the maize transcriptome. Various sequenced tags were plotted on each of the target transcripts (Additional file 11). The cleaved target transcripts were categorized into five classes (categories 0, 1, 2, 3, and 4) as reported previously for *Arabidopsis*[47], grapevine [49], rice [51], and soybean [50].

**Figure 3:**
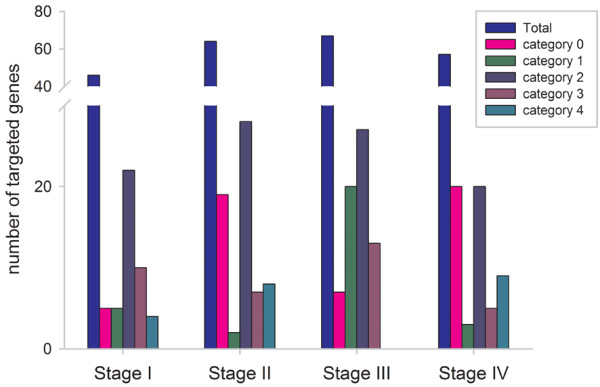
Summary of cleaved miRNA target categories found with degradome analyses in four developmental stages of maize ears.

For conserved miRNAs and ta-siRNAs, 120 target genes (234 transcripts) were identified in ears at the four stages of development (Table 3, Figure 3 Additional file 12). Reads associated with most of these miRNA targets were over-represented (Additional file 11). However, only 15% of the miRNA targets (19 out of 127) were identified in all four stages. The targets were classified into categories 0–4 based on the abundance of degradome tags indicative of miRNA-mediated cleavage. In stage I, II, III, and IV, there were 5, 19, 7, and 20 targets classified as category 0 (where miRNA-guided cleavage remnants were the most abundantly recovered species) [53]. There were 5, 2, 20, and 3 targets in stage I, II, III, and IV, respectively, classified as category 1 (where the abundance of cleavage products was equal to the maximum, and there was more than one maximum position on the transcript). In stage I, II, III, and IV, there were 22, 28, 27, and 20 targets classified as category 2 (where the abundance of cleavage sequences was less than the maximum but greater than the median for the transcript). In stage I, II, III, and IV there were 10, 7, 13 and 5 target transcripts classified as category 3 (where the total abundance of degradome sequences at the cleavage site was equal to or less than the median for the transcript). All other transcripts were classified as category 4 (where only one raw read matched the 5′ end of a slicing remnant). Only 4, 8, 0, and 9 targets in stage I, II, III, and IV, respectively, were in category 4. Among the identified targets, category 2 was the most abundant category among the four degradome libraries (Tables 3, 4 and Additional file 11).

**Table 3:** Conserved maize miRNA targets identified by degradome sequencing in this study

**miRNA**^**a**^	**Target gene**	**Location of the target site**	**Repeat normalized read abundance (category)**^**b**^				**Target gene annotation**^**c**^	**Other degradome evidence**	**5′-RACE or genetic experiment**
			**I**	**II**	**III**	**IV**			
miR156	GRMZM2G156621	CDS	2.50 (3)	3.50 (2)	39.00 (2)		SBP-box transcription factor^#^	[26]	N
miR156	GRMZM2G067624	3′-UTR	18.00 (2)	46.50 (2)	51.50 (2)	97.00 (2)	SBP-box transcription factor^#^	[26,28]	N
miR156	GRMZM2G163813	CDS/3′-UTR	3.33 (2)	17.67 (2)			SBP-box transcription factor^#^	[26,28]	N
miR156	GRMZM2G113779	3′-UTR	18.00 (2)	4.00 (2)	86.00 (2)	352.00 (0)	SBP-box transcription factor	[28]	N
miR156	GRMZM2G126827	CDS	2.50 (3)	3.50 (2)	39.00 (2)		SBP-box transcription factor^#^	[26]	N
miR156	GRMZM2G318882	5′-UTR	0.03 (1)				SBP-box transcriptionfactor	N	N
miR156	GRMZM2G136158	CDS	0.03 (1)				SBP-box transcription factor	N	N
miR156	GRMZM2G460544	CDS			2.00 (3)		SBP-box transcription factor^#^	[26,28]	N
miR156	GRMZM5G878561	CDS			6.50 (3)	7.50 (0)	SBP-box transcription factor	[26]	N
miR156	GRMZM2G307588	CDS			2.00 (3)		SBP-box transcription factor^#^	[26,28]	N
miR156	GRMZM2G371033	CDS			6.50 (1)	7.50 (0)	SBP-box transcription factor	[26]	N
miR156	GRMZM2G160917	CDS/3′-UTR			2.00 (3)		SBP-box transcription factor^#^	[26,28]	N
miR156	GRMZM2G126018	CDS			2.00 (2)		SBP-box transcription factor#	[28]	N
miR159	GRMZM2G139688	CDS	76.00 (2)	169.00 (0)	86.00 (2)	3.00 (2)	MYB domain transcription factor^#^	[26,28]	N
miR159	GRMZM2G038195	CDS	2.5 (2)		5.50 (2)		Metallophosphoesterase^#^	N	N
miR159	GRMZM2G093789	CDS		0.67 (3)			MYB domain transcription factor^#^	[28]	N
miR159	GRMZM2G167088	CDS		2.00 (2)		0.33 (4)	MYB domain transcription factor^#^	N	N
miR159	GRMZM2G416652	CDS		2.00 (2)		0.33 (4)	Homeodomain-like#	N	N
miR159	GRMZM2G028054	CDS		24.00 (2)			MYB domain transcription factor^#^	[26,28]	N
miR159	GRMZM2G075064	CDS		0.67 (2)			MYB domain transcription factor^#^	N	N
miR159	GRMZM2G423833	CDS		0.67 (3)			MYB domain transcription factor^#^	[28]	N
miR159	GRMZM2G089361	CDS				1.33 (3)	TCP Transcription factor^#^	[26]	N
miR159	GRMZM2G387828	CDS				4.00 (1)	unknown	N	N
miR160	GRMZM2G005284	CDS	2.00 (1)	2.00 (2)			Auxin response factor^#^	N	N
miR160	AC207656.3_FGT002	CDS	6.50 (0)	18.00 (2)			Auxin response factor^#^	[26]	N
miR160	GRMZM2G159399	CDS	8.5 (2)	23.00 (2)		1.00 (4)	Auxin response factor^#^	[26,28]	N
miR160	GRMZM5G808366	CDS	10.00 (0)	3.00 (2)	32.00 (1)		Auxin response factor	N	N
miR160	GRMZM2G153233	CDS	24.00 (0)	68.00 (0)	37.00 (0)	1.00 (4)	Auxin response factor^#^	[26,28]	N
miR160	GRMZM2G390641	CDS		2.33 (0)	5.00 (2)		Auxin response factor^#^	[26]	N
miR160	GRMZM2G081406	CDS		2.33 (3)	5.00 (3)		Auxin response factor^#^	[26,28]	N
miR162	GRMZM2G040762	CDS				1.00 (4)	DICER-LIKE1	N	N
miR164	GRMZM2G457630	5′-UTR		2.50 (0)			No apical meristem (NAM) protein	N	N
miR164	GRMZM2G370846	3′-UTR			2.33 (1)		No apical meristem (NAM) protein	N	N
miR164	GRMZM2G370850	3′-UTR			2.33 (1)		No apical meristem (NAM) protein	N	N
miR164	GRMZM2G393433	CDS			2.50 (3)		Helix-loop-helix DNA-binding^#^	[28]	N
miR164	GRMZM2G163975	CDS	-	-	2.50 (1)	-	Helix-loop-helix DNA-binding^#^	N	N
miR166	GRMZM2G109987	CDS	0.6 (3)	2.40 (2)	15.80 (2)	27.80 (2)	bZIP transcription factor (rld1)^#^	[28]	N
miR166	GRMZM2G042250	CDS		2.00 (2)	15.75 (2)	17.00 (2)	bZIP transcription factor (rld2)^#^	[28]	N
miR166	GRMZM2G469551	CDS			25.50 (2)	28.00 (0)	bZIP transcription factor^#^	[26]	N
miR166	GRMZM2G123644	CDS				0.60 (2)	unknown	[26]	N
miR166	GRMZM2G336718	CDS				0.60 (2)	unknown	[26]	N
miR166	GRMZM2G055957	3′-UTR				9.50 (0)	unknown	[26,28]	N
miR166	GRMZM2G178102	CDS				0.60 (3)	bZIP transcription factor^#^	[26,28]	N
miR166	GRMZM2G003509	CDS				0.60 (2)	bZIP transcription factor^#^	[26,28]	N
miR167	GRMZM2G028980	CDS		77.00 (0)			Auxin response factor^#^	[26]	N
miR167	GRMZM2G089640	CDS		74.00 (0)		40.50 (0)	Auxin response factor^#^	[26,30]	N
miR167	GRMZM2G078274	CDS			6.60 (2)	147.80 (0)	Auxin response factor^#^	[26,30]	N
miR167	GRMZM2G475882	CDS			6.60 (2)		Auxin response factor^#^	[26,28]	N
miR169	GRMZM5G829103	3′-UTR	32.00 (2)	1923.40 (0)	24.60 (2)	3.40 (2)	CCAAT-binding transcription factor	[26,28]	N
miR169	GRMZM2G165488	3′-UTR	7.25 (2)	2804.25 (0)	8.25 (2)	1.25 (2)	CCAAT-binding transcription factor^#^	[26,28,30]	[30]
miR169	GRMZM2G037630	3′-UTR	12.00 (2)	13.00 (2)		2.00 (2)	CCAAT-binding transcription factor	[26,28]	N
miR169	GRMZM2G000686	3′-UTR	3.60 (2)	21.80 (0)	2.70 (2)	0.10 (4)	CCAAT-binding transcription factor^#^	[26,28]	N
miR169	GRMZM5G853836	3′-UTR	21.00 (2)	50.00 (2)	27.50 (2)		unknown	[26,28]	N
miR169	GRMZM5G857944	3′-UTR	129.67 (0)	139.00 (0)	67.33 (0)	16.00 (0)	CCAAT-binding transcription factor	[26,28]	N
miR169	GRMZM2G038303	3′-UTR	17.00 (2)	4.00 (2)	23.00 (0)	12.00 (0)	CCAAT-binding transcription factor^#^	[28,30]	N
miR169	GRMZM5G849099	3′-UTR	4.00 (2)				unknown	N	N
miR169	GRMZM2G409430	CDS	3.50 (0)				unknown	N	N
miR169	GRMZM2G091964	3′-UTR	138.67 (2)	257.33 (0)	27.00 (2)	99.33 (0)	CCAAT-binding transcription factor^#^	[26,28,30]	[30]
miR169	GRMZM2G078124	CDS/3′-UTR		1.00 (2)			Molluscan rhodopsin C-terminal tail^#^	[28,30]	[30]
miR171	GRMZM2G418899	CDS	1.80 (2)	80.40 (0)	4.60 (2)	0.60 (2)	GRAS transcription factor^#^	[26,29]	N
miR171	GRMZM2G037792	CDS	1.80 (3)	80.40 (0)	4.60 (3)	0.60 (3)	GRAS transcription factor^#^	[26,28,29]	[25]
miR171	GRMZM5G825321	CDS	1.80 (2)	80.40 (0)	4.60 (3)	0.60 (2)	GRAS transcription factor	[26,28]	N
miR171	GRMZM2G110579	CDS	0.33 (4)	35.67 (2)	2.67 (3)	1.67 (2)	GRAS transcription factor	[26,28]	N
miR171	GRMZM2G098800	CDS	1.80 (2)	29.67 (0)	2.67 (3)	1.67 (2)	GRAS transcription factor^#^	[26,28,29]	N
miR172	GRMZM2G176175	CDS	1.40 (3)	9.20 (0)	6.40 (0)	1.80 (0)	AP2 transcription factor (sid1)^#*^	[28,30]	[32,33]
miR172	GRMZM5G862109	CDS/3′-UTR	1.40 (3)	80.20 (2)	8.73 (2)	1.80 (2)	AP2 transcription factor (ids1)^#*^	[28]	[32,33]
miR172	GRMZM2G138676	CDS	0.20 (4)				AP2 transcription factor	N	N
miR172	GRMZM2G174784	CDS/3′-UTR	1.00 (3)	3.00 (2)	15.00 (2)		AP2 transcription factor^#^	[28]	N
miR172	GRMZM2G076602	CDS		10.00 (0)	13.00 (0)		AP2 transcription factor^#^	[30]	N
miR172	GRMZM5G879527	3′-UTR				2.50 (0)	AP2 transcription factor	N	N
miR172	GRMZM2G160730	3′-UTR				0.50 (4)	Glossy15^#*^	[28,30]	[36]
miR319	GRMZM2G412073	CDS	4.00 (3)				unknown^#^	N	N
miR319	GRMZM2G028054	CDS	5.00 (2)	2.00 (2)		7.33 (2)	MYB domain transcription factor^#^	[26,28]	N
miR319	GRMZM2G180568	CDS		3.00 (0)	17.00 (1)		unknown	N	N
miR319	GRMZM2G020805	CDS		1.00 (4)			unknown	[26,28]	N
miR319	GRMZM2G089361	CDS		5.00 (2)	8.33 (0)	88.00 (0)	TCP Transcription factor^#^	[26,28,30]	N
miR319	GRMZM2G115516	CDS		5.00 (2)	8.33 (2)	88.00 (0)	TCP Transcription factor^#^	[26,28]	N
miR319	GRMZM2G109843	CDS				53.33 (0)	MYB domain transcription factor	[30]	N
miR319	GRMZM2G056612	CDS				70.33 (0)	TCP Transcription factor	N	N
miR319	GRMZM2G015037	CDS				0.33 (4)	MYB domain transcription factor	[26]	N
miR319	AC205574.3_FG006	CDS				0.33 (4)	TCP Transcription factor	[28]	N
miR390	GRMZM2G124744	3′-UTR		35.33 (0)			Inorganic pyrophosphatase	[28]	N
miR390	GRMZM2G155490	3′-UTR			56.00 (0)		unknown^#^	N	N
miR390	GRMZM5G806469	3′-UTR			13.00 (2)		unknown	[28]	N
miR390	GRMZM2G304745	CDS			16.00 (1)		Leucine-rich repeat^#^	N	N
miR393	GRMZM2G135978	CDS		7.00 (2)	10.00 (2)		Transport inhibitor response 1-like	[26,28]	N
miR394	GRMZM2G119650	CDS	3.00 (3)	20.00 (2)		3.00 (2)	Cyclin-like F-box^#^	[26]	[28]
miR394	GRMZM2G064954	CDS		18.00 (2)	14.00 (2)	13.00 (2)	Cyclin-like F-box^#^	[26,30]	N
miR395	GRMZM2G149952	CDS		1.00 (4)			ATP-sulfurylase^#^	[26,28,30]	N
miR396	GRMZM2G018414	CDS	2.50 (2)			447.50 (0)	Glutamine-Leucine-Glutamine^#^	N	N
miR396	GRMZM2G099862	CDS	5.00 (2)		2.25 (3)	30.75 (0)	Glutamine-Leucine-Glutamine^#^	[30]	N
miR396	GRMZM2G098594	CDS	1.00 (2)			0.33 (3)	Glutamine-Leucine-Glutamine^#^	N	N
miR396	GRMZM2G119359	CDS	1.00 (3)			0.33 (3)	Glutamine-Leucine-Glutamine	N	N
miR396	GRMZM2G478709	CDS	8.00 (2)	0.50 (4)	4.00 (3)		SKI-interacting protein SKIP^#^	N	N
miR396	GRMZM2G124566	CDS		0.33 (4)			Growth-regulating factor^#^	N	N
miR396	GRMZM2G045977	CDS		0.33 (4)			Growth-regulating factor^#^	N	N
miR396	GRMZM2G041223	CDS		0.33 (4)			Putative growth-regulating factor 6^#^	[30]	N
miR396	GRMZM2G129147	CDS		0.33 (4)			Growth-regulating factor (GDF5)^#^	[30]	N
miR396	GRMZM2G178261	CDS		0.50 (3)			Growth-regulating factor 1 (GDF1)^#^	[28,30]	N
miR396	GRMZM2G443903	CDS		0.50 (3)			K Homology^#^	[30]	N
miR396	GRMZM2G033612	CDS			16.00 (2)	637.00 (0)	Glutamine-Leucine-Glutamine	N	N
miR397	GRMZM2G094699	CDS			5.00 (2)		unknown	N	N
miR398	AC234183.1_FGT002	CDS				17.00 (0)	unknown	N	N
miR399	GRMZM2G153087	CDS	1.00 (4)				PHD finger protein	N	N
miR399	GRMZM2G082384	CDS			2.25 (3)		Mrp, conserved site;ATPase-like	N	N
miR529	GRMZM2G318882	5′-UTR	0.03 (1)				SBP-box transcription factor	N	N
miR529	GRMZM2G136158	CDS	0.03 (1)	0.04 (1)	0.01 (1)	0.001 (1)	SBP-box transcription factor	N	N
miR529	GRMZM2G031501	5′-UTR	0.20 (4)				unknown	[26,30]	N
miR529	GRMZM5G878561	CDS		1.50 (3)		1.50 (2)	SBP-box transcription factor	[26]	N
miR529	GRMZM2G031983	5′-UTR		0.04 (1)	0.01 (1)		SBP-box transcription factor	N	N
miR529	GRMZM2G371033	CDS		1.50 (3)		1.50 (2)	SBP-box transcription factor	[26]	N
miR529	GRMZM2G163813	CDS/3′-UTR		0.33 (4)			SBP-box transcription factor^#^	[26]	N
miR529	GRMZM2G149022	CDS			0.01 (1)		unknown	N	N
miR529	GRMZM2G084947	5′-UTR			0.01 (1)		unknown	N	N
miR529	GRMZM2G169121	3′-UTR			0.01 (1)		unknown	N	N
miR529	GRMZM2G062052	5′-UTR			0.01 (1)		ZCN19 protein	N	N
miR529	GRMZM2G148074	5′-UTR			0.01 (1)		Transcription factor, K-box	N	N
miR529	GRMZM2G031870	5′-UTR			0.01 (1)		SBP-box transcription factor	N	N
miR529	GRMZM2G096234	3′-UTR			0.01 (1)		Non-specific lipid-transfer protein	N	N
miR529	GRMZM2G141955	5′-UTR			0.01 (1)		SBP-box transcription factor	N	N
miR529	AC233899.1_FGT004	5′-UTR			0.01 (1)		homeodomain-leucine zipper transcription factor	N	N
miR529	GRMZM2G165355	CDS			0.01 (1)		zinc finger protein 7	N	N
miR529	GRMZM2G131280	3′-UTR				0.001 (1)	SBP-box transcription factor	N	N
TAS3	GRMZM2G056120	CDS	0.50 (3)	1.25 (3)	11.25 (2)	5.00 (2)	DNA binding	N	N
TAS3	GRMZM5G874163	3′-UTR	0.50 (3)	1.58 (2)	11.25 (2)	5.00 (2)	DNA binding	N	N
TAS3	GRMZM2G437460	CDS		11.00 (2)	8.50 (2)	0.50 (4)	Auxin response factor	N	N
TAS3	GRMZM2G030710	CDS			1287.00 (0)	1.00 (4)	DNA binding	N	N

^a^MiRNA data from miRBase 19.0. ^b^Calculation based on the method in Addo-Quaye *et al.*[47],. ^c^Gene annotations fromB73 RefGen_v2 (release 5b. 60 in February 2011). ^*^validated in Zhai et al; Zhao et al. [28,30], ^#^Predicted by Zhang *et al*. [17]. Abbreviation: tpm, transcripts per million; CDS, coding sequence; UTR, untranslated region; siRNA, small interfering RNA.

**Table 4:** Non-conserved maize miRNA targets identified by degradome sequencing in this study

**miRNA**^**a**^	**Target gene**	**Location of the target site**	**Repeat normalized read abundance (category)**^**b**^				**Target gene annotation**^**c**^	**Other degradome evidence**	**5′-RACE or genetic experiment**
			**I**	**II**	**III**	**IV**			
miR2275	GRMZM2G044004	3′-UTR	0.33 (4)				nucleic acid binding protein	N	N
miR2275	GRMZM2G154532	5′-UTR/CDS				0.57 (2)	NAD dependent epimerase/dehydratase family protein	N	N
miR2275	GRMZM2G329532	CDS				1.00 (1)	unknown	N	N
miR2118	GRMZM2G066406	5′-UTR			16.00 (1)	2.00 (1)	unknown	N	N
miR2118	GRMZM2G031788	CDS				2.00 (1)	unknown	N	N
miRs4	GRMZM2G078124	CDS/3′-UTR		1.00 (2)			Molluscan rhodopsin C-terminal tail	N	N
miRs4	GRMZM5G829103	3′-UTR		1923.40 (0)			CCAAT-binding transcription factor^#^	[26,28]	N
miRs4	GRMZM2G165488	3′-UTR		2804.25 (0)			CCAAT-binding transcription factor^#^	[26,28]	N
miRs4	GRMZM2G000686	3′-UTR		21.80 (0)			CCAAT-binding transcription factor^#^	[26,28]	N
miRs9	GRMZM2G467356	CDS				12.00 (0)	Ferredoxin	N	N
miRs14	GRMZM2G079683	3′-UTR	1.00 (4)				unknown	N	N
miRs15	GRMZM2G091189	3′-UTR		0.33 (4)			transcription factor	N	N
miRs17	GRMZM2G160041	CDS	0.25 (4)			1.75 (0)	DNA binding protein	N	N
miRs17	GRMZM2G085550	3′-UTR			5.00 (1)		unknown	N	N

^a^MiRNA data from miRBase 19.0. ^b^Calculation based on the method in Addo-Quaye et al. [47], ^c^Gene annotations fromB73 RefGen_v2 (release 5b. 60 in February 2011). ^#^Predicted by Zhang et al. [17]. *Abbreviation*: tpm, transcripts per million; CDS, coding sequence; UTR, untranslated region; siRNA, small interfering RNA.

We identified target genes for almost all of the 22 conserved miRNA families. The conserved miRNAs were able to target various numbers of genes, ranging from 1 to 18. Among the conserved miRNA families, zma-miR156 and zma-miR529 had the highest number of gene targets. zma-miR156 targeted 13 unique genes including *SPL* genes and zma-miR529 targeted 18 unique genes including *ZCN19* (a possible maize FT ortholog) (Table 3), indicating that these two families might play key roles in ear development [31,54]. Most of the conserved miRNAs targeted multiple gene loci. Their gene targets were members of different families of transcription factors, such as SBP-box transcription factor, AUXIN RESPONSE FACTOR (ARF), TCP, MYB, bZIP, AP2, and GRAS. We also identified 57 new target genes of conserved miRNAs in maize (Table 3). Among the 127 miRNA target genes, 67 (53%) had been predicted previously [17], 56 (44%) cross-validated with other degradome libraries prepared from plants under different stress conditions [25,28-30], and 8 have been validated by 5′-RACE and/or genetic experiments [25,28,30,32,33,36]. The targets of conserved miRNAs were highly abundant in the four sequenced target libraries, and were often classified as category 0, 1, or 2 targets (Table 3, Additional file 11). For instance, miR169 targeted seven different *CCAAT*-binding transcription factors in the four stages (category 0 or 2) with very high abundance, but it also guided the slicing of three other non-conserved targets with very low abundance. Interestingly, some target transcripts were regulated by pairs of miRNAs: both miR156 and miR529 targeted five members of the same *SBP* family, and the miR159/319 pair regulated three *MYB* domain transcription factors. This result suggested that there is complex regulation of these genes by these miRNA pairs, consistent with the findings of a previous study [49] (Table 3).

Out of 26 non-conserved zma-miRNAs including 21 new miRNAs with four corresponding miRNA*, we identified targets for only seven miRNAs (miR2118, miR2775, miRs4, miRs9, miRs14, miRs15 and miRs17). We used absolute numbers to plot the cleavages on target mRNAs; this was referred to as a target plot (t-plot) by German *et al.*[48]. Except for miRs4, the targets mostly belonged to category 2 or 4 with very low abundance, which differed from the targets of conserved miRNAs (Tables 3, 4). Four identified targets of miRs4 (category 0 or 2) were the same as those of miR169, providing further evidence that miRs4 is a member of the miR169 family.

### GO analysis of targets regulated by differentially expressed miRNAs

In our study, we predicted 72 genes (117 transcripts) for 62 differentially expressed miRNAs from 11 miRNA families. More than 90% of these miRNAs had putative functions (Tables 2, 3 and Additional file 13). 73% of these differentially expressed miRNA families played an important role in post-transcriptional regulation by targeting mRNAs encoding transcription factors in *SBP*, *ARF*, *GRAS*, and *AP2* families (Tables 2, 3 and Additional file 13). GO analysis revealed that the target genes were involved in a wide spectrum of regulatory functions and biological processes including regulation of transcription, DNA binding, response to hormone stimulus, nucleic acid metabolic process, gene expression, cellular macromolecule synthesis, and cellular nitrogen compound metabolism. This was consistent with the fact that the small RNA and the degradome libraries for miRNA sequence analysis were constructed from developing maize ears (Additional file 14). In addition, a specific feature of our study was that we found more genes in families involved in metabolic process, biological regulation, cellular biosynthetic process, and nucleic acid binding function at the later stages of maize ear development (Table 2, Additional file 14). The accumulation of dry matter such as starch and saccharides is the main event in ear development, and a large number of target genes may participate in this pathway. The differentially expressed miRNAs may regulate expression of these target genes to control ear development and biomass yield in maize.

## Discussion

Small RNAs play important roles in gene regulation in plants [12]. In this study, we have annotated miRNA genes based on the complete assembly of the maize genome. In total, 98 known miRNAs and 26 new miRNAs were identified in maize ears by deep sequencing. This confirmed previous results reported by Zhang et al. [17]. These newly identified miRNAs may belong to lineage-specific families, and showed little or no expression at the miRNA level. We identified 62 miRNAs as differentially expressed miRNAs by microarray assays.

The recently reported high-throughput experimental approach [47,48] allowed us to create a detailed miRNA: target interaction atlas for maize. In the current work, we identified a total of 131 genes (245 transcripts) targeted by 102 small RNAs including 98 miRNAs and 4 ta-siRNAs (Tables 3, 4 and Additional file 12). Among the 131 genes, 54 were cross-validated in other degradome libraries [25,28-30], by 5′-RACE, and/or by genetic experiments [17,25,30,32,36], showing that degradome sequencing is a powerful tool for identifying targets regulated by miRNAs. Surprisingly, most highly conserved miRNAs were detectable in maize ears at all four developmental stages (Additional file 8), but sliced targets were not detected at all stages (Additional file 12). It is possible that the differentially expressed miRNAs regulate both the spatial pattern and the level of target mRNA expression, as previously demonstrated in some cases [55,56]. It is equally possible that this represents a limitation of degradome sequencing. Results can be affected by many unpredictable factors such as ligation efficiency, PCR bias, etc. There were 127 target genes of 22 conserved miRNA families. Among the target genes, 72.4% encoded transcription factors (Table 3). These targets were not only conserved families, such as *SBP*, *MYB*, *ARF*, *bZIP* (basic-leucine Zipper), *NAC*, *GRAS*, *AP2*, and *TCP* transcription factor gene families, but also non-conserved genes encoding metallophosphoesterase, *DICER-LIKE1,* No Apical Meristem (*NAM*) proteins, and *PHD* finger proteins. The conserved targets may participate in maize ear development. We also identified 13 genes targeted by non-conserved miRNAs. One *ARF* gene and three DNA-binding transcription factor genes cleaved by ta-siRNAs were also identified (Table 4). The conserved miRNAs silenced more targets than did maize-specific miRNAs. It is possible that conserved miRNAs play a crucial role in post-transcriptional regulation in different plant species [12]. However, maize-specific miRNAs may function only to regulate gene expression during gramineae- or maize-specific biological processes. Although conserved miRNAs mainly regulate genes encoding transcription factors, maize-specific miRNAs are considered to be young miRNAs that have evolved recently, and are often expressed at lower levels than conserved miRNAs [39,57].

**Figure 4:**
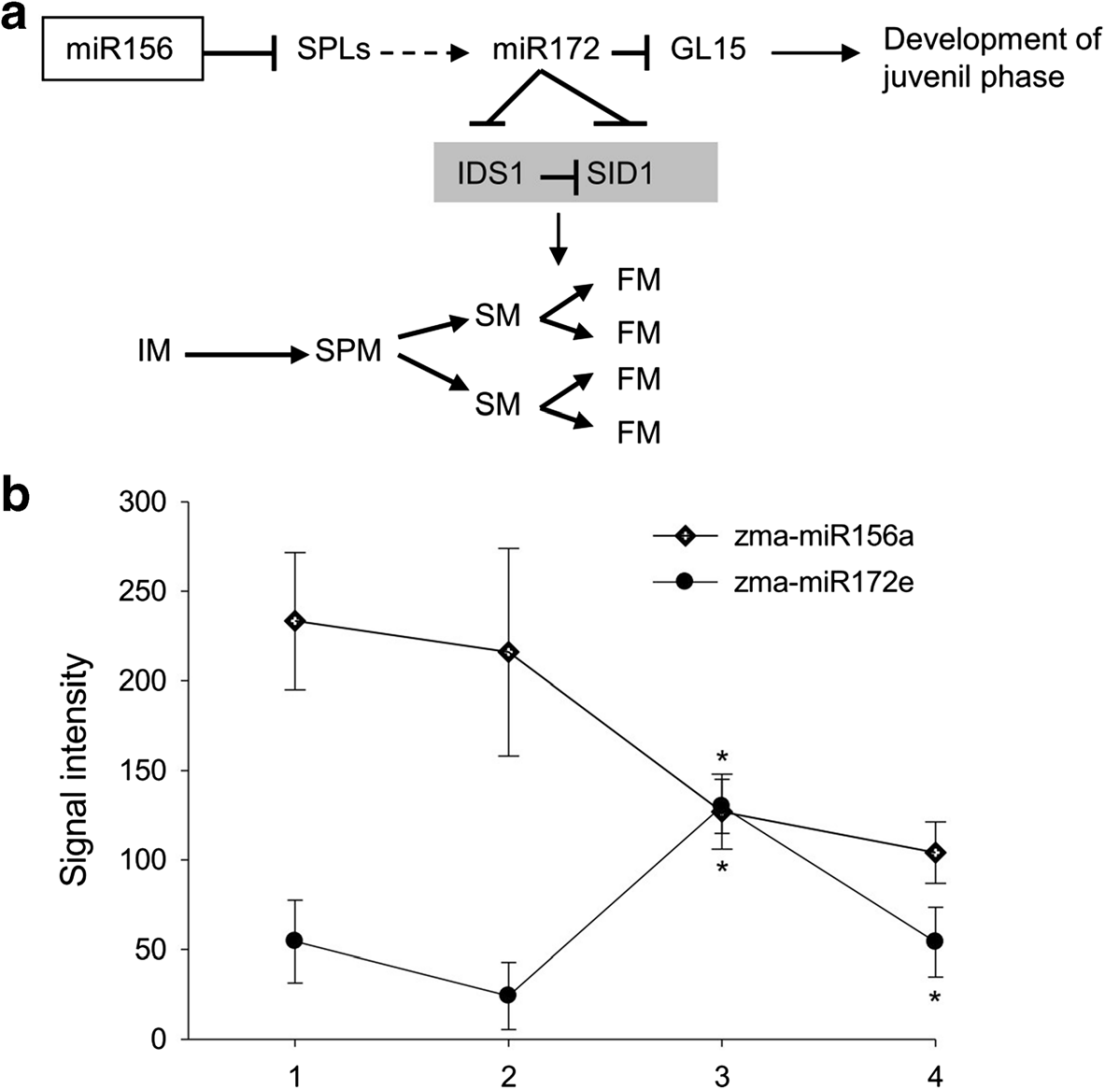
**miR156 and miR172 in maize flower development (Adapted from Poethig (2009). (a)** The development of the maize ear (female inflorescence) and the juvenile phase regulated by miR156 and miR172. In the ear, the inflorescence meristem (IM, stage I) directly gives rise to spikelet pair meristems (SPM, stage II). SPMs give rise to two spikelet meristems (SM, stage III), which subsequently form two floral meristems (FM, stage IV). miR172 and its targets *IDS1* and *SID1* function to influence the SM to FM conversion. *IDS1* is also a negative regulator of its homolog *SID1*. Both *IDS1* and *SID1* are positive regulators of the SM to FM transition. **(b)** The expression pattern of zma-miR156a and zma-miR172e. * indicated the significant difference in adjacent two developmental stages (one-way ANOVA and Fisher’s *LSD*, *p* < 0.01, *n* = 4).

Previous studies showed that miR156 and miR172 function throughout flower development from the earliest stages (floral induction, stage I) to very late stages (floral organ cell-type specification, stage IV) [31-34]. miR156a-l probably targets several SPL genes during the juvenile-to-adult phase transition in maize (Figure 4a, Tables 2 and 3), and is postulated to indirectly activate miR172 via *SPL*[31]. miR172 has been shown to negatively regulate GL15 (Table 3), which promotes maintenance of the juvenile state [31]. The levels of miR156 and miR172 are conflicting during phase transition (Figure 4b). Meanwhile, miR172e likely controls *IDS1* and *SID1*, which are responsible for maize spikelet sex determination and meristem cell fate [32-34], by both translational repression and mRNA degradation (Table 3; Figure 4a). Beyond miR156 and miR172, miR164 targets genes encoding *NAM* proteins, and may be involved in regulating ear development (Table 3), similar to how miR164 is postulated to regulate *NAC*-domain targets in Arabidopsis [58]. Although most miRNA families appear to target a single class of targets, the miR159/319 family regulates both *MYB* and *TCP* transcription factors, which may control petal morphogenesis as previously reported [59].

Some miRNAs have been shown to be involved the signaling pathway that mediates responses to the phytohormone auxin. For example, miR167 targets four *AUXIN RESPONSE FACTOR* (*ARF*) genes, and miR160 targets six *ARF* genes. In addition to the miRNAs mentioned above, one miRNA family (miR162) targets a gene central to miRNA genesis; the differentially expressed zma-miR162 targets *DICER-LIKE1* (*DCL1*), a homolog of *DCL1* in *Arabidopsis* that is required for miRNA accumulation [60]. In summary, genome-wide identification of all targets provided useful information to explore the functions of miRNAs in maize.

## Conclusions

In this study, we have confirmed the expression of conserved, known non-conserved and new maize miRNAs using high-throughput approaches to better understand the role of miRNAs in developmental maize ears. Besides, we have identified 131 target genes of both known and new miRNAs and ta-siRNAs using recently developed tools for the global identification of miRNA targets. Specifically, 72 genes (117 transcripts) targeted by 62 differentially expressed miRNAs from 11 miRNA families may play important roles in ear development in maize. Maize represents a model for cultivated crop plants. As these characters are quite different for other model plants (e.g. Arabidopsis and Medicago), we expect to discover new roles of miRNAs in post-transcriptional regulation. We also provided some evidence of the important function of miRNAs in regulating developmental process. Identification and characterization of this important class of regulatory genes in maize may improve our understanding of molecular mechanism controlling maize ear development.

## Methods

### Plant materials and RNA isolation

Seed of maize inbred lineB73 was first sterilized and germinated in an incubator, then grown in a controlled environment at 28°C/21°C (day/night) under a 16-h day/8-h night photoperiod with a relative humidity of 70%. Ear development can be divided into four stages: the growth point elongation phase, spikelet differentiation phase, the floret primordium differentiation phase and floret organ differentiation phase. Plant materials (ears) were collected as described previously [48]. Briefly, ears were manually collected at the four developmental stages according to the plant features (number of visible leaves, leaf age index, and number of unfolded and folded leaves) combined with microscopic observation. All the samples were harvested and immediately frozen in liquid nitrogen and stored at −80°C. The total RNA from each sample was then isolated using Trizol (Invitrogen, Carlsbad, CA) according to the manufacturer’s instructions.

### Small RNA library preparation and sequencing

The total RNAs were pooled for each of four developmental stages for Solexa sequencing. After small RNA cloning, the sequencing procedures were conducted as described previously [61]. In brief, sequencing was performed as follows: approximate 100 ug of total RNA was purified by polyacrylamide gel electrophoresis (PAGE), to enrich for molecules in the range of 18–30nt, and ligated with adapters to the 5′ and 3′ terminals of the RNA. Then, small RNA molecules were used as templates for cDNA synthesis. In total, 18 PCR cycles and agarose gels were used for amplification and fragments of around 90 nt including both small RNA and adaptors, separately. The purified DNA was used Solexa sequence analysis performed by the Illumina platform. Digital-quality data were generated from the image files produced by the sequencer. After quality control using common pipeline, clean reads were directly used for further bioinformatics analysis.

### Degradome library construction

Small cDNA libraries using the sliced ends of poly adenylated transcripts from maize ears of four developmental stages were constructed according to previous reports [47,48]. By using the Oligotex kit (Qiagen, location), 200 μg of total RNA was used for extracting poly (A) RNA, which were ligated with an RNA adapter consisting of a *MmeI* recognition site in its 3′ end. After ligation, first-strand cDNA was generated using oligod (T) and the PCR product was amplified using five PCR cycles. The PCR product was purified and digested with *MmeI*. The digested PCR product was then ligated to a double-stranded DNA oligonucleotide with degenerate nucleotides at the 5′-or 3′-ends. The ligation product was further gel purified and amplified using 10 PCR cycles. The final PCR product was purified and sequenced using Illumina’s sequencing by synthesis (SBS) sequencing technology.

### MiRNA microarray assays

MiRNA microarray assays of different developmental stages were performed by LC Sciences (Houston, TX, USA). The custom μparaflo™ microfluidic chip contained 632 unique plant miRNAs of release version 18, representing 1,187 miRNAs (including miRNA*) from 4 plant species (http://microrna.sanger.ac.uk/) [13], and 26 additional unique miRNAs of maize identified by Solexa sequencing, representing 26 novel miRNAs. Each chip contained four repetitions of each probe. In total, the 1,215 miRNAs were composed of 224, 496, 148 and 347 miRNAs from *Arabidopsis thaliana*, *Oryza sativa*, *Sorghum bicolor* and *Zea mays*, separately. RNA labeling, microarray hybridization, array scanning, and data’s analysis were performed essentially as previously described [46].

### Bioinformatics analysis of sequencing data

Both small RNA reads and degradome reads were generated by Illumina Genome Analyzer II. As for the small RNA library, the data were processed and analyzed as previously described by Wang *et al.*[27] and Zhang *et al.*[17]. In brief, unique reads ranging from18-25 nt were collected and mapped to the maize genome (B73 RefGen_v2 (release 5b. 60 in February 2011)) reference sequences [62] by SOAP2 [37]. After removing sequences matching non-coding rRNAs, tRNAs, snRNAs and snoRNAs in the Rfam and NCBI Genbank databases, the matched Solexa reads that were extracted 250 nt of the sequence flanking the genomic sequences were used for RNA secondary structure prediction, which was performed by mFold 3.5 [63] and analyzed by MIREAP (https://sourceforge.net/projects/mireap/) to identify new candidates using default settings. The candidate miRNA list was further trimmed based on the criteria as described [17,42]. Based on the hairpin structure of the pre-miRNA, the corresponding miRNA star sequence was also identified.

Degradome reads were filtered using custom Perl script. The remaining distinct 20–21 nt sequences that perfectly matched maize contigs were collected for further analysis. The 15 nt upstream and 5′ end of the reads that mapped to maize contigs were extracted to generate 30-sequence tags, which were used to align to newly identified miRNAs and miRBase (Release19.0, August, 2012) using the Cleave and pipeline [52]. Alignments were collected as candidate targets if they fulfilled the criteria as described before [50].

GO functional enrichment analysis of all candidate targets during different developmental stages was carried out using Blast2GO (version 2.3.5, http://www.blast2go.org/) and GO annotations were performed using AgriGO (http://bioinfo.cau.edu.cn/agriGO/). KEGG pathway analyses of differentially expressed genes were performed using Cytoscape software (version 2.6.2) (http://www.cytoscape.org/) with the ClueGO plugin (http://www.ici.upmc.fr/cluego/cluegoDownload.shtml) [64].

### Stem-loop quantitative real-time PCR (qRT-PCR) analysis

Validations of 13 randomly selected mature miRNAs were carried out by stem-loop reverse transcription-PCR (RT–PCR) (Additional file 15). Total RNA (200 ng) was used to initiate the reverse transcription reaction. Primers for the stem–loop RT-PCR were designed using methods as described by Chen *et al.*[65] and Varkonyi-Gasic *et al.*[66]. The stem-loop RT-PCR was using the Applied Bio systems 7500 Real-Time PCR System (Applied Bio systems, Foster City, CA). All primers were listed in Additional file 12: Table S8. All reactions were run in triplicate. 5S rRNAs was used as the internal control for stem–loop RT-PCR [67].

### Data access

The RNA sequencing data have been deposited in the NCBI under the accession number GSE47837.

## Competing interests

The authors declare that they have no competing interests.

## Authors' contributions

HL, GP, TL and ZZ designed the study. HL, CQ, ZC, TZ, XY, SC, HZ, XH, YS, YZ, and MX performed the analyses. HL, CQ, TZ, XY, TL, ZZ, and GP drafted the manuscript. All of the authors critically revised and provided final approval of this manuscript.

## Supplementary Material

Additional file 1: Table S1 Summary of reads that match various RNAs. Click here for file

Additional file 2: Figure S1 Step-by-step schematic representation of the strategy for maize miRNAs discovery and validation. Click here for file

Additional file 3: Table S2 MIREAP output. Click here for file

Additional file 4: Table S3 MIREAP summary, miRAlign output and MiPred output. Click here for file

Additional file 5: Table S4 The mature sequences of maize miRNAs and their cloning frequencies. Click here for file

Additional file 6: Table S5 The detailed precursor sequences and exact genome locations of all maize miRNAs. Click here for file

Additional file 7: Figure S2 The sequence conservation of mature miRNAs between members of known miR169 family and miRs4. Click here for file

Additional file 8: Table S6 Statistic test results of miRNA microarray during ear development. Click here for file

Additional file 9: Figure S3 Clustering of differently expressed miRNA members during the process of ear development in maize. M-1, M-2, M-3 and M-4 represent stage I, stage II, stage III and stage IV, respectively. Click here for file

Additional file 10: Table S7 Summary statistics of 3′ RNA ends sequenced from maize. Click here for file

Additional file 11: Figure S4 T-plots for some miRNA and ta-siRNA targets in four developmental stages of maize ears. Click here for file

Additional file 12: Table S8 Cleaved miRNA and ta-siRNA targets in four developmental stages of maize ears. Click here for file

Additional file 13: Table S9 List of targets regulated by differentially expressed miRNAs in four developmental stages of maize ears. Click here for file

Additional file 14: Table S10 Functional classification of targets regulated by differentially expressed miRNAs based on Gene Ontology (GO). Click here for file

Additional file 15: Table S11 Primers of stem-loop qRT-PCR assay used for 12 randomly selected miRNAs in this study. Click here for file
