# Supplementary material for: Identification of miRNAs and their target genes in developing maize ears by combined small RNA and degradome sequencing
Source: BMC Genomics. 2014 Jan 14;15:25. doi: 10.1186/1471-2164-15-25 (PMC3901417; doi:10.1186/1471-2164-15-25)
Supplement: Additional file 2: Figure S1 — Step-by-step schematic representation of the strategy for maize miRNAs discovery and validation. [file 1471-2164-15-25-S2.pdf]

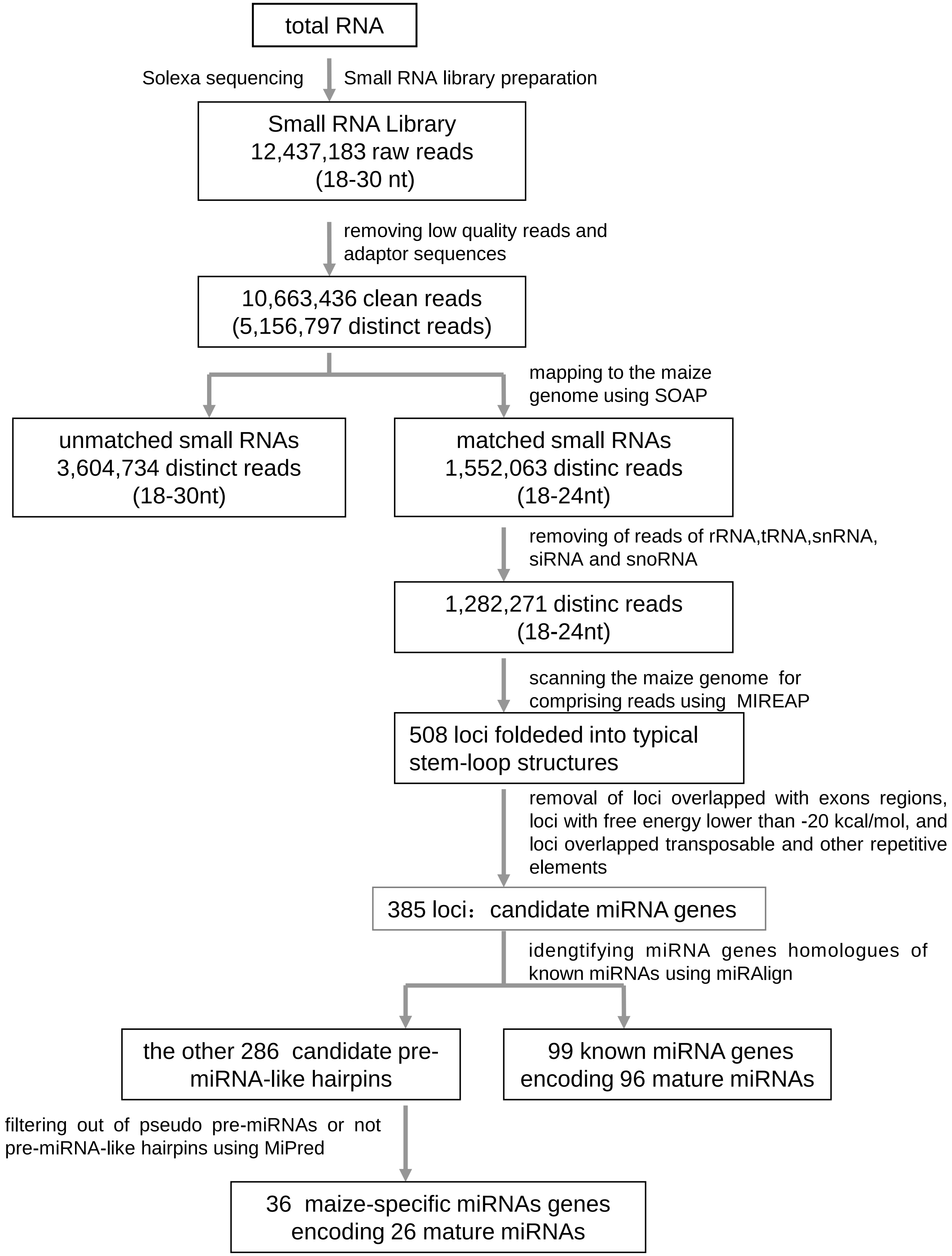

Fig. S1. Step-by-step schematic representation of the strategy for maize miRNAs discovery and validation.
