# Supplementary figures and images for: Identification of miRNAs and their target genes in developing maize ears by combined small RNA and degradome sequencing
Source: BMC Genomics. 2014 Jan 14;15:25. doi: 10.1186/1471-2164-15-25 (PMC3901417; doi:10.1186/1471-2164-15-25)

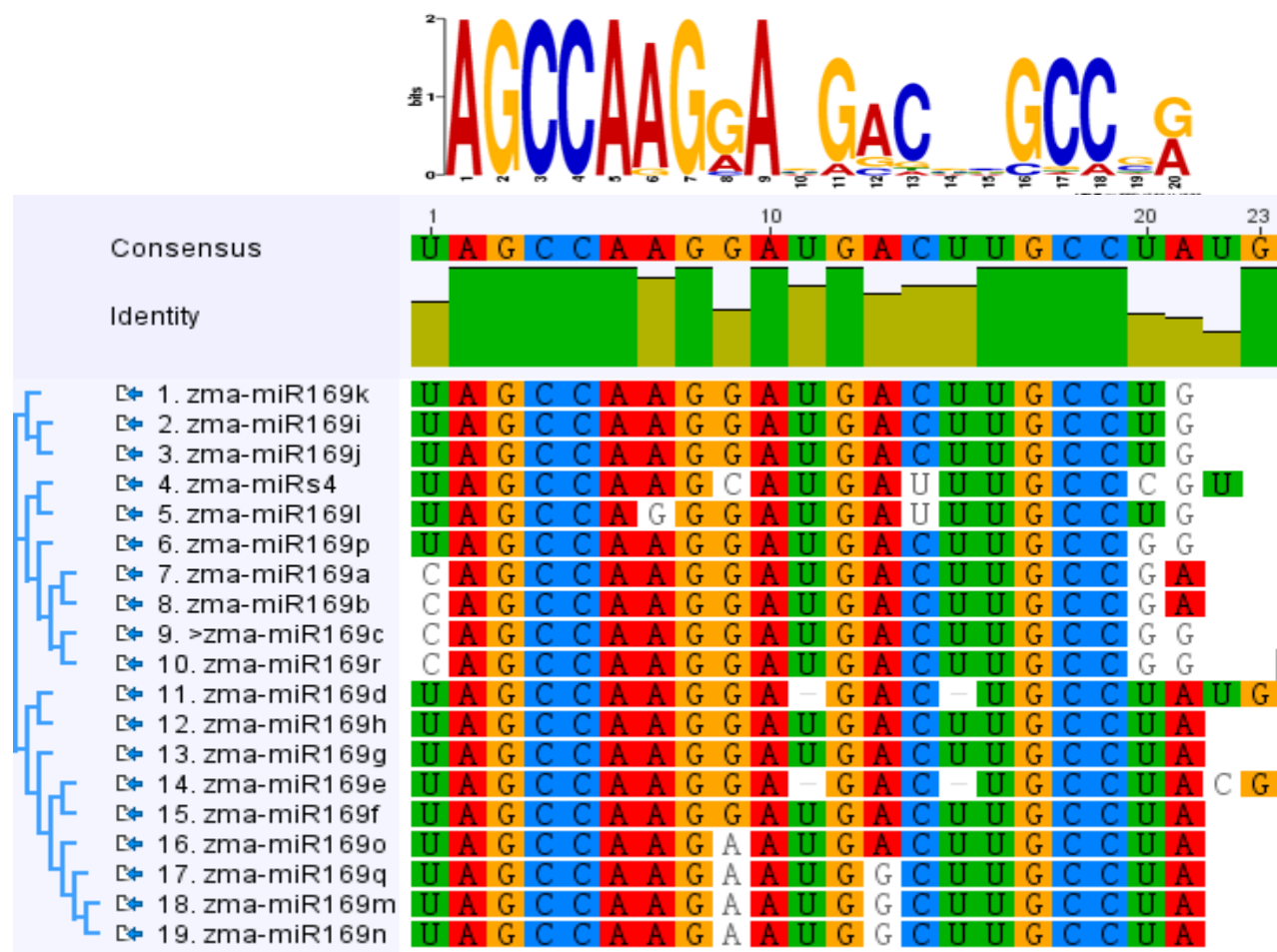

Fig. S2. The sequence conservation of mature miRNAs between members of known miR169 family and miRs4

Supplement: Additional file 7: Figure S2 — The sequence conservation of mature miRNAs between members of known miR169 family and miRs4. [file 1471-2164-15-25-S7.pdf]
