# Supplementary material for: Identification of miRNAs and their target genes in developing maize ears by combined small RNA and degradome sequencing
Source: BMC Genomics. 2014 Jan 14;15:25. doi: 10.1186/1471-2164-15-25 (PMC3901417; doi:10.1186/1471-2164-15-25)
Supplement: Additional file 9: Figure S3 — Clustering of differently expressed miRNA members during the process of ear development in maize. M-1, M-2, M-3 and M-4 represent stage I, stage II, stage III and stage IV, respectively. [file 1471-2164-15-25-S9.pdf]

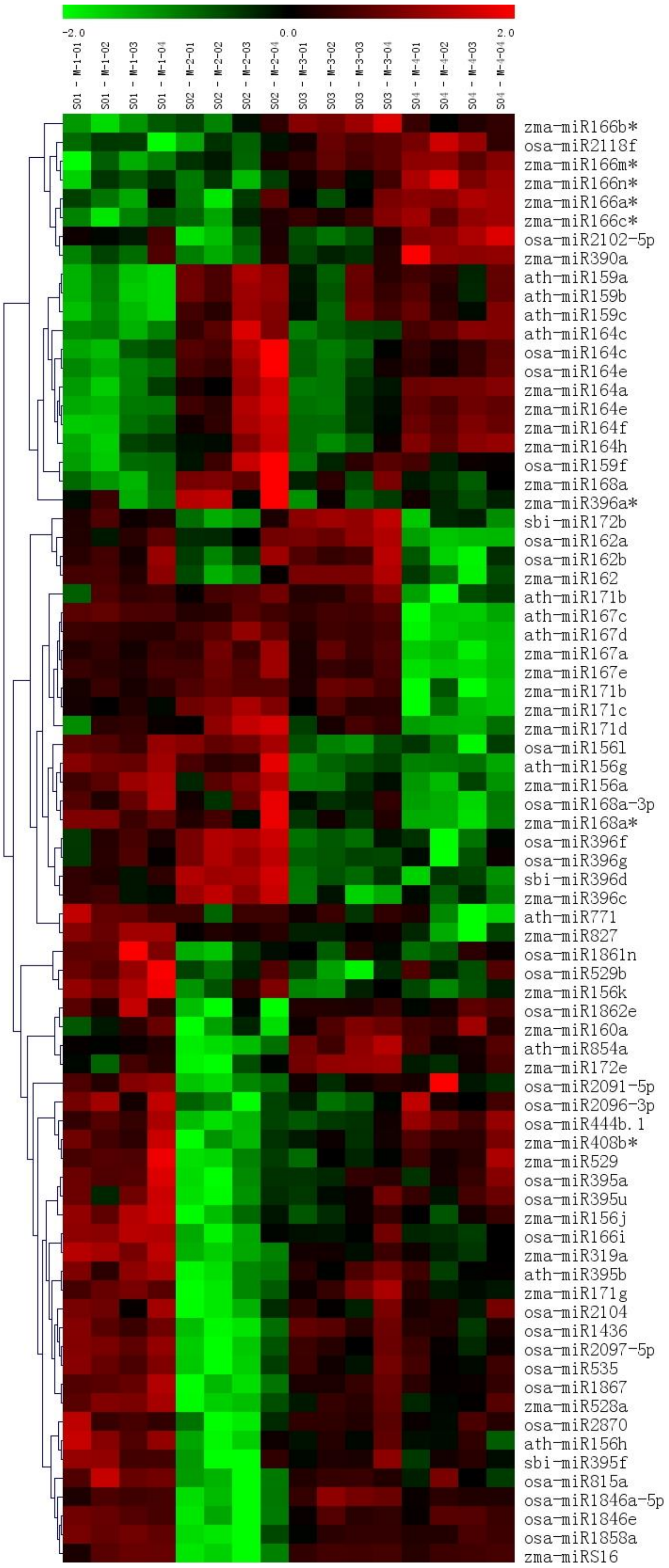

Fig. S3. Clustering of differently expressed miRNA members during the process of ear development in maize. M-1, M-2, M-3 and M-4 represent stage I, stage II, stage III and stage IV, respectively.
