# Supplementary material for: Identification of miRNAs and their target genes in developing maize ears by combined small RNA and degradome sequencing
Source: BMC Genomics. 2014 Jan 14;15:25. doi: 10.1186/1471-2164-15-25 (PMC3901417; doi:10.1186/1471-2164-15-25)
Supplement: Additional file 11: Figure S4 — T-plots for some miRNA and ta-siRNA targets in four developmental stages of maize ears. [file 1471-2164-15-25-S11.pdf]

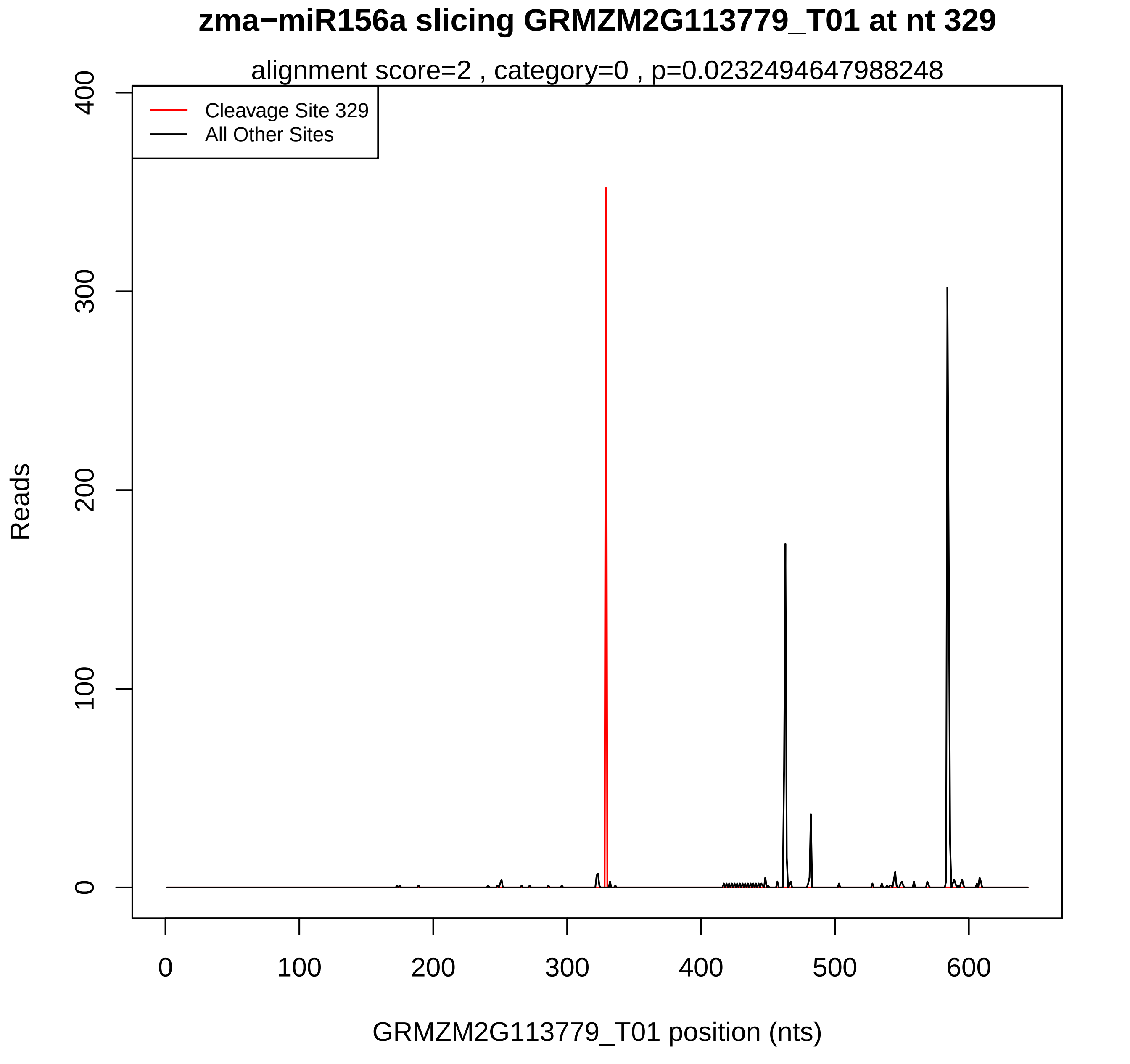

zma−miR156a slicing GRMZM2G113779_T01 at nt 329
alignment score=2 , category=0 , p=0.0232494647988248
Cleavage Site 329
All Other Sites
Reads
0
100
200
300
400
0
100
200
300
400
500
600
GRMZM2G113779_T01 position (nts)

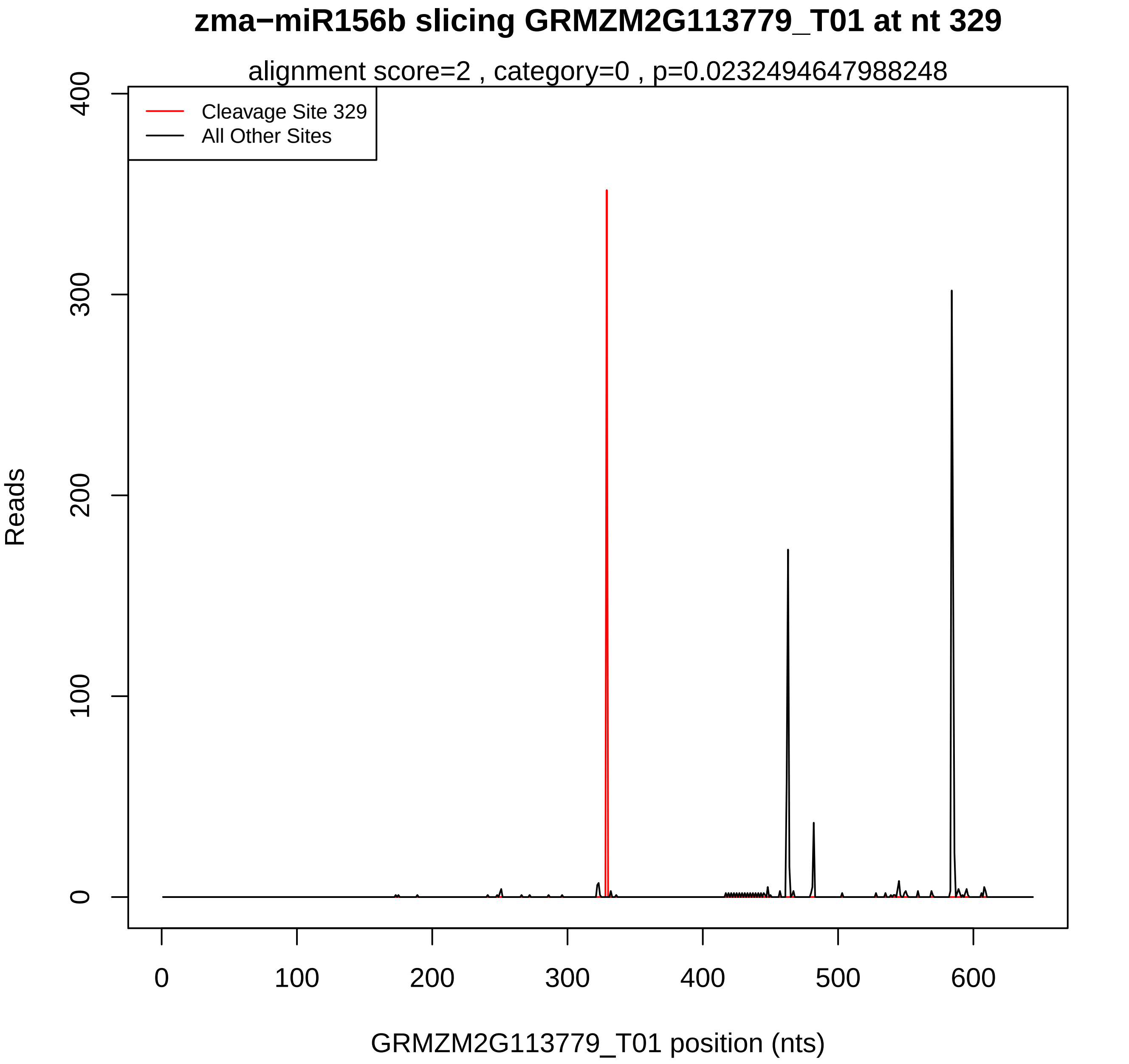

zma−miR156b slicing GRMZM2G113779_T01 at nt 329
alignment score=2 , category=0 , p=0.0232494647988248
Cleavage Site 329
All Other Sites
Reads
0
100
200
300
400
0
100
200
300
400
500
600
GRMZM2G113779_T01 position (nts)

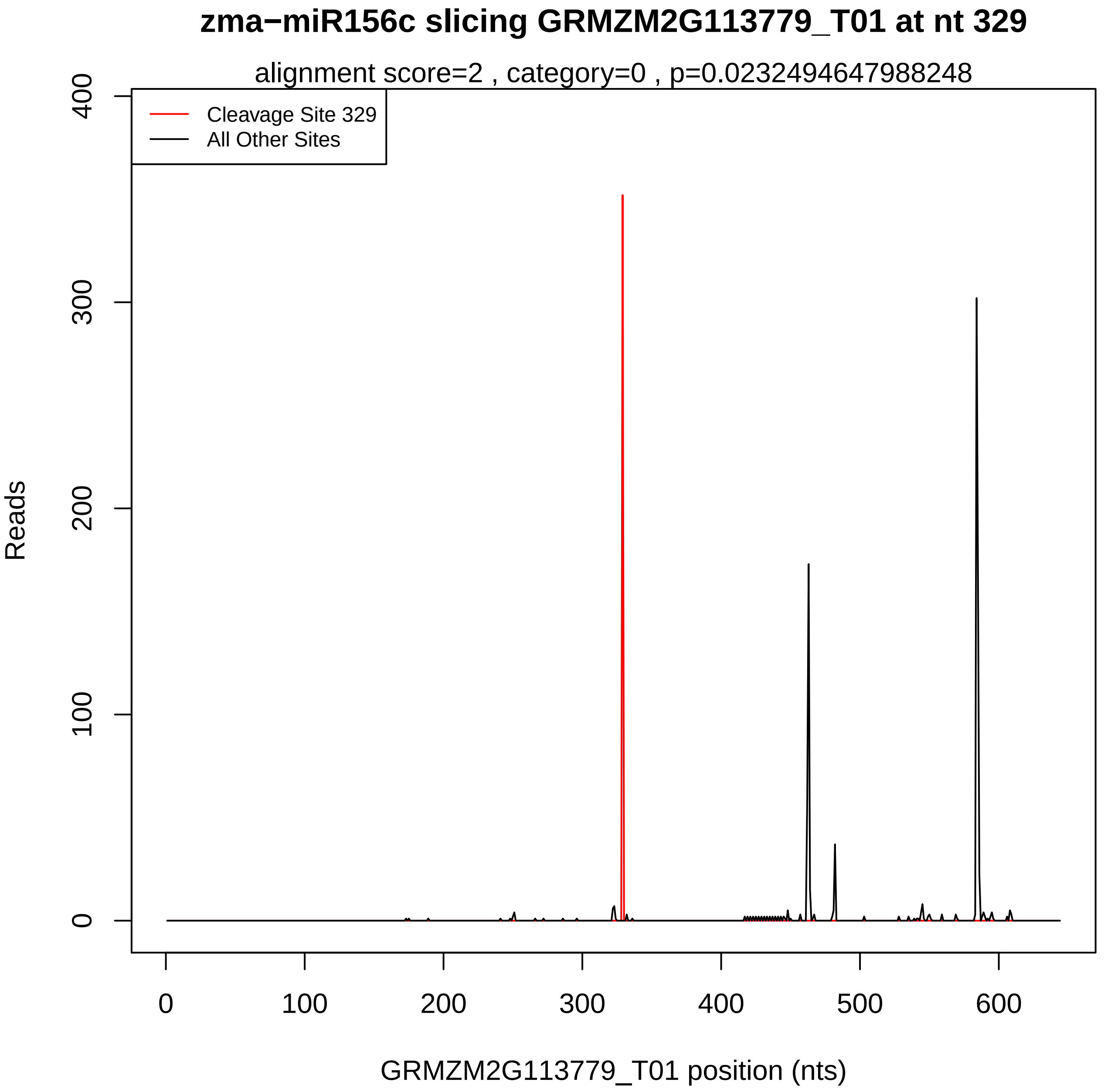
zma−miR156c slicing GRMZM2G113779_T01 at nt 329
alignment score=2 , category=0 , p=0.0232494647988248
Cleavage Site 329
All Other Sites
Reads
0
100
200
300
400
0
100
200
300
400
500
600
GRMZM2G113779_T01 position (nts)

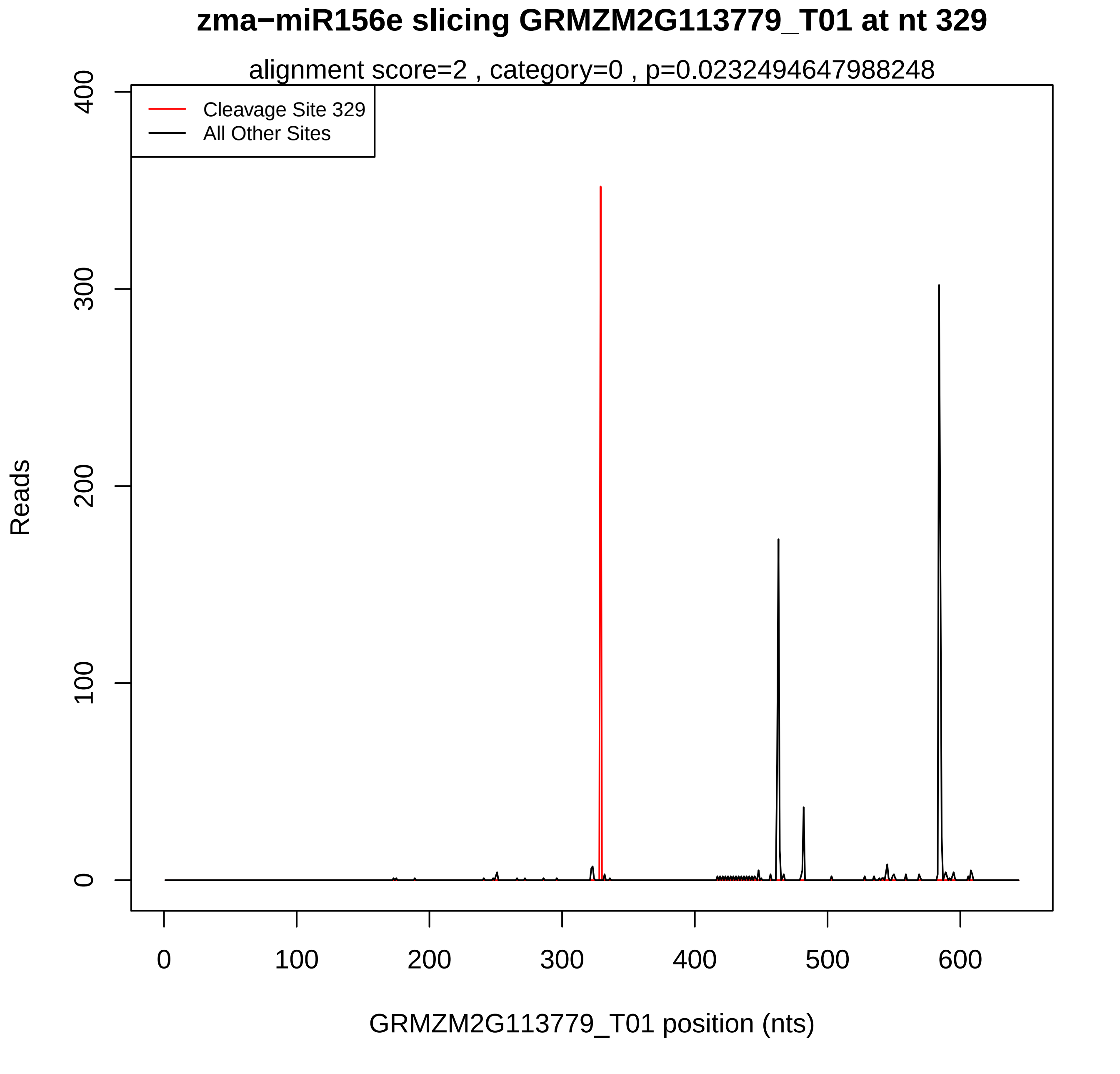

zma−miR156e slicing GRMZM2G113779_T01 at nt 329
alignment score=2 , category=0 , p=0.0232494647988248
Cleavage Site 329
All Other Sites
Reads
0
100
200
300
400
0
100
200
300
400
500
600
GRMZM2G113779_T01 position (nts)

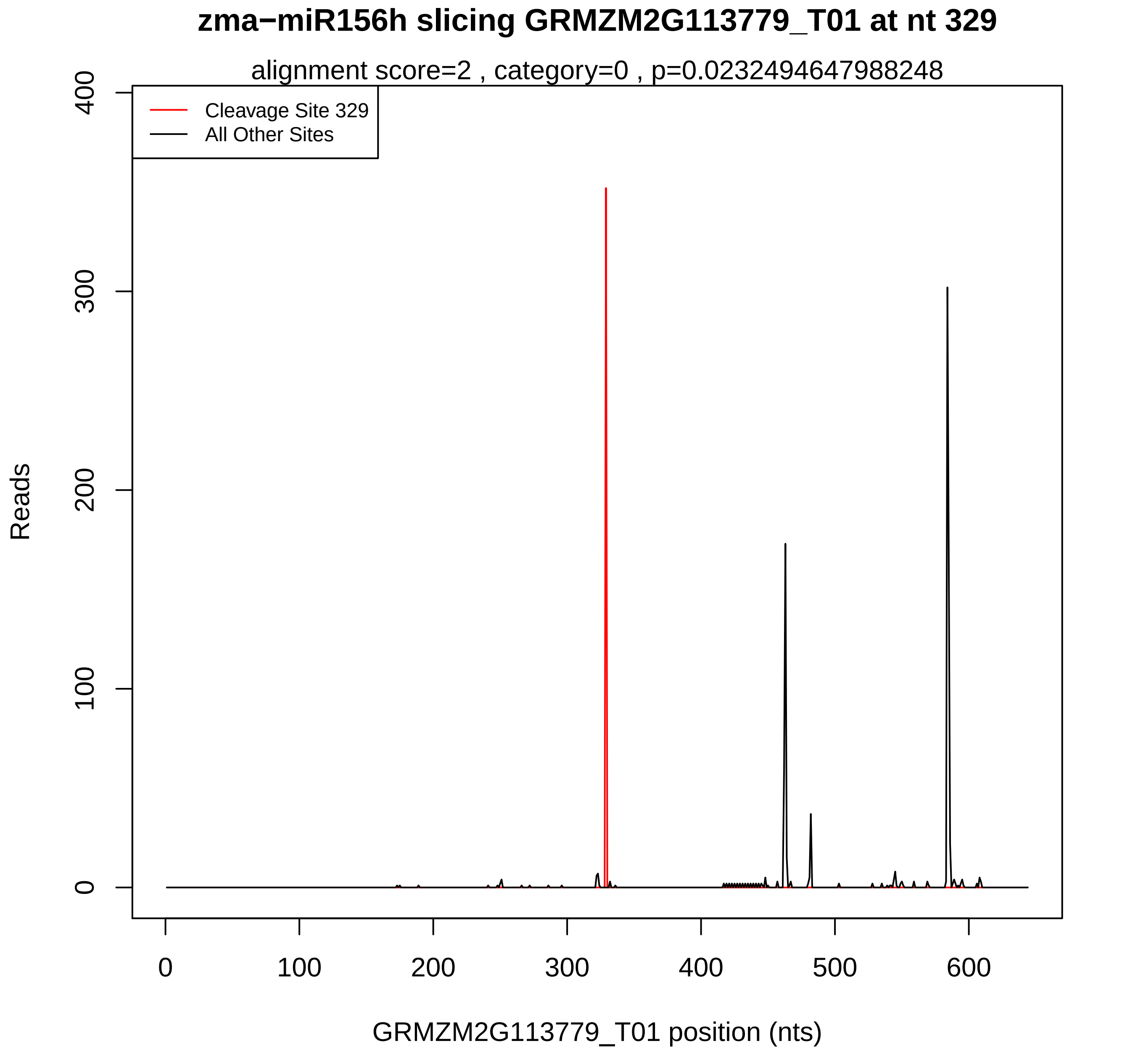

zma−miR156h slicing GRMZM2G113779_T01 at nt 329
alignment score=2 , category=0 , p=0.0232494647988248
Cleavage Site 329
All Other Sites
Reads
0
100
200
300
400
0
100
200
300
400
500
600
GRMZM2G113779_T01 position (nts)

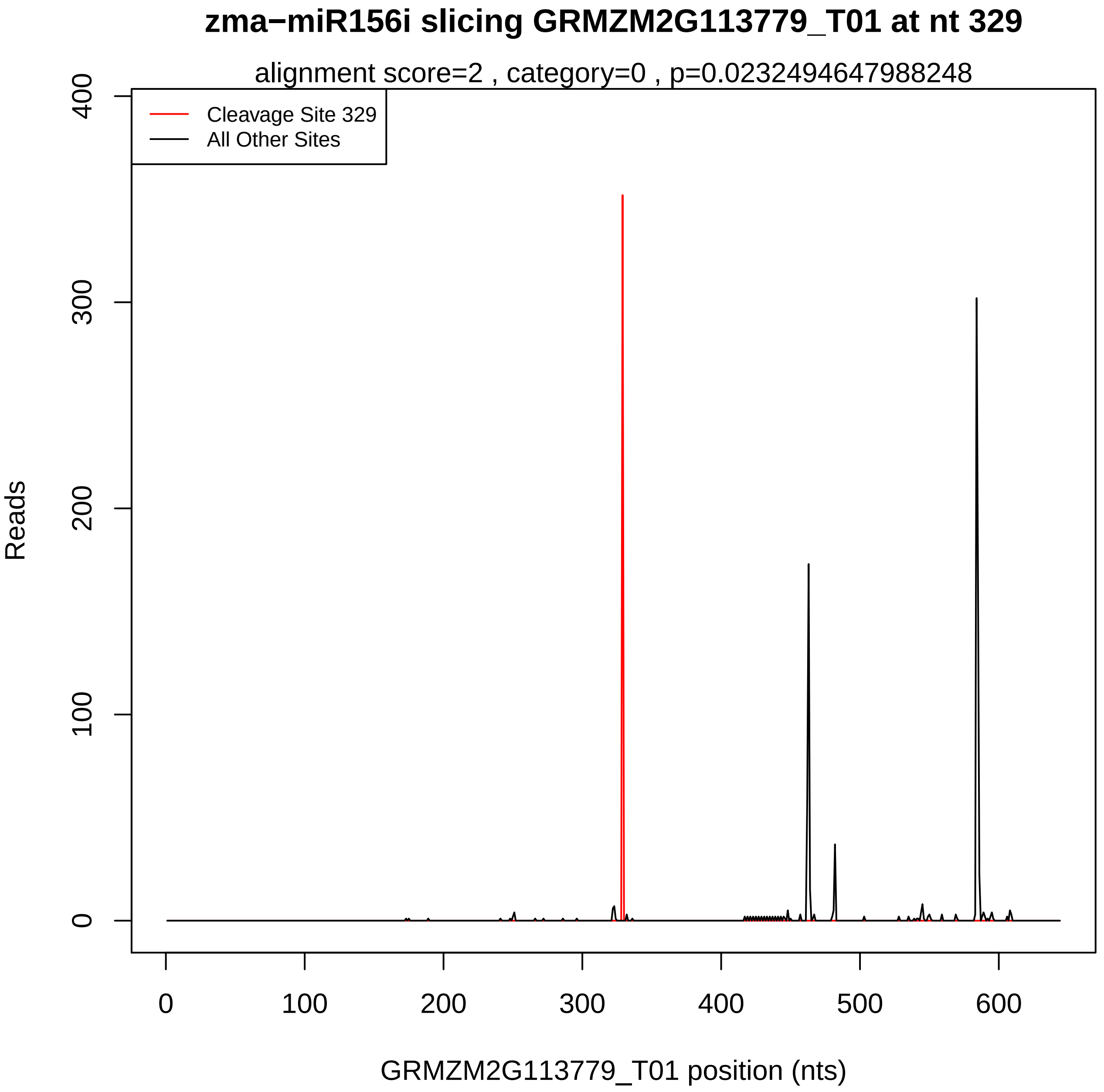

zma−miR156i slicing GRMZM2G113779_T01 at nt 329
alignment score=2 , category=0 , p=0.0232494647988248
Cleavage Site 329
All Other Sites
Reads
0
100
200
300
400
0
100
200
300
400
500
600
GRMZM2G113779_T01 position (nts)

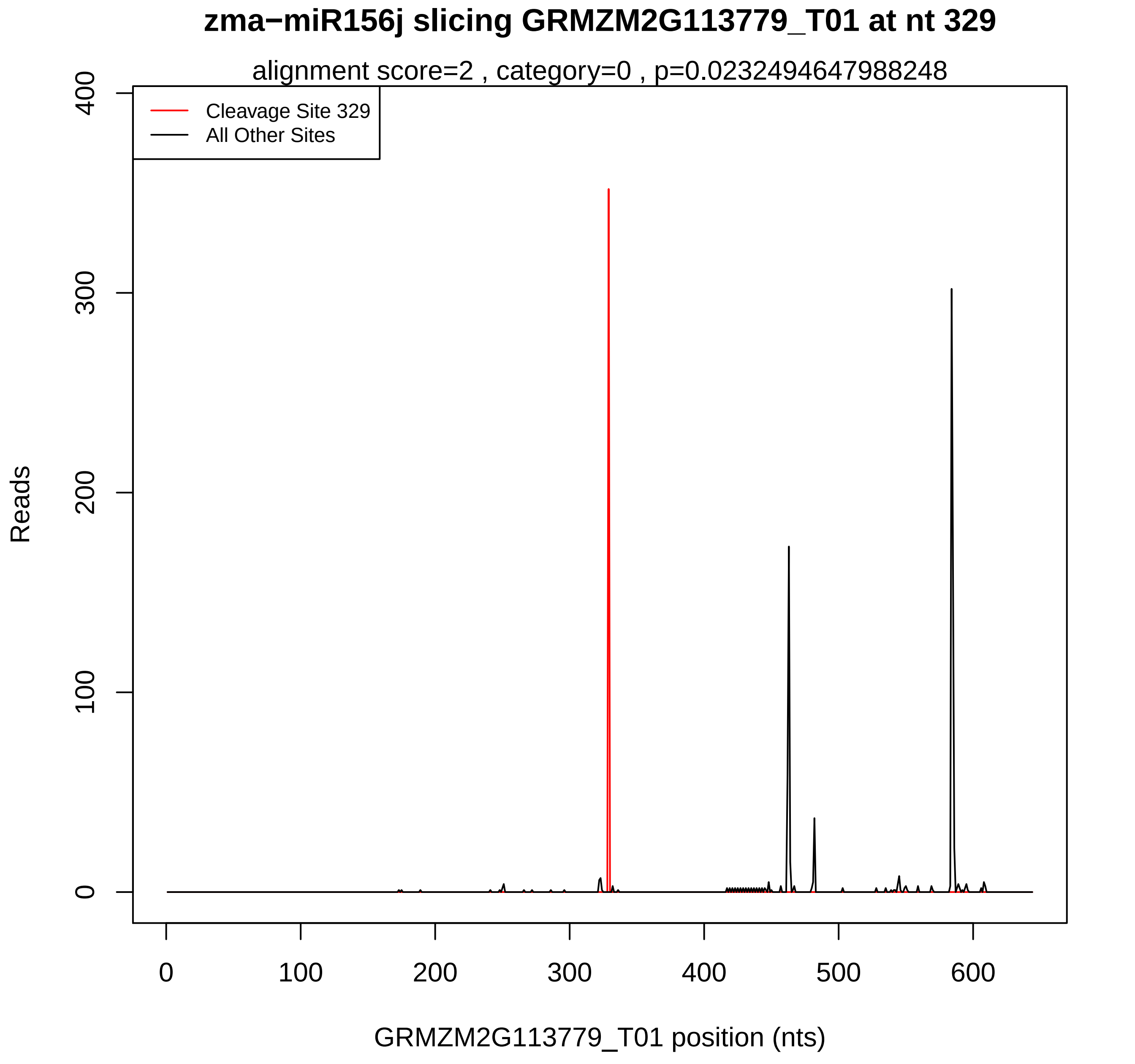
zma−miR156j slicing GRMZM2G113779_T01 at nt 329
alignment score=2 , category=0 , p=0.0232494647988248
Cleavage Site 329
All Other Sites
Reads
0
100
200
300
400
0
100
200
300
400
500
600
GRMZM2G113779_T01 position (nts)

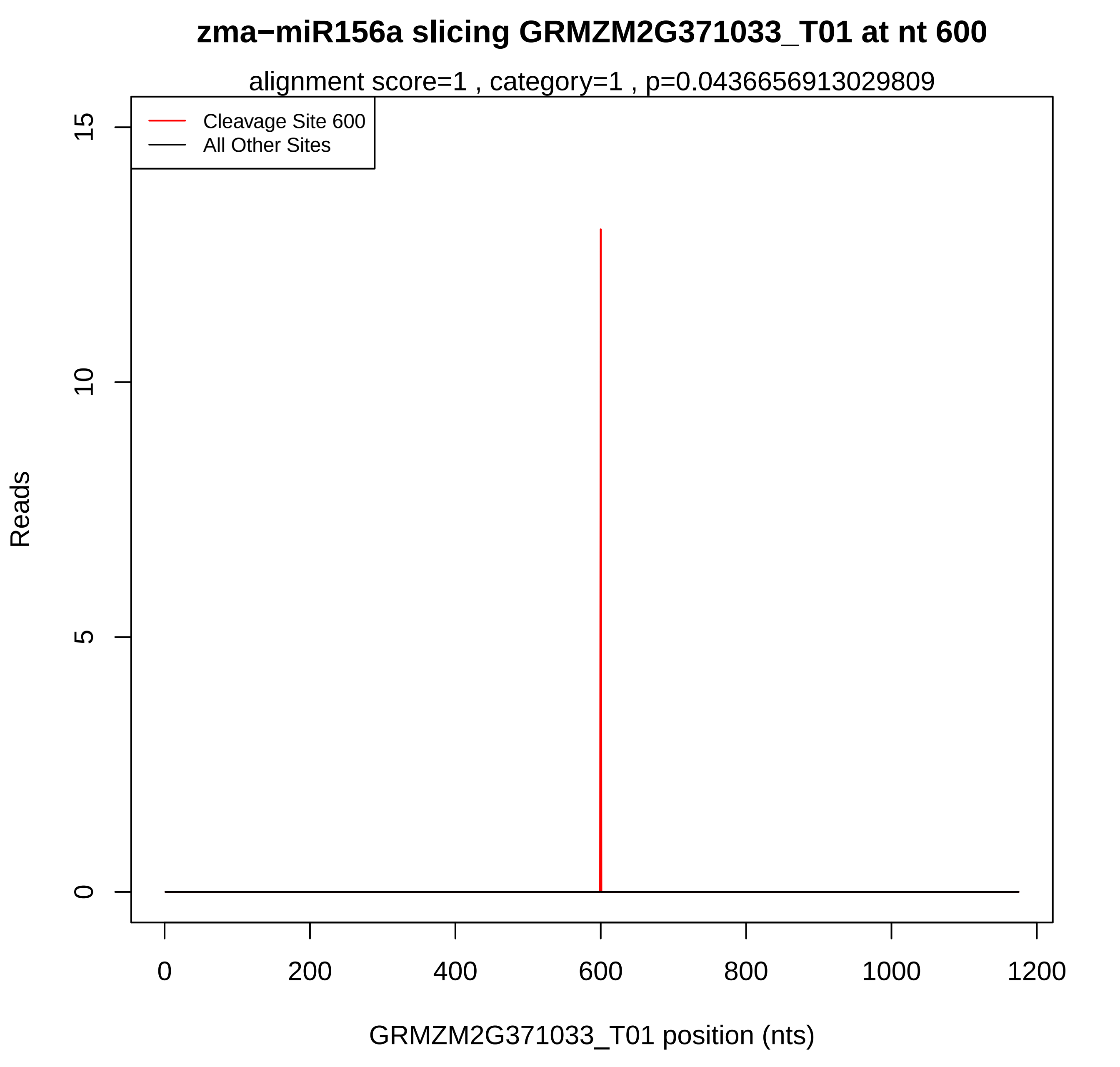
zma−miR156a slicing GRMZM2G371033_T01 at nt 600
alignment score=1 , category=1 , p=0.0436656913029809
Cleavage Site 600
All Other Sites
Reads
0
5
10
15
0
200
400
600
800
1000
1200
GRMZM2G371033_T01 position (nts)

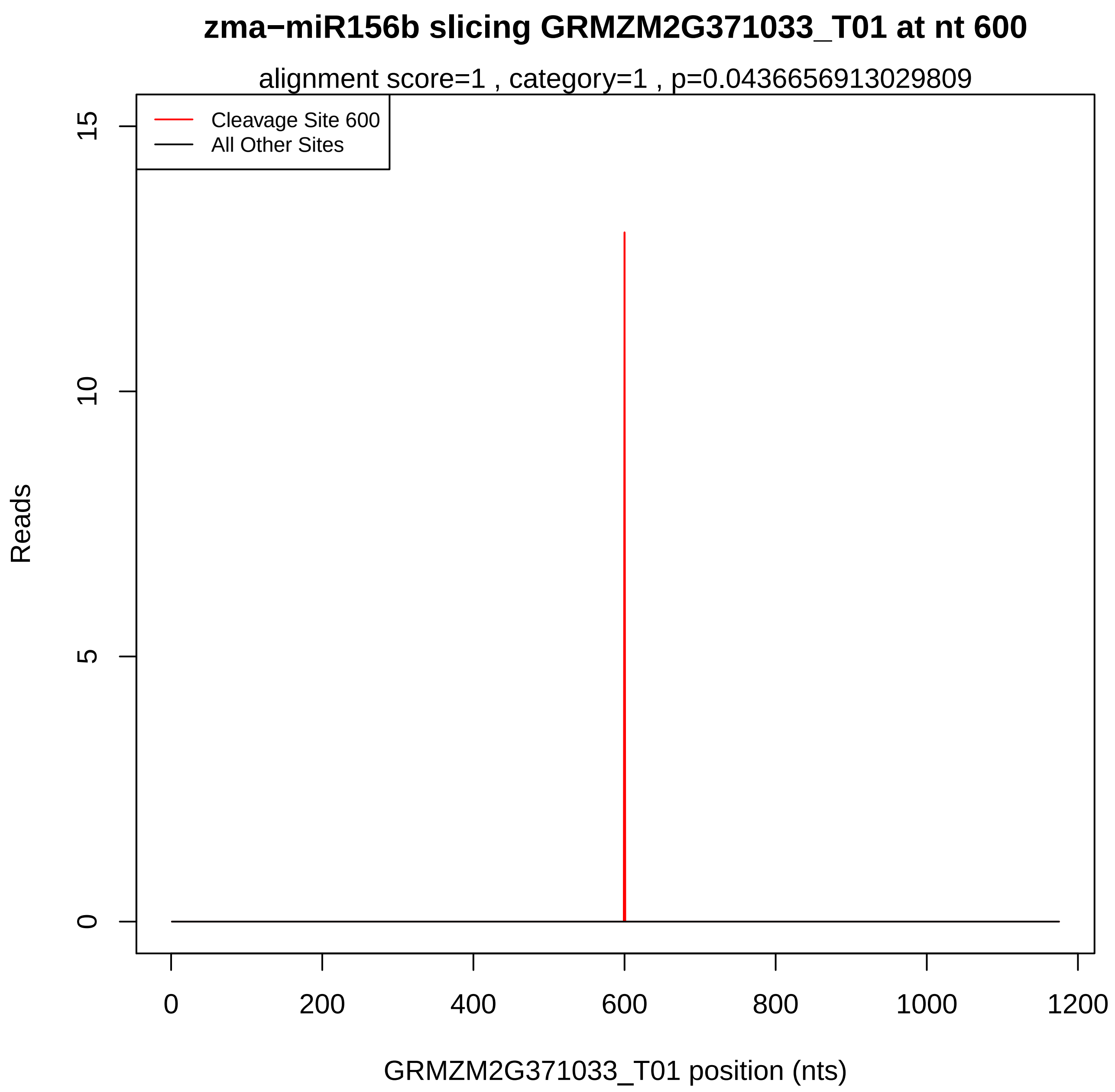
zma−miR156b slicing GRMZM2G371033_T01 at nt 600
alignment score=1 , category=1 , p=0.0436656913029809
Cleavage Site 600
All Other Sites
Reads
0
5
10
15
0
200
400
600
800
1000
1200
GRMZM2G371033_T01 position (nts)

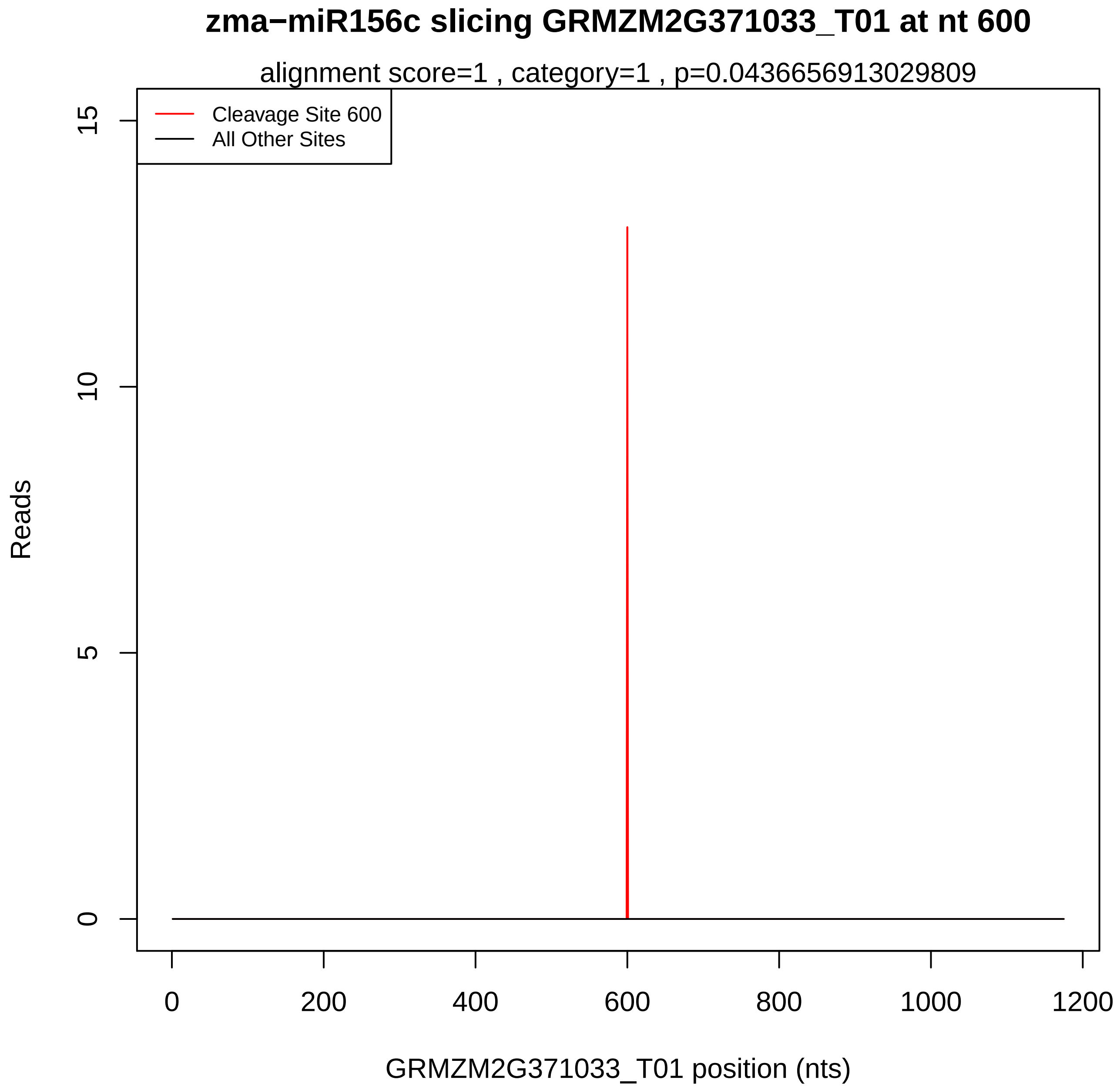

zma−miR156c slicing GRMZM2G371033_T01 at nt 600
alignment score=1 , category=1 , p=0.0436656913029809
Cleavage Site 600
All Other Sites
Reads
0
5
10
15
0
200
400
600
800
1000
1200
GRMZM2G371033_T01 position (nts)

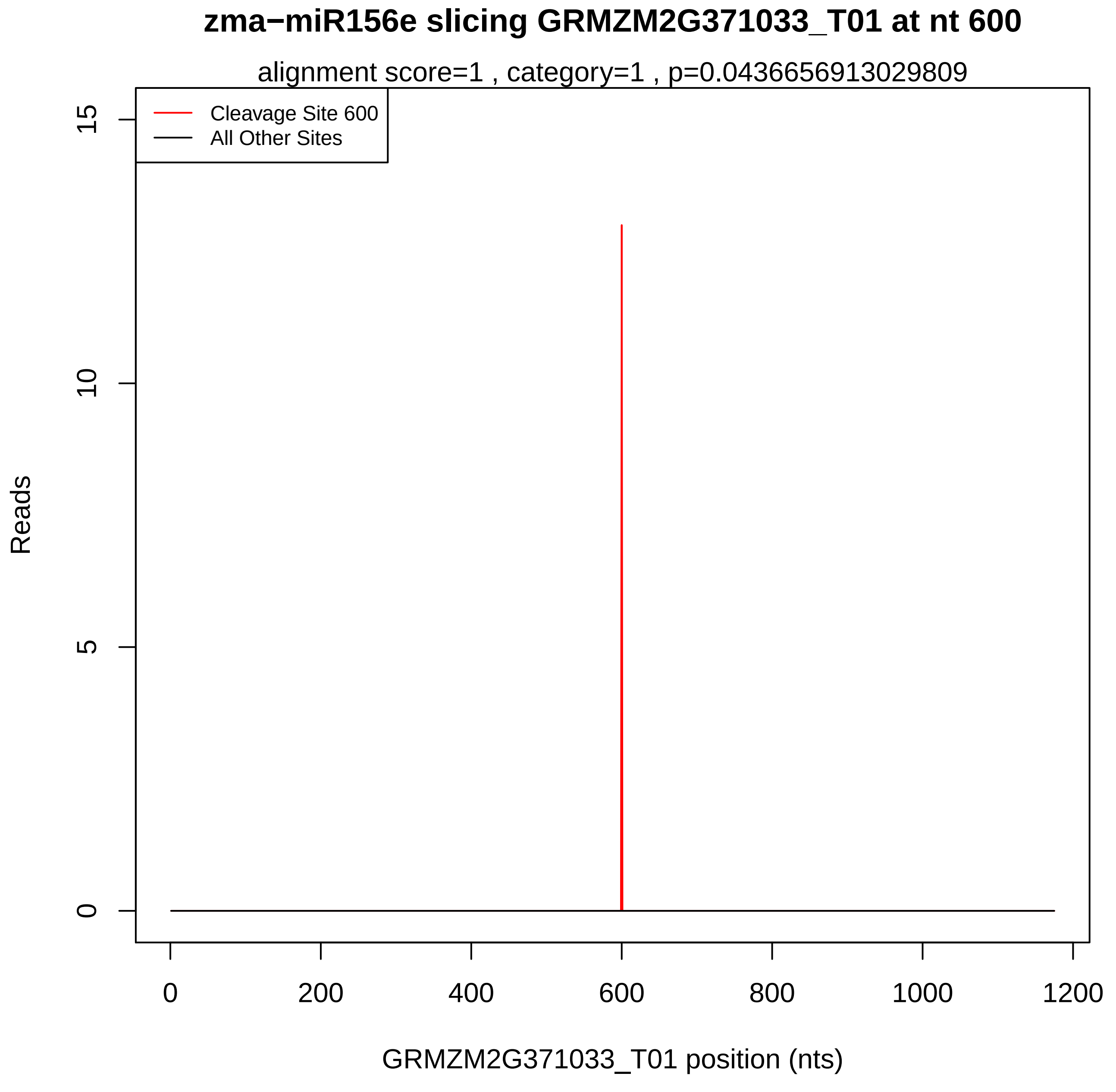

zma−miR156e slicing GRMZM2G371033_T01 at nt 600
alignment score=1 , category=1 , p=0.0436656913029809
Cleavage Site 600
All Other Sites
Reads
0
5
10
15
0
200
400
600
800
1000
1200
GRMZM2G371033_T01 position (nts)

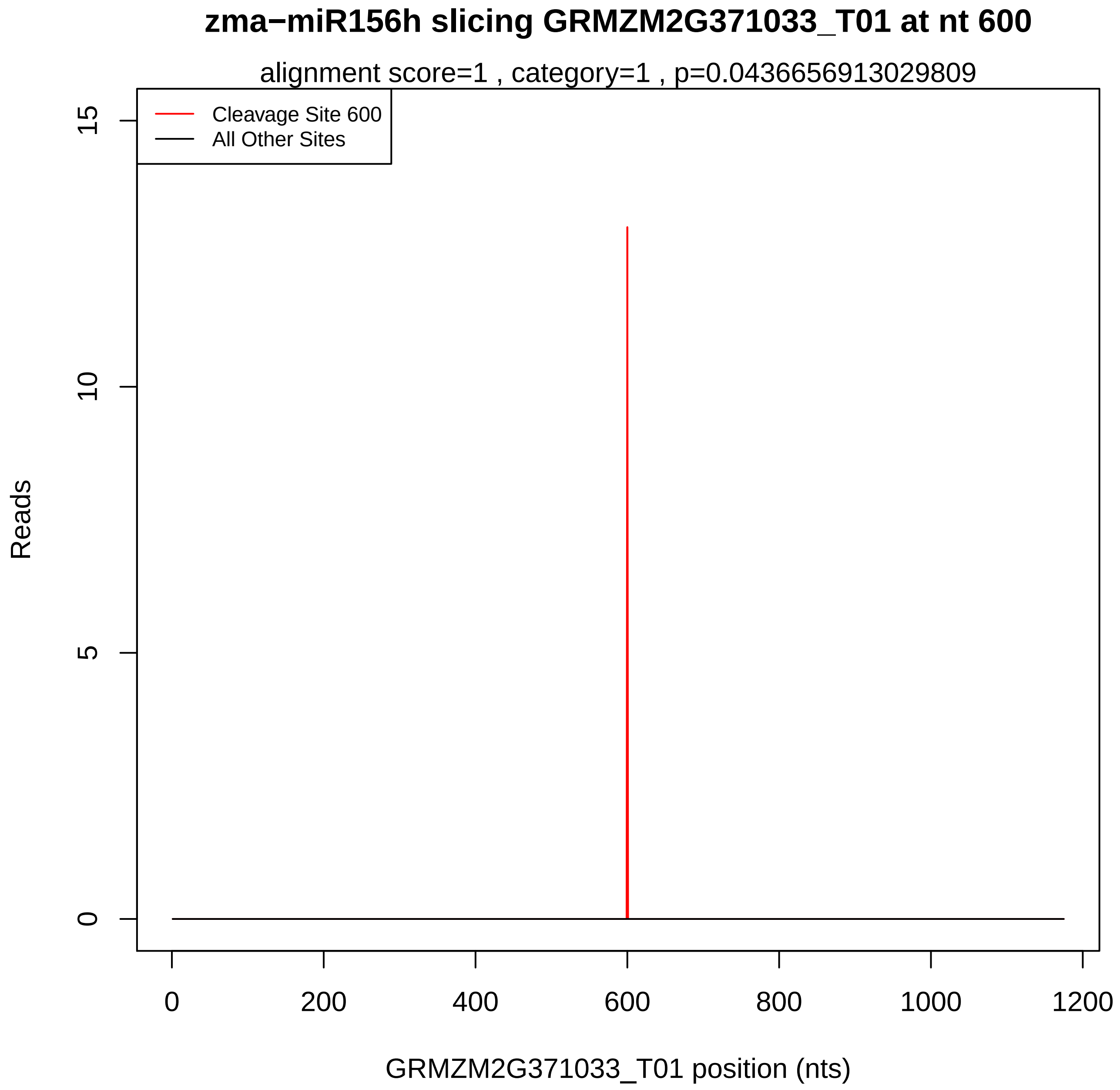
zma−miR156h slicing GRMZM2G371033_T01 at nt 600
alignment score=1 , category=1 , p=0.0436656913029809
Cleavage Site 600
All Other Sites
Reads
0
5
10
15
0
200
400
600
800
1000
1200
GRMZM2G371033_T01 position (nts)

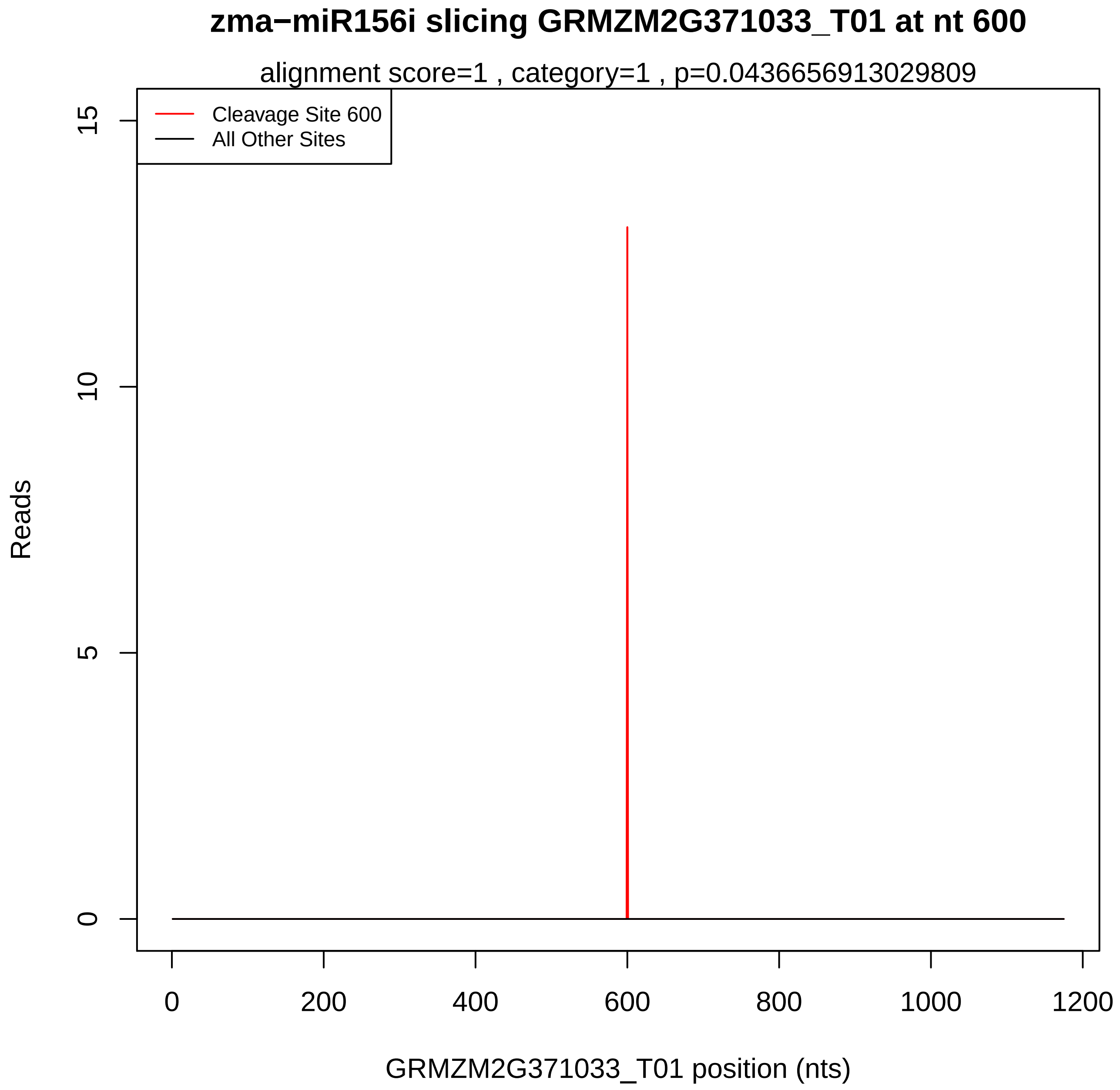

zma−miR156i slicing GRMZM2G371033_T01 at nt 600
alignment score=1 , category=1 , p=0.0436656913029809
Cleavage Site 600
All Other Sites
Reads
0
5
10
15
0
200
400
600
800
1000
1200
GRMZM2G371033_T01 position (nts)

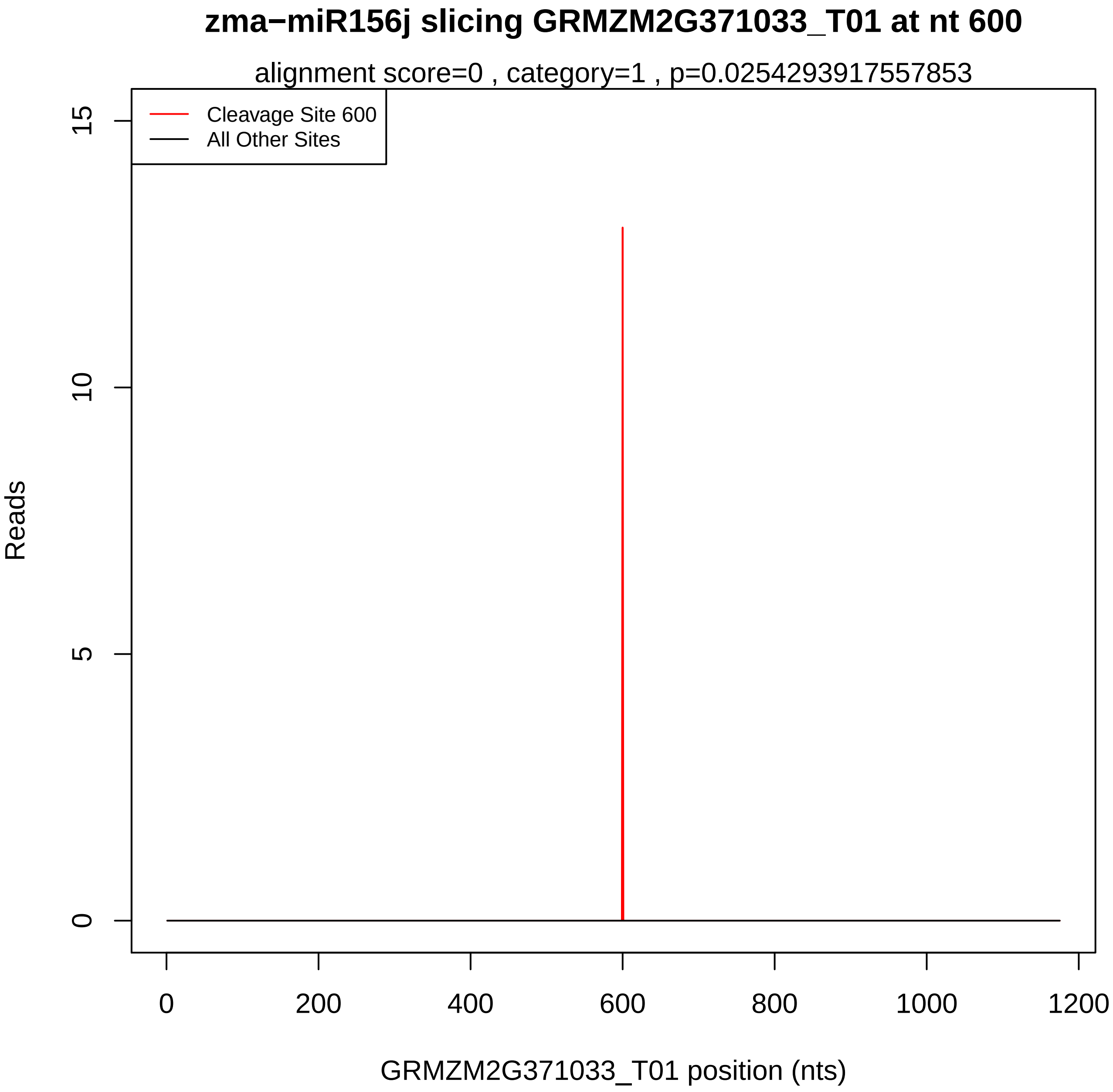
zma−miR156j slicing GRMZM2G371033_T01 at nt 600
alignment score=0 , category=1 , p=0.0254293917557853
Cleavage Site 600
All Other Sites
Reads
0
5
10
15
0
200
400
600
800
1000
1200
GRMZM2G371033_T01 position (nts)

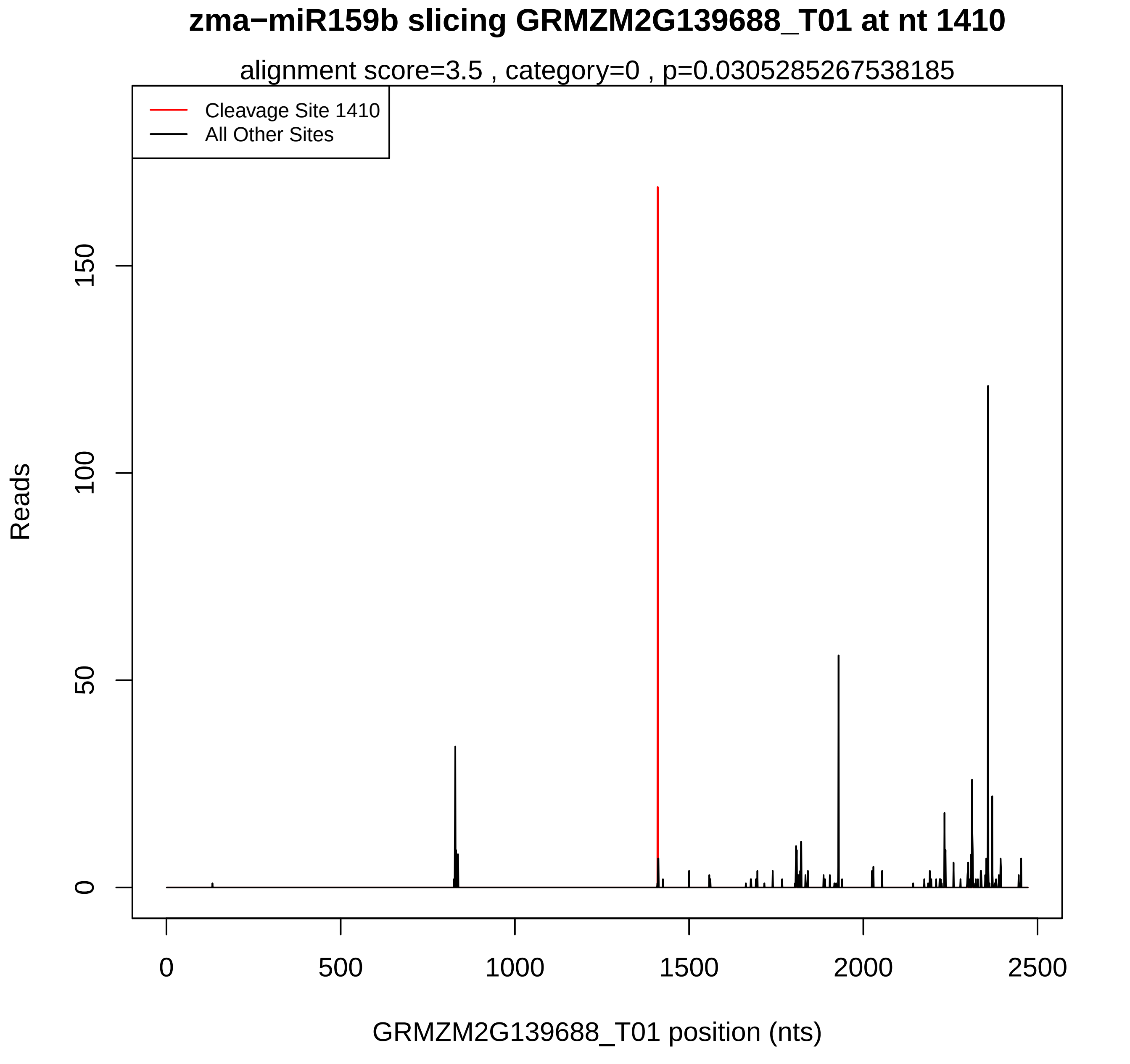
zma−miR159b slicing GRMZM2G139688_T01 at nt 1410
alignment score=3.5 , category=0 , p=0.0305285267538185
Cleavage Site 1410
All Other Sites
Reads
0
50
100
150
0
500
1000
1500
2000
2500
GRMZM2G139688_T01 position (nts)

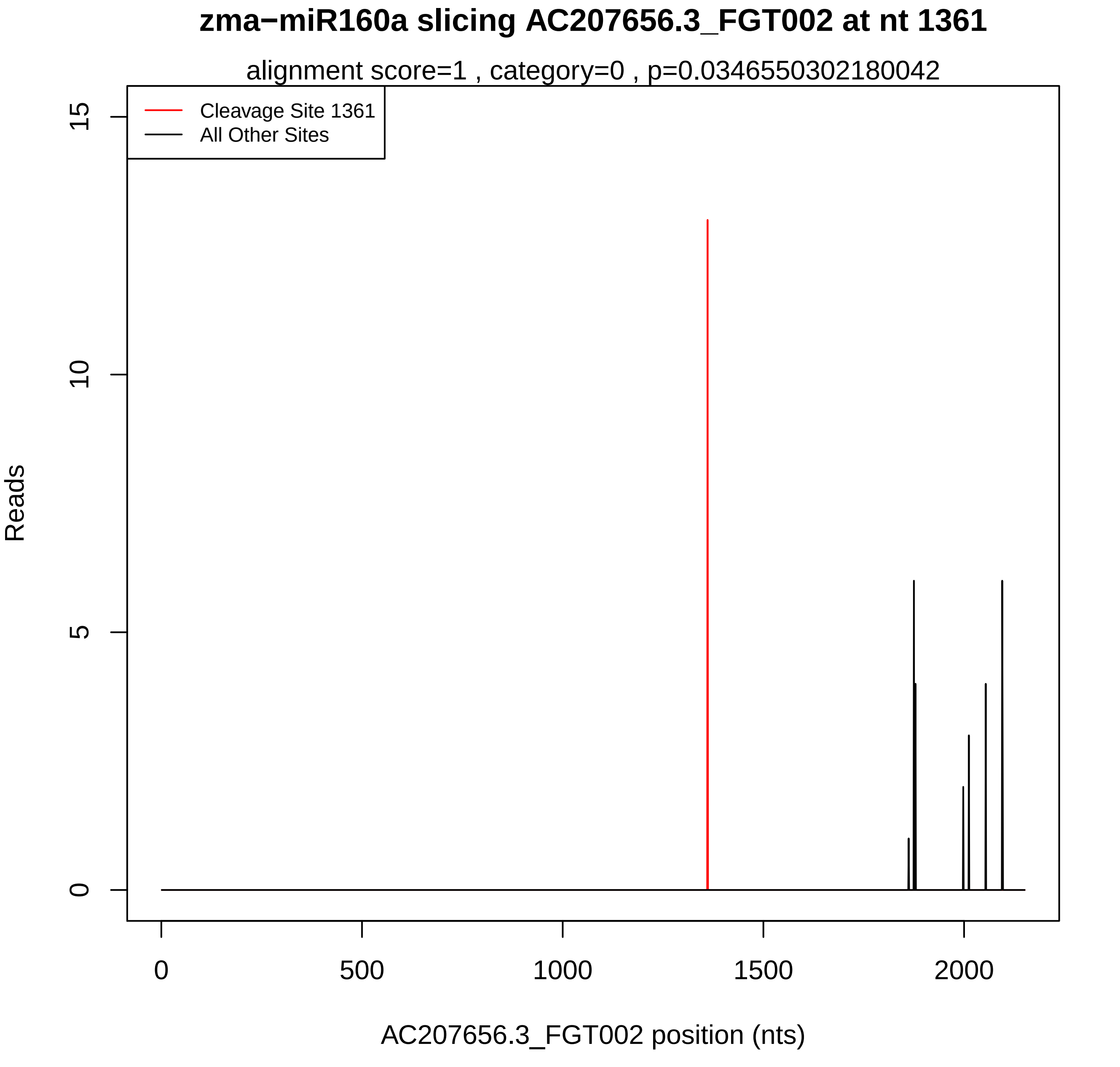
zma−miR160a slicing AC207656.3_FGT002 at nt 1361
alignment score=1 , category=0 , p=0.0346550302180042
Cleavage Site 1361
All Other Sites
Reads
0
5
10
15
0
500
1000
1500
2000
AC207656.3_FGT002 position (nts)

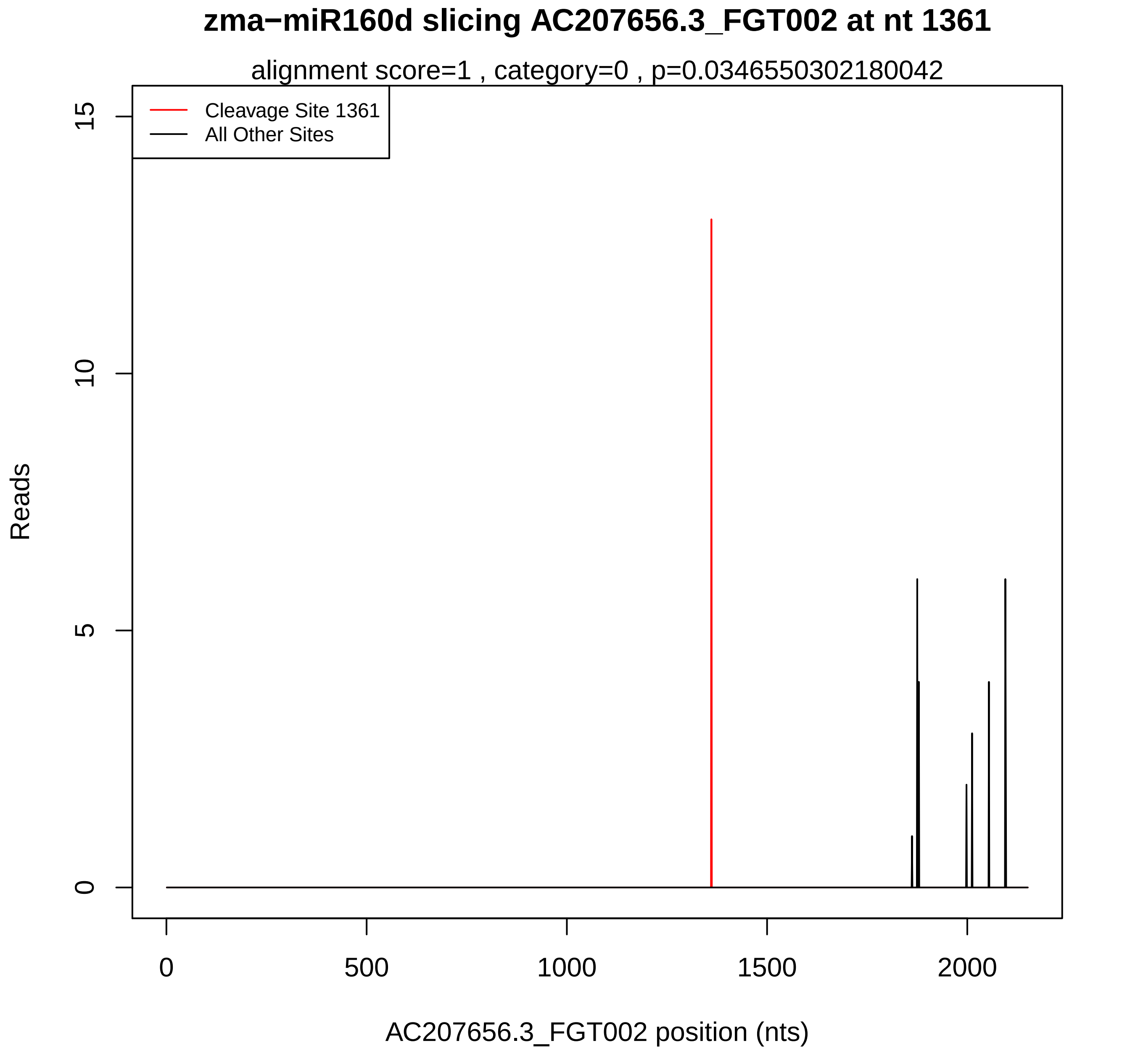

zma−miR160d slicing AC207656.3_FGT002 at nt 1361
alignment score=1 , category=0 , p=0.0346550302180042
Cleavage Site 1361
All Other Sites
Reads
0
5
10
15
0
500
1000
1500
2000
AC207656.3_FGT002 position (nts)

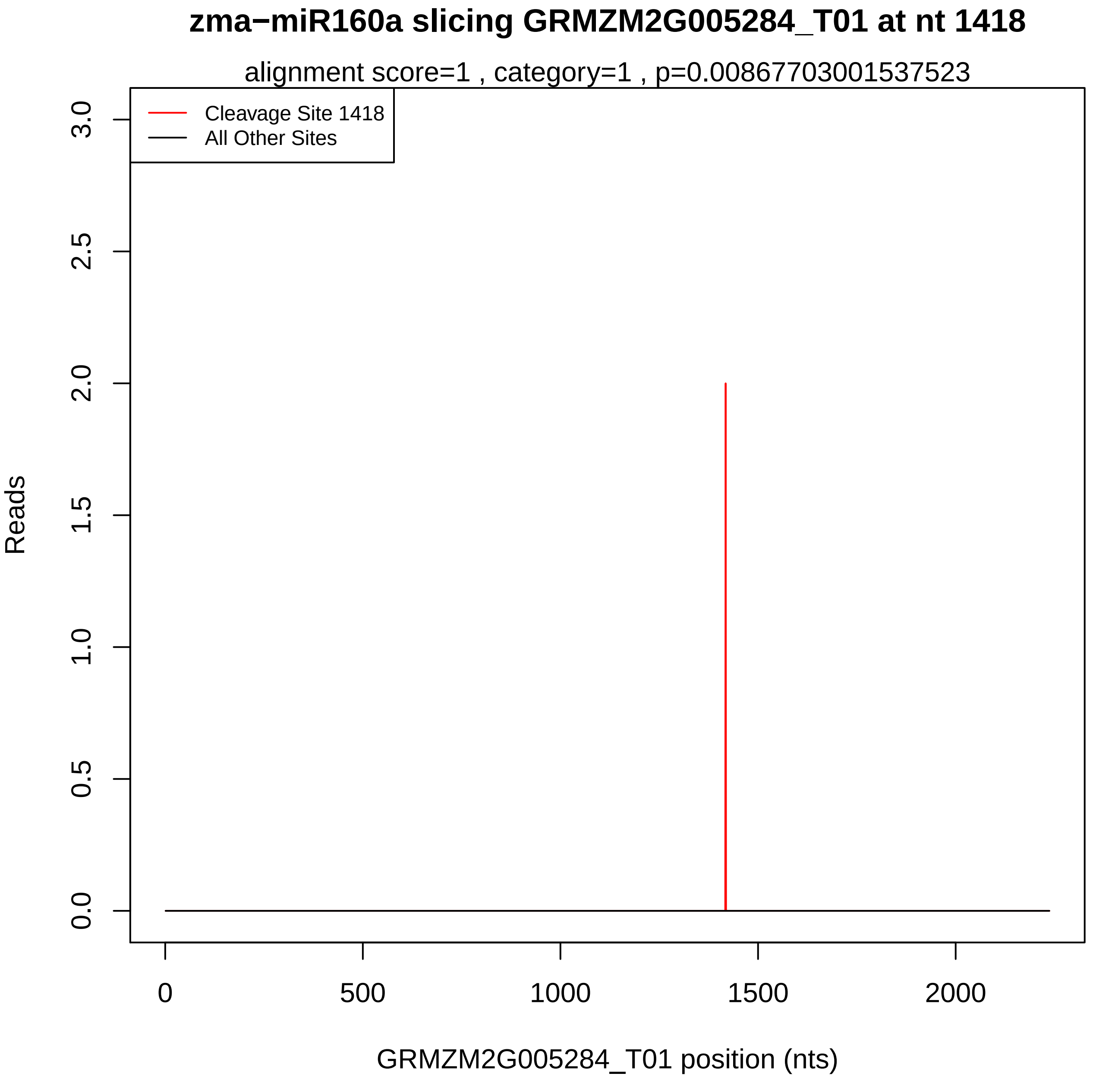
zma−miR160a slicing GRMZM2G005284_T01 at nt 1418
alignment score=1 , category=1 , p=0.00867703001537523
Cleavage Site 1418
All Other Sites
Reads
0.0
0.5
1.0
1.5
2.0
2.5
3.0
0
500
1000
1500
2000
GRMZM2G005284_T01 position (nts)

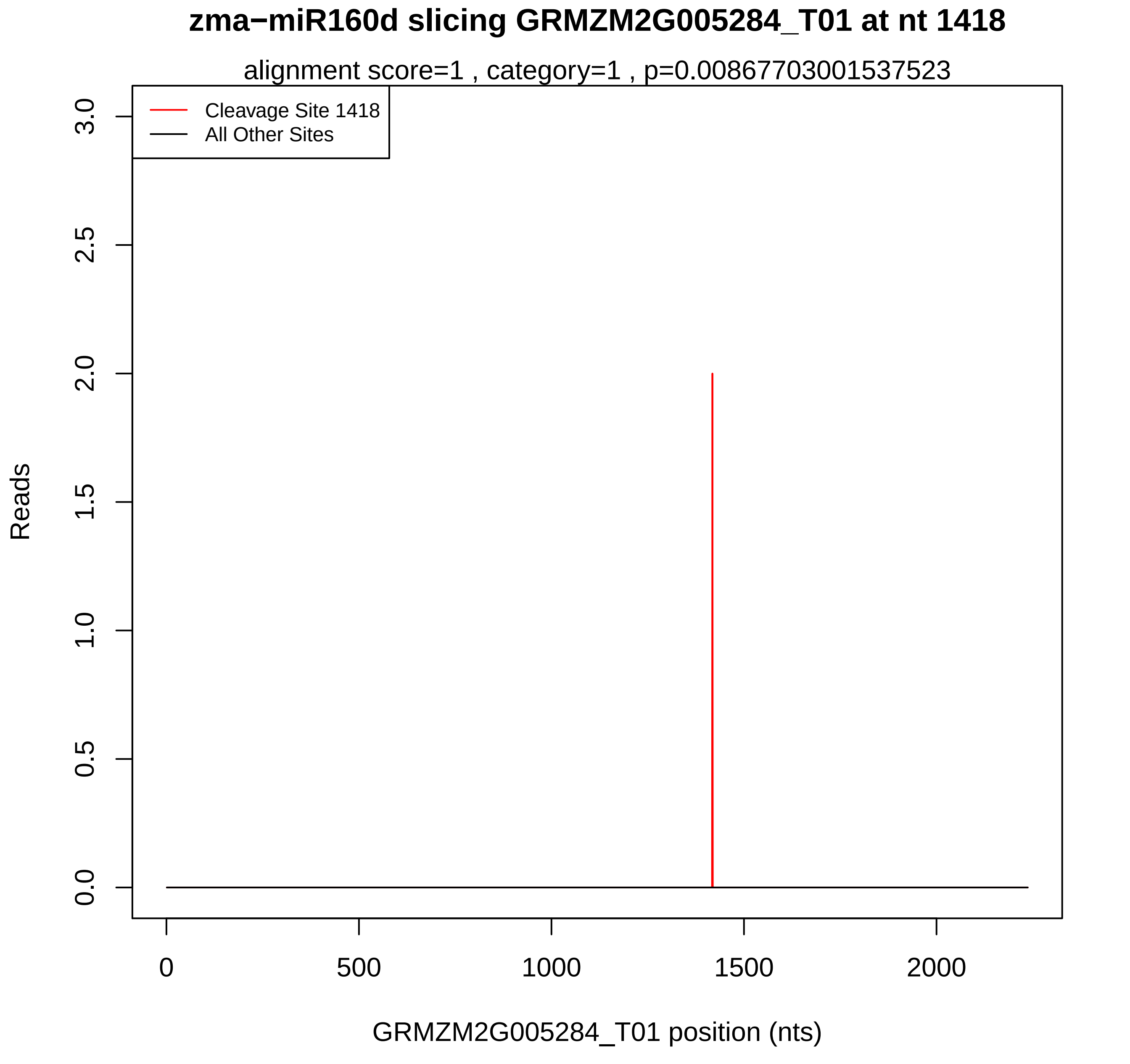

zma−miR160d slicing GRMZM2G005284_T01 at nt 1418
alignment score=1 , category=1 , p=0.00867703001537523
Cleavage Site 1418
All Other Sites
Reads
0.0
0.5
1.0
1.5
2.0
2.5
3.0
0
500
1000
1500
2000
GRMZM2G005284_T01 position (nts)

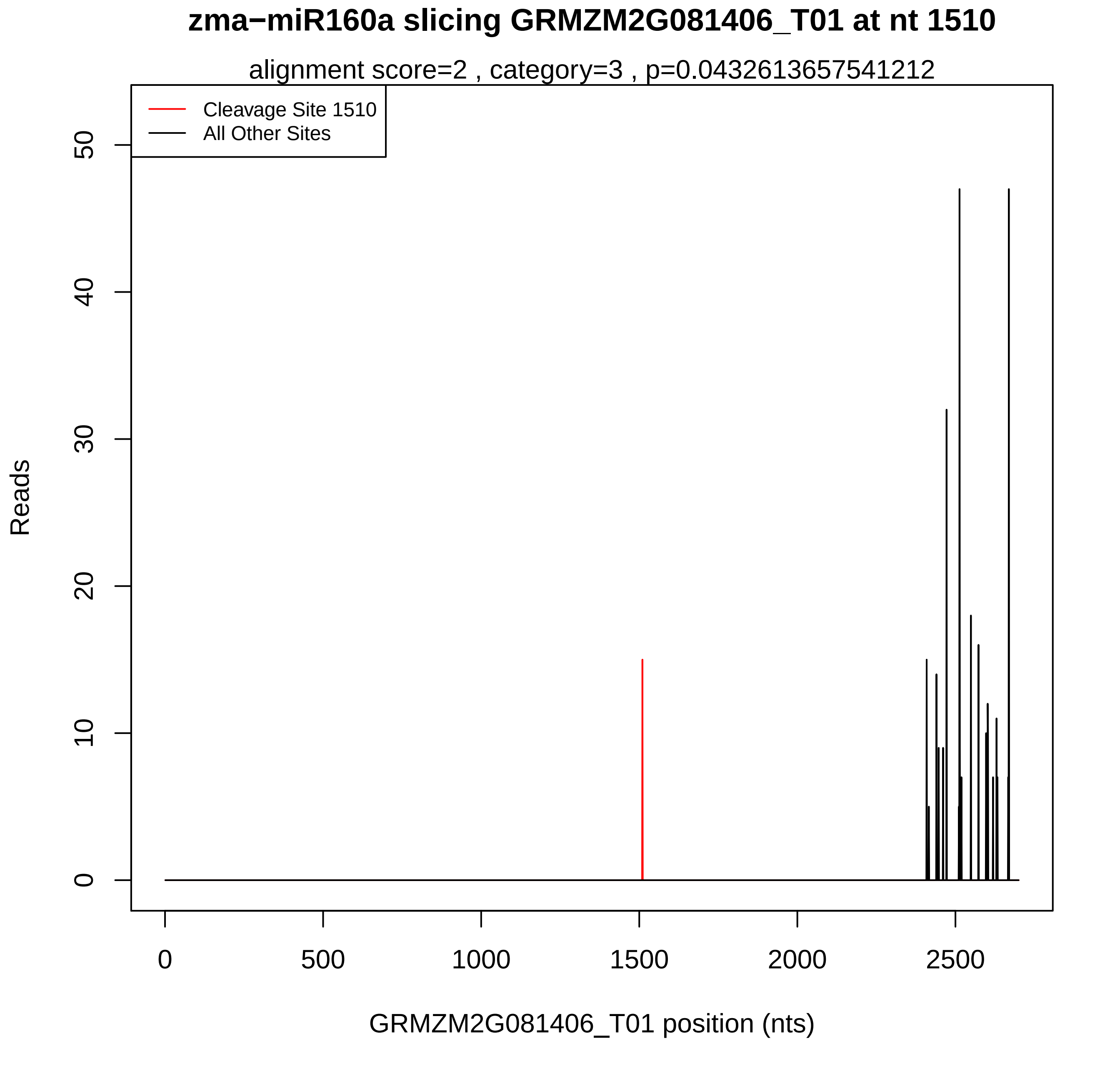

zma−miR160a slicing GRMZM2G081406_T01 at nt 1510
alignment score=2 , category=3 , p=0.0432613657541212
Cleavage Site 1510
All Other Sites
Reads
0
10
20
30
40
50
0
500
1000
1500
2000
2500
GRMZM2G081406_T01 position (nts)

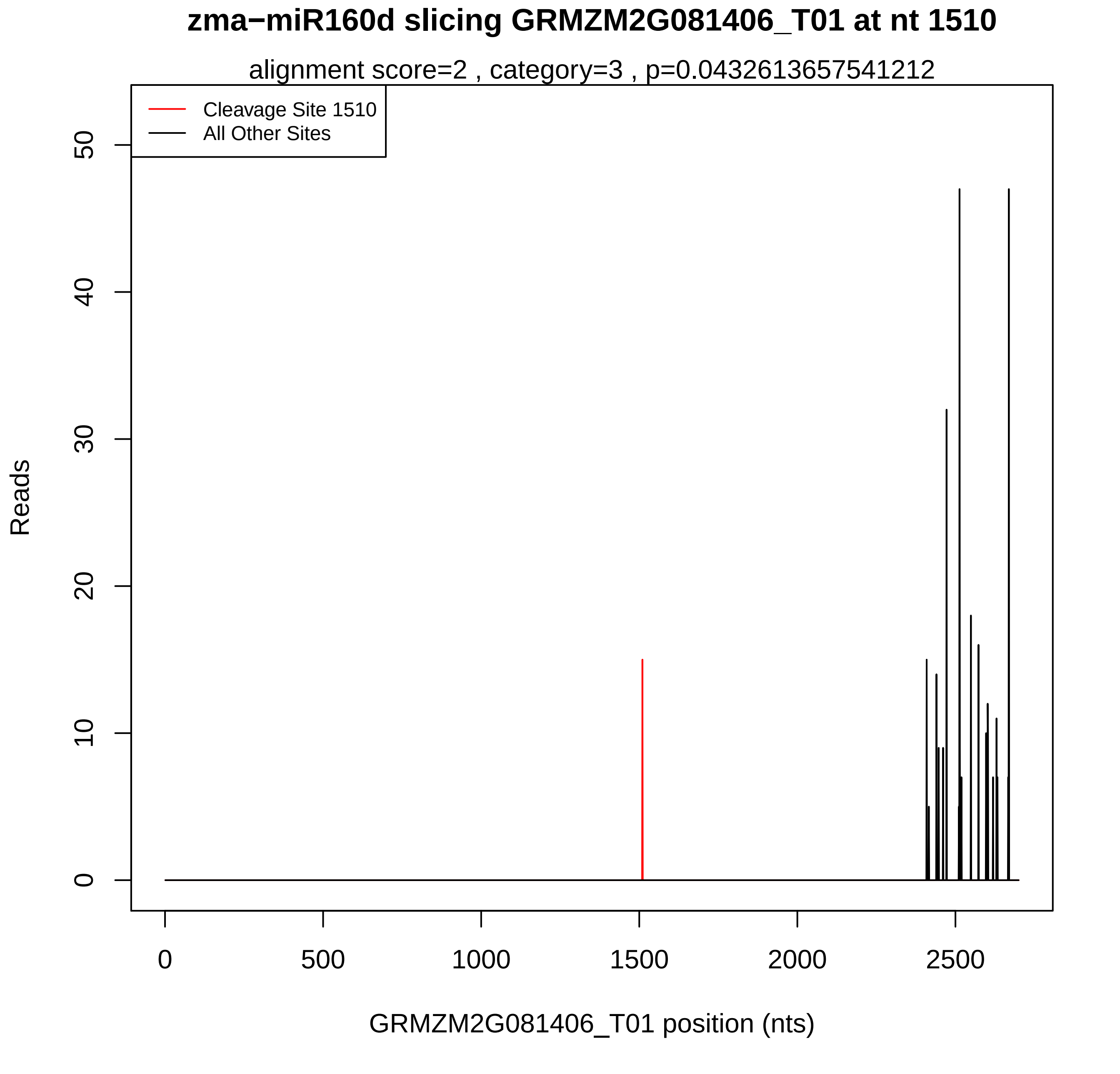
zma−miR160d slicing GRMZM2G081406_T01 at nt 1510
alignment score=2 , category=3 , p=0.0432613657541212
Cleavage Site 1510
All Other Sites
Reads
0
10
20
30
40
50
0
500
1000
1500
2000
2500
GRMZM2G081406_T01 position (nts)

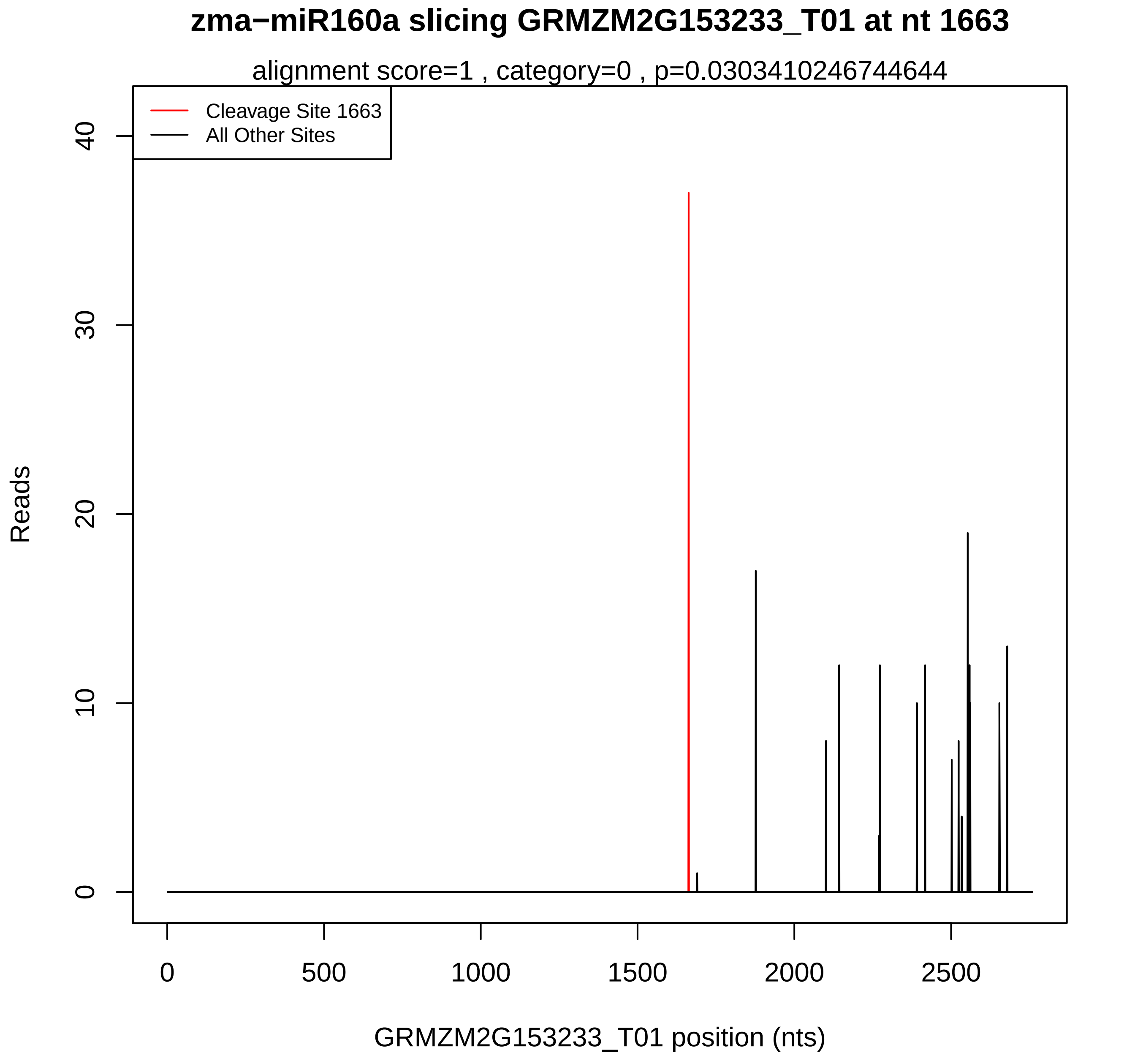

zma−miR160a slicing GRMZM2G153233_T01 at nt 1663
alignment score=1 , category=0 , p=0.0303410246744644
Cleavage Site 1663
All Other Sites
Reads
0
10
20
30
40
0
500
1000
1500
2000
2500
GRMZM2G153233_T01 position (nts)

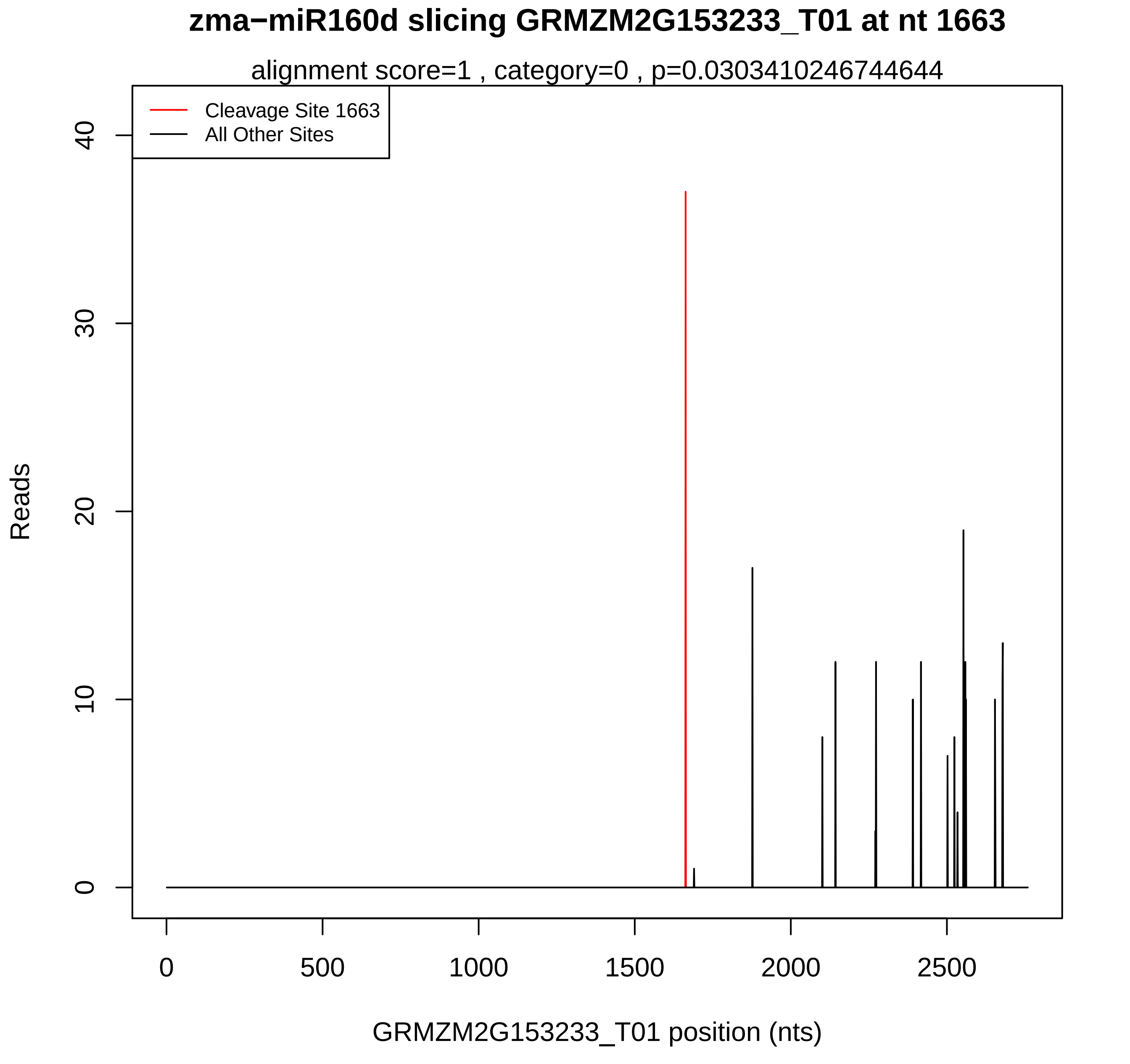
zma−miR160d slicing GRMZM2G153233_T01 at nt 1663
alignment score=1 , category=0 , p=0.0303410246744644
Cleavage Site 1663
All Other Sites
Reads
0
10
20
30
40
0
500
1000
1500
2000
2500
GRMZM2G153233_T01 position (nts)

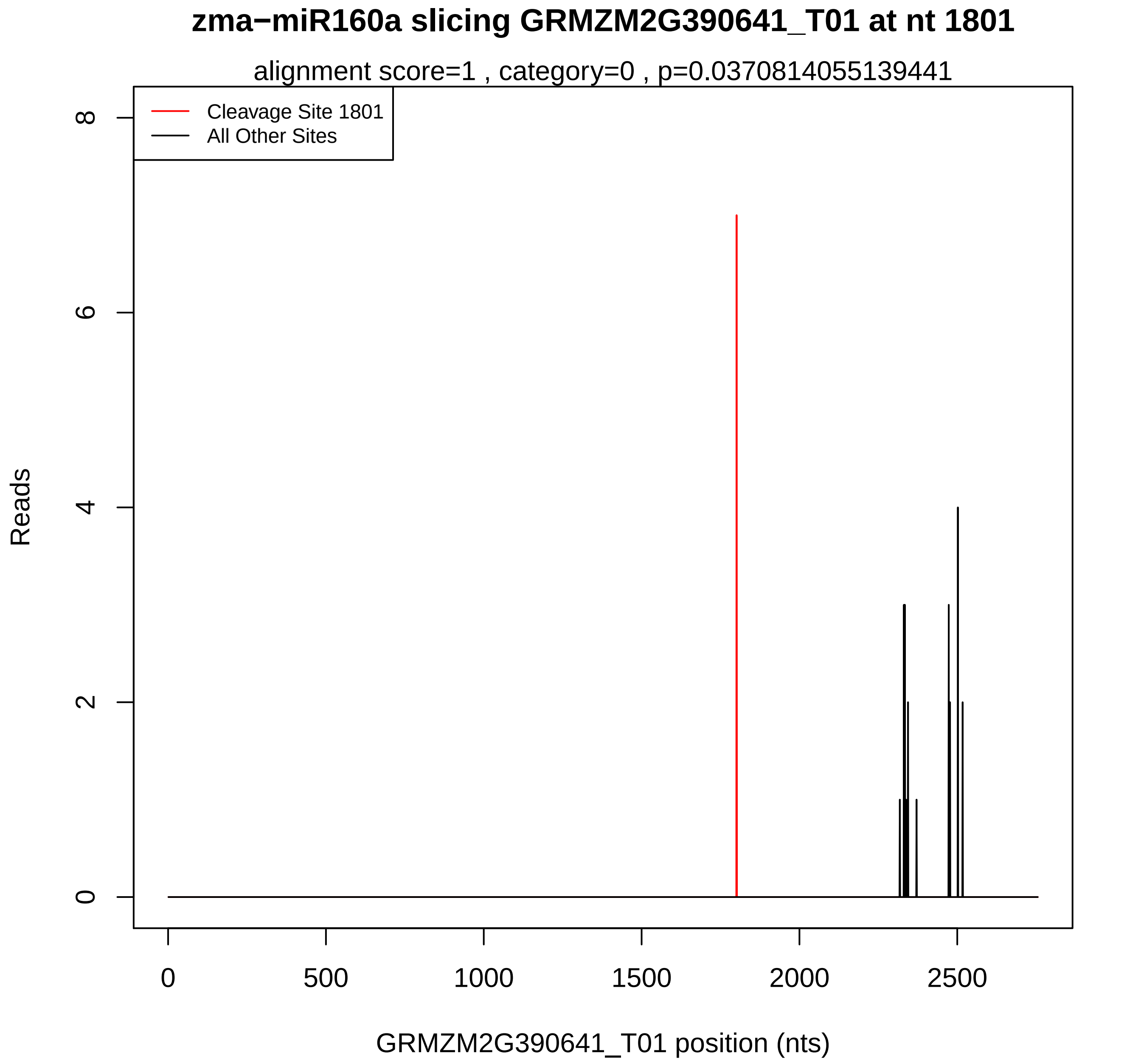

zma−miR160a slicing GRMZM2G390641_T01 at nt 1801
alignment score=1 , category=0 , p=0.0370814055139441
Cleavage Site 1801
All Other Sites
Reads
0
2
4
6
8
0
500
1000
1500
2000
2500
GRMZM2G390641_T01 position (nts)

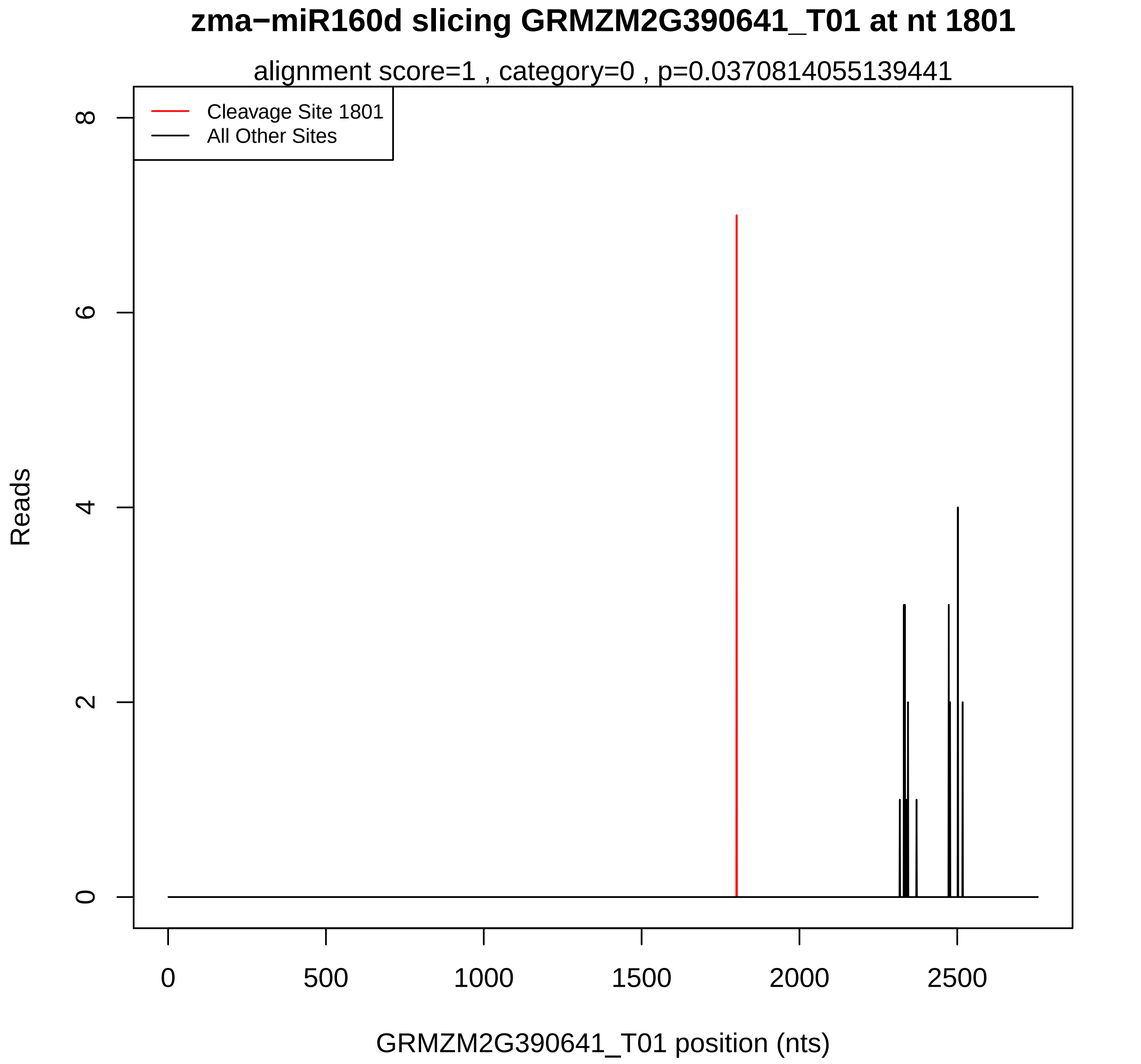
zma−miR160d slicing GRMZM2G390641_T01 at nt 1801
alignment score=1 , category=0 , p=0.0370814055139441
Cleavage Site 1801
All Other Sites
Reads
0
2
4
6
8
0
500
1000
1500
2000
2500
GRMZM2G390641_T01 position (nts)

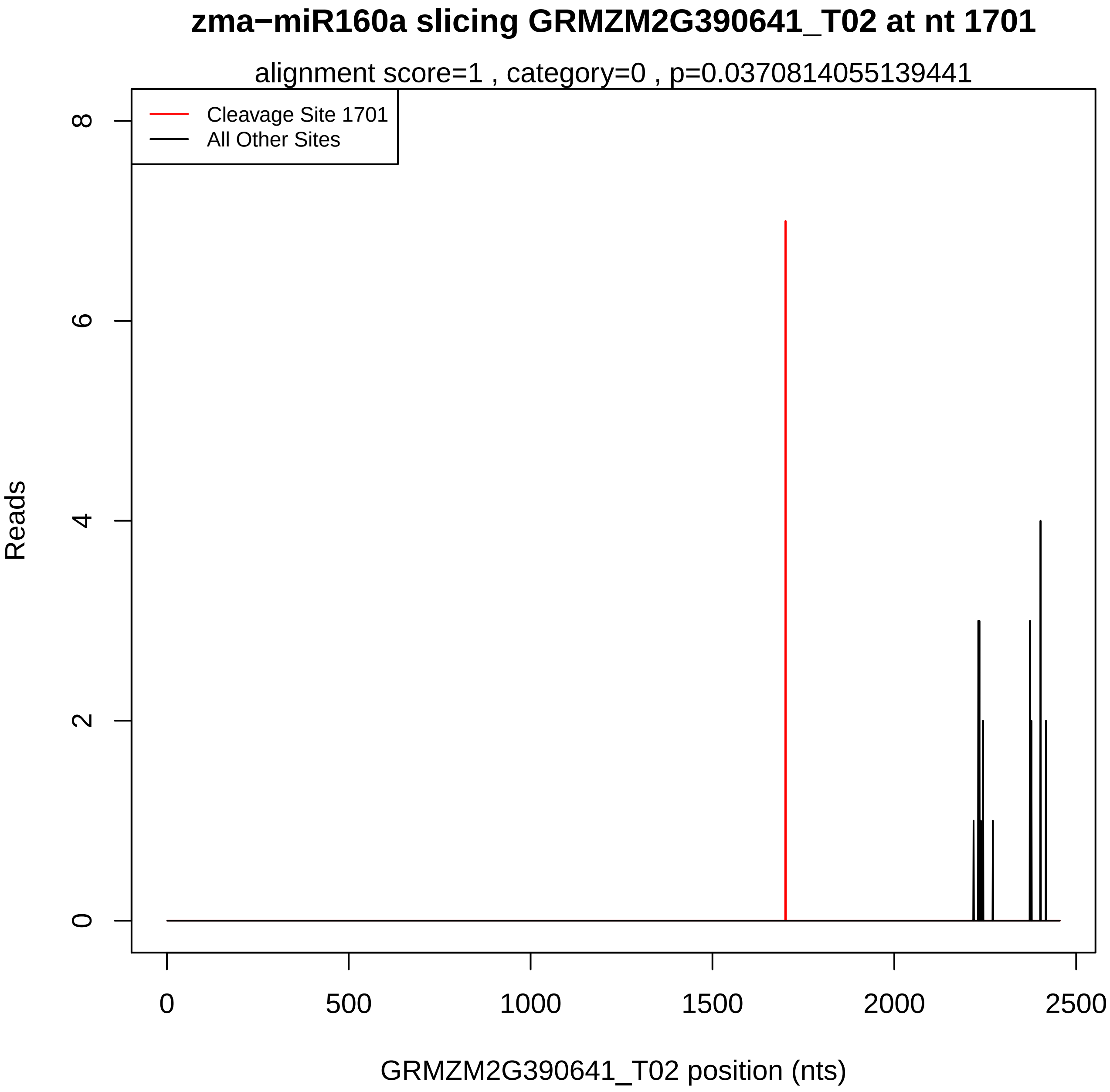
zma−miR160a slicing GRMZM2G390641_T02 at nt 1701
alignment score=1 , category=0 , p=0.0370814055139441
Cleavage Site 1701
All Other Sites
Reads
0
2
4
6
8
0
500
1000
1500
2000
2500
GRMZM2G390641_T02 position (nts)

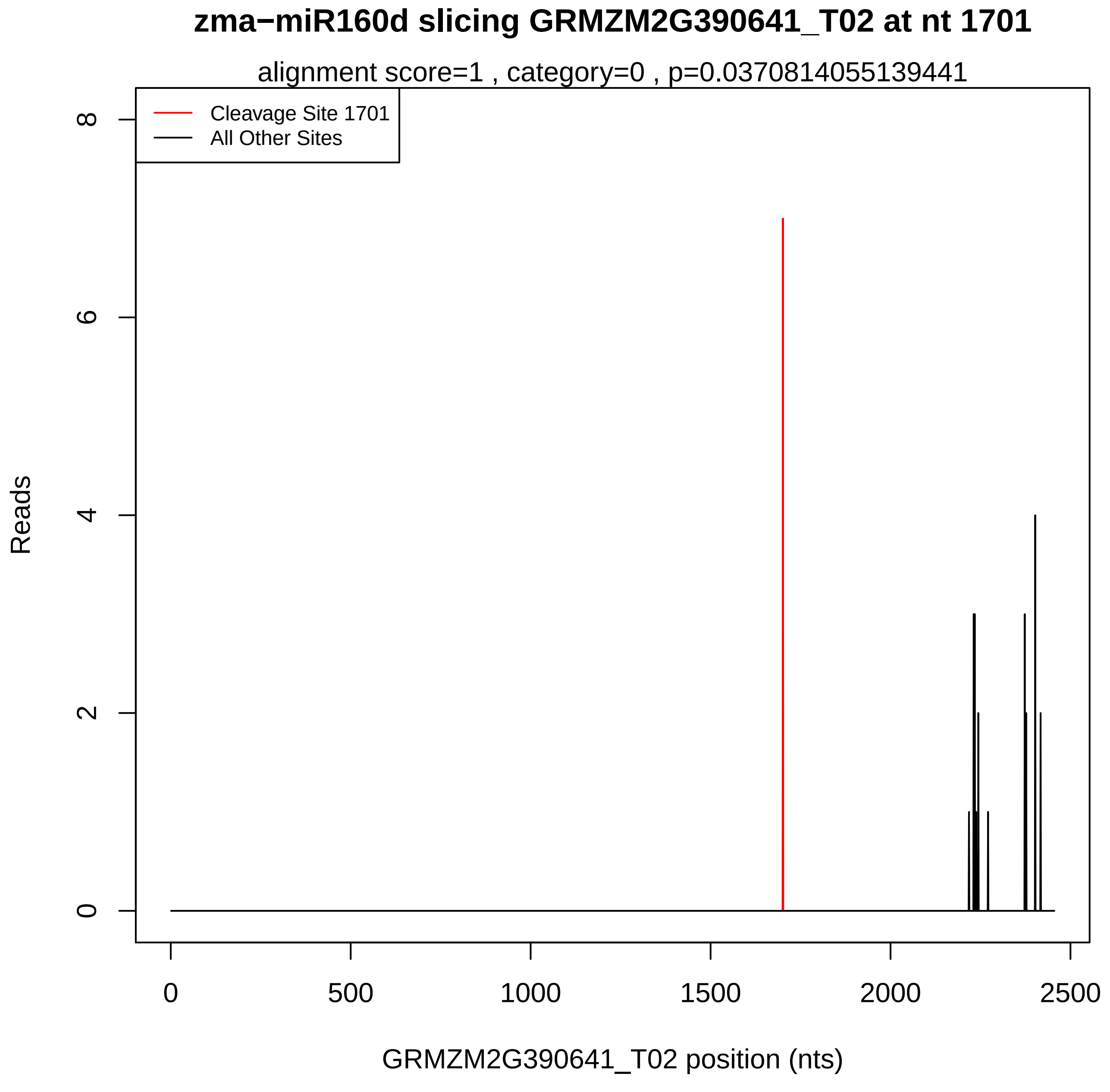
zma−miR160d slicing GRMZM2G390641_T02 at nt 1701
alignment score=1 , category=0 , p=0.0370814055139441
Cleavage Site 1701
All Other Sites
Reads
0
2
4
6
8
0
500
1000
1500
2000
2500
GRMZM2G390641_T02 position (nts)

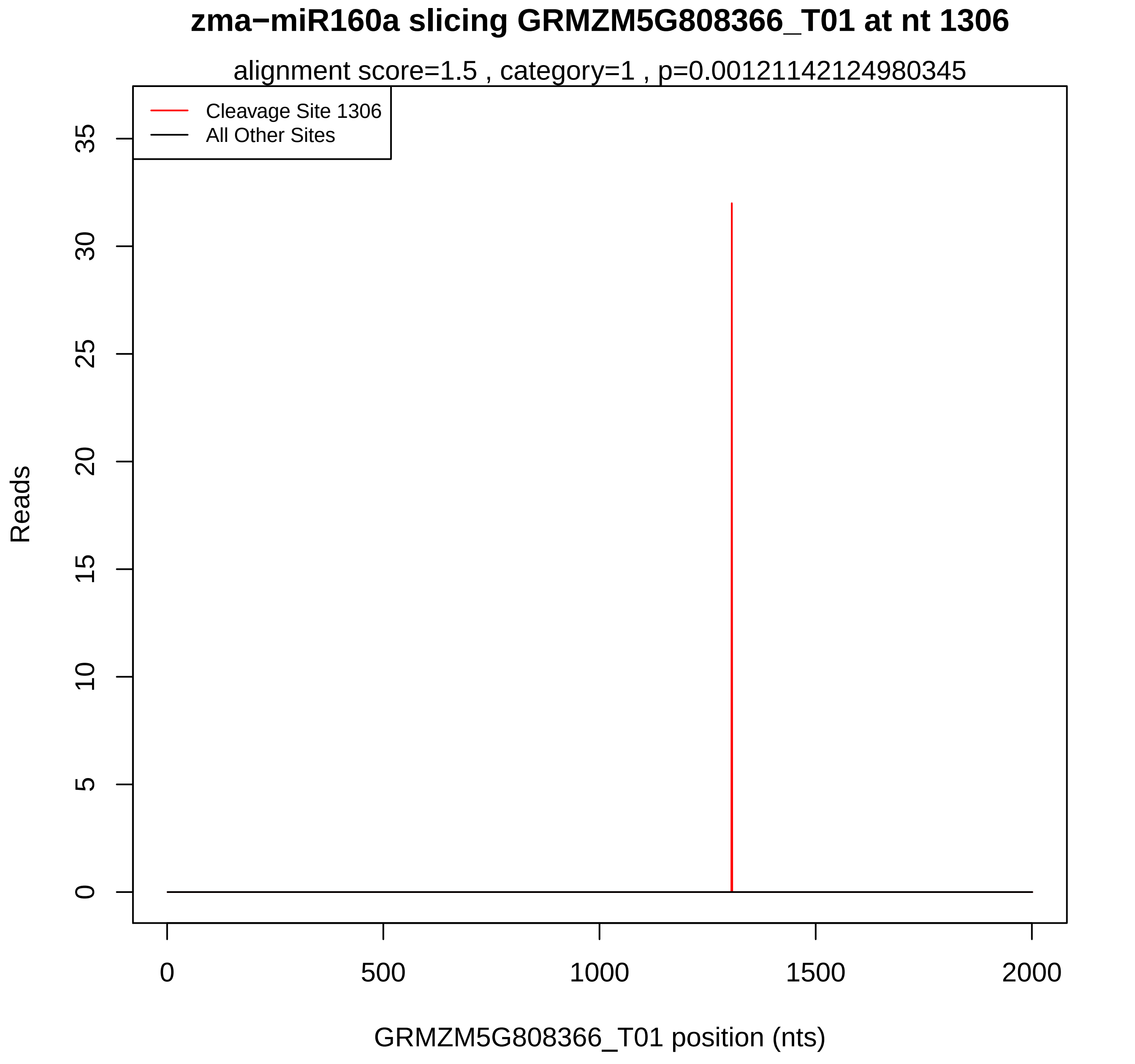
zma−miR160a slicing GRMZM5G808366_T01 at nt 1306
alignment score=1.5 , category=1 , p=0.00121142124980345
Cleavage Site 1306
All Other Sites
Reads
0
5
10
15
20
25
30
35
0
500
1000
1500
2000
GRMZM5G808366_T01 position (nts)

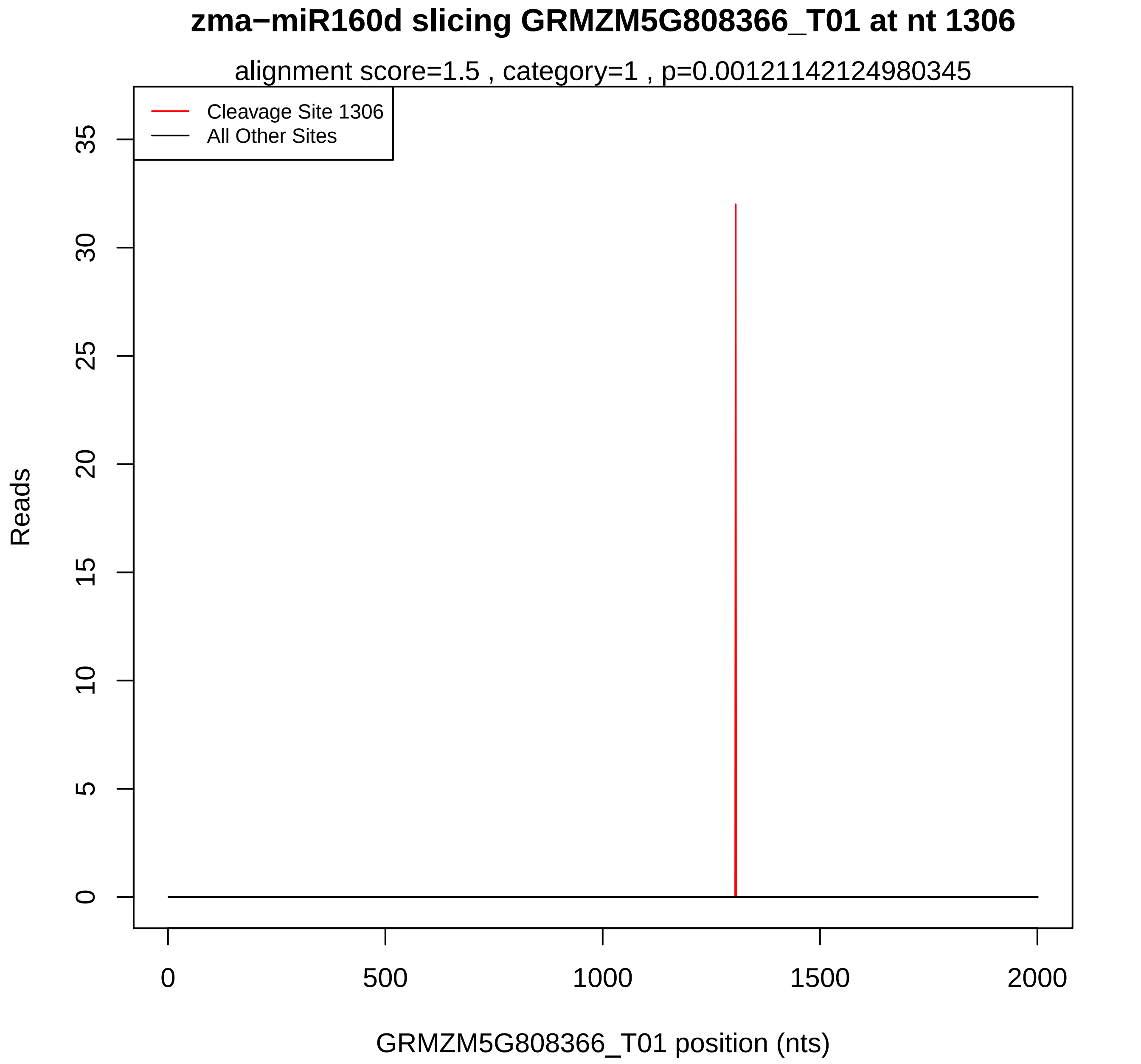
zma−miR160d slicing GRMZM5G808366_T01 at nt 1306
alignment score=1.5 , category=1 , p=0.00121142124980345
Cleavage Site 1306
All Other Sites
Reads
0
5
10
15
20
25
30
35
0
500
1000
1500
2000
GRMZM5G808366_T01 position (nts)

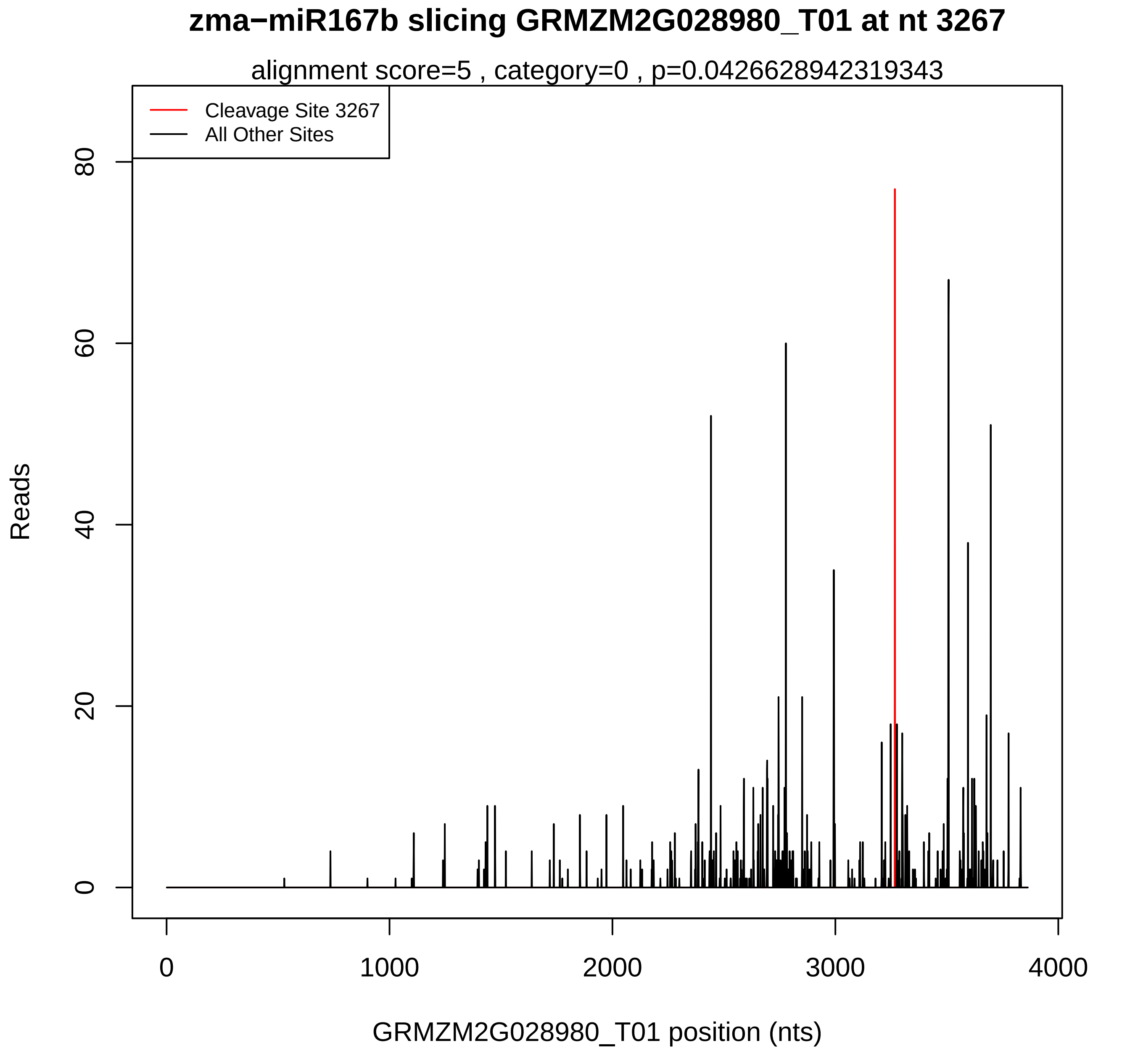
zma−miR167b slicing GRMZM2G028980_T01 at nt 3267
alignment score=5 , category=0 , p=0.0426628942319343
Cleavage Site 3267
All Other Sites
Reads
0
20
40
60
80
0
1000
2000
3000
4000
GRMZM2G028980_T01 position (nts)

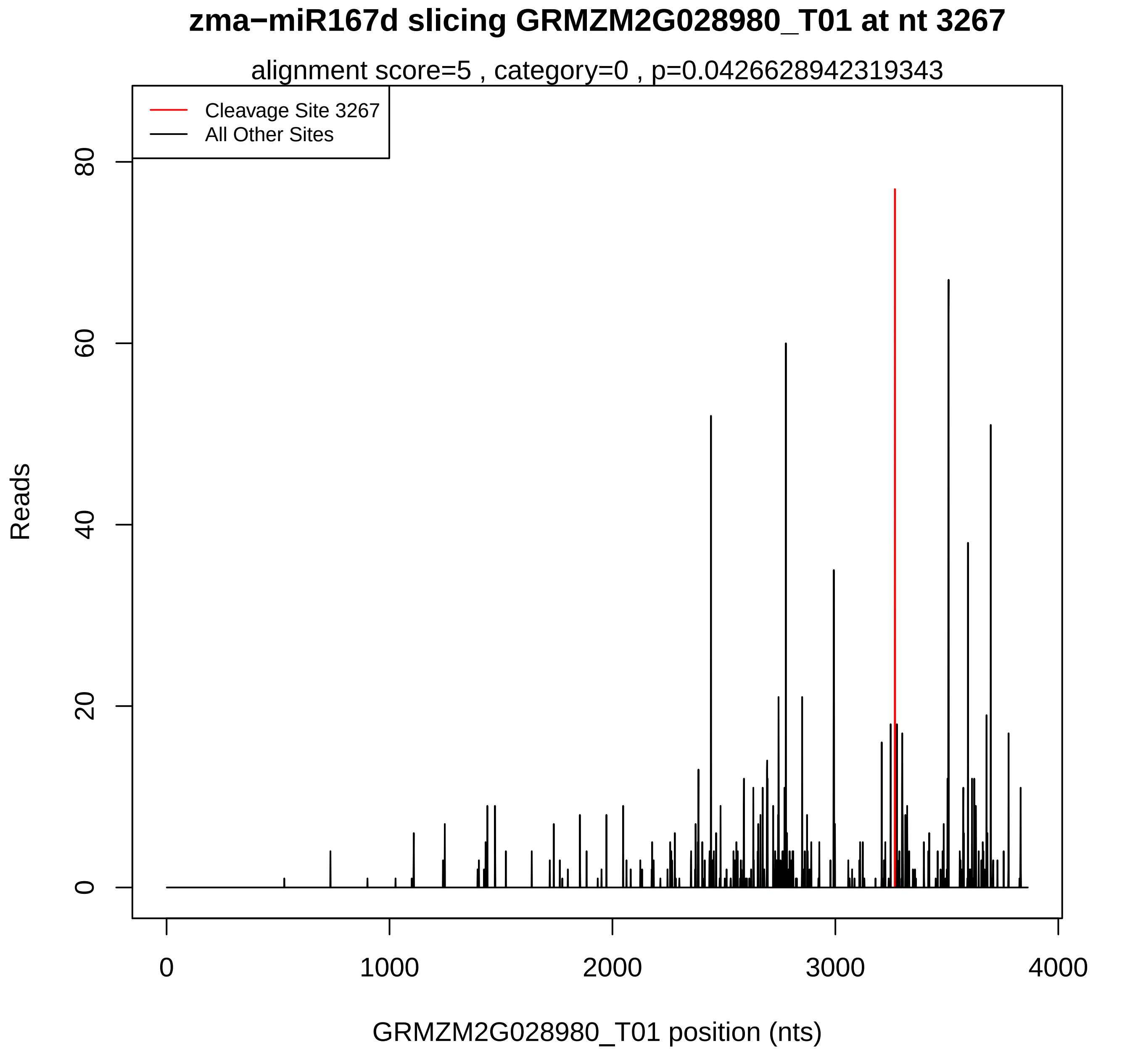

zma−miR167d slicing GRMZM2G028980_T01 at nt 3267
alignment score=5 , category=0 , p=0.0426628942319343
Cleavage Site 3267
All Other Sites
Reads
0
20
40
60
80
0
1000
2000
3000
4000
GRMZM2G028980_T01 position (nts)

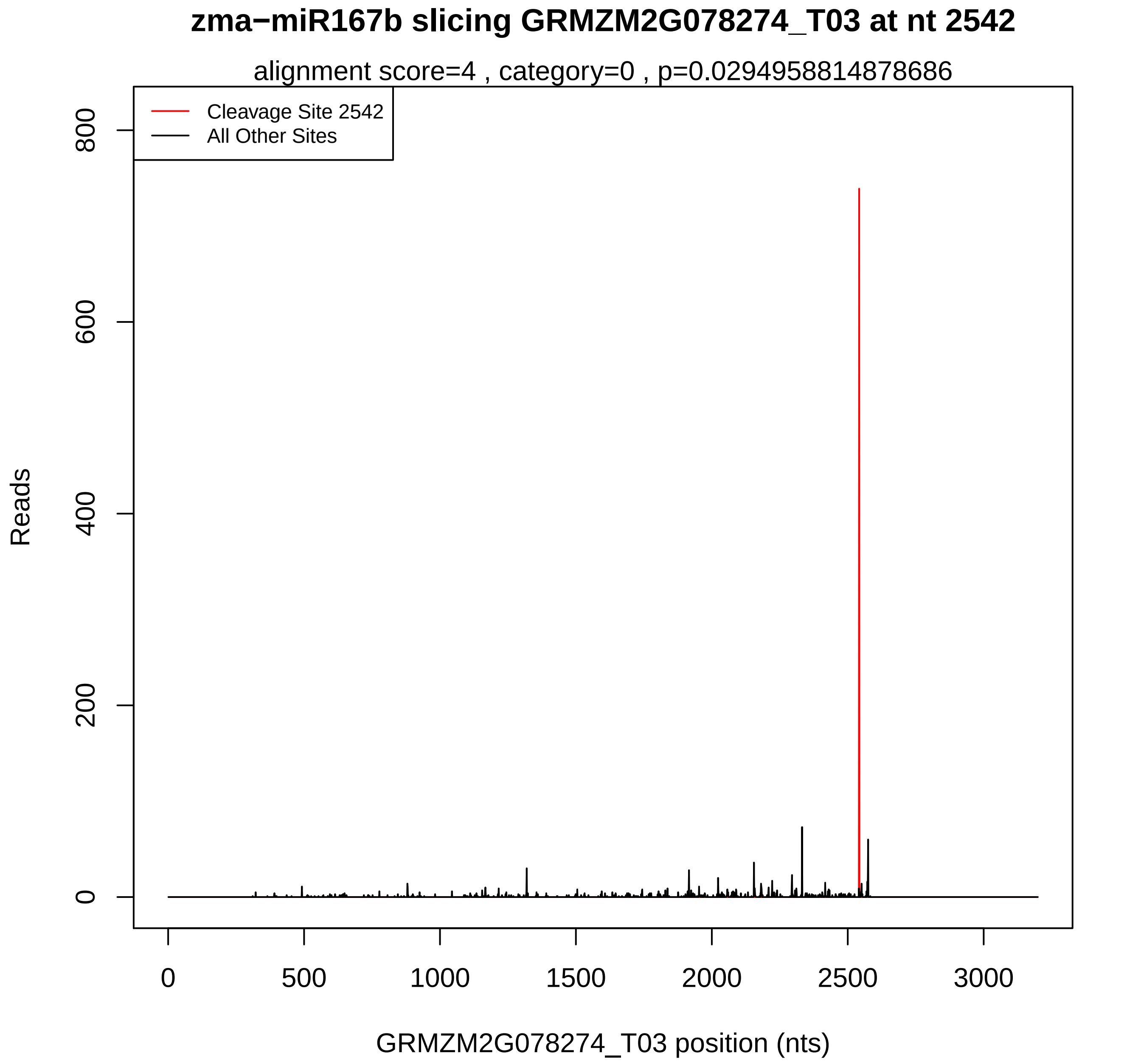
zma−miR167b slicing GRMZM2G078274_T03 at nt 2542
alignment score=4 , category=0 , p=0.0294958814878686
Cleavage Site 2542
All Other Sites
Reads
0
200
400
600
800
0
500
1000
1500
2000
2500
3000
GRMZM2G078274_T03 position (nts)

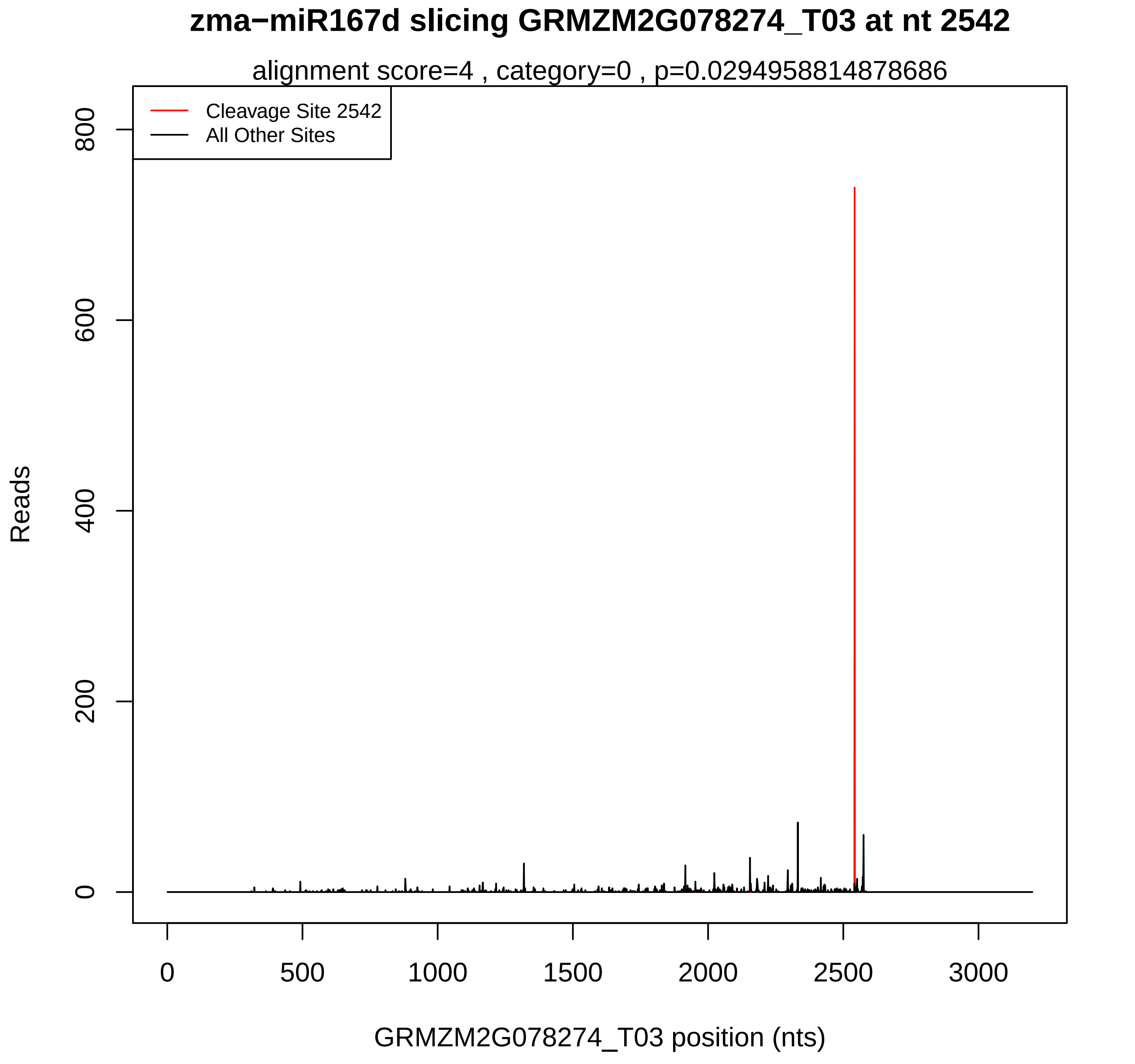
zma−miR167d slicing GRMZM2G078274_T03 at nt 2542
alignment score=4 , category=0 , p=0.0294958814878686
Cleavage Site 2542
All Other Sites
Reads
0
200
400
600
800
0
500
1000
1500
2000
2500
3000
GRMZM2G078274_T03 position (nts)

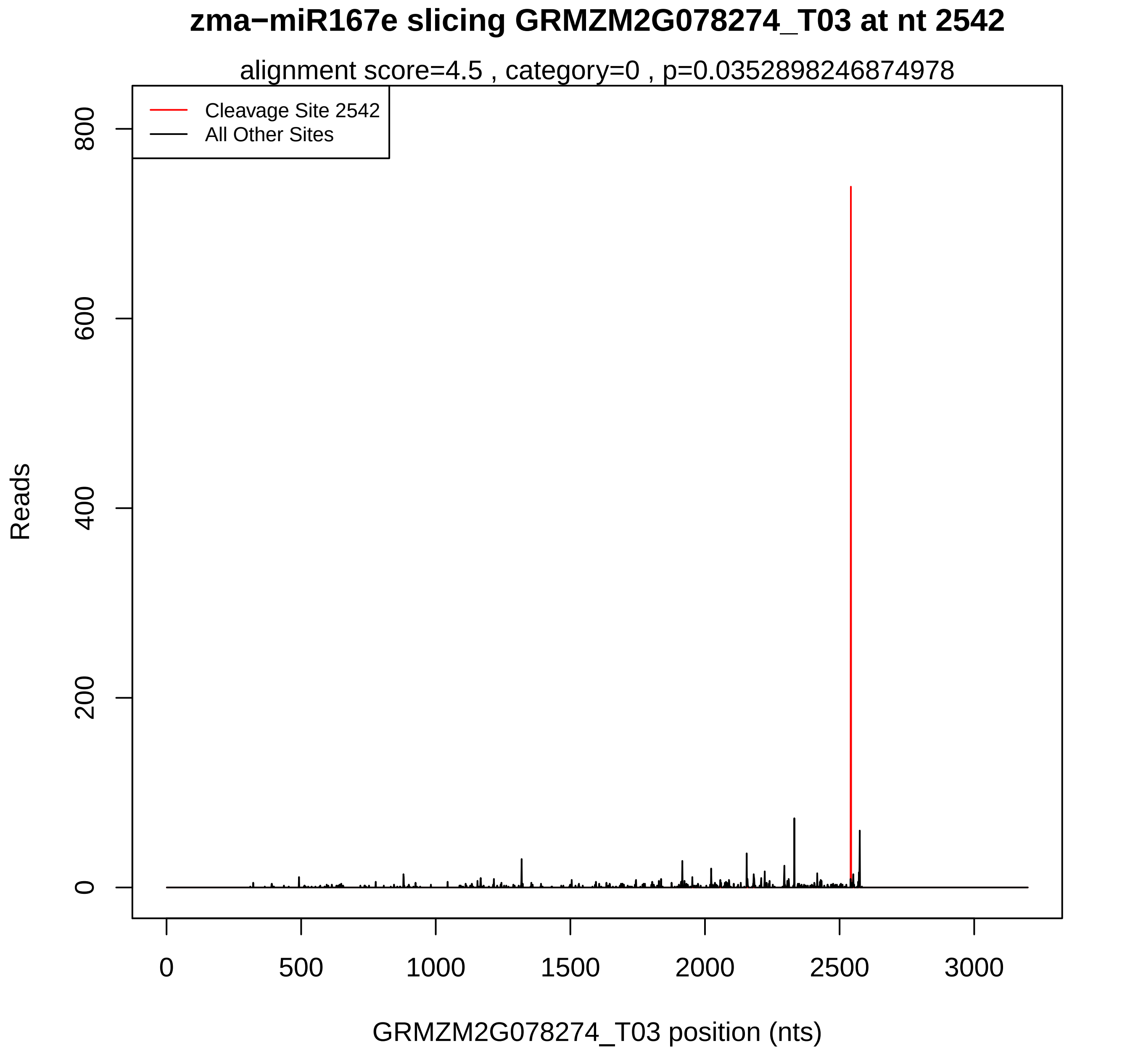
zma−miR167e slicing GRMZM2G078274_T03 at nt 2542
alignment score=4.5 , category=0 , p=0.0352898246874978
Cleavage Site 2542
All Other Sites
Reads
0
200
400
600
800
0
500
1000
1500
2000
2500
3000
GRMZM2G078274_T03 position (nts)

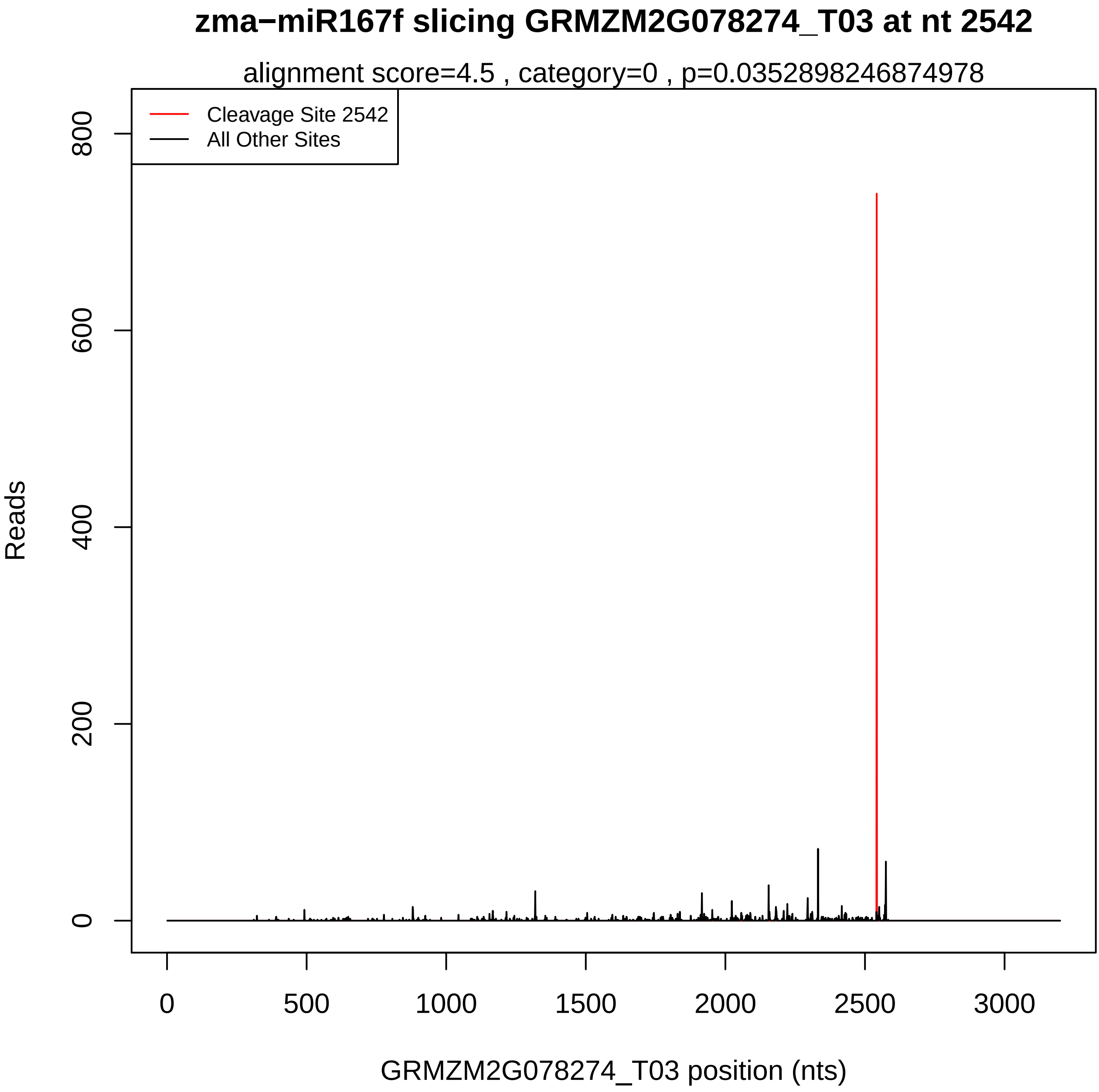
zma−miR167f slicing GRMZM2G078274_T03 at nt 2542
alignment score=4.5 , category=0 , p=0.0352898246874978
Cleavage Site 2542
All Other Sites
Reads
0
200
400
600
800
0
500
1000
1500
2000
2500
3000
GRMZM2G078274_T03 position (nts)

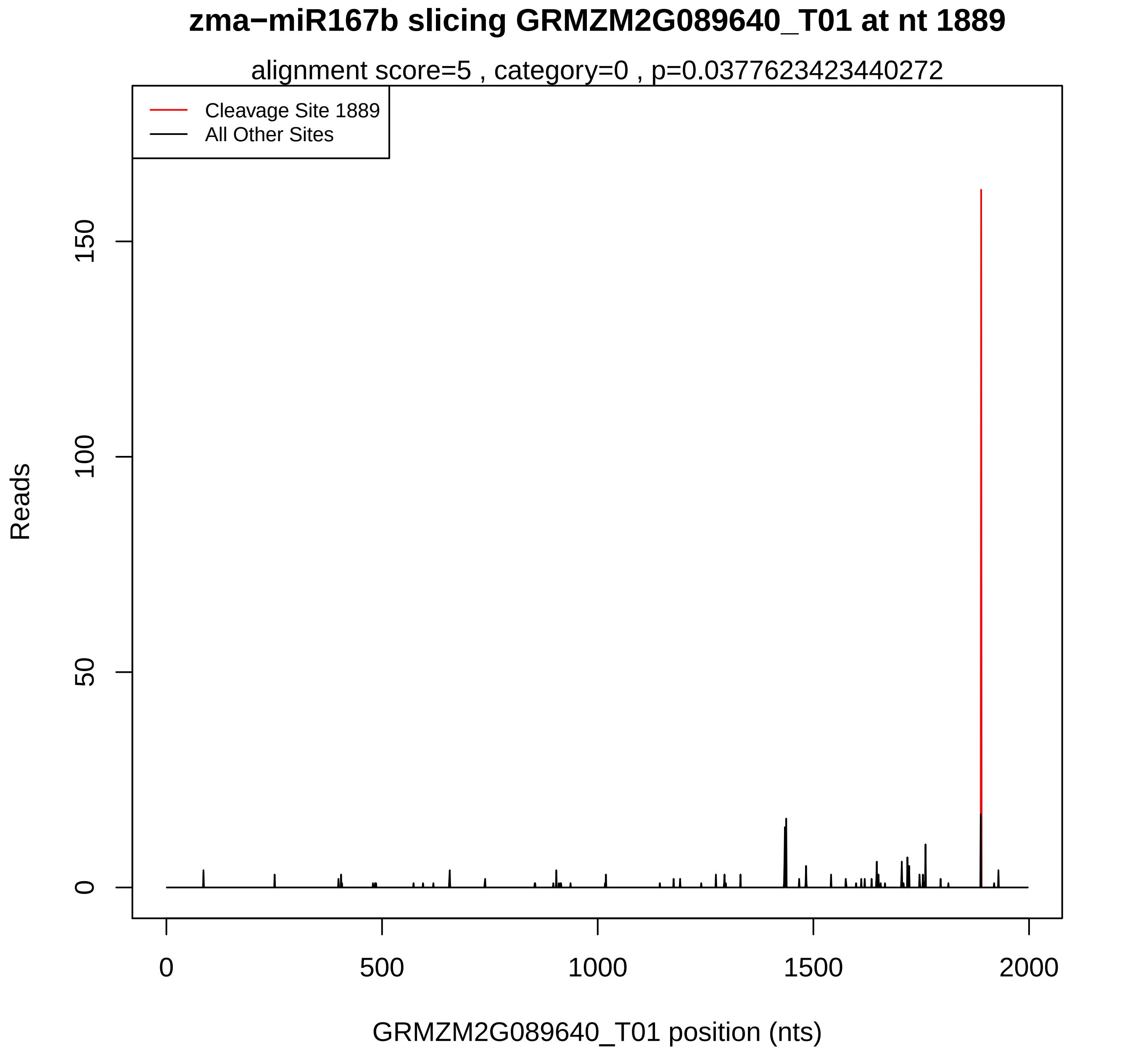

zma−miR167b slicing GRMZM2G089640_T01 at nt 1889
alignment score=5 , category=0 , p=0.0377623423440272
Cleavage Site 1889
All Other Sites
Reads
0
50
100
150
0
500
1000
1500
2000
GRMZM2G089640_T01 position (nts)

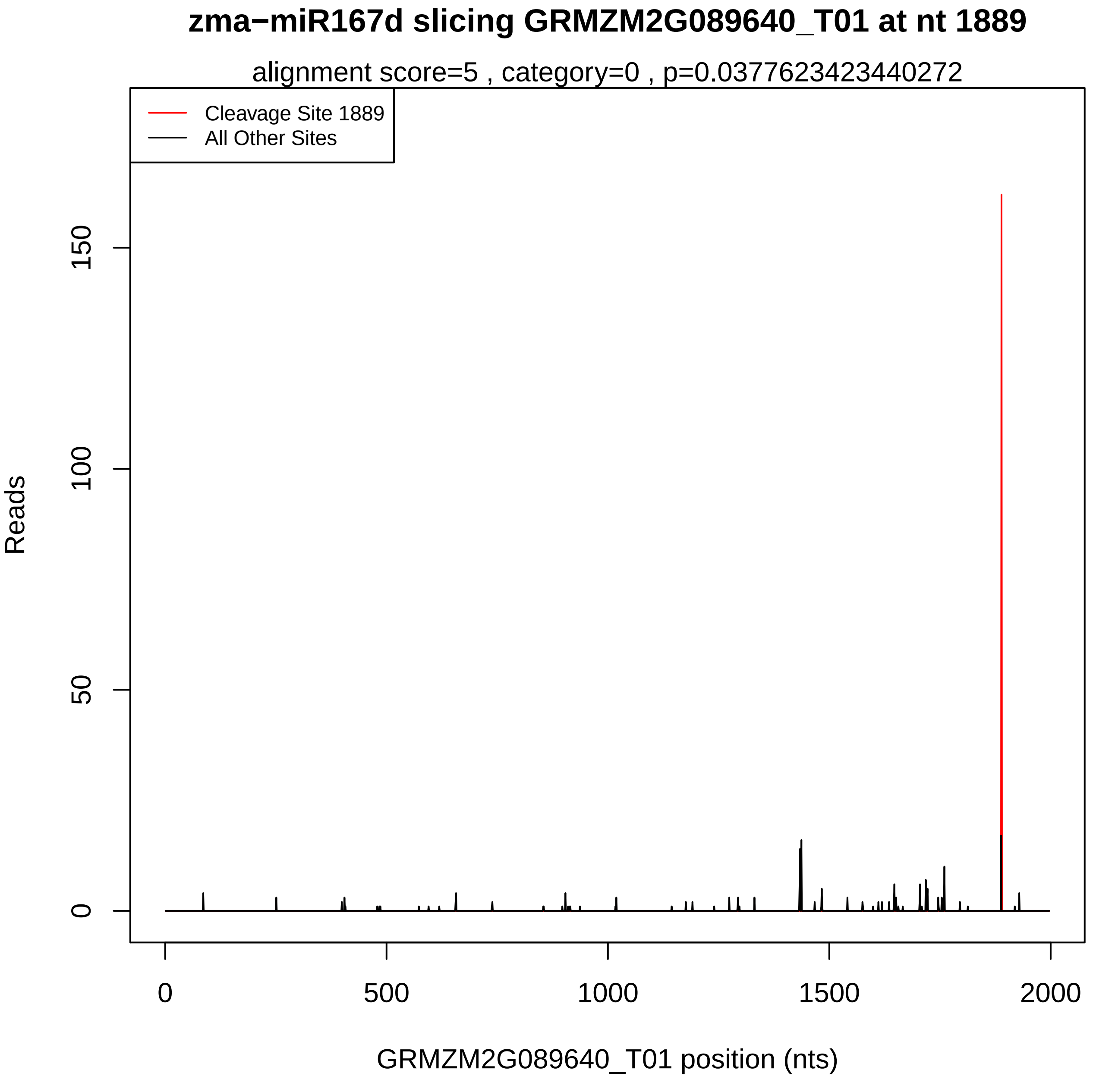
zma−miR167d slicing GRMZM2G089640_T01 at nt 1889
alignment score=5 , category=0 , p=0.0377623423440272
Cleavage Site 1889
All Other Sites
Reads
0
50
100
150
0
500
1000
1500
2000
GRMZM2G089640_T01 position (nts)

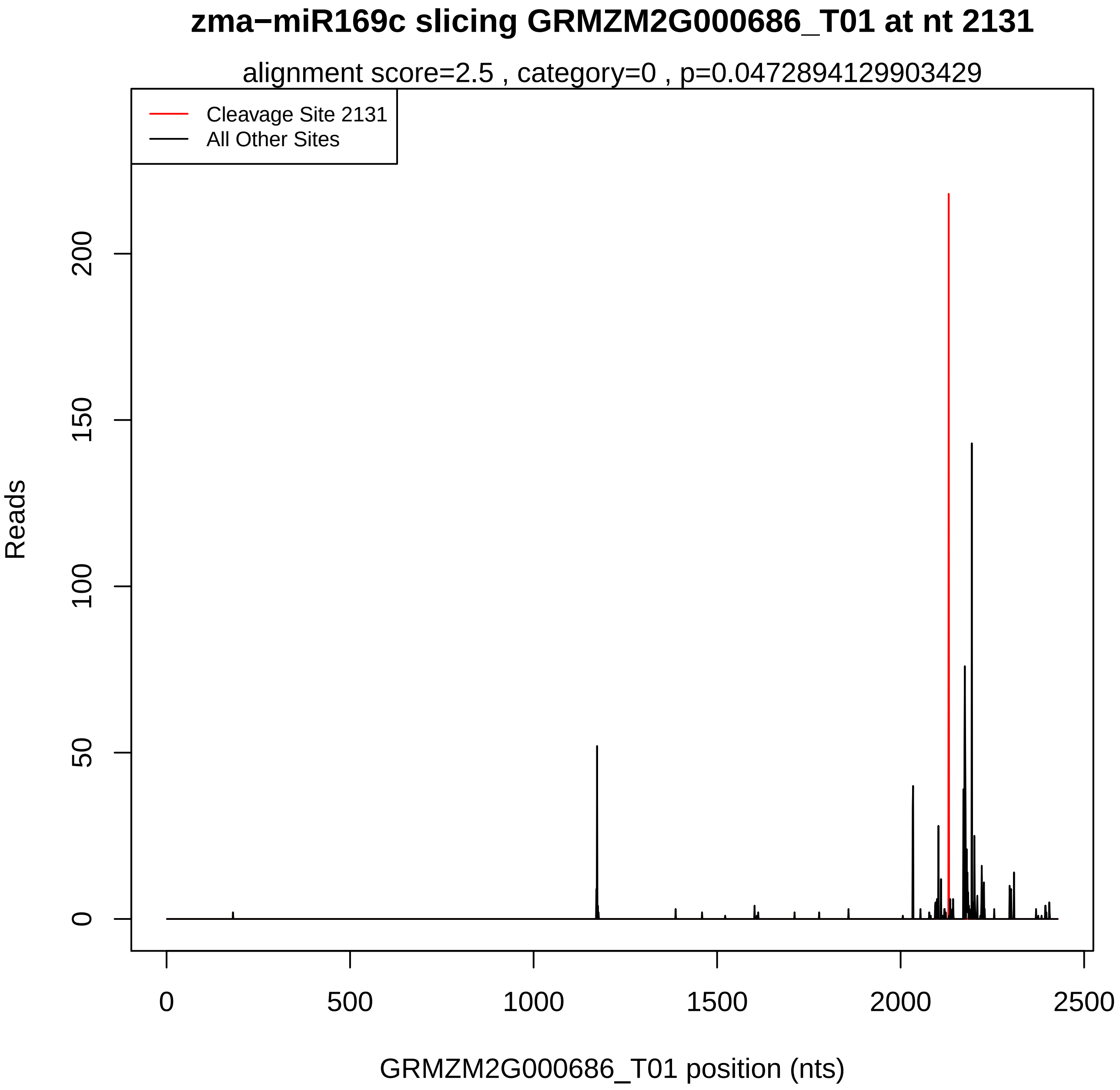

zma−miR169c slicing GRMZM2G000686_T01 at nt 2131
alignment score=2.5 , category=0 , p=0.0472894129903429
Cleavage Site 2131
All Other Sites
Reads
0
50
100
150
200
0
500
1000
1500
2000
2500
GRMZM2G000686_T01 position (nts)

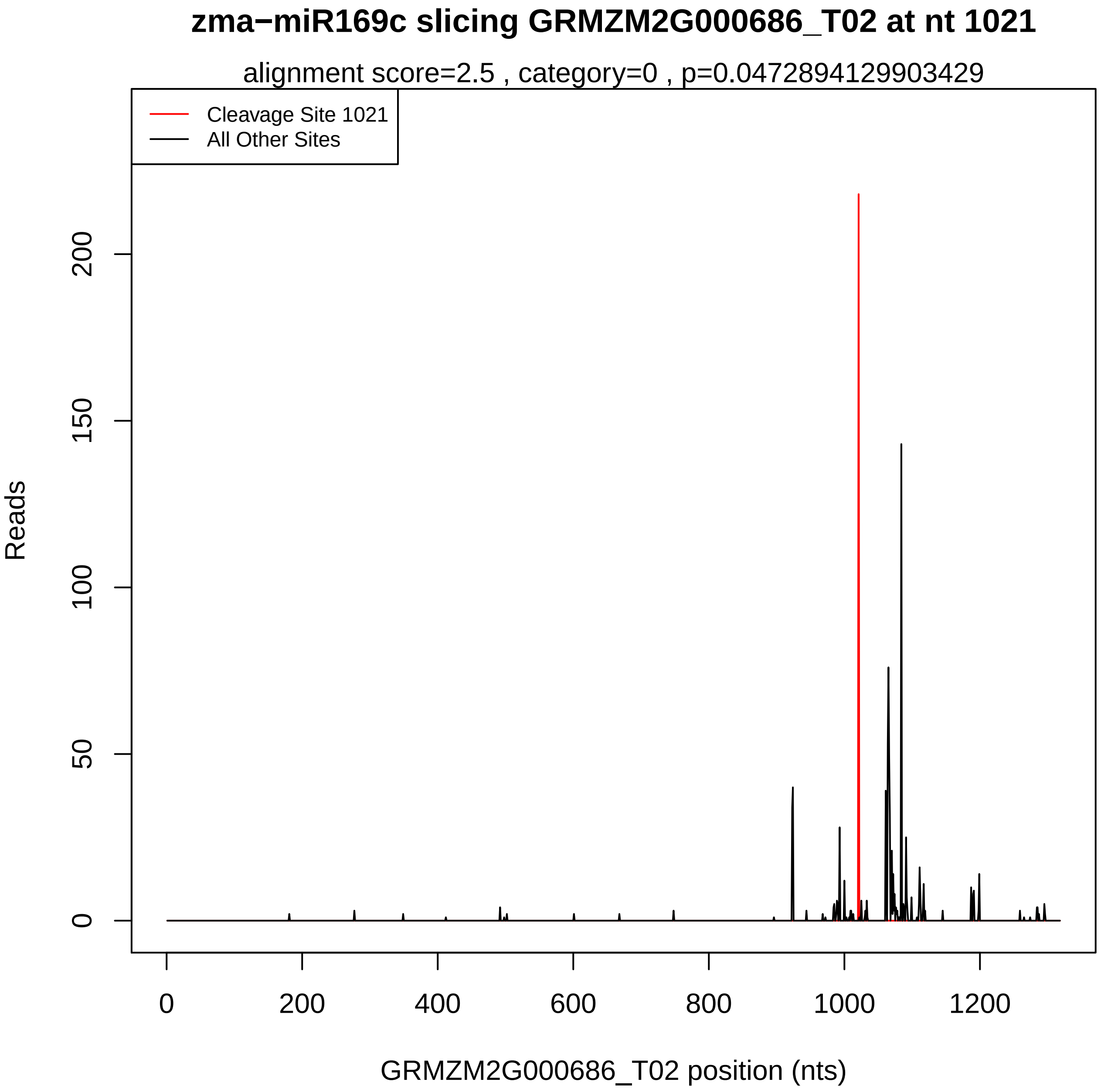

zma−miR169c slicing GRMZM2G000686_T02 at nt 1021
alignment score=2.5 , category=0 , p=0.0472894129903429
Cleavage Site 1021
All Other Sites
Reads
0
50
100
150
200
0
200
400
600
800
1000
1200
GRMZM2G000686_T02 position (nts)

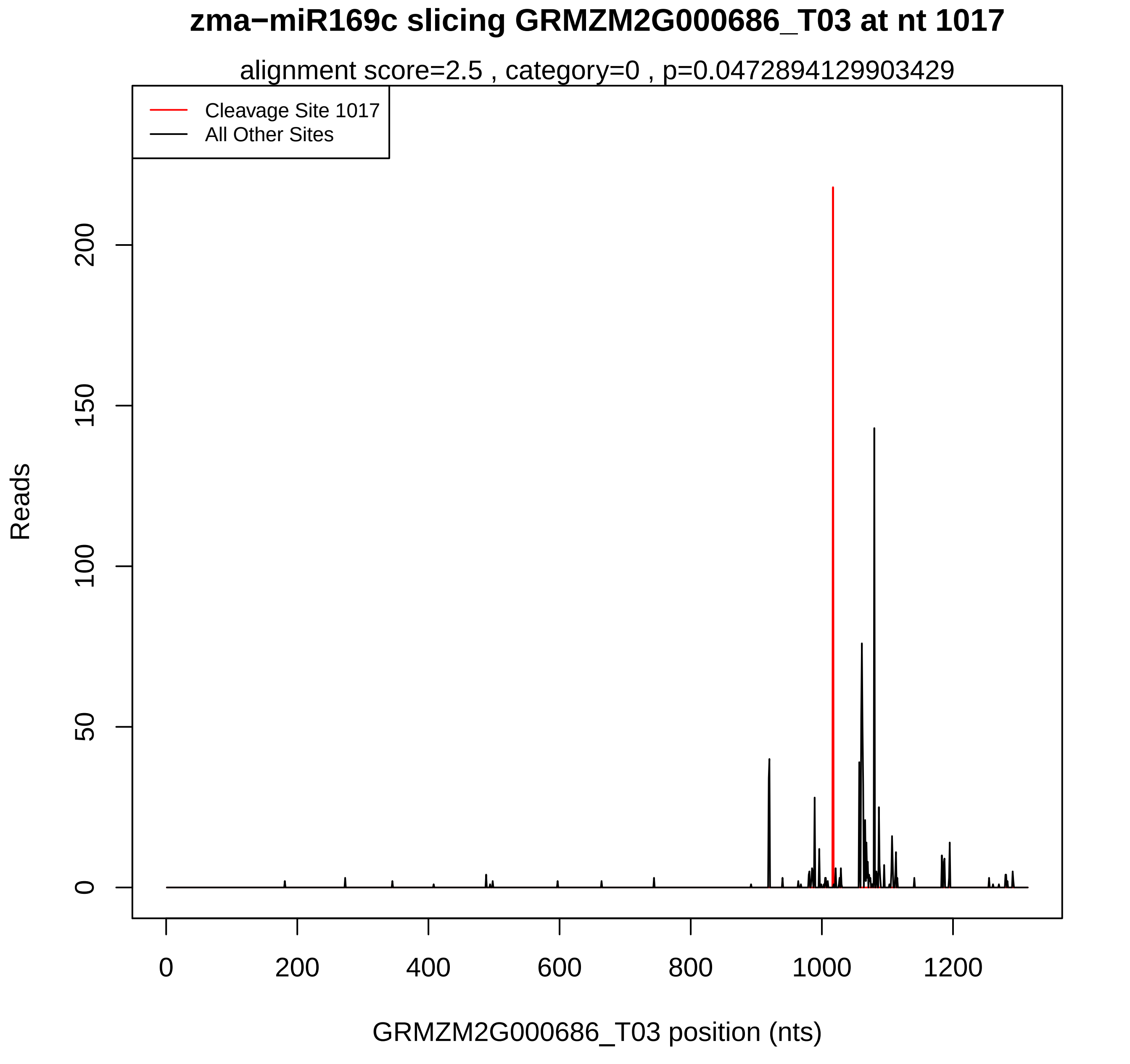
zma−miR169c slicing GRMZM2G000686_T03 at nt 1017
alignment score=2.5 , category=0 , p=0.0472894129903429
Cleavage Site 1017
All Other Sites
Reads
0
50
100
150
200
0
200
400
600
800
1000
1200
GRMZM2G000686_T03 position (nts)

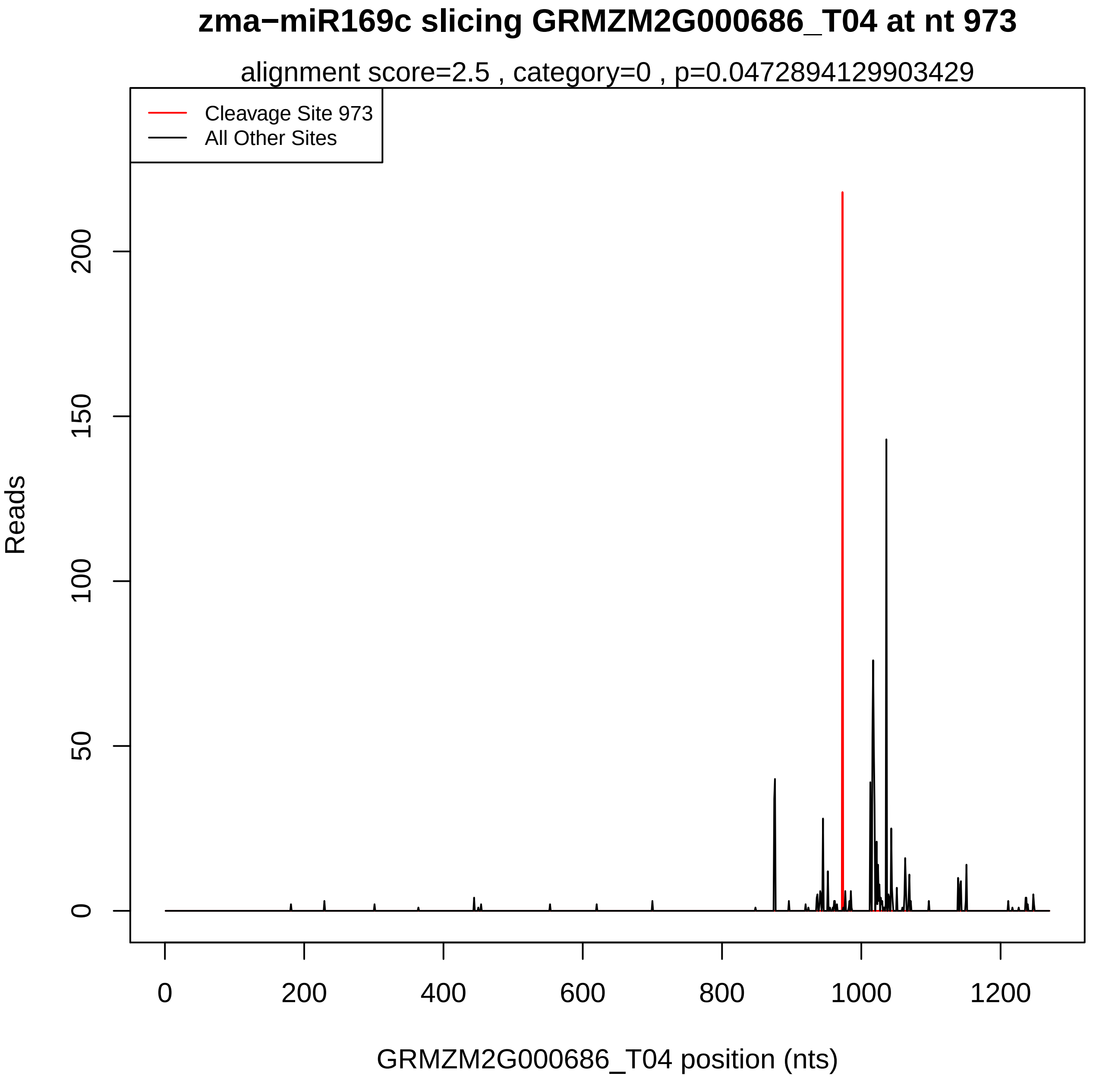
zma−miR169c slicing GRMZM2G000686_T04 at nt 973
alignment score=2.5 , category=0 , p=0.0472894129903429
Cleavage Site 973
All Other Sites
Reads
0
50
100
150
200
0
200
400
600
800
1000
1200
GRMZM2G000686_T04 position (nts)

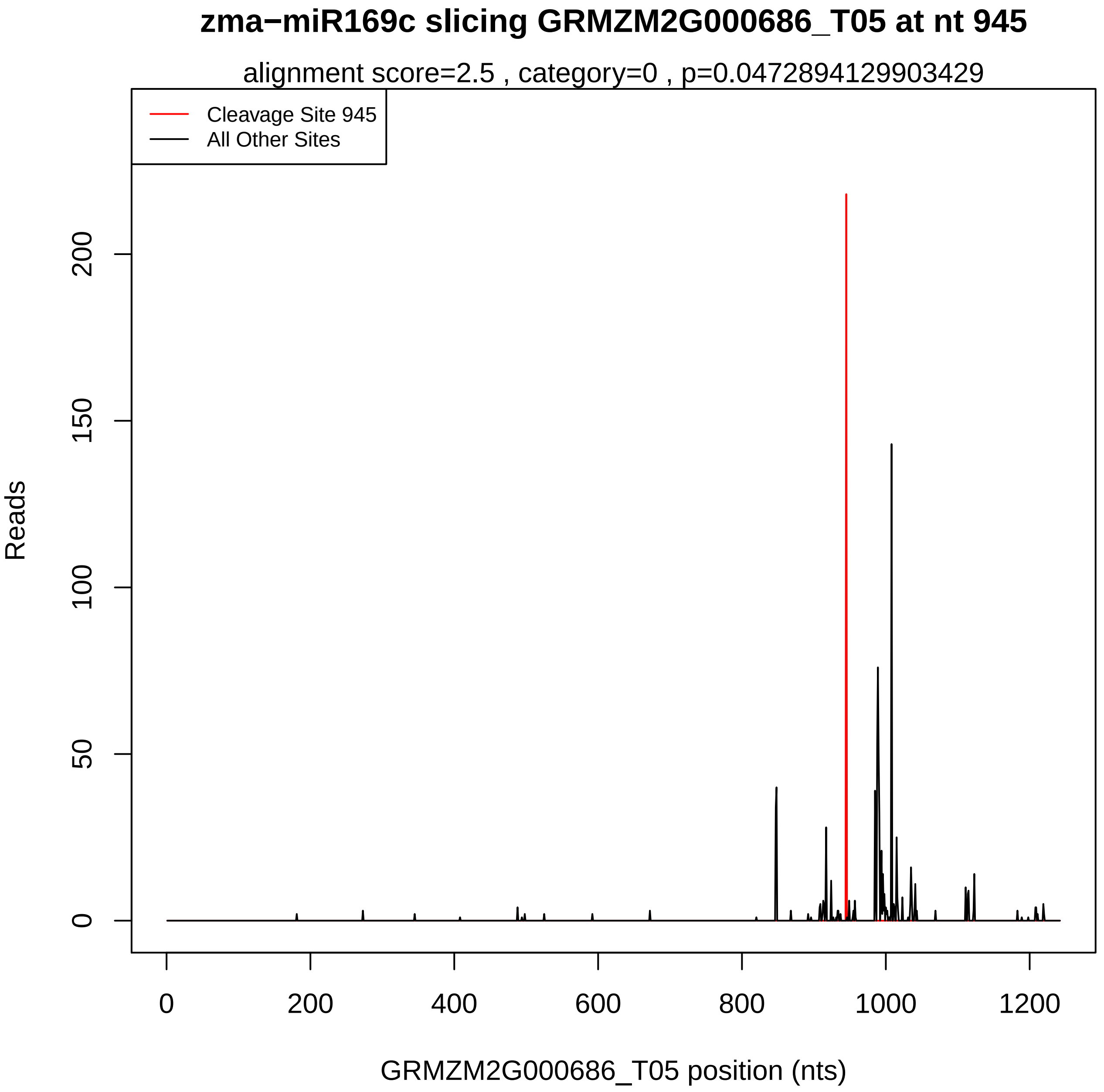
zma−miR169c slicing GRMZM2G000686_T05 at nt 945
alignment score=2.5 , category=0 , p=0.0472894129903429
Cleavage Site 945
All Other Sites
Reads
0
50
100
150
200
0
200
400
600
800
1000
1200
GRMZM2G000686_T05 position (nts)

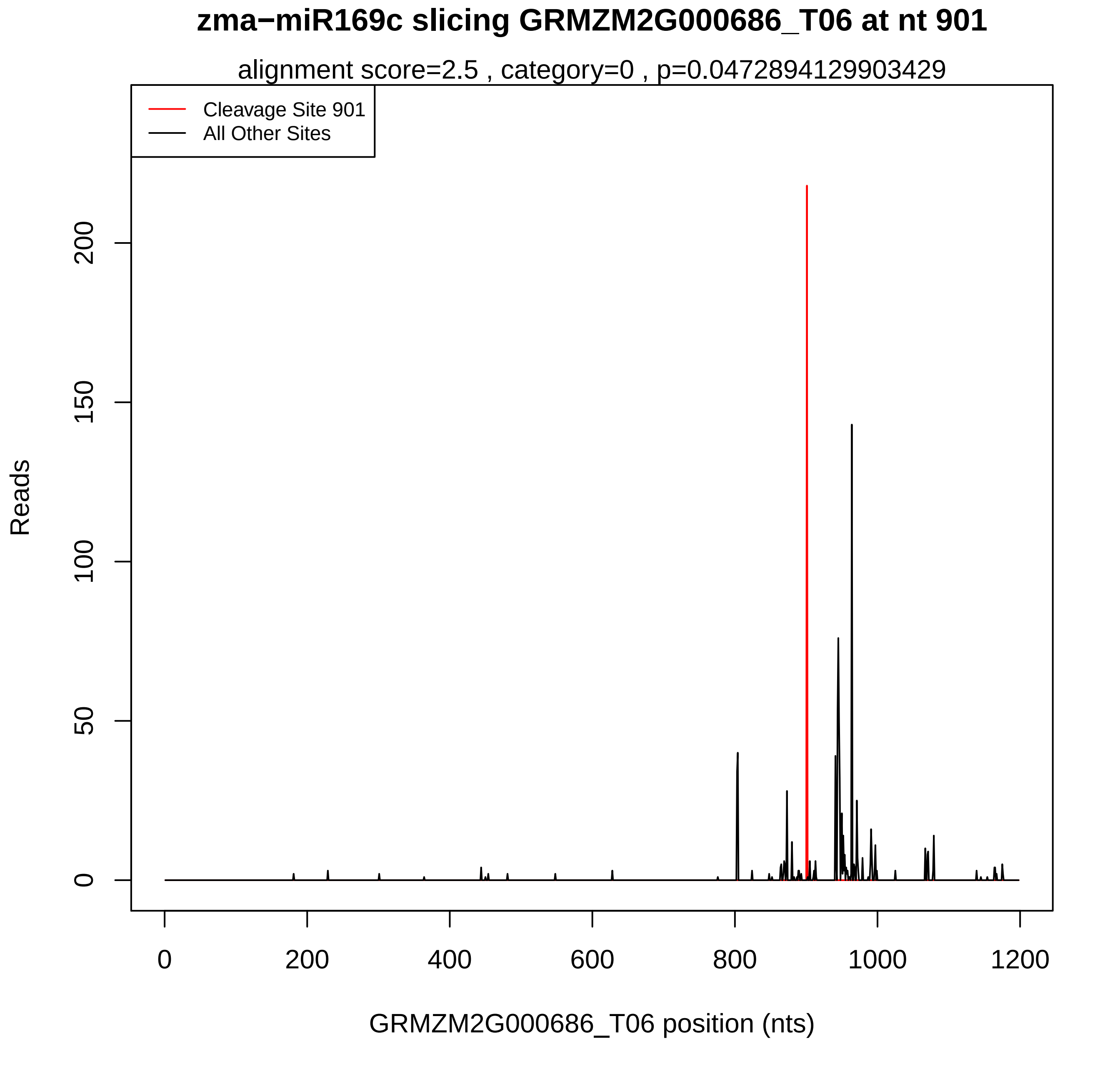
zma−miR169c slicing GRMZM2G000686_T06 at nt 901
alignment score=2.5 , category=0 , p=0.0472894129903429
Cleavage Site 901
All Other Sites
Reads
0
50
100
150
200
0
200
400
600
800
1000
1200
GRMZM2G000686_T06 position (nts)

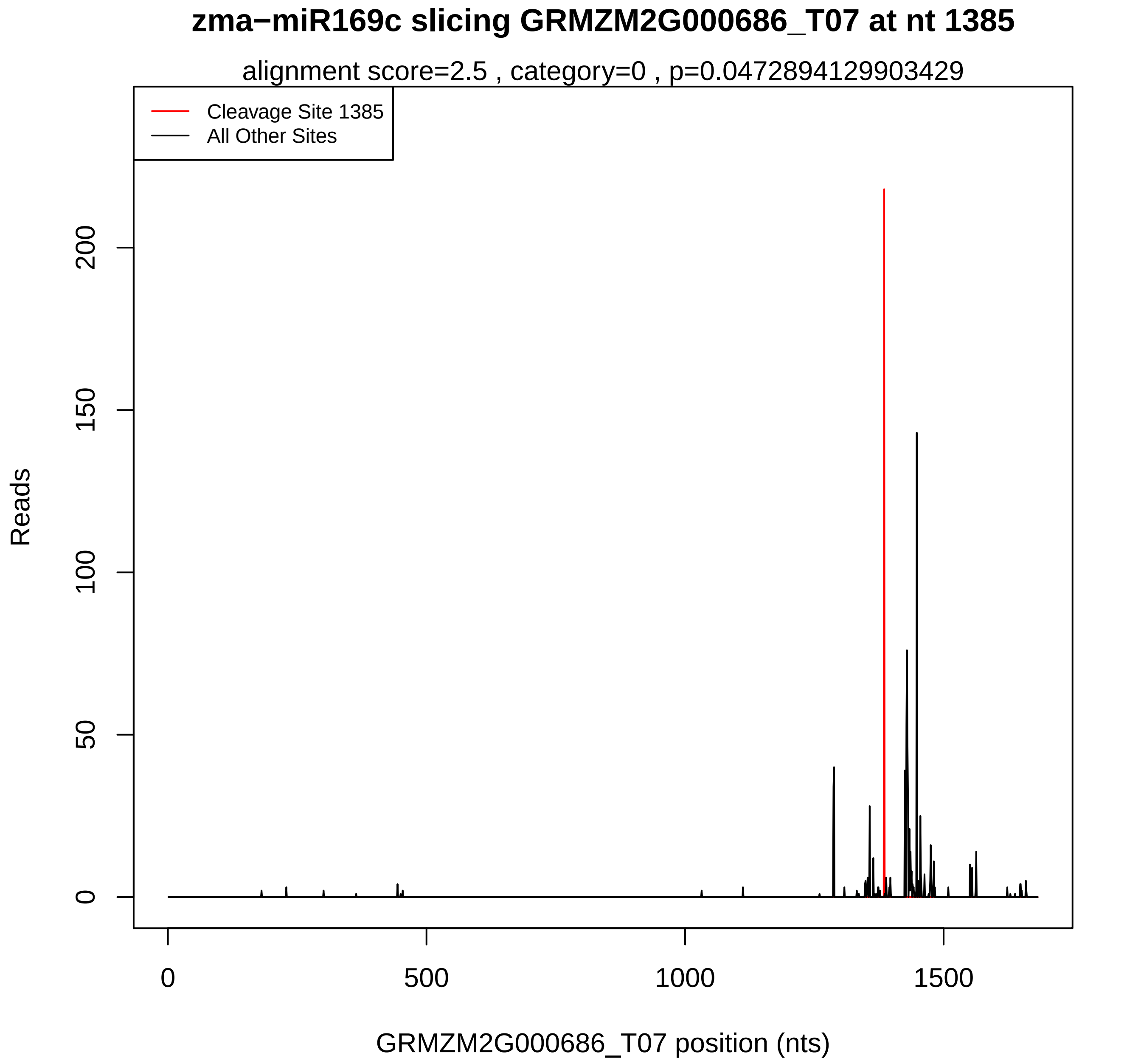
zma−miR169c slicing GRMZM2G000686_T07 at nt 1385
alignment score=2.5 , category=0 , p=0.0472894129903429
Cleavage Site 1385
All Other Sites
Reads
0
50
100
150
200
0
500
1000
1500
GRMZM2G000686_T07 position (nts)

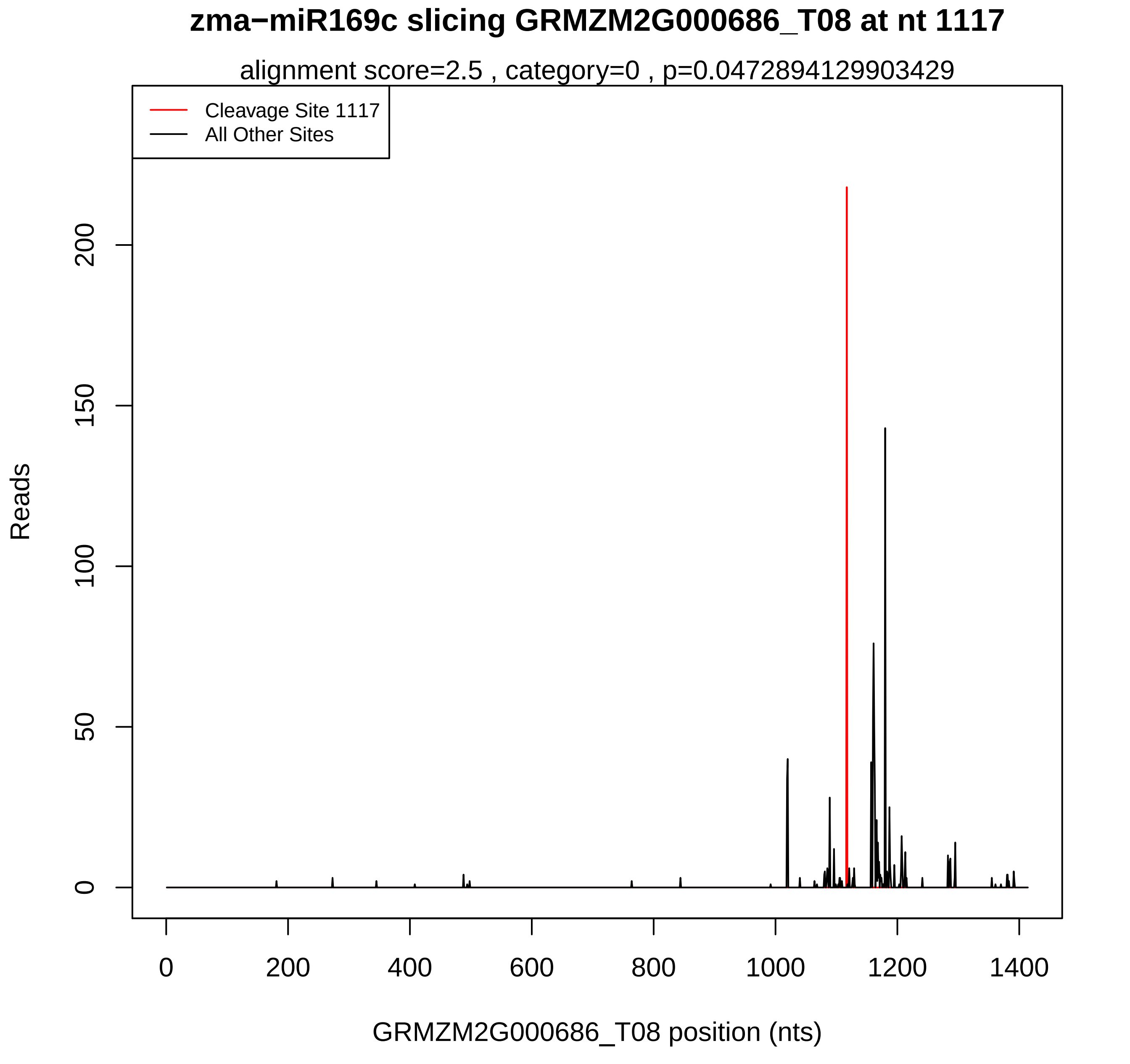
zma−miR169c slicing GRMZM2G000686_T08 at nt 1117
alignment score=2.5 , category=0 , p=0.0472894129903429
Cleavage Site 1117
All Other Sites
Reads
0
50
100
150
200
0
200
400
600
800
1000
1200
1400
GRMZM2G000686_T08 position (nts)

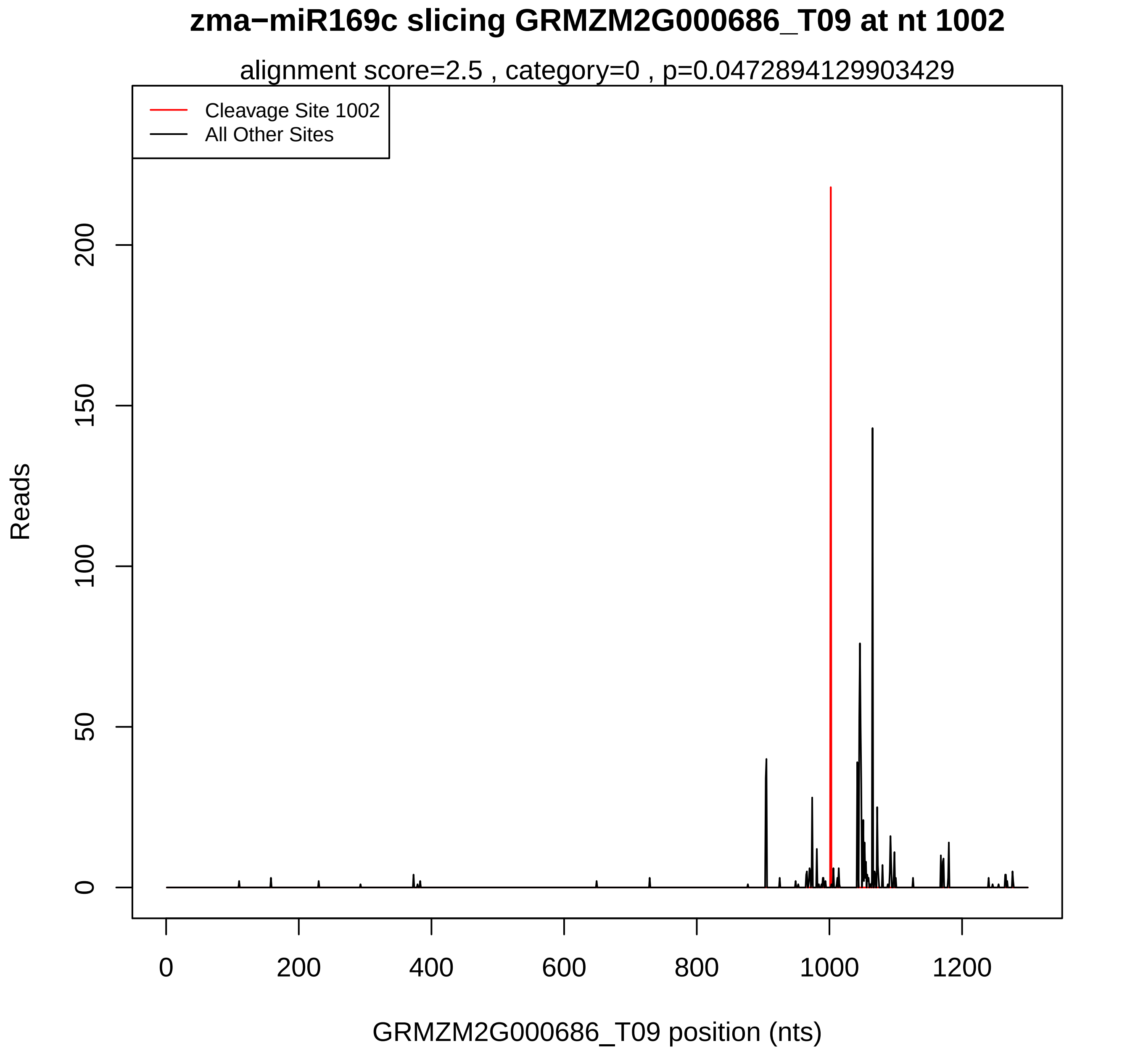
zma−miR169c slicing GRMZM2G000686_T09 at nt 1002
alignment score=2.5 , category=0 , p=0.0472894129903429
Cleavage Site 1002
All Other Sites
Reads
0
50
100
150
200
0
200
400
600
800
1000
1200
GRMZM2G000686_T09 position (nts)

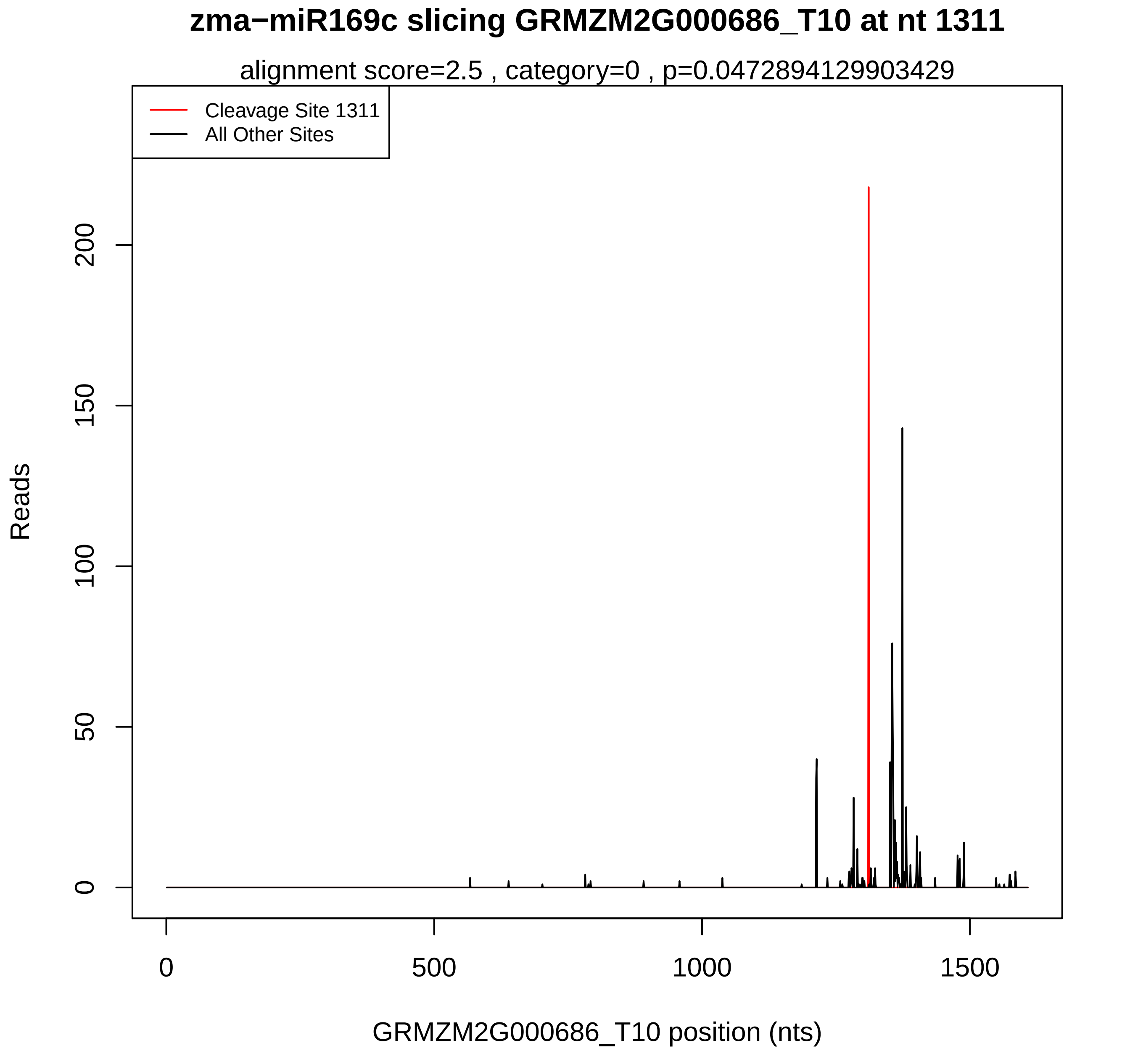
zma−miR169c slicing GRMZM2G000686_T10 at nt 1311
alignment score=2.5 , category=0 , p=0.0472894129903429
Cleavage Site 1311
All Other Sites
Reads
0
50
100
150
200
0
500
1000
1500
GRMZM2G000686_T10 position (nts)

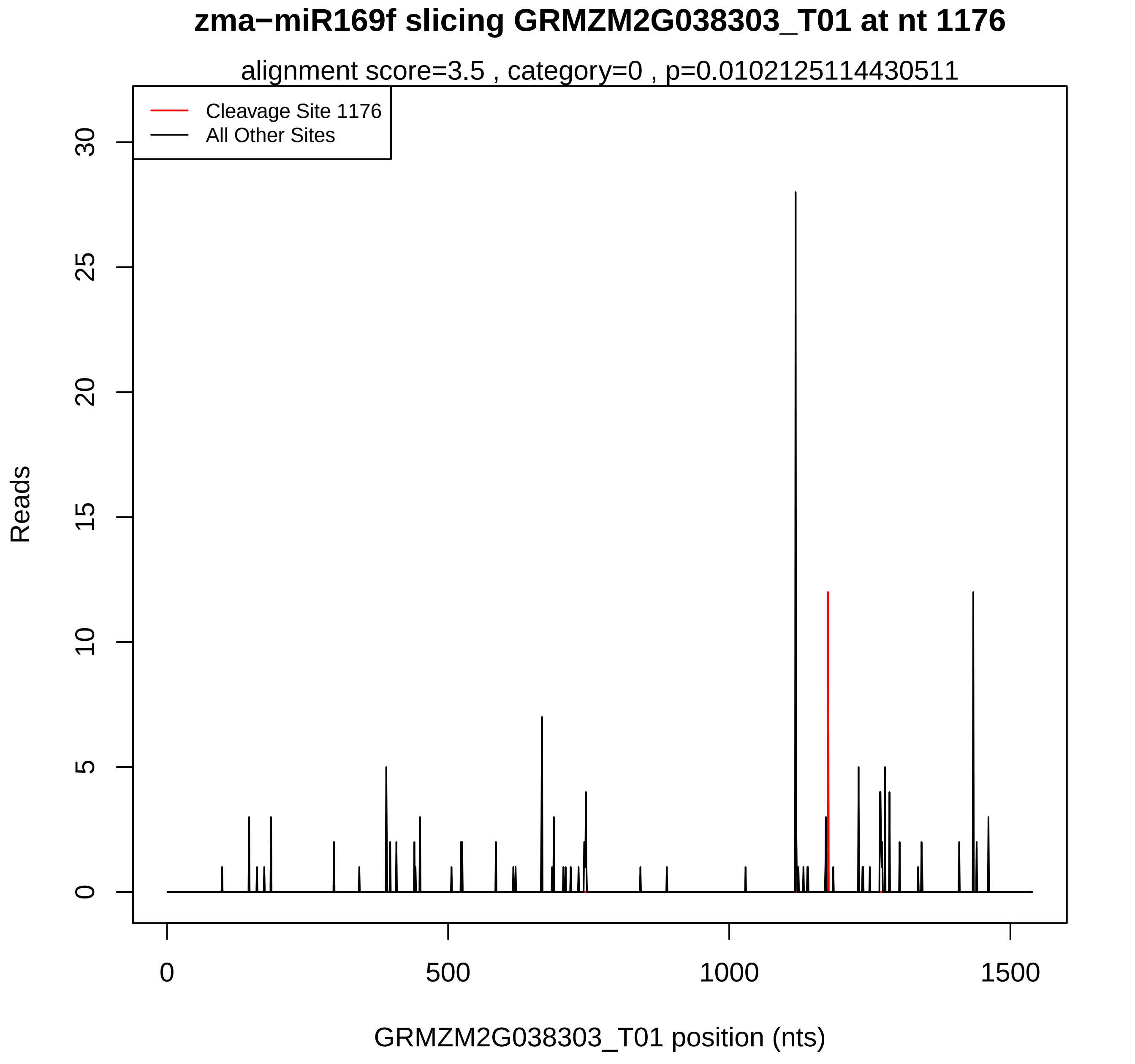
zma−miR169f slicing GRMZM2G038303_T01 at nt 1176
alignment score=3.5 , category=0 , p=0.0102125114430511
Cleavage Site 1176
All Other Sites
Reads
0
5
10
15
20
25
30
0
500
1000
1500
GRMZM2G038303_T01 position (nts)

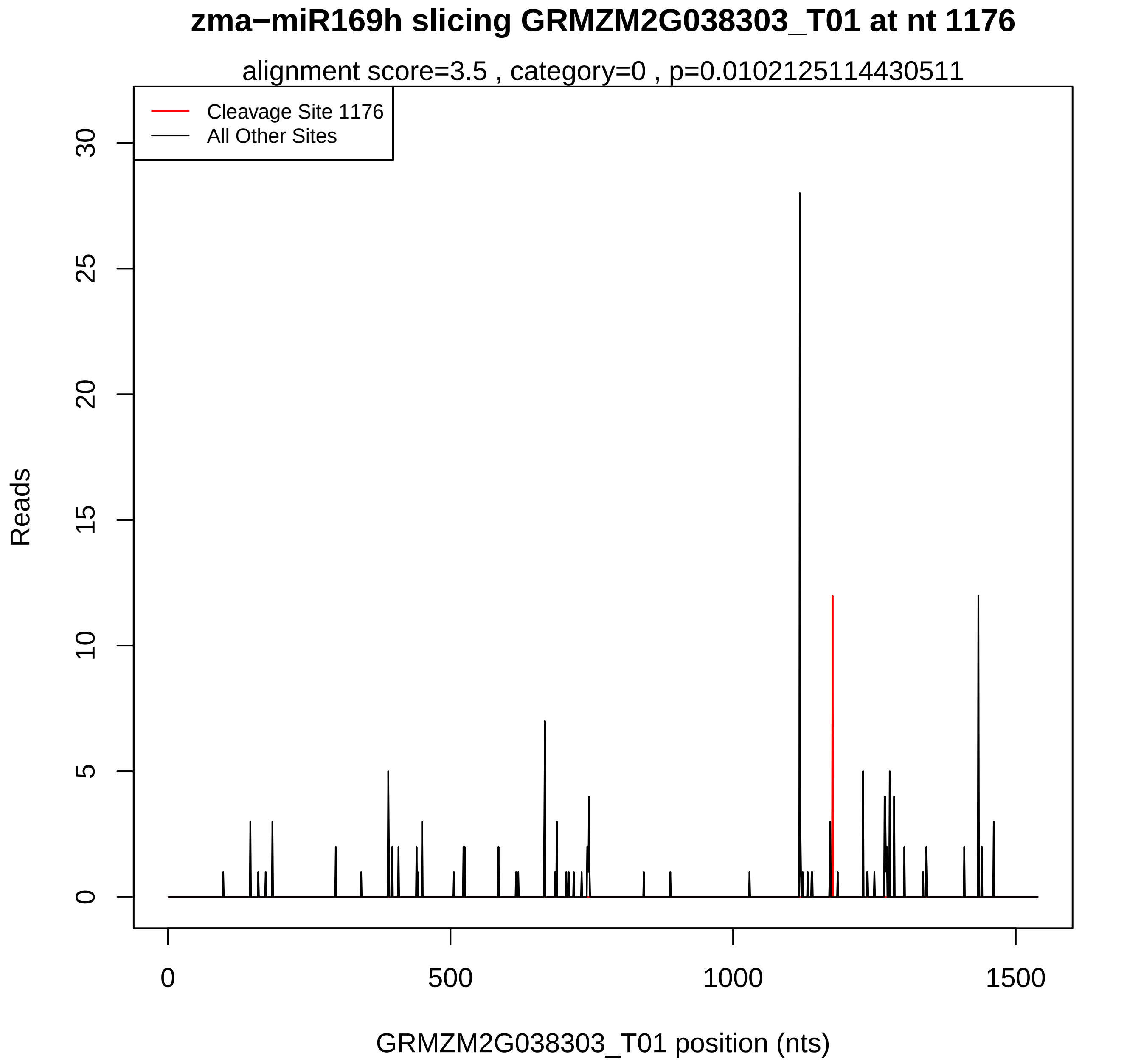

zma−miR169h slicing GRMZM2G038303_T01 at nt 1176
alignment score=3.5 , category=0 , p=0.0102125114430511
Cleavage Site 1176
All Other Sites
Reads
0
5
10
15
20
25
30
0
500
1000
1500
GRMZM2G038303_T01 position (nts)

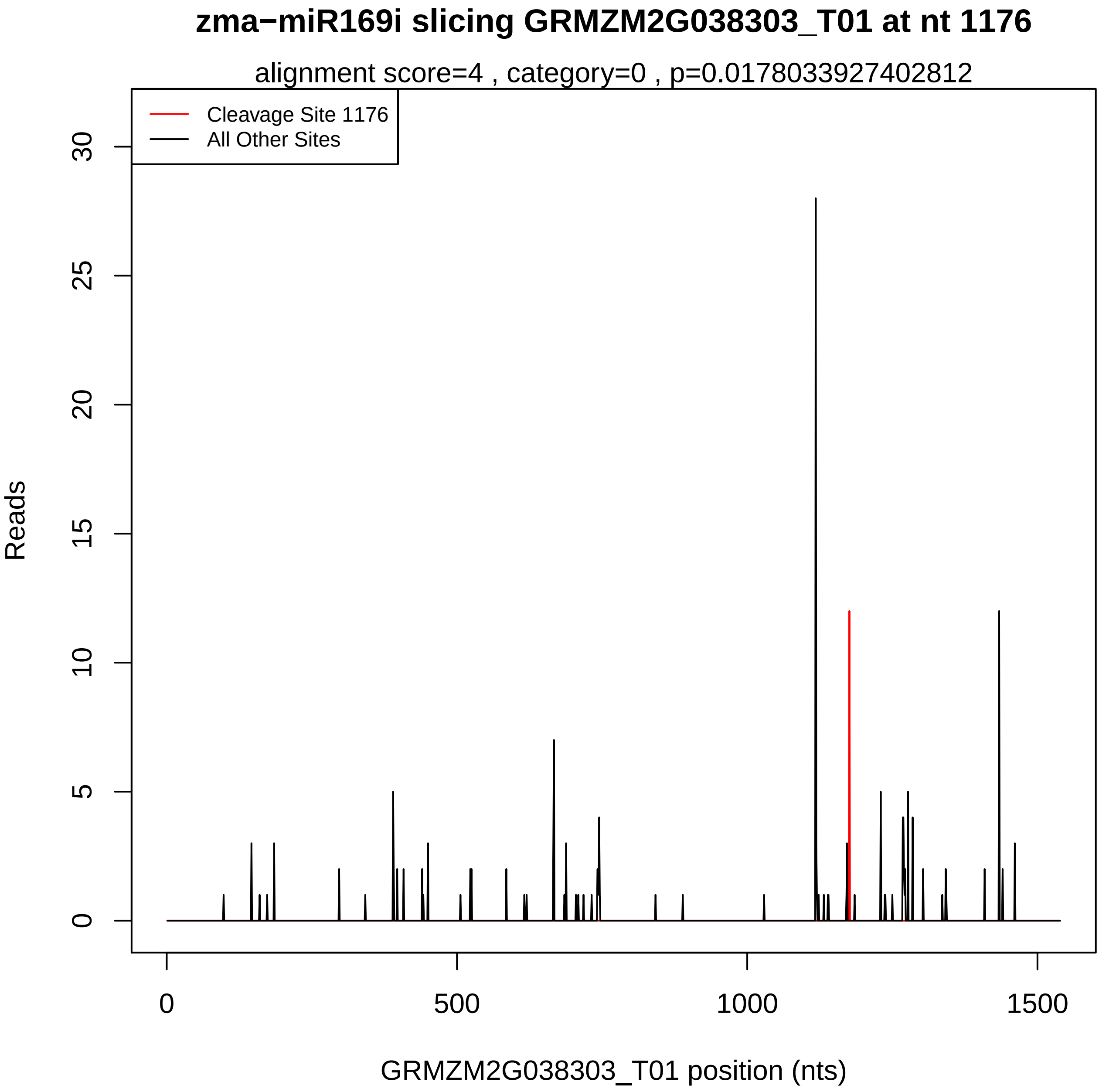
zma−miR169i slicing GRMZM2G038303_T01 at nt 1176
alignment score=4 , category=0 , p=0.0178033927402812
Cleavage Site 1176
All Other Sites
Reads
0
5
10
15
20
25
30
0
500
1000
1500
GRMZM2G038303_T01 position (nts)

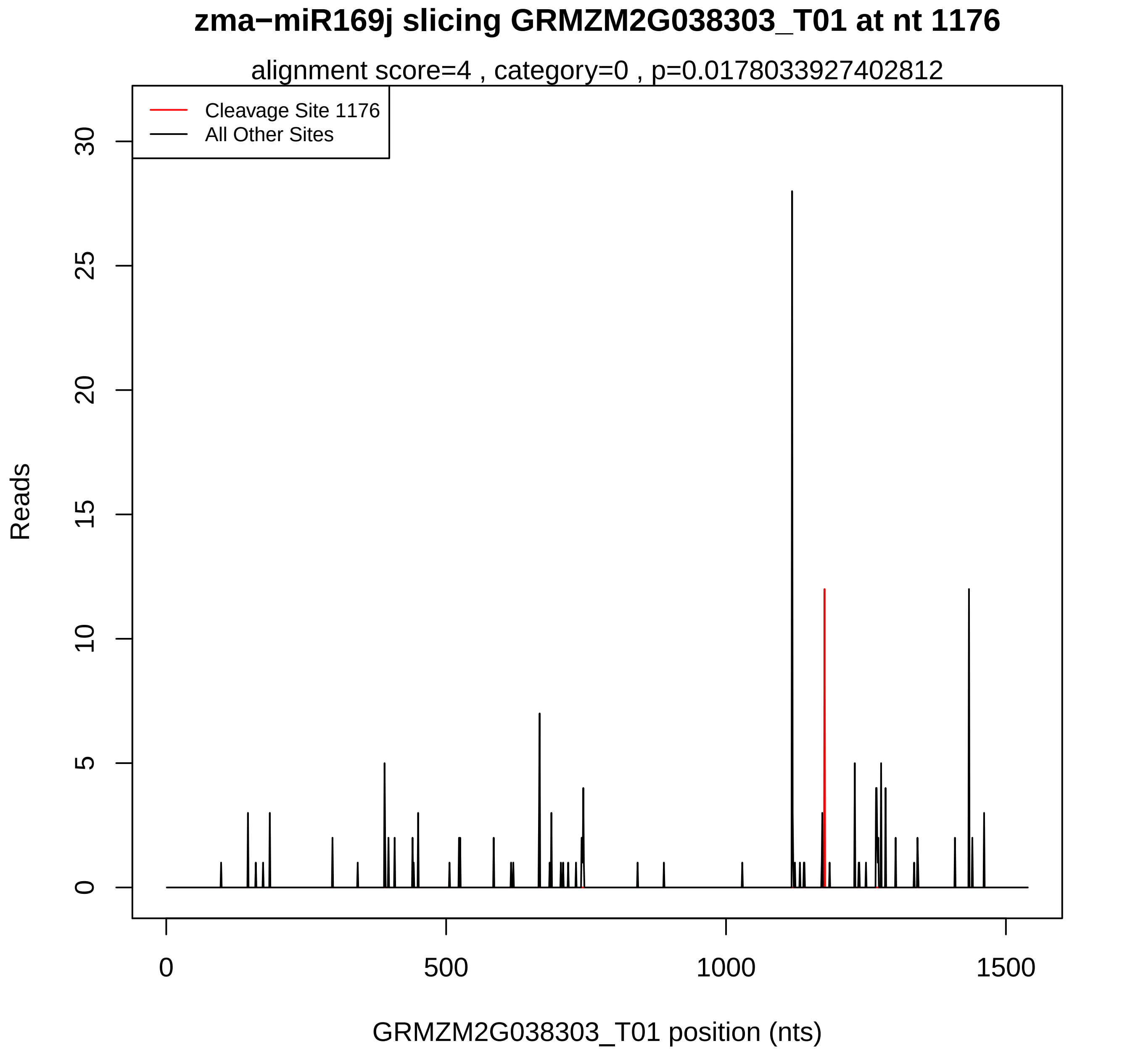
zma−miR169j slicing GRMZM2G038303_T01 at nt 1176
alignment score=4 , category=0 , p=0.0178033927402812
Cleavage Site 1176
All Other Sites
Reads
0
5
10
15
20
25
30
0
500
1000
1500
GRMZM2G038303_T01 position (nts)

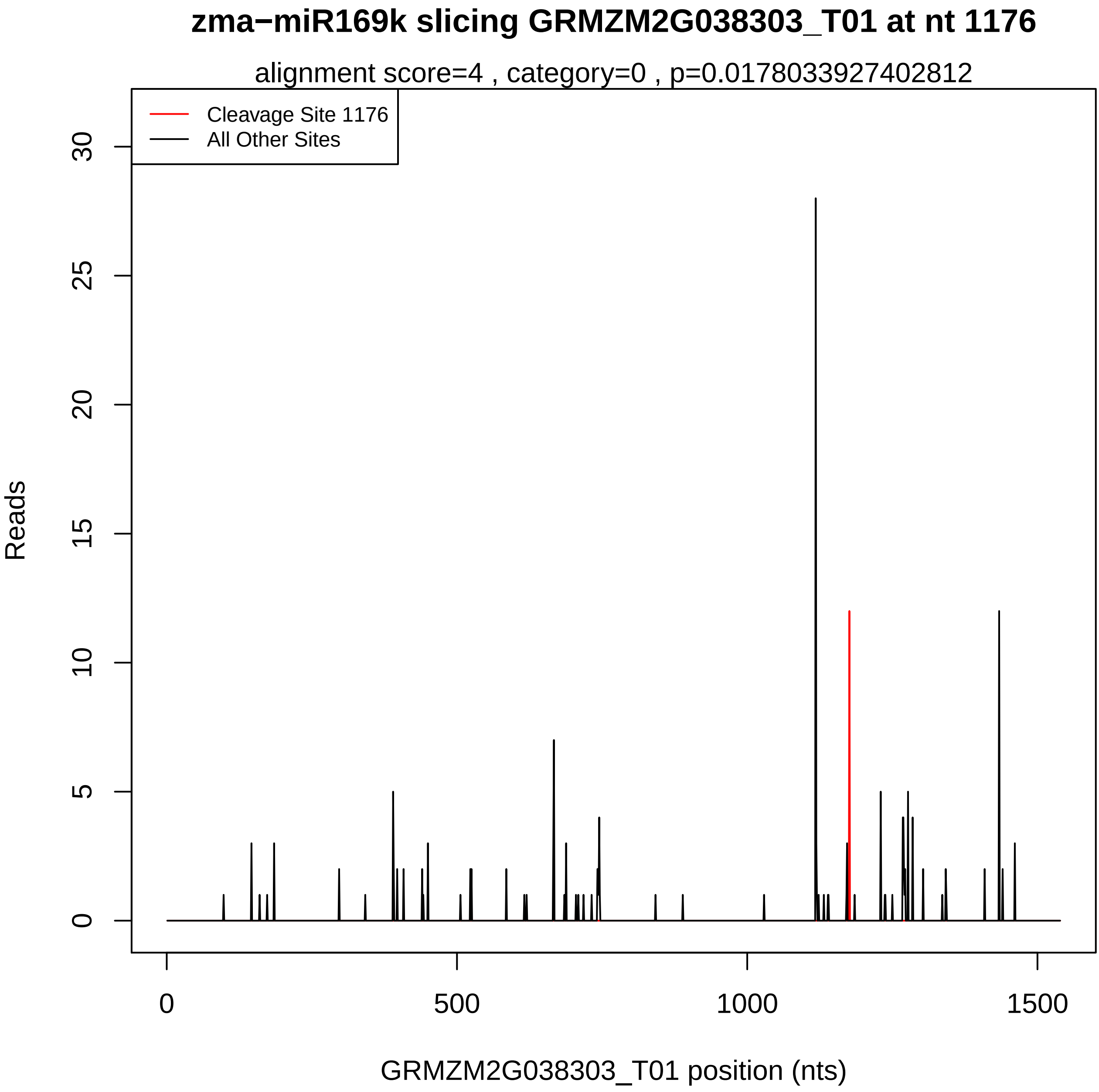

zma−miR169k slicing GRMZM2G038303_T01 at nt 1176
alignment score=4 , category=0 , p=0.0178033927402812
Cleavage Site 1176
All Other Sites
Reads
0
5
10
15
20
25
30
0
500
1000
1500
GRMZM2G038303_T01 position (nts)

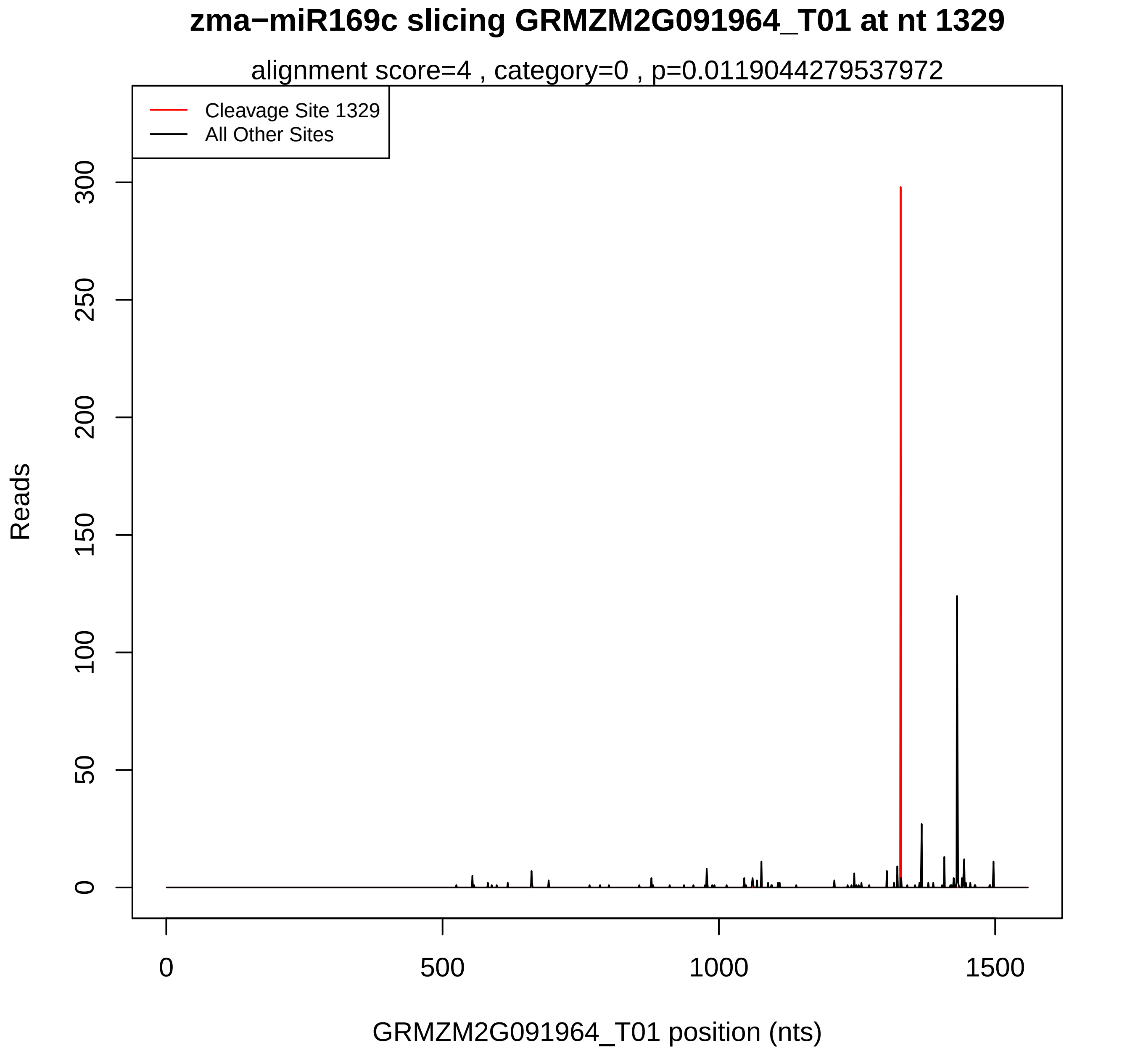
zma−miR169c slicing GRMZM2G091964_T01 at nt 1329
alignment score=4 , category=0 , p=0.0119044279537972
Cleavage Site 1329
All Other Sites
Reads
0
50
100
150
200
250
300
0
500
1000
1500
GRMZM2G091964_T01 position (nts)

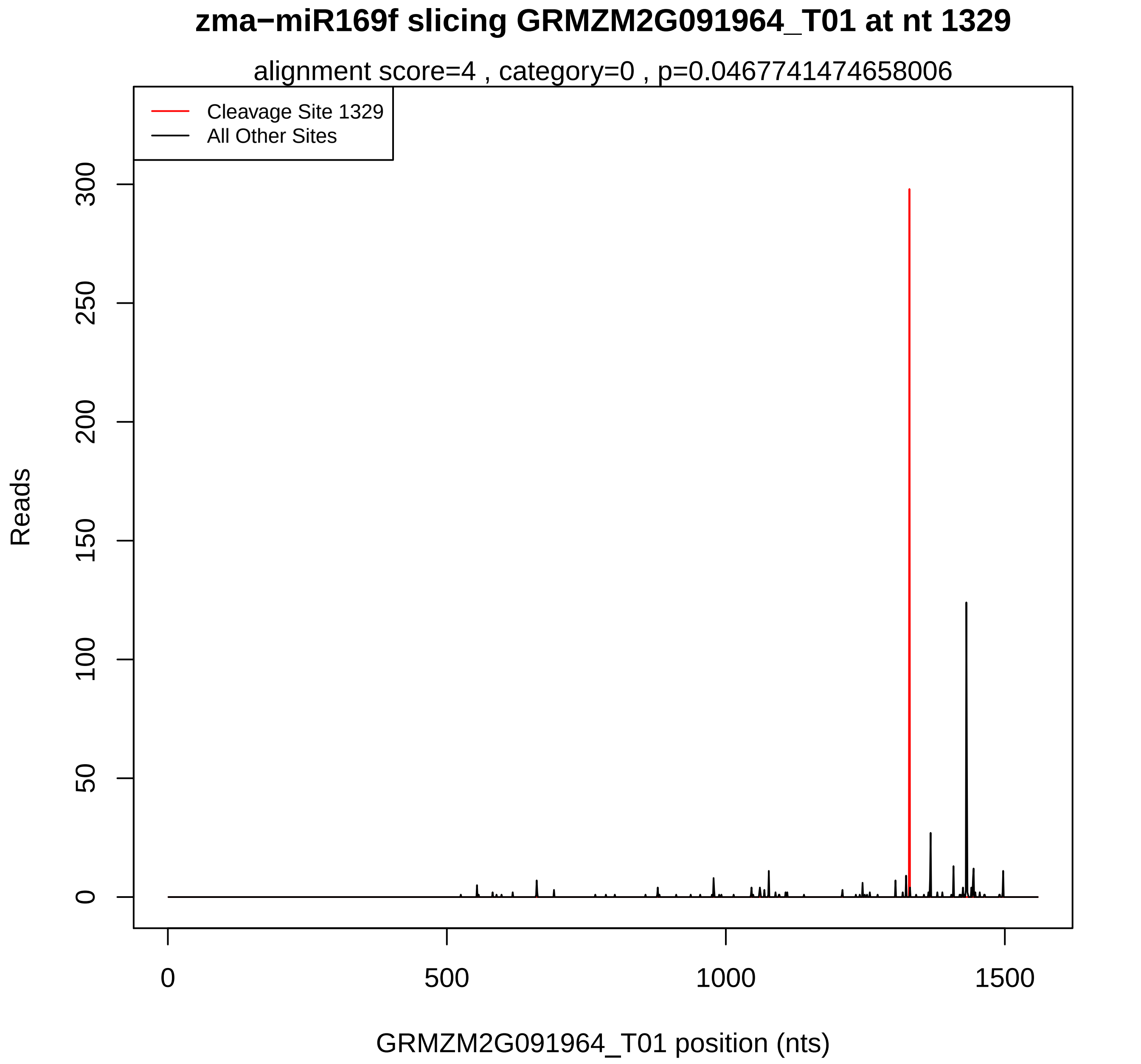

zma−miR169f slicing GRMZM2G091964_T01 at nt 1329
alignment score=4 , category=0 , p=0.0467741474658006
Cleavage Site 1329
All Other Sites
Reads
0
50
100
150
200
250
300
0
500
1000
1500
GRMZM2G091964_T01 position (nts)

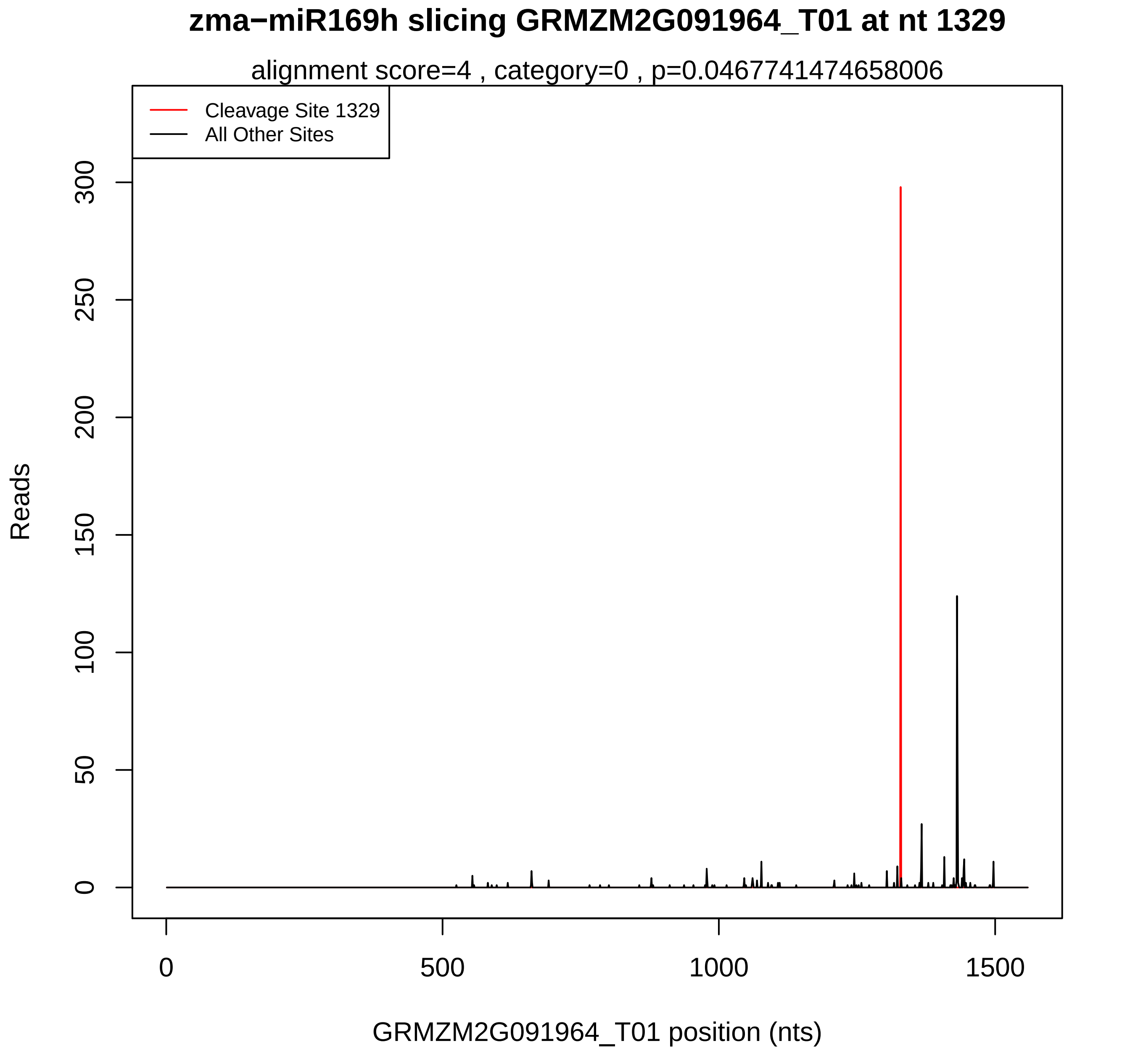
zma−miR169h slicing GRMZM2G091964_T01 at nt 1329
alignment score=4 , category=0 , p=0.0467741474658006
Cleavage Site 1329
All Other Sites
Reads
0
50
100
150
200
250
300
0
500
1000
1500
GRMZM2G091964_T01 position (nts)

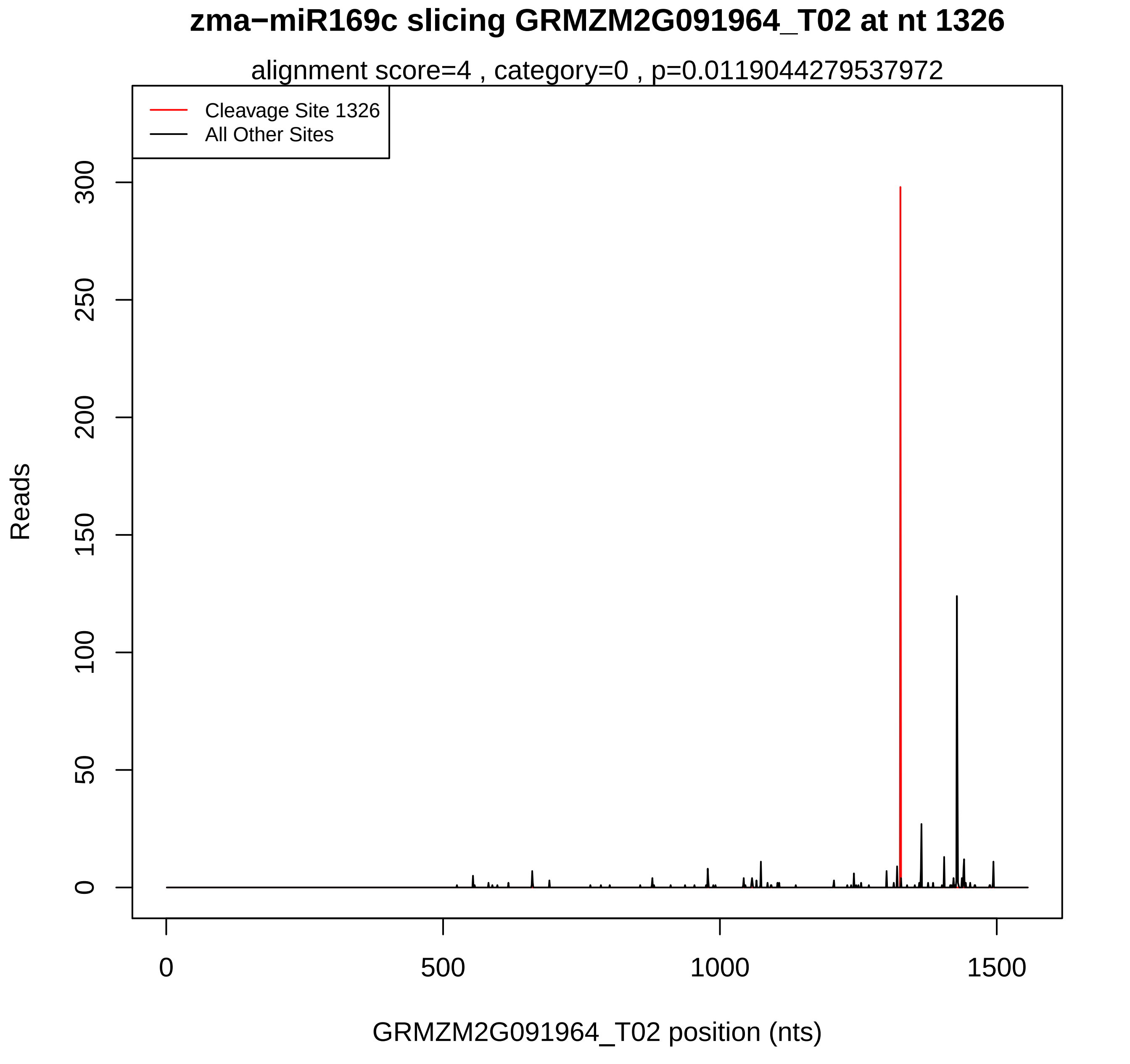
zma−miR169c slicing GRMZM2G091964_T02 at nt 1326
alignment score=4 , category=0 , p=0.0119044279537972
Cleavage Site 1326
All Other Sites
Reads
0
50
100
150
200
250
300
0
500
1000
1500
GRMZM2G091964_T02 position (nts)

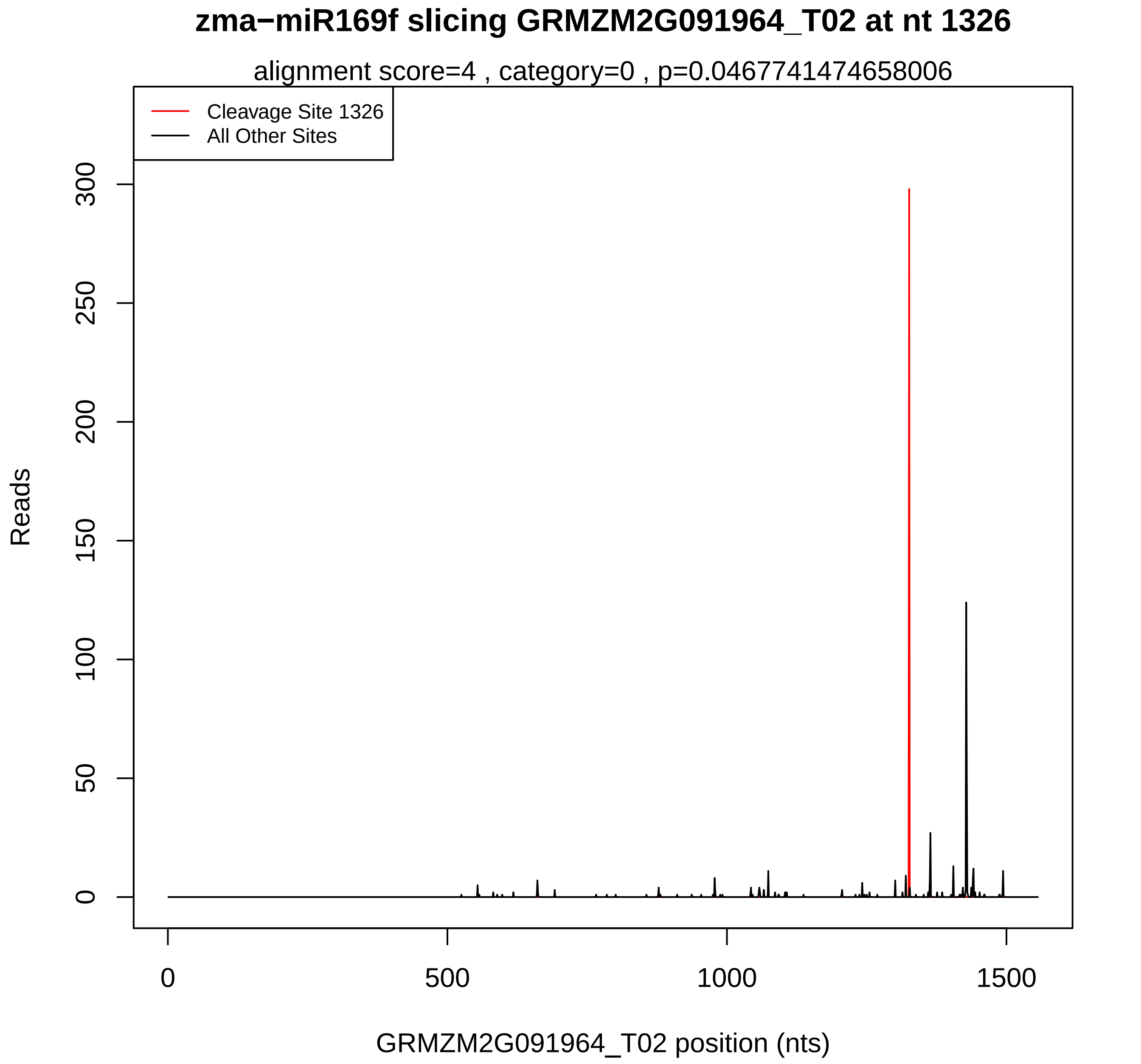

zma−miR169f slicing GRMZM2G091964_T02 at nt 1326
alignment score=4 , category=0 , p=0.0467741474658006
Cleavage Site 1326
All Other Sites
Reads
0
50
100
150
200
250
300
0
500
1000
1500
GRMZM2G091964_T02 position (nts)

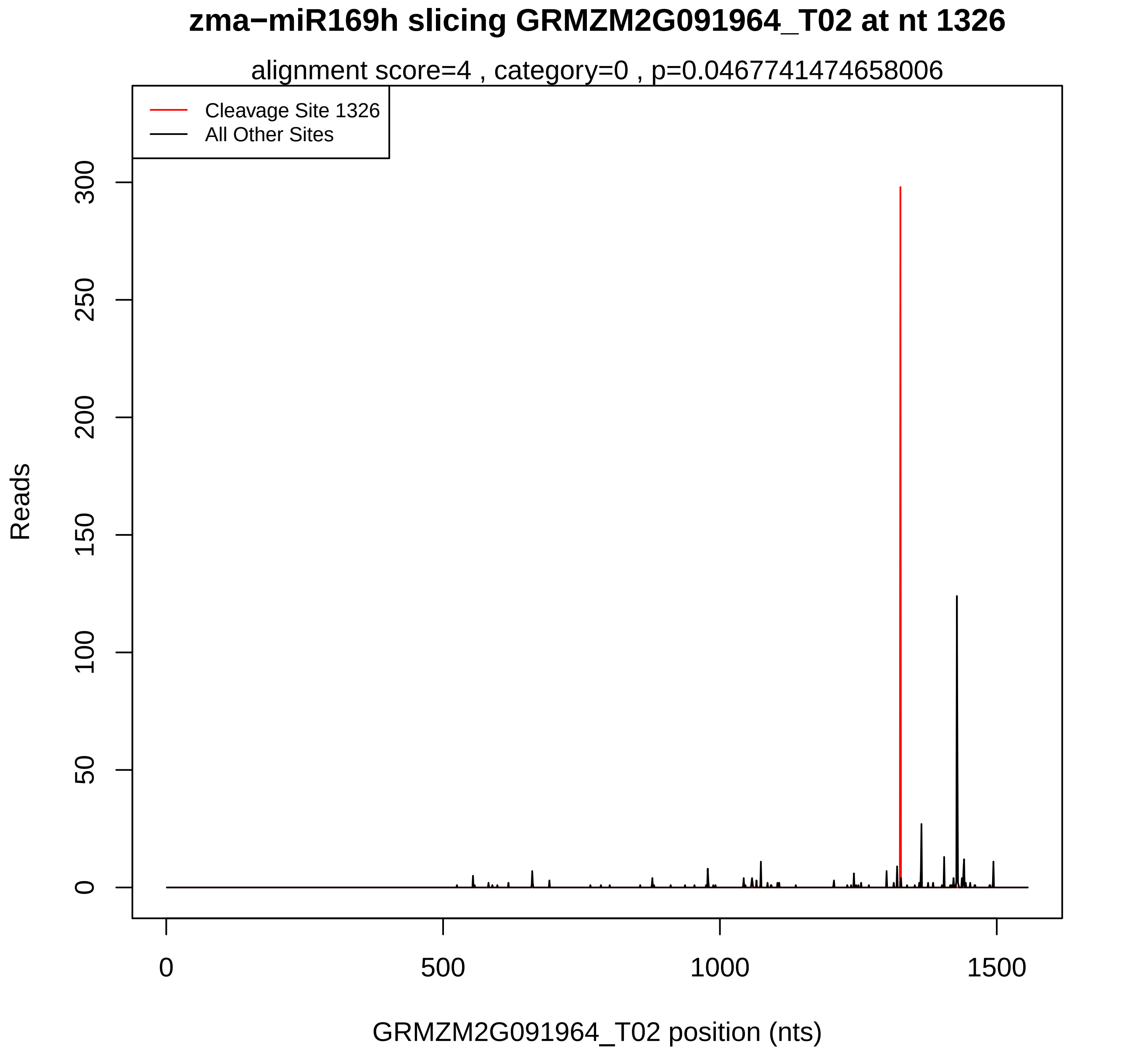

zma−miR169h slicing GRMZM2G091964_T02 at nt 1326
alignment score=4 , category=0 , p=0.0467741474658006
Cleavage Site 1326
All Other Sites
Reads
0
50
100
150
200
250
300
0
500
1000
1500
GRMZM2G091964_T02 position (nts)

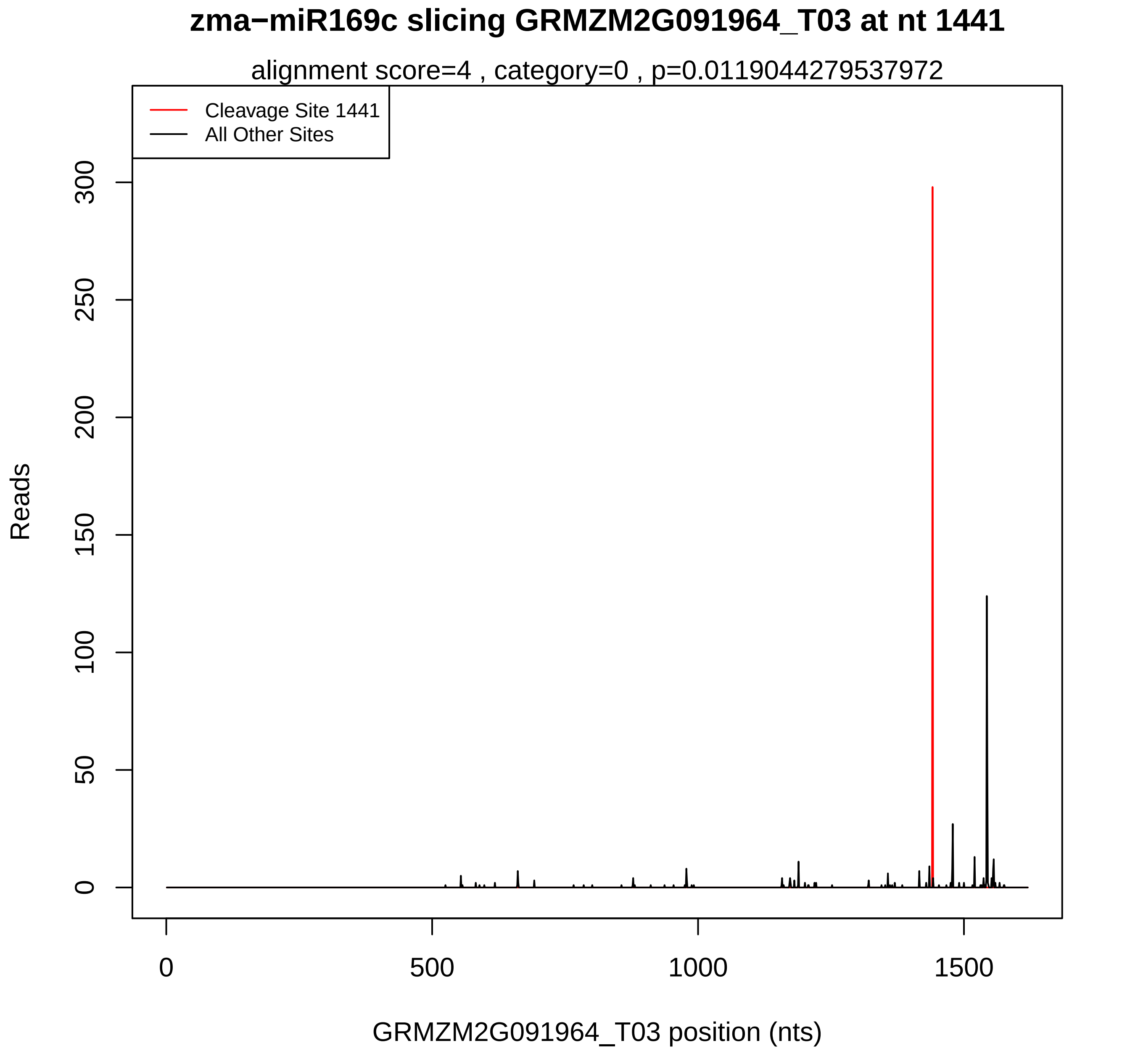
zma−miR169c slicing GRMZM2G091964_T03 at nt 1441
alignment score=4 , category=0 , p=0.0119044279537972
Cleavage Site 1441
All Other Sites
Reads
0
50
100
150
200
250
300
0
500
1000
1500
GRMZM2G091964_T03 position (nts)

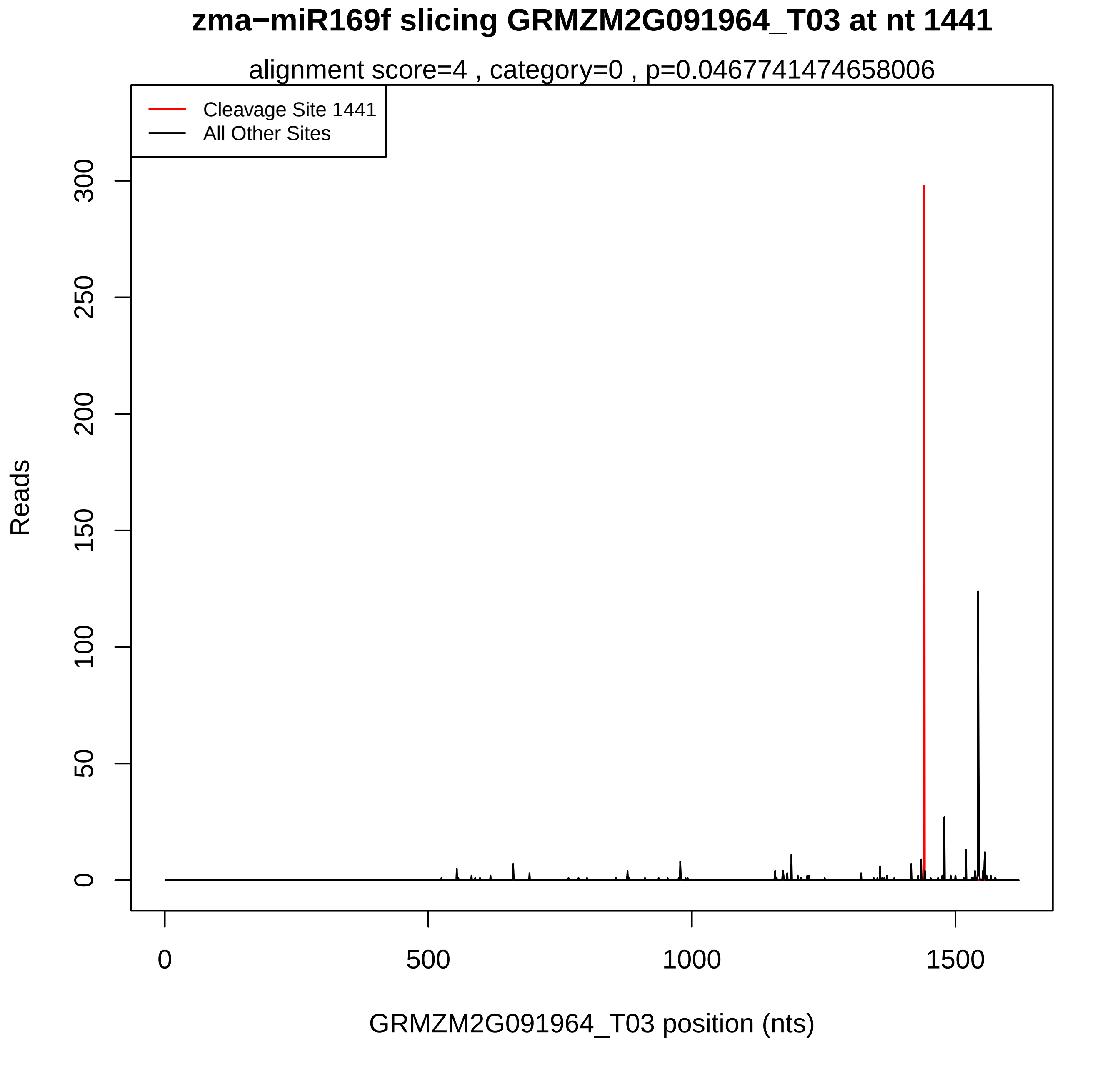

zma−miR169f slicing GRMZM2G091964_T03 at nt 1441
alignment score=4 , category=0 , p=0.0467741474658006
Cleavage Site 1441
All Other Sites
Reads
0
50
100
150
200
250
300
0
500
1000
1500
GRMZM2G091964_T03 position (nts)

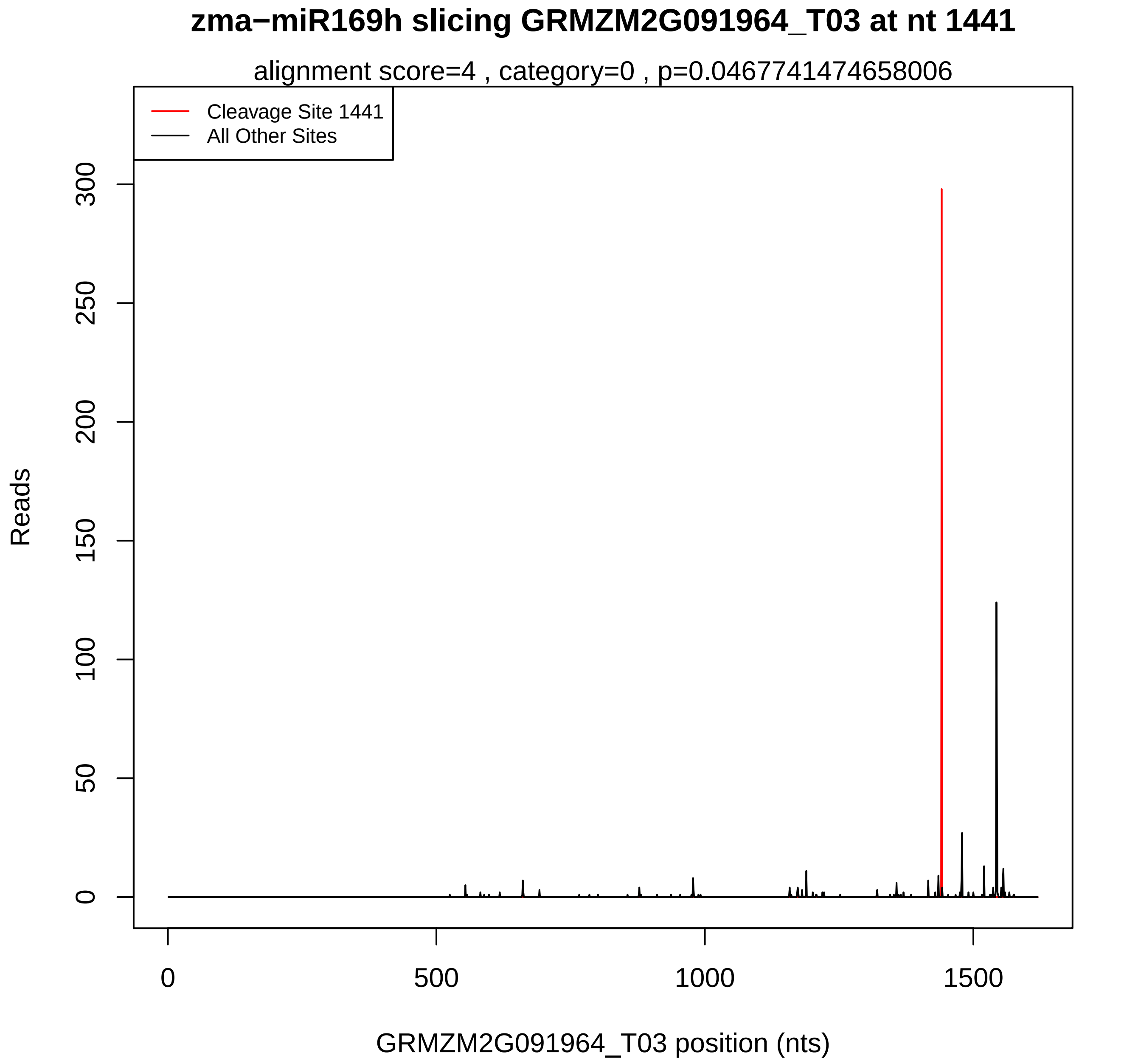
zma−miR169h slicing GRMZM2G091964_T03 at nt 1441
alignment score=4 , category=0 , p=0.0467741474658006
Cleavage Site 1441
All Other Sites
Reads
0
50
100
150
200
250
300
0
500
1000
1500
GRMZM2G091964_T03 position (nts)

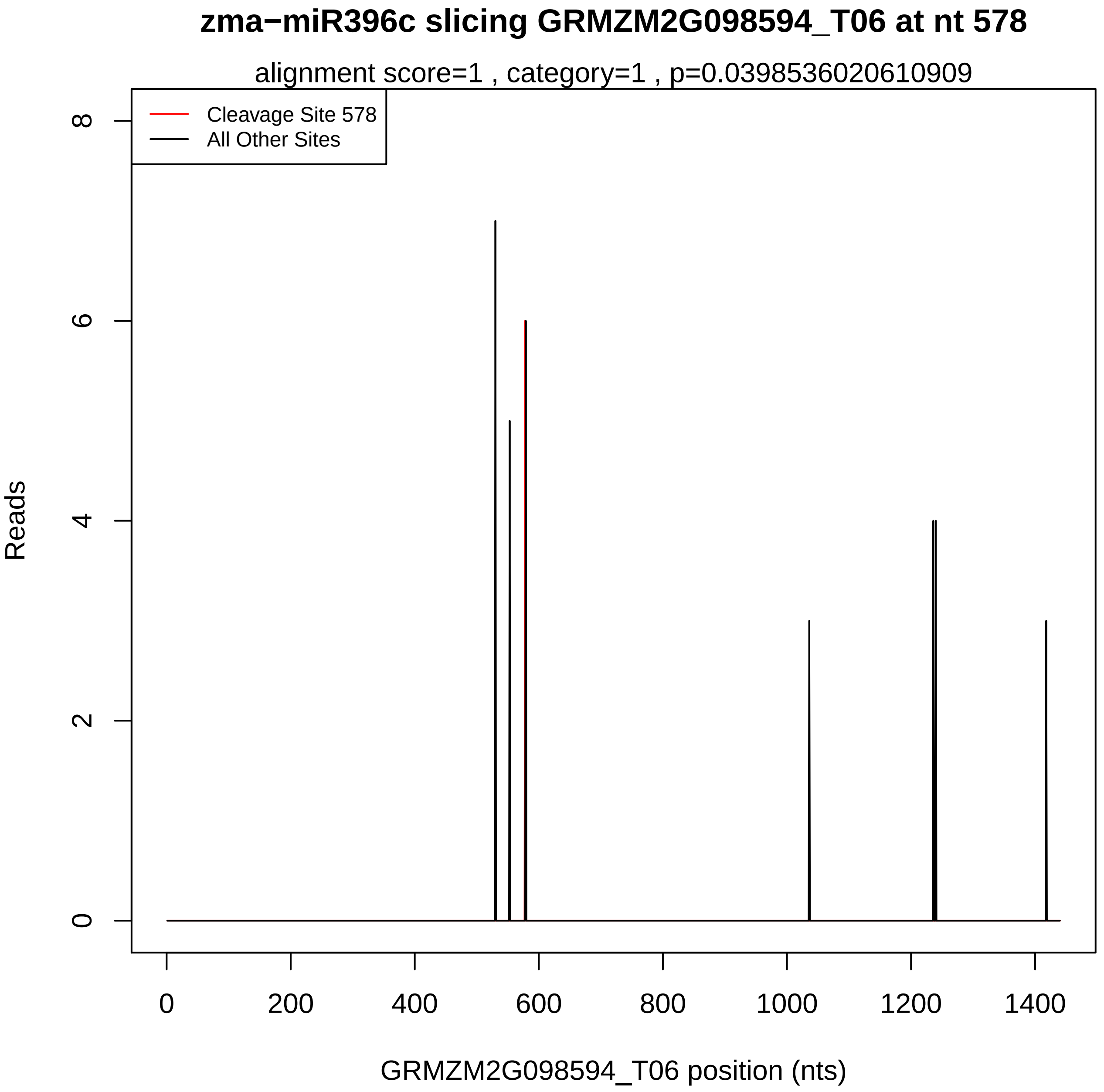
zma−miR396c slicing GRMZM2G098594_T06 at nt 578
alignment score=1 , category=1 , p=0.0398536020610909
Cleavage Site 578
All Other Sites
Reads
0
2
4
6
8
0
200
400
600
800
1000
1200
1400
GRMZM2G098594_T06 position (nts)

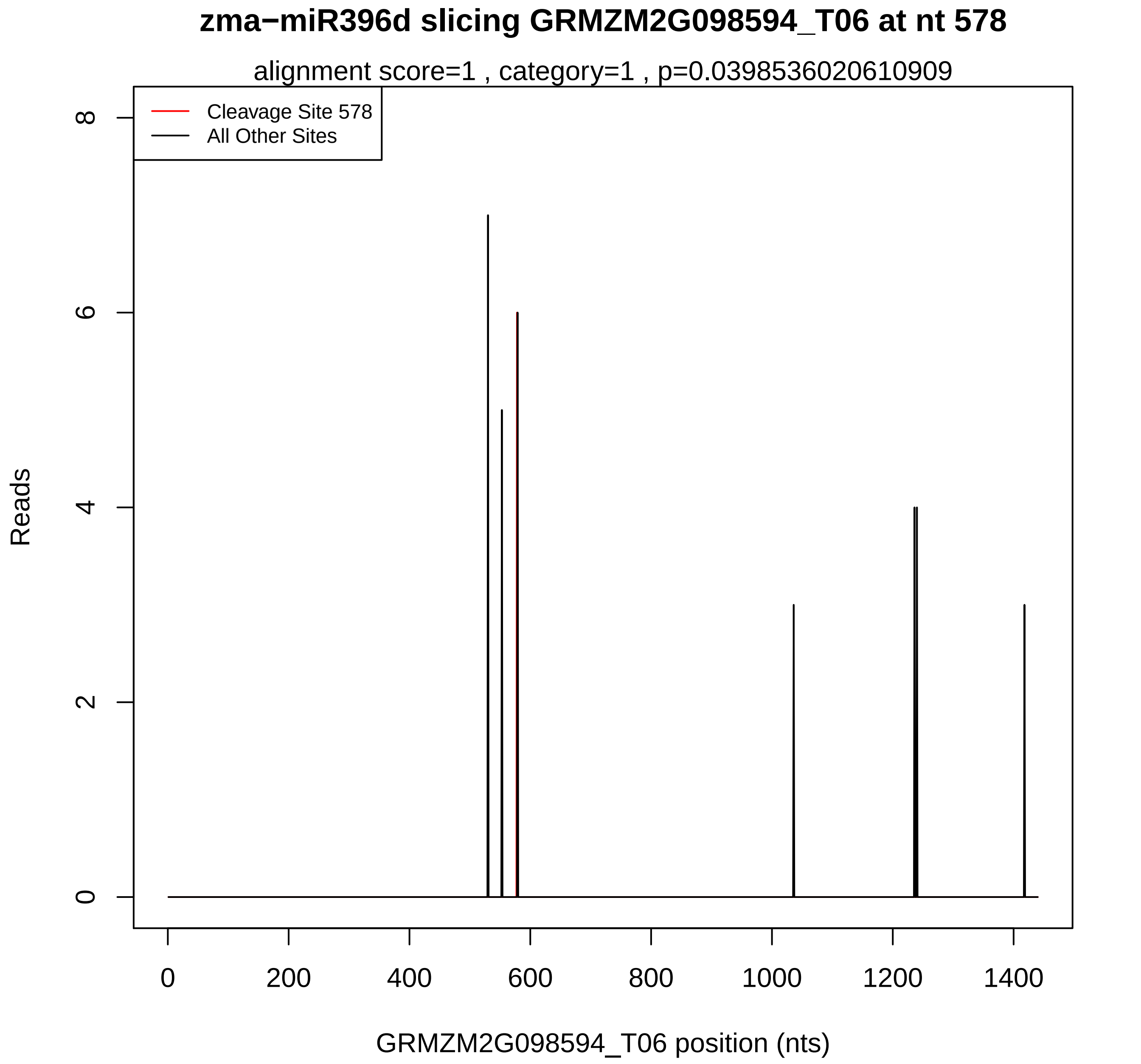
zma−miR396d slicing GRMZM2G098594_T06 at nt 578
alignment score=1 , category=1 , p=0.0398536020610909
Cleavage Site 578
All Other Sites
Reads
0
2
4
6
8
0
200
400
600
800
1000
1200
1400
GRMZM2G098594_T06 position (nts)

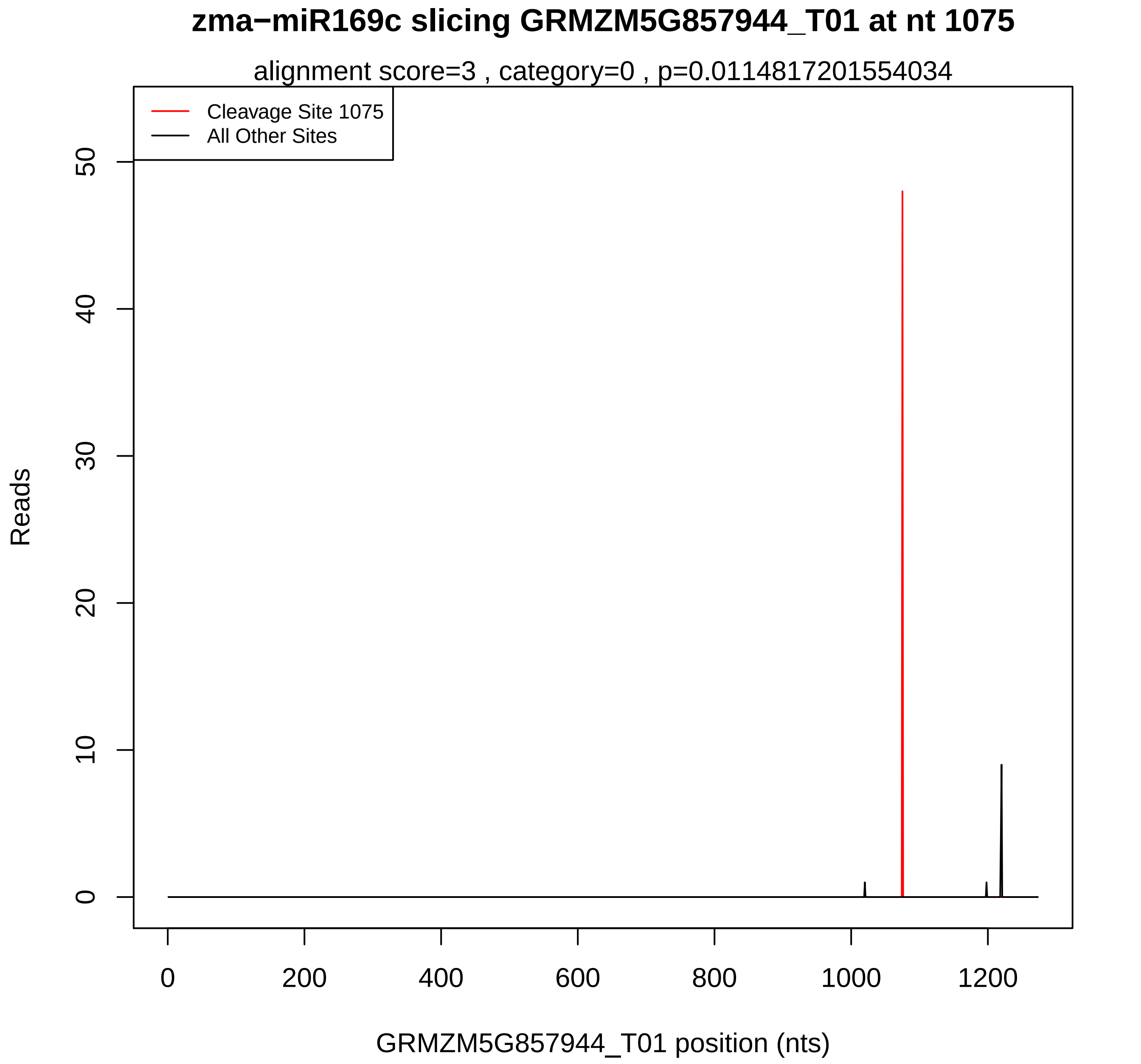

zma−miR169c slicing GRMZM5G857944_T01 at nt 1075
alignment score=3 , category=0 , p=0.0114817201554034
Cleavage Site 1075
All Other Sites
Reads
0
10
20
30
40
50
0
200
400
600
800
1000
1200
GRMZM5G857944_T01 position (nts)

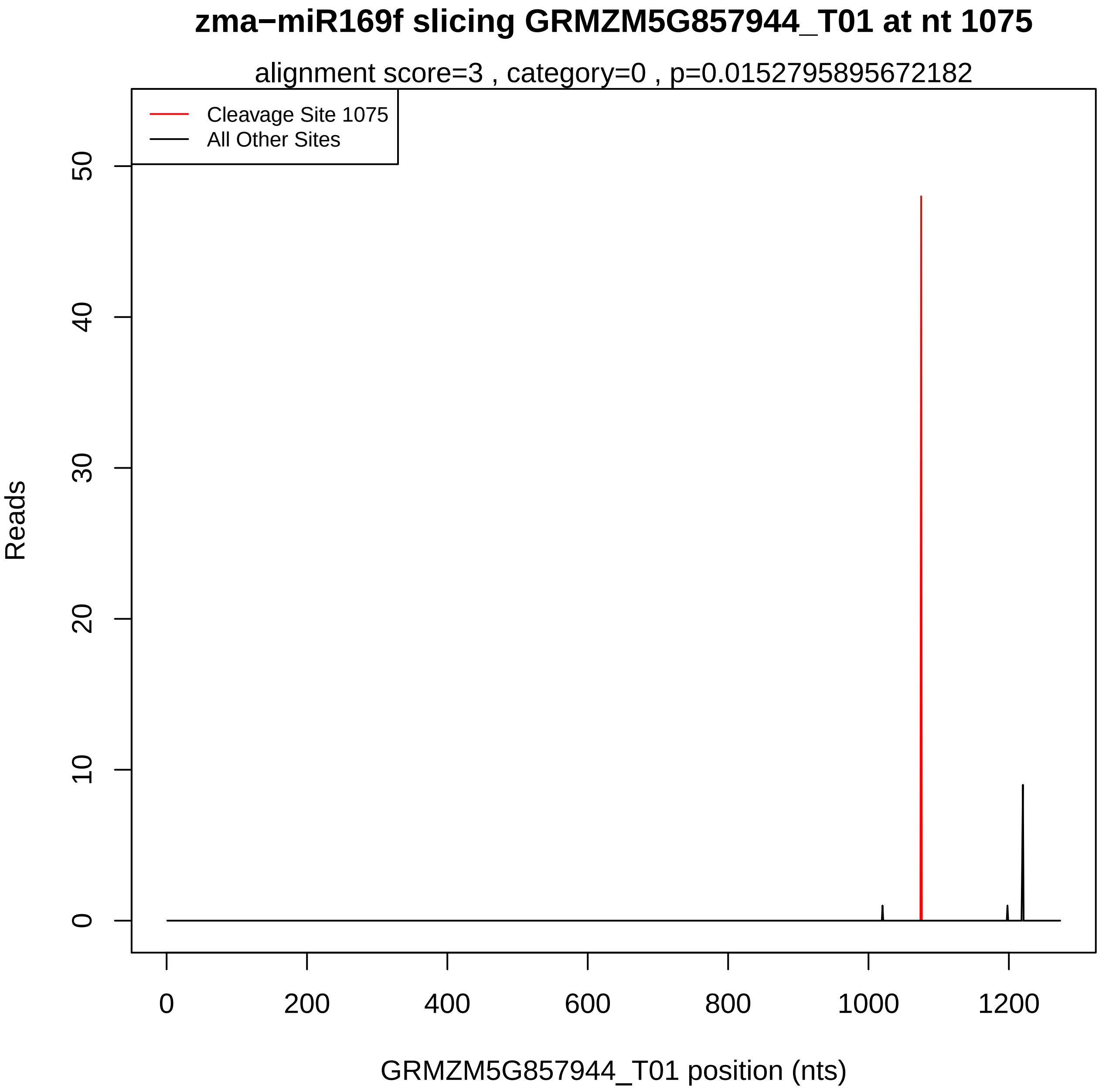

zma−miR169f slicing GRMZM5G857944_T01 at nt 1075
alignment score=3 , category=0 , p=0.0152795895672182
Cleavage Site 1075
All Other Sites
Reads
0
10
20
30
40
50
0
200
400
600
800
1000
1200
GRMZM5G857944_T01 position (nts)

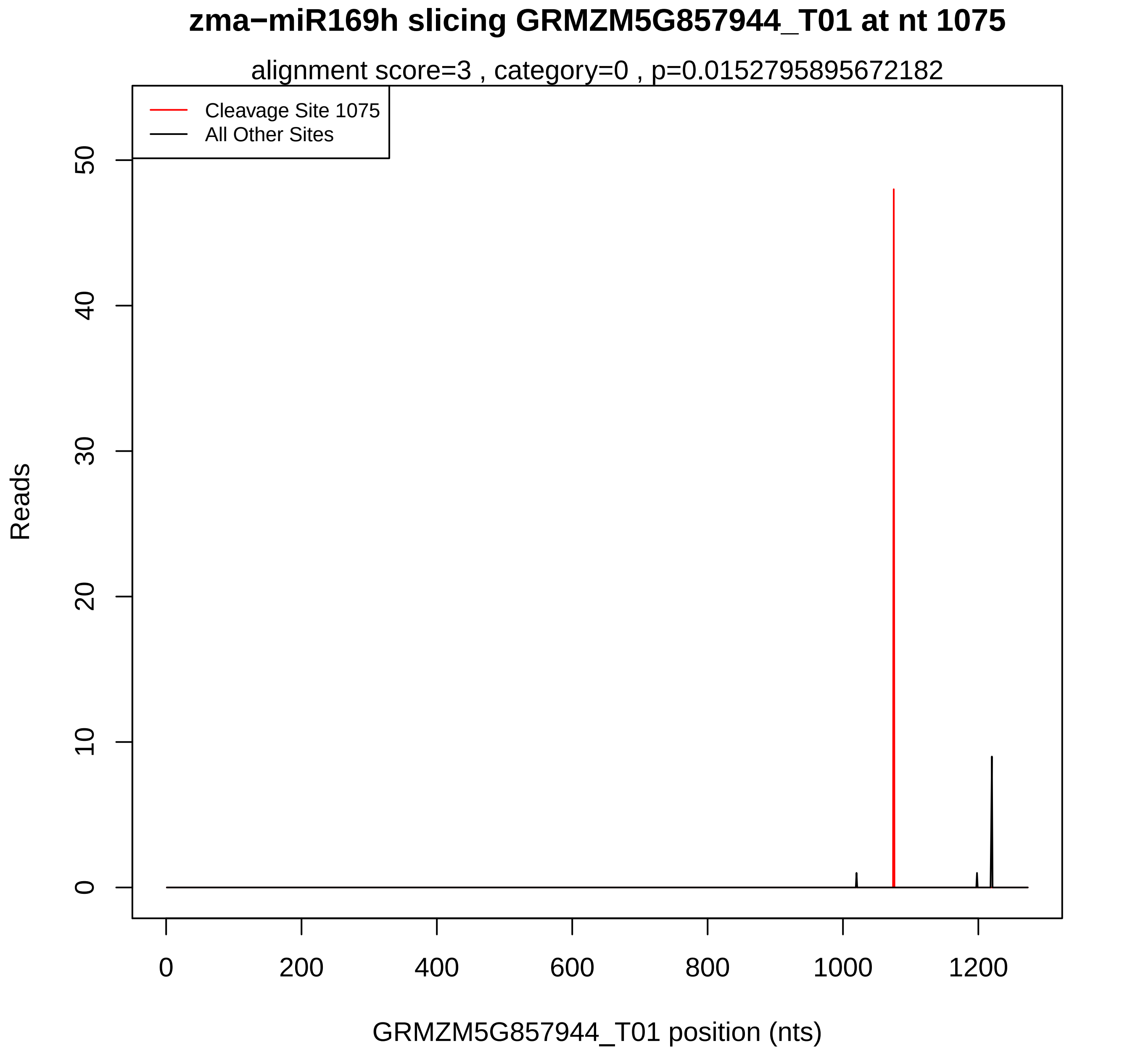
zma−miR169h slicing GRMZM5G857944_T01 at nt 1075
alignment score=3 , category=0 , p=0.0152795895672182
Cleavage Site 1075
All Other Sites
Reads
0
10
20
30
40
50
0
200
400
600
800
1000
1200
GRMZM5G857944_T01 position (nts)

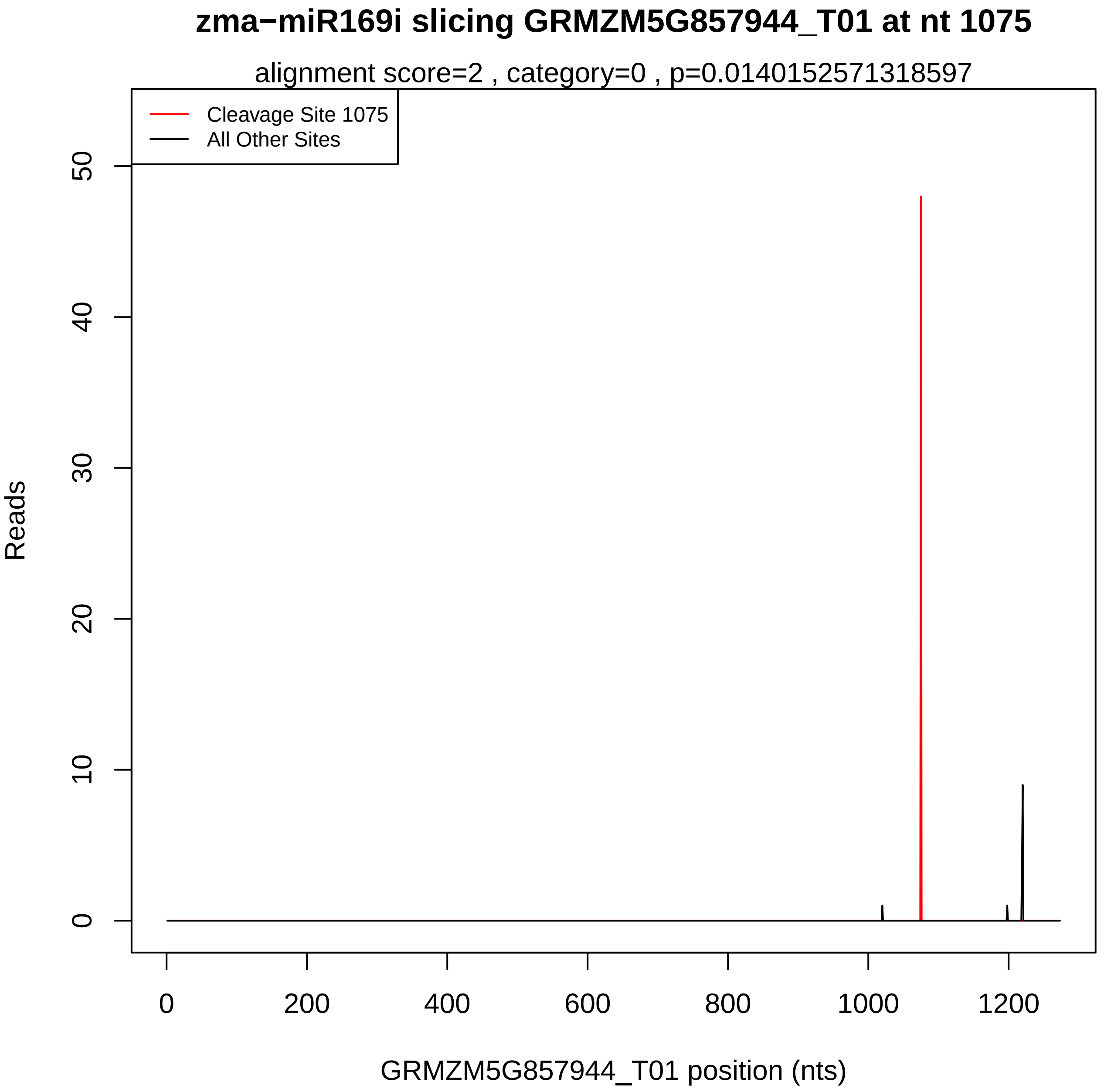

zma−miR169i slicing GRMZM5G857944_T01 at nt 1075
alignment score=2 , category=0 , p=0.0140152571318597
Cleavage Site 1075
All Other Sites
Reads
0
10
20
30
40
50
0
200
400
600
800
1000
1200
GRMZM5G857944_T01 position (nts)

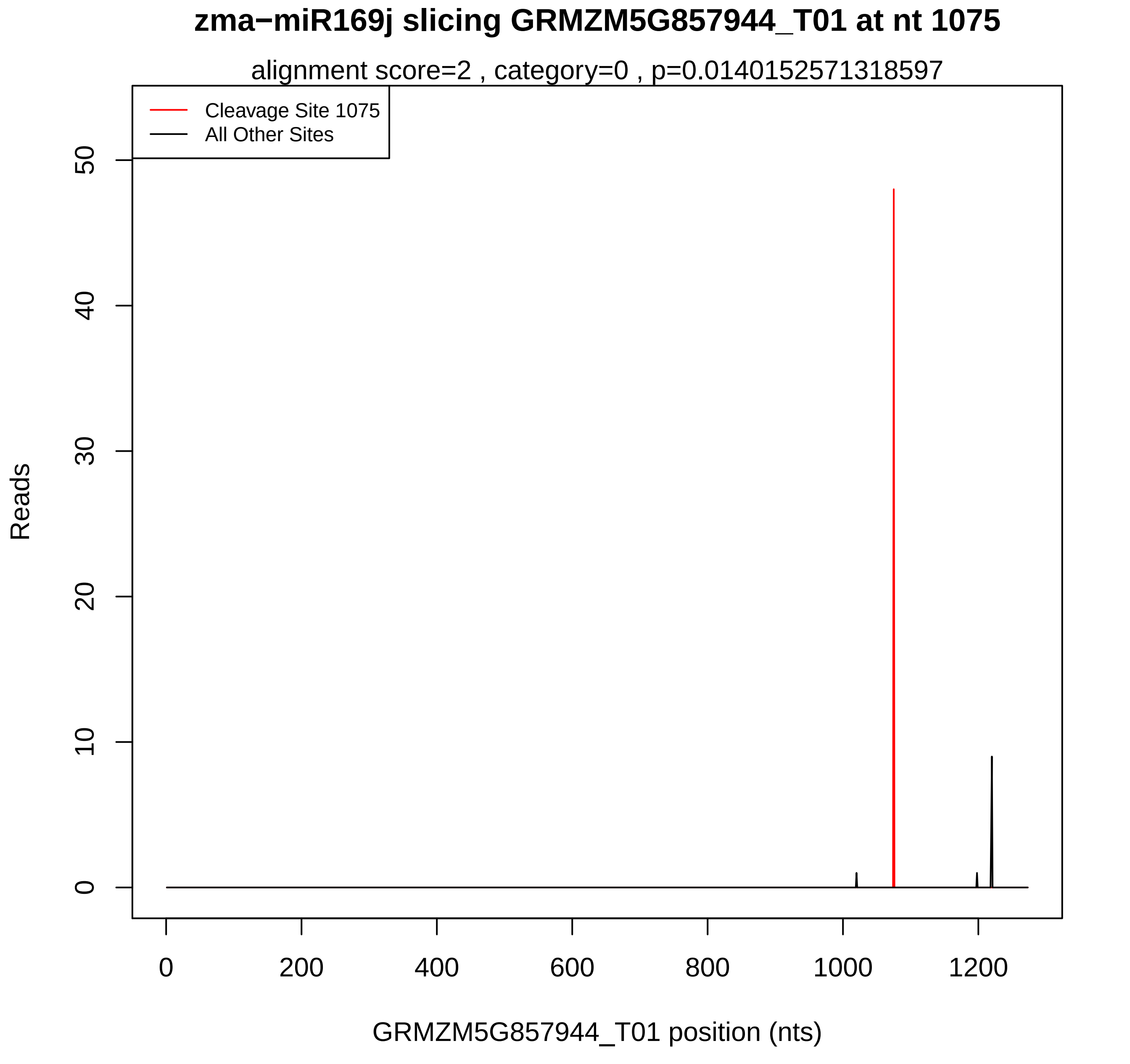

zma−miR169j slicing GRMZM5G857944_T01 at nt 1075
alignment score=2 , category=0 , p=0.0140152571318597
Cleavage Site 1075
All Other Sites
Reads
0
10
20
30
40
50
0
200
400
600
800
1000
1200
GRMZM5G857944_T01 position (nts)

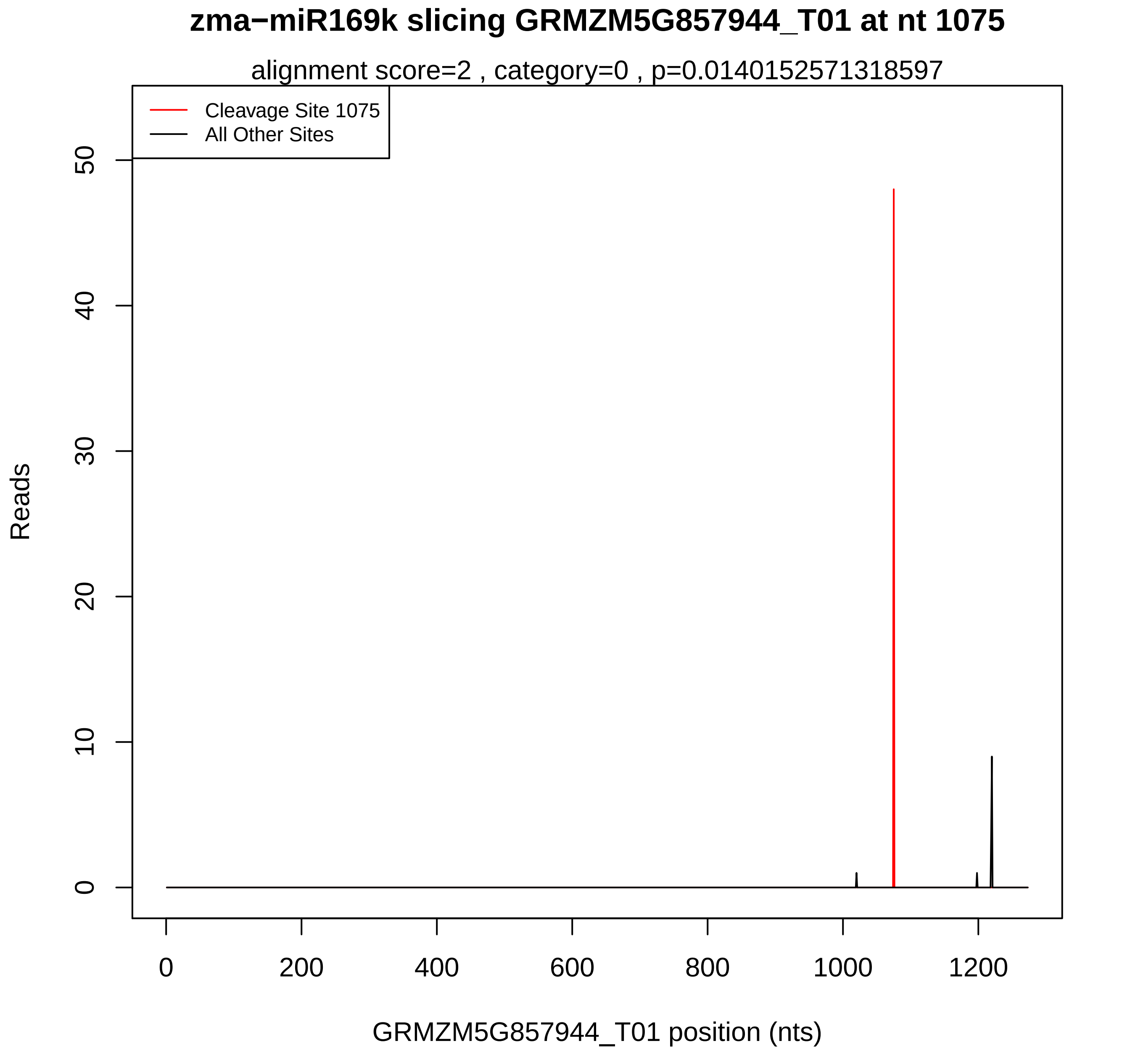
zma−miR169k slicing GRMZM5G857944_T01 at nt 1075
alignment score=2 , category=0 , p=0.0140152571318597
Cleavage Site 1075
All Other Sites
Reads
0
10
20
30
40
50
0
200
400
600
800
1000
1200
GRMZM5G857944_T01 position (nts)

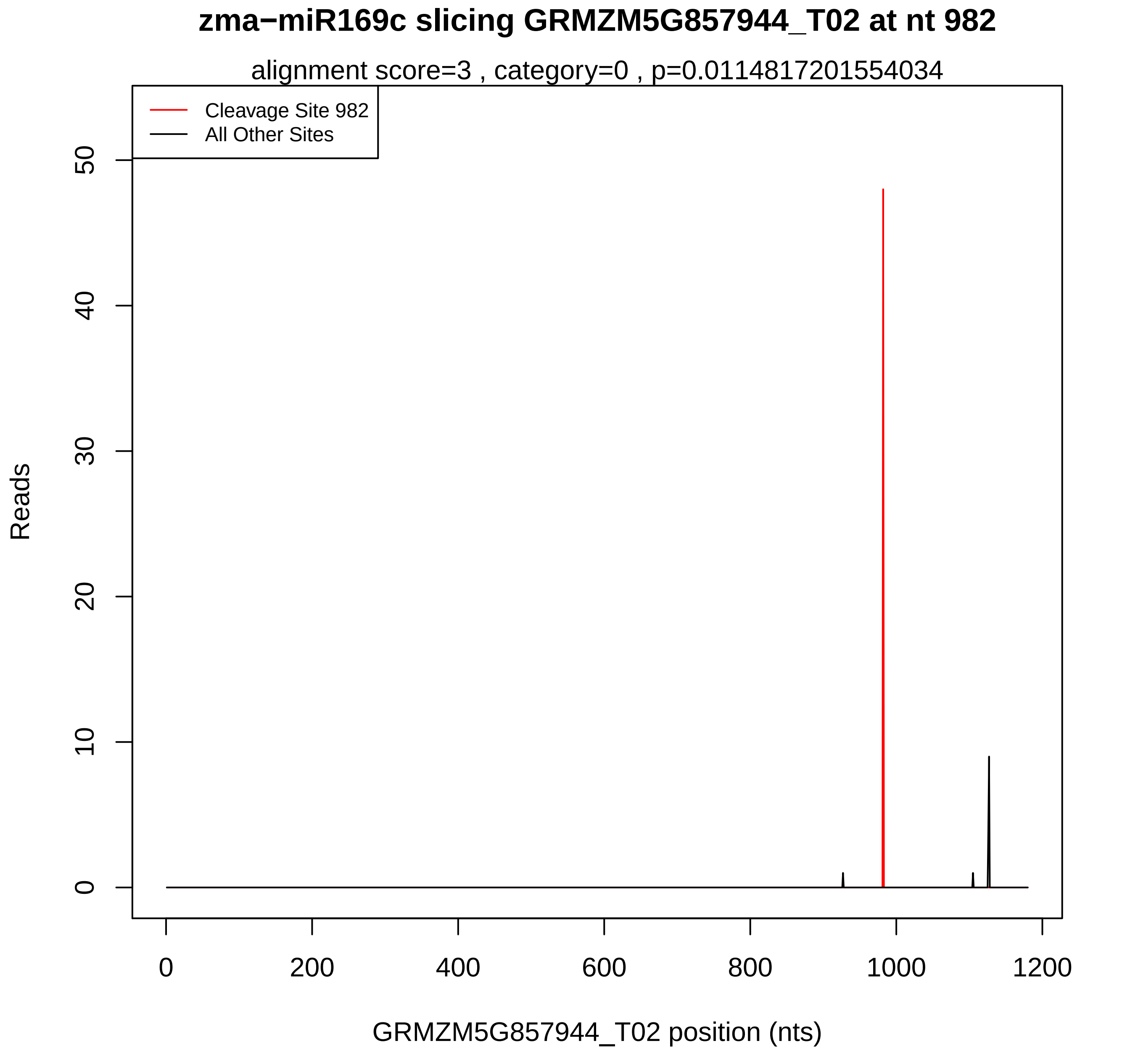
zma−miR169c slicing GRMZM5G857944_T02 at nt 982
alignment score=3 , category=0 , p=0.0114817201554034
Cleavage Site 982
All Other Sites
Reads
0
10
20
30
40
50
0
200
400
600
800
1000
1200
GRMZM5G857944_T02 position (nts)

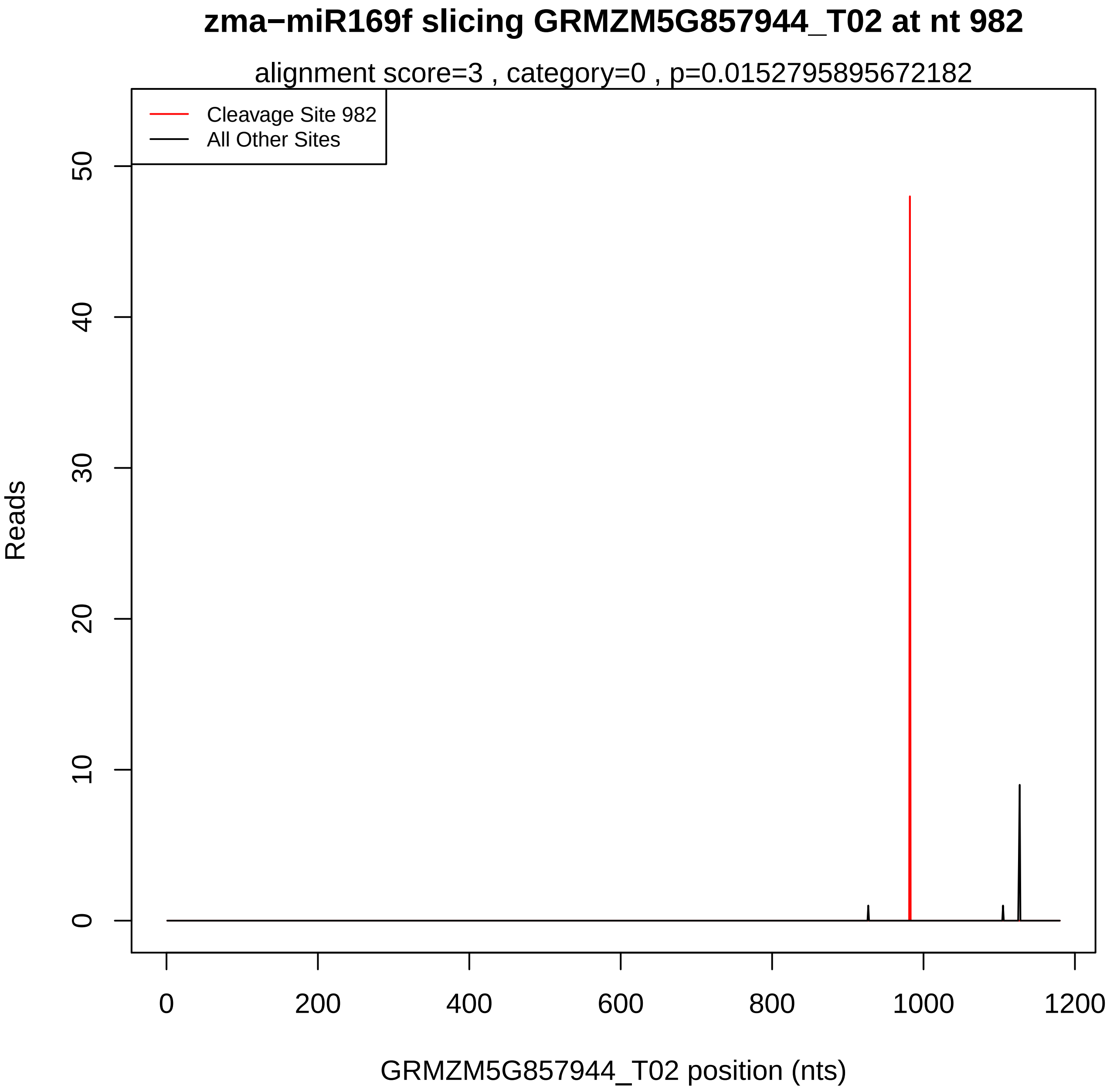
zma−miR169f slicing GRMZM5G857944_T02 at nt 982
alignment score=3 , category=0 , p=0.0152795895672182
Cleavage Site 982
All Other Sites
Reads
0
10
20
30
40
50
0
200
400
600
800
1000
1200
GRMZM5G857944_T02 position (nts)

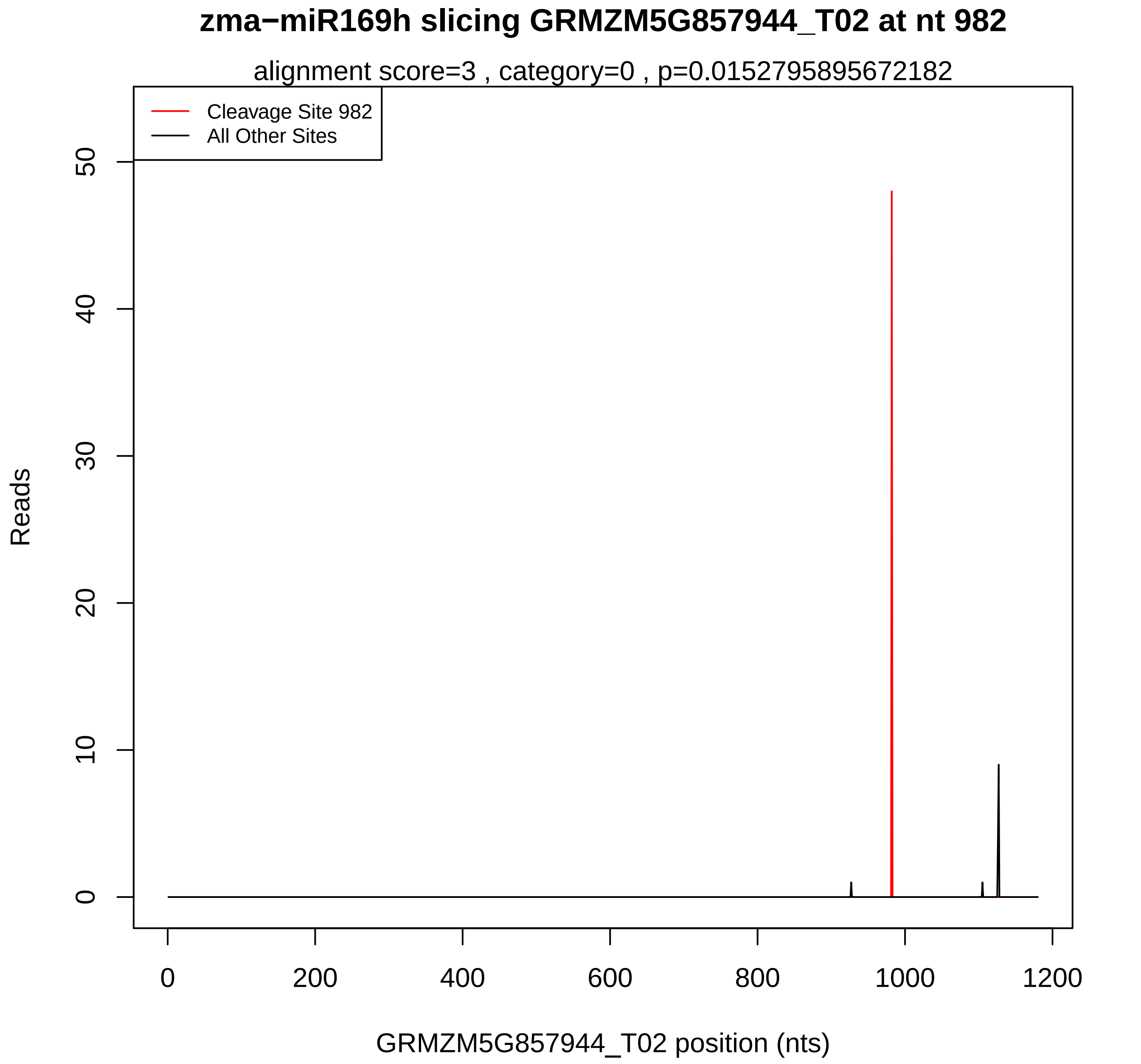
zma−miR169h slicing GRMZM5G857944_T02 at nt 982
alignment score=3 , category=0 , p=0.0152795895672182
Cleavage Site 982
All Other Sites
Reads
0
10
20
30
40
50
0
200
400
600
800
1000
1200
GRMZM5G857944_T02 position (nts)

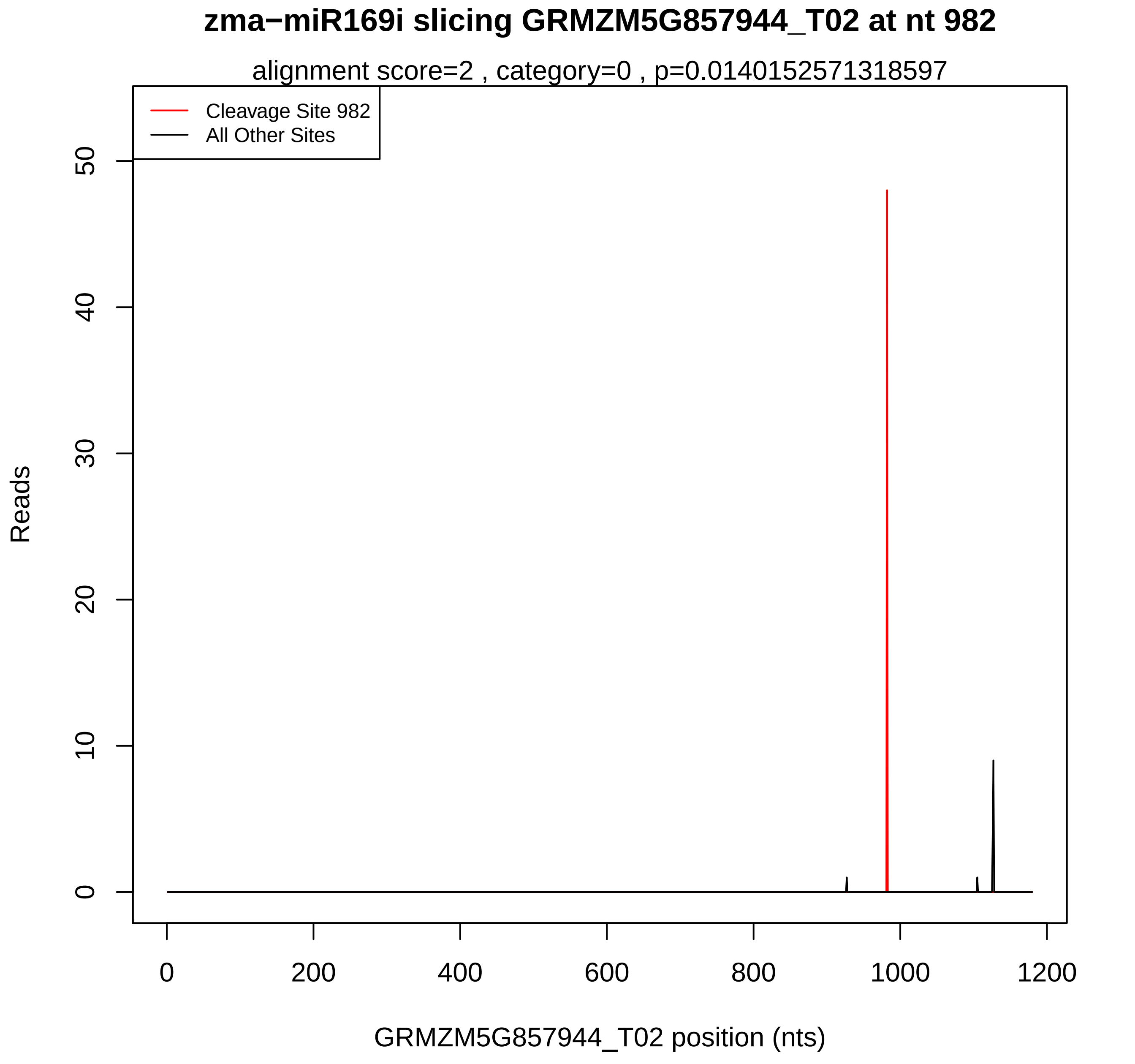

zma−miR169i slicing GRMZM5G857944_T02 at nt 982
alignment score=2 , category=0 , p=0.0140152571318597
Cleavage Site 982
All Other Sites
Reads
0
10
20
30
40
50
0
200
400
600
800
1000
1200
GRMZM5G857944_T02 position (nts)

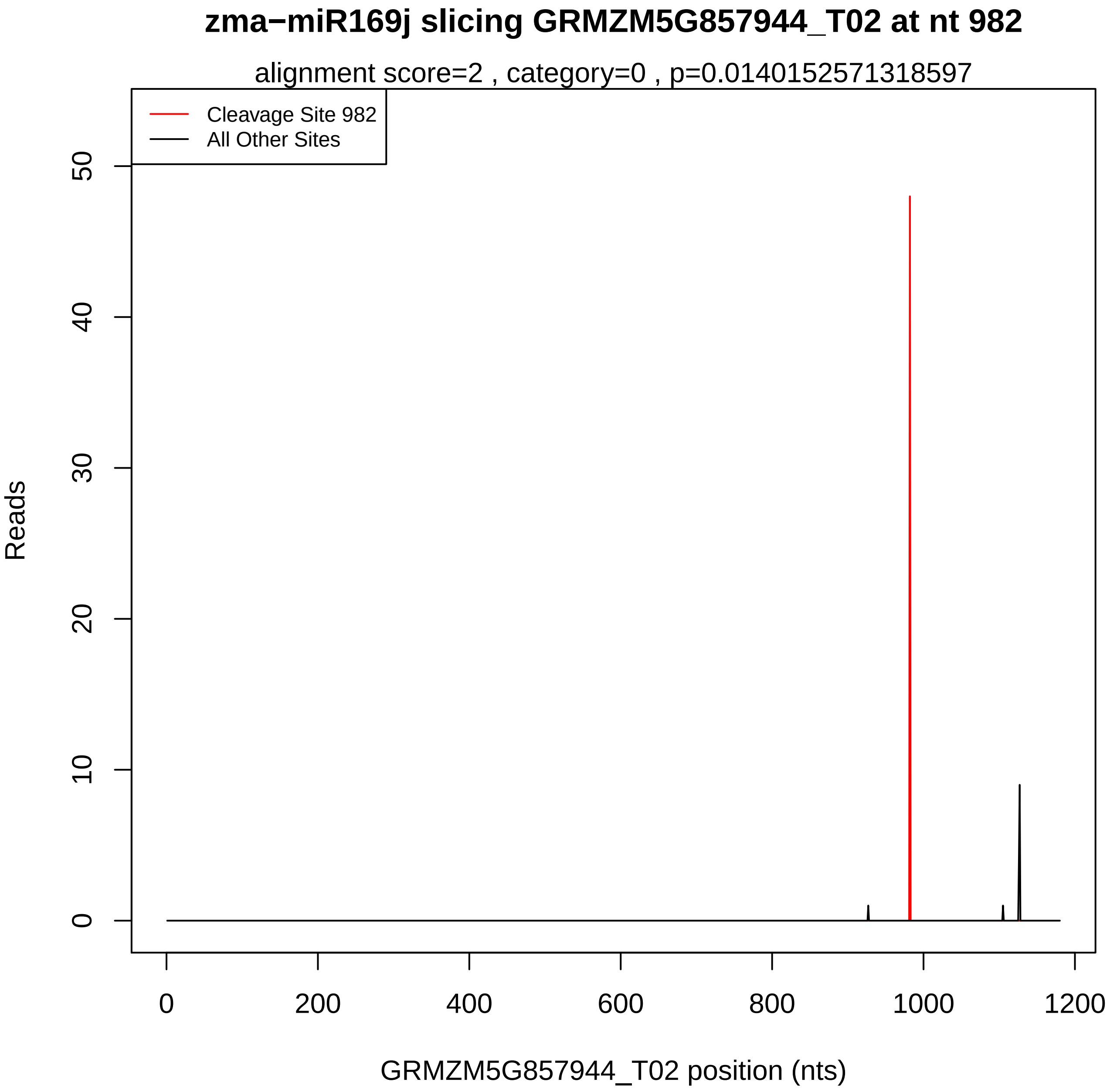

zma−miR169j slicing GRMZM5G857944_T02 at nt 982
alignment score=2 , category=0 , p=0.0140152571318597
Cleavage Site 982
All Other Sites
Reads
0
10
20
30
40
50
0
200
400
600
800
1000
1200
GRMZM5G857944_T02 position (nts)

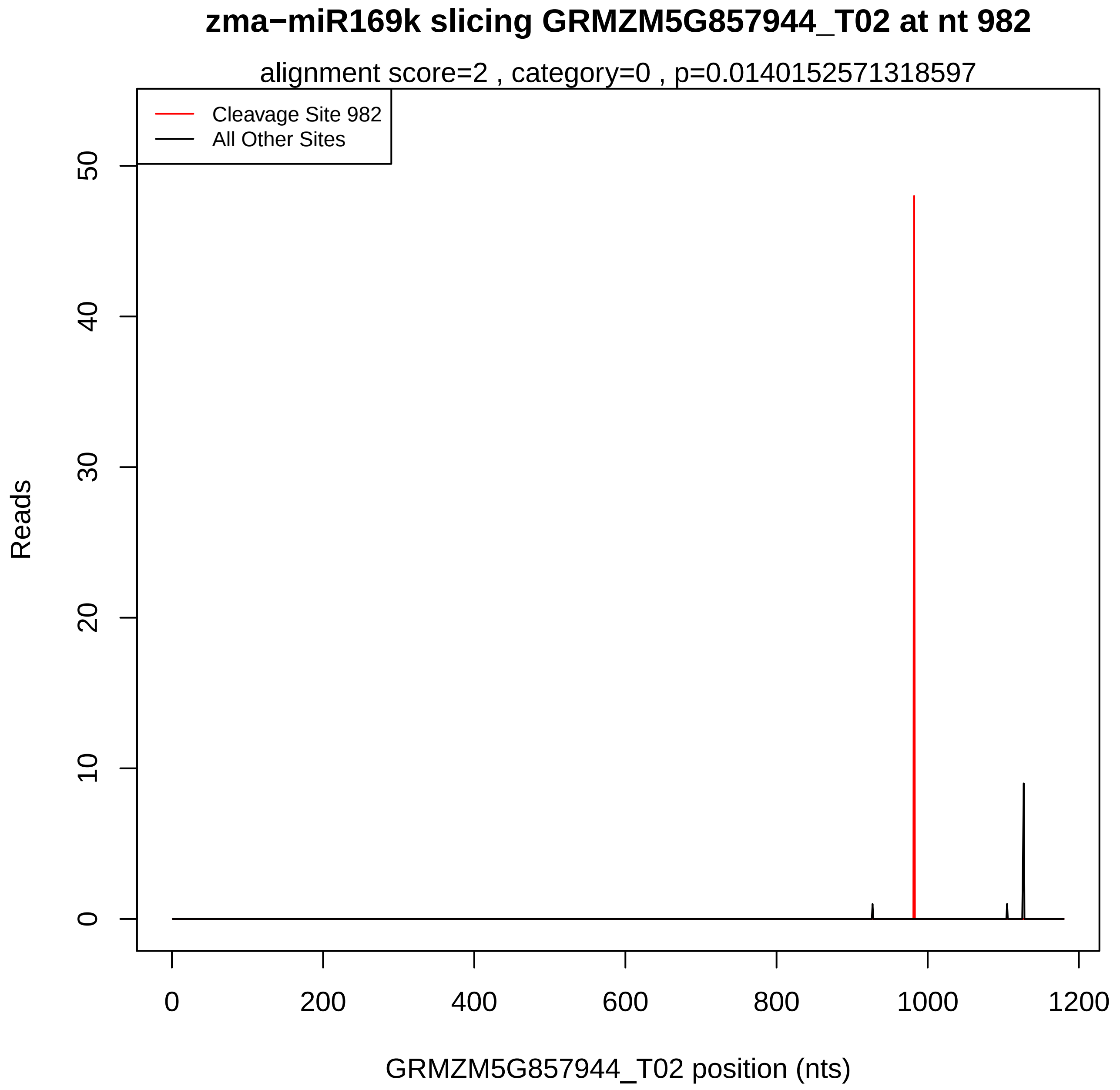

zma−miR169k slicing GRMZM5G857944_T02 at nt 982
alignment score=2 , category=0 , p=0.0140152571318597
Cleavage Site 982
All Other Sites
Reads
0
10
20
30
40
50
0
200
400
600
800
1000
1200
GRMZM5G857944_T02 position (nts)

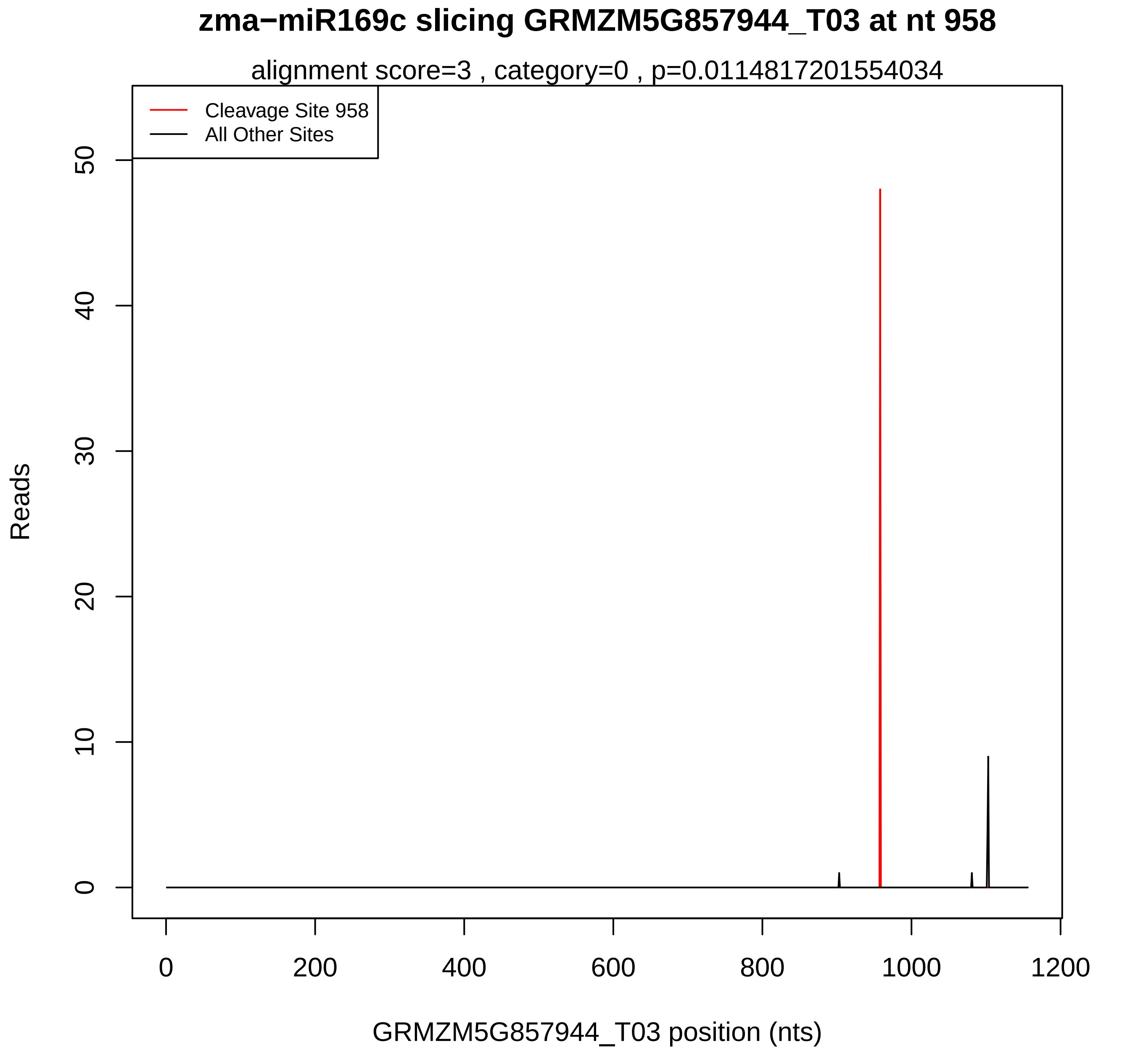
zma−miR169c slicing GRMZM5G857944_T03 at nt 958
alignment score=3 , category=0 , p=0.0114817201554034
Cleavage Site 958
All Other Sites
Reads
0
10
20
30
40
50
0
200
400
600
800
1000
1200
GRMZM5G857944_T03 position (nts)

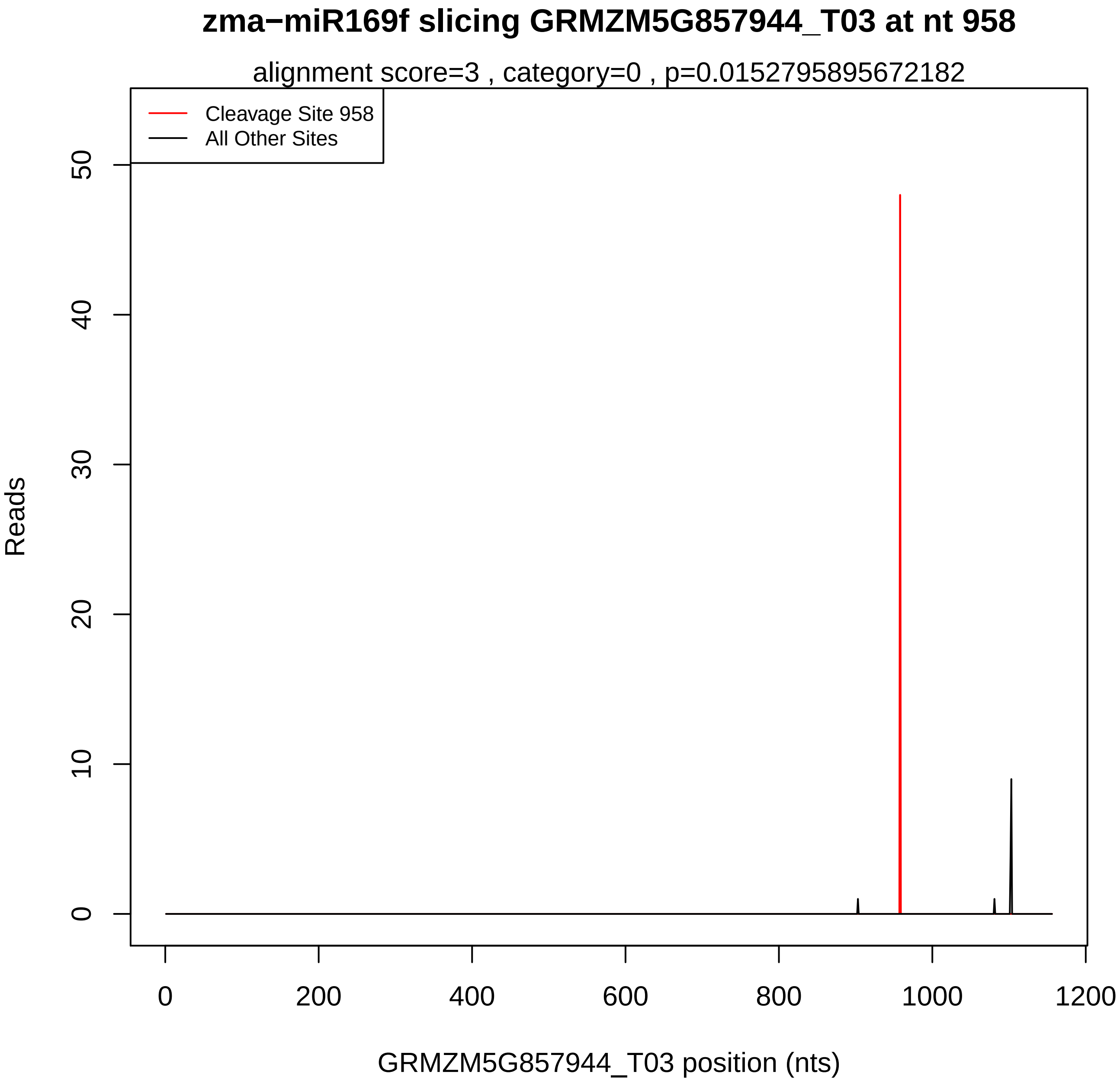

zma−miR169f slicing GRMZM5G857944_T03 at nt 958
alignment score=3 , category=0 , p=0.0152795895672182
Cleavage Site 958
All Other Sites
Reads
0
10
20
30
40
50
0
200
400
600
800
1000
1200
GRMZM5G857944_T03 position (nts)

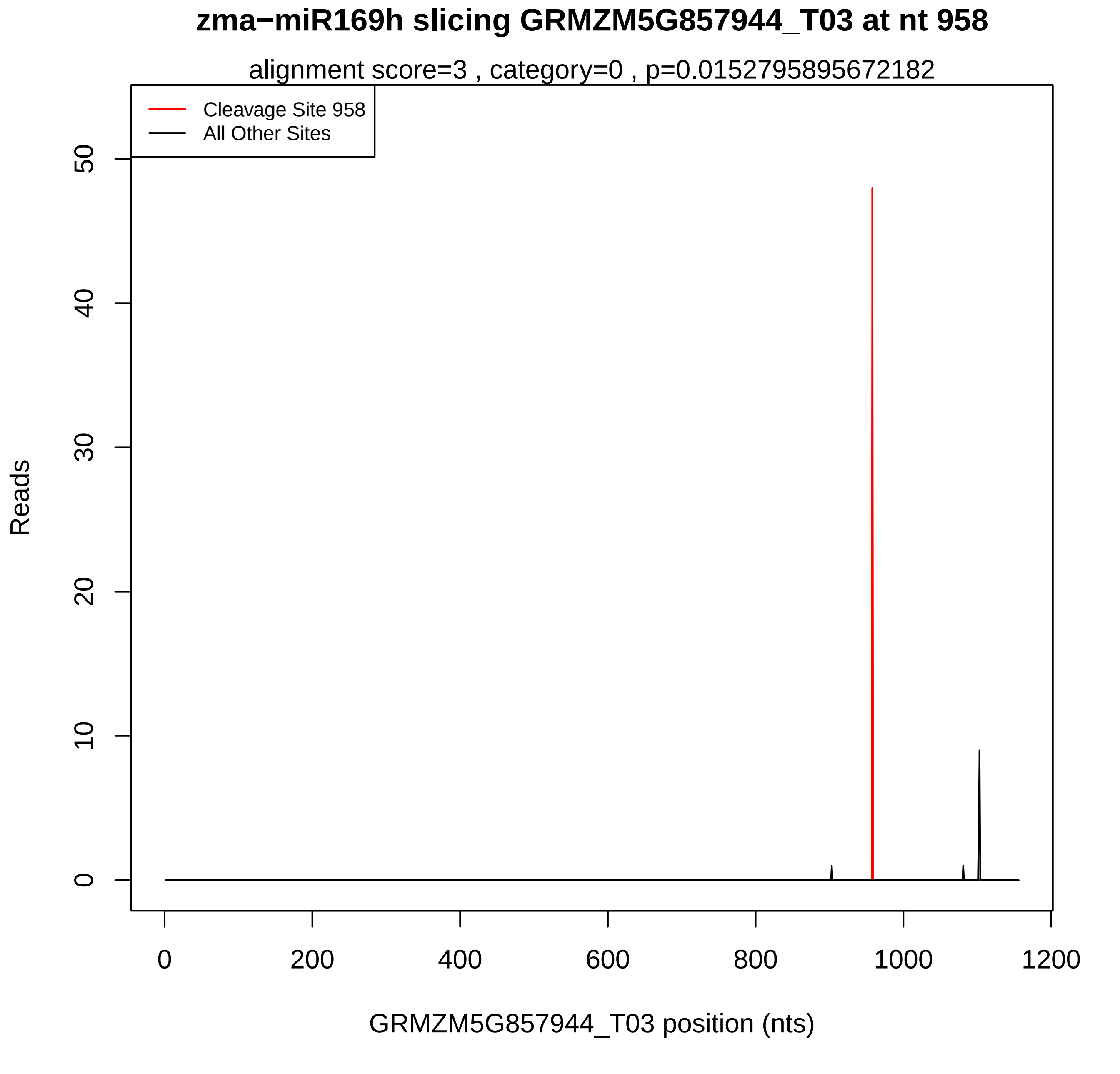
zma−miR169h slicing GRMZM5G857944_T03 at nt 958
alignment score=3 , category=0 , p=0.0152795895672182
Cleavage Site 958
All Other Sites
Reads
0
10
20
30
40
50
0
200
400
600
800
1000
1200
GRMZM5G857944_T03 position (nts)

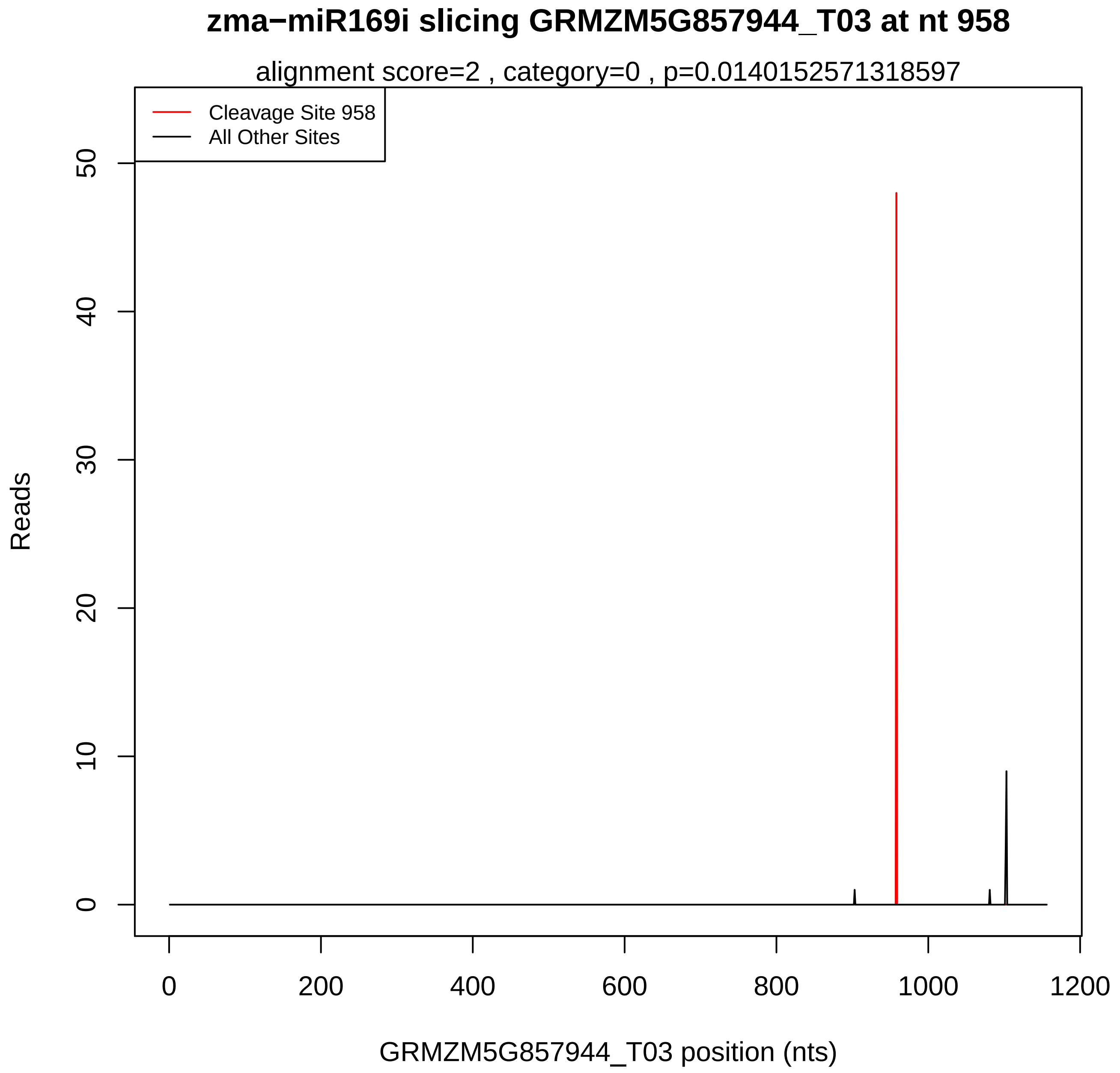
zma−miR169i slicing GRMZM5G857944_T03 at nt 958
alignment score=2 , category=0 , p=0.0140152571318597
Cleavage Site 958
All Other Sites
Reads
0
10
20
30
40
50
0
200
400
600
800
1000
1200
GRMZM5G857944_T03 position (nts)

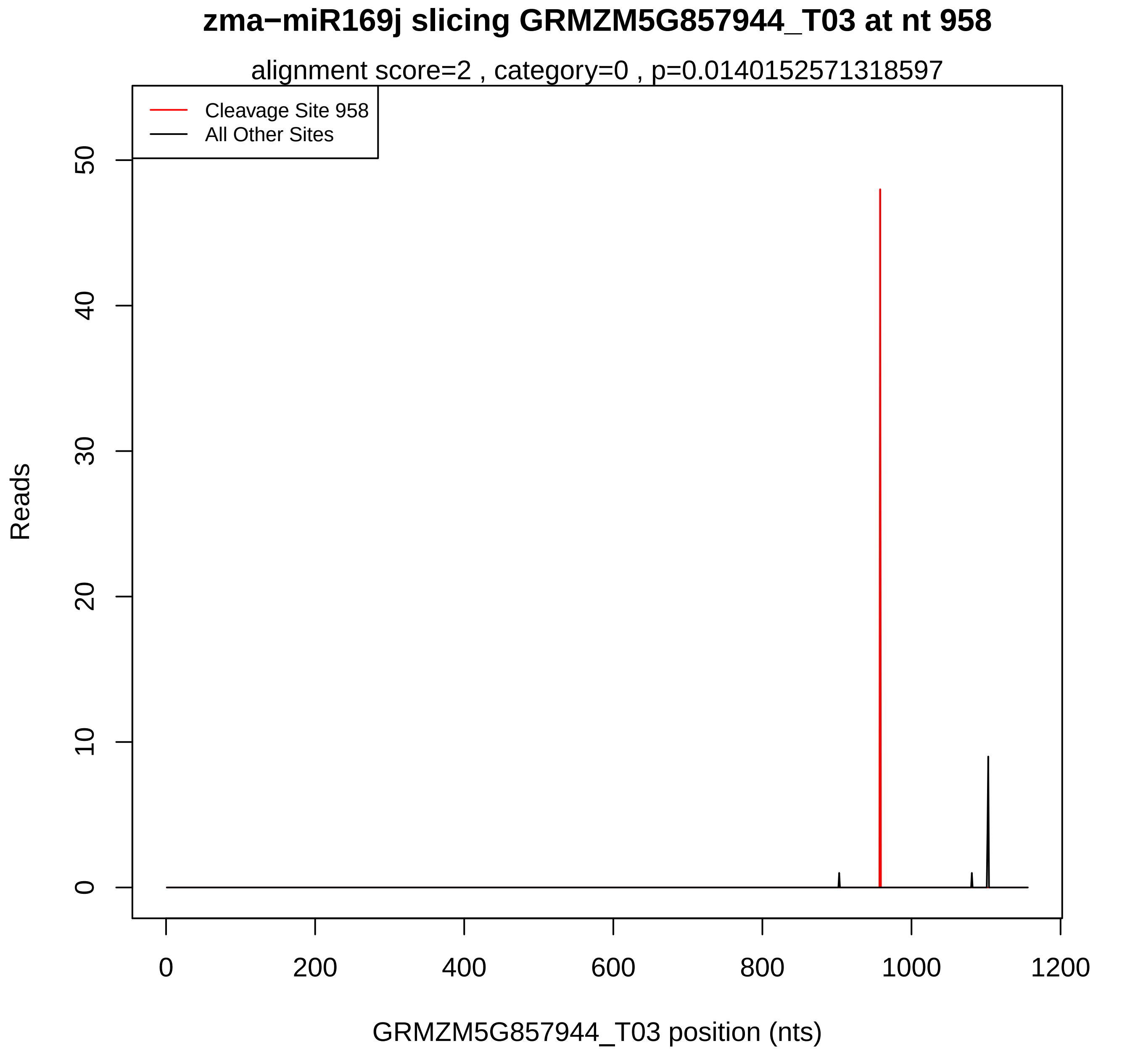

zma−miR169j slicing GRMZM5G857944_T03 at nt 958
alignment score=2 , category=0 , p=0.0140152571318597
Cleavage Site 958
All Other Sites
Reads
0
10
20
30
40
50
0
200
400
600
800
1000
1200
GRMZM5G857944_T03 position (nts)

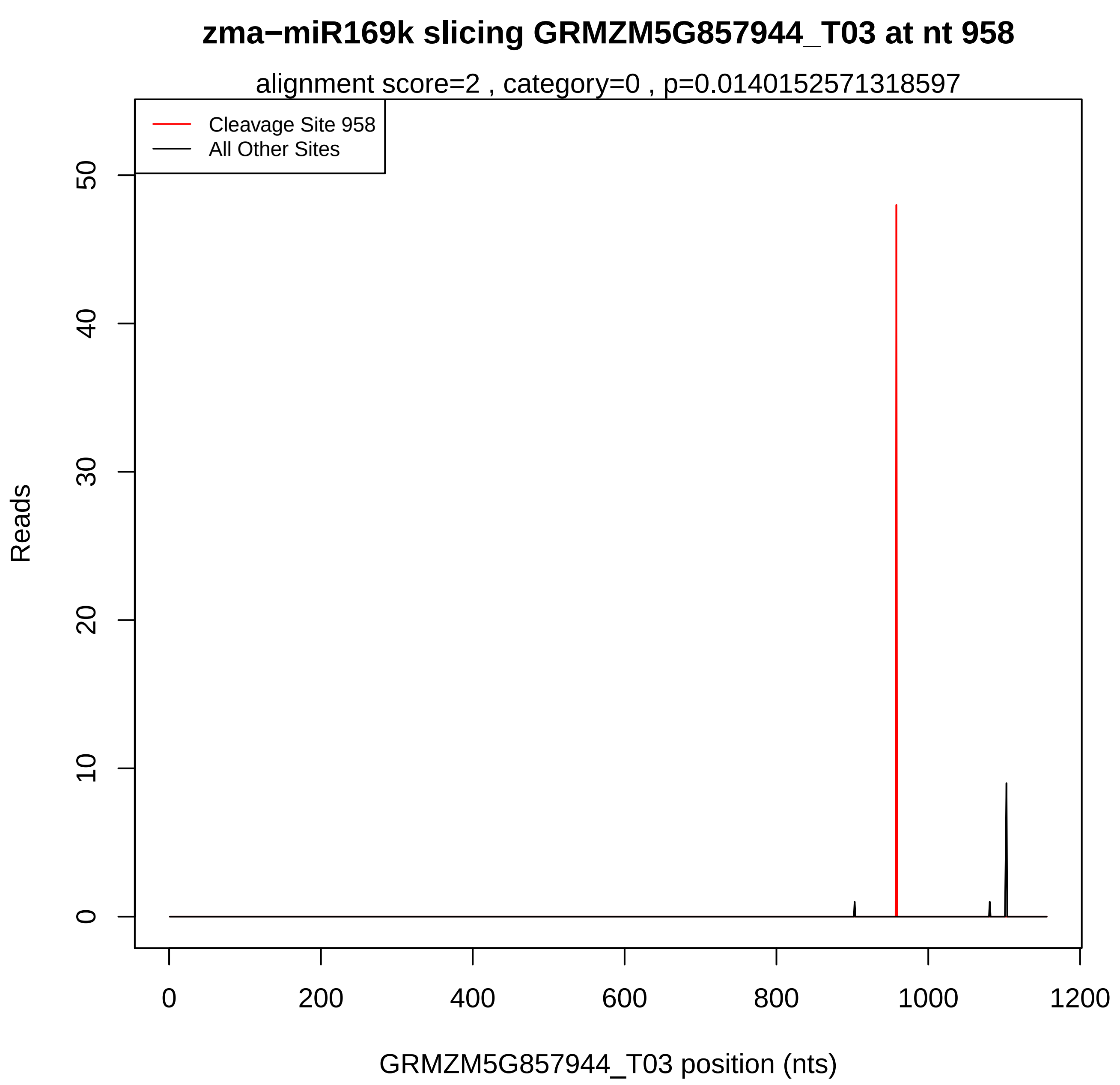
zma−miR169k slicing GRMZM5G857944_T03 at nt 958
alignment score=2 , category=0 , p=0.0140152571318597
Cleavage Site 958
All Other Sites
Reads
0
10
20
30
40
50
0
200
400
600
800
1000
1200
GRMZM5G857944_T03 position (nts)

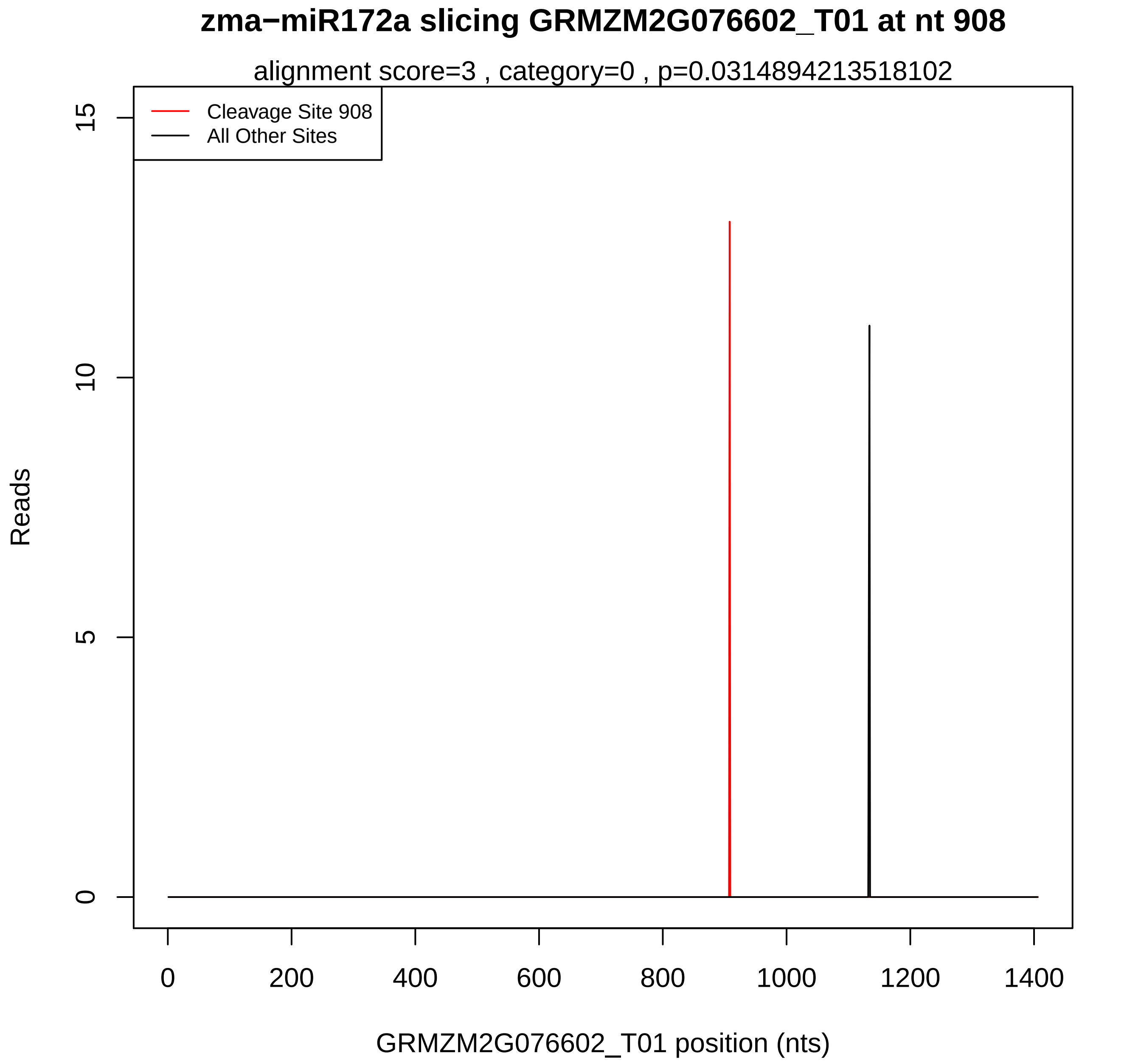

zma−miR172a slicing GRMZM2G076602_T01 at nt 908
alignment score=3 , category=0 , p=0.0314894213518102
Cleavage Site 908
All Other Sites
Reads
0
5
10
15
0
200
400
600
800
1000
1200
1400
GRMZM2G076602_T01 position (nts)

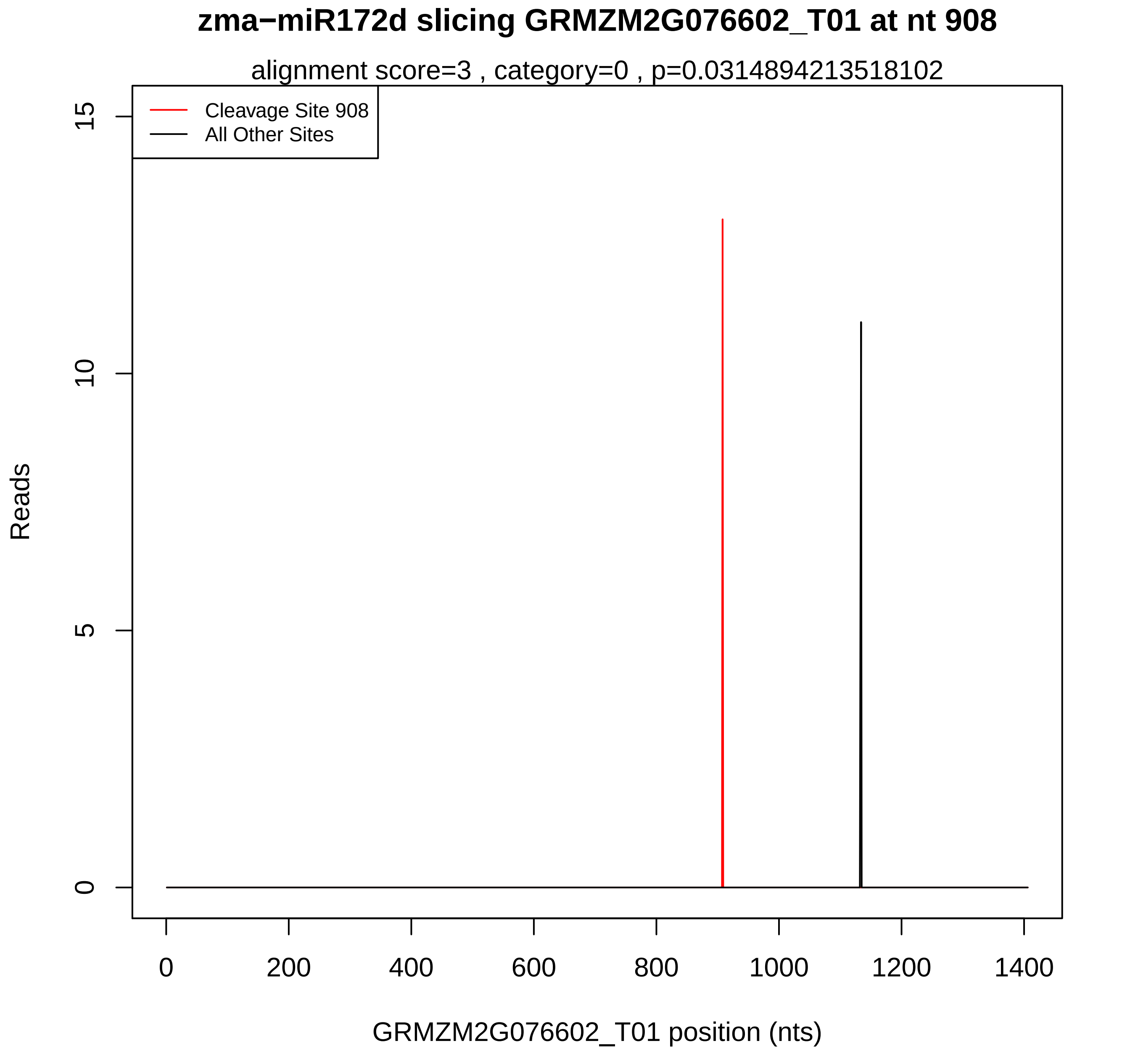
zma−miR172d slicing GRMZM2G076602_T01 at nt 908
alignment score=3 , category=0 , p=0.0314894213518102
Cleavage Site 908
All Other Sites
Reads
0
5
10
15
0
200
400
600
800
1000
1200
1400
GRMZM2G076602_T01 position (nts)

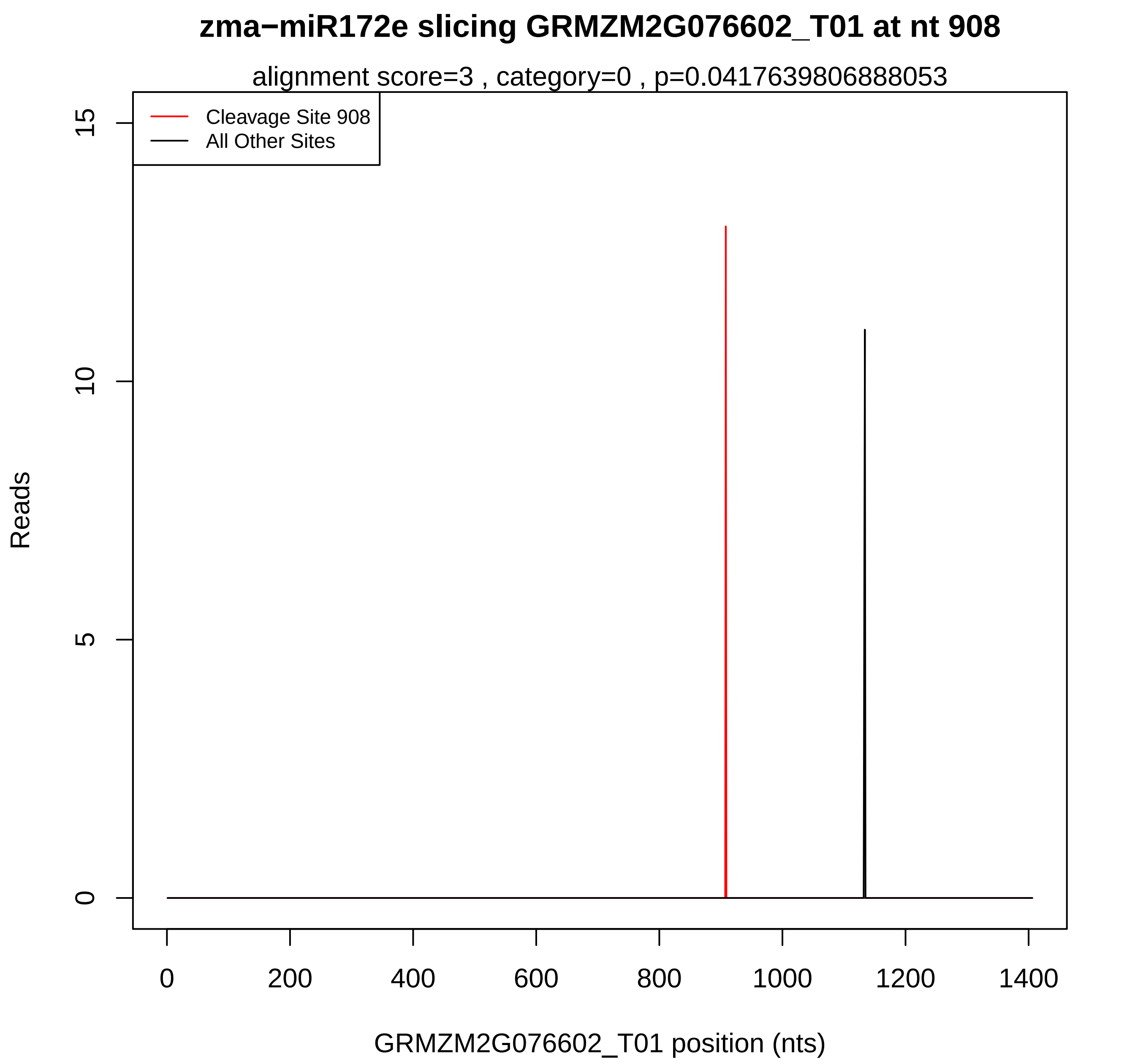
zma−miR172e slicing GRMZM2G076602_T01 at nt 908
alignment score=3 , category=0 , p=0.0417639806888053
Cleavage Site 908
All Other Sites
Reads
0
5
10
15
0
200
400
600
800
1000
1200
1400
GRMZM2G076602_T01 position (nts)

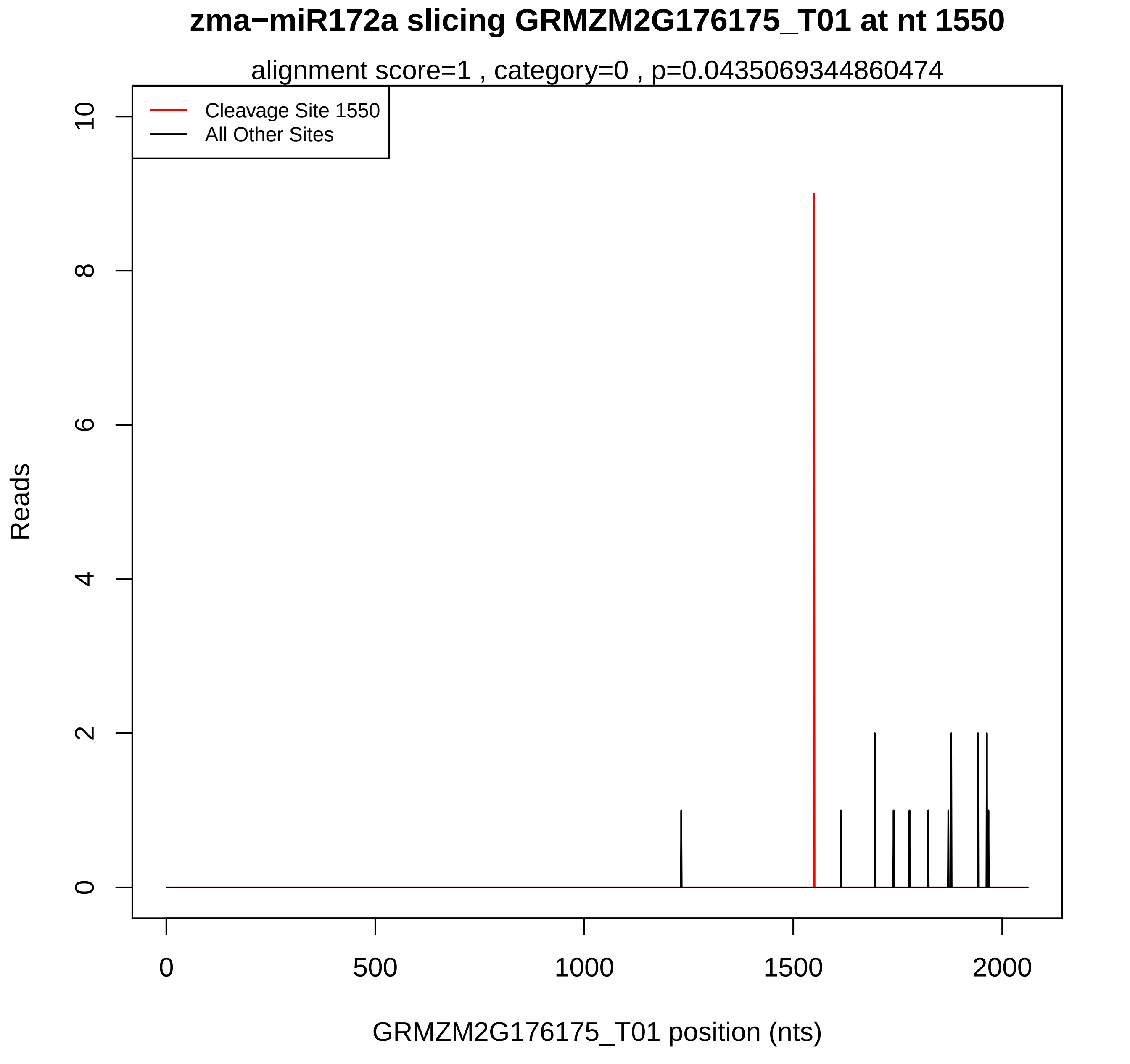

zma−miR172a slicing GRMZM2G176175_T01 at nt 1550
alignment score=1 , category=0 , p=0.0435069344860474
Cleavage Site 1550
All Other Sites
Reads
0
2
4
6
8
10
0
500
1000
1500
2000
GRMZM2G176175_T01 position (nts)

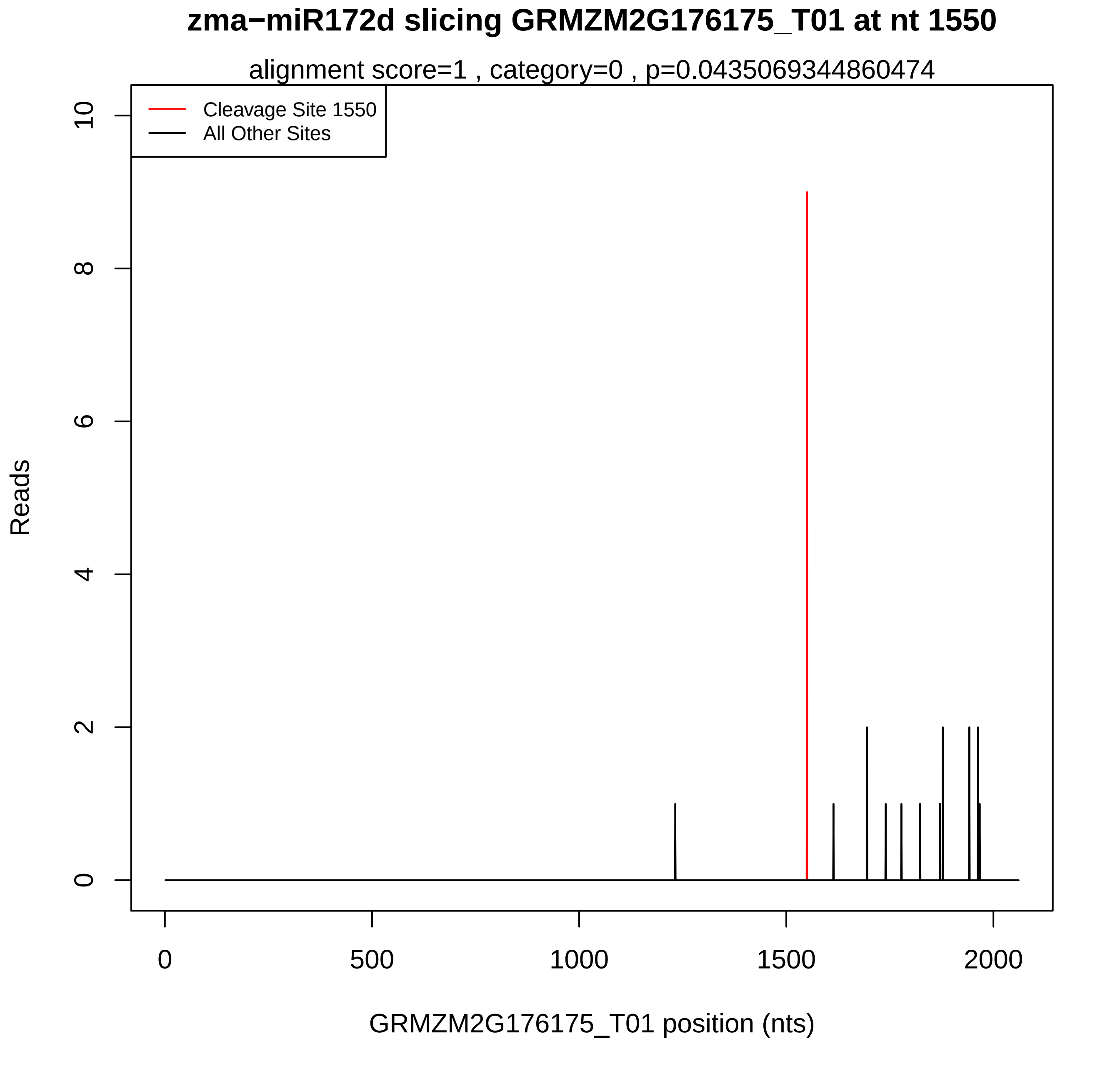

zma−miR172d slicing GRMZM2G176175_T01 at nt 1550
alignment score=1 , category=0 , p=0.0435069344860474
Cleavage Site 1550
All Other Sites
Reads
0
2
4
6
8
10
0
500
1000
1500
2000
GRMZM2G176175_T01 position (nts)

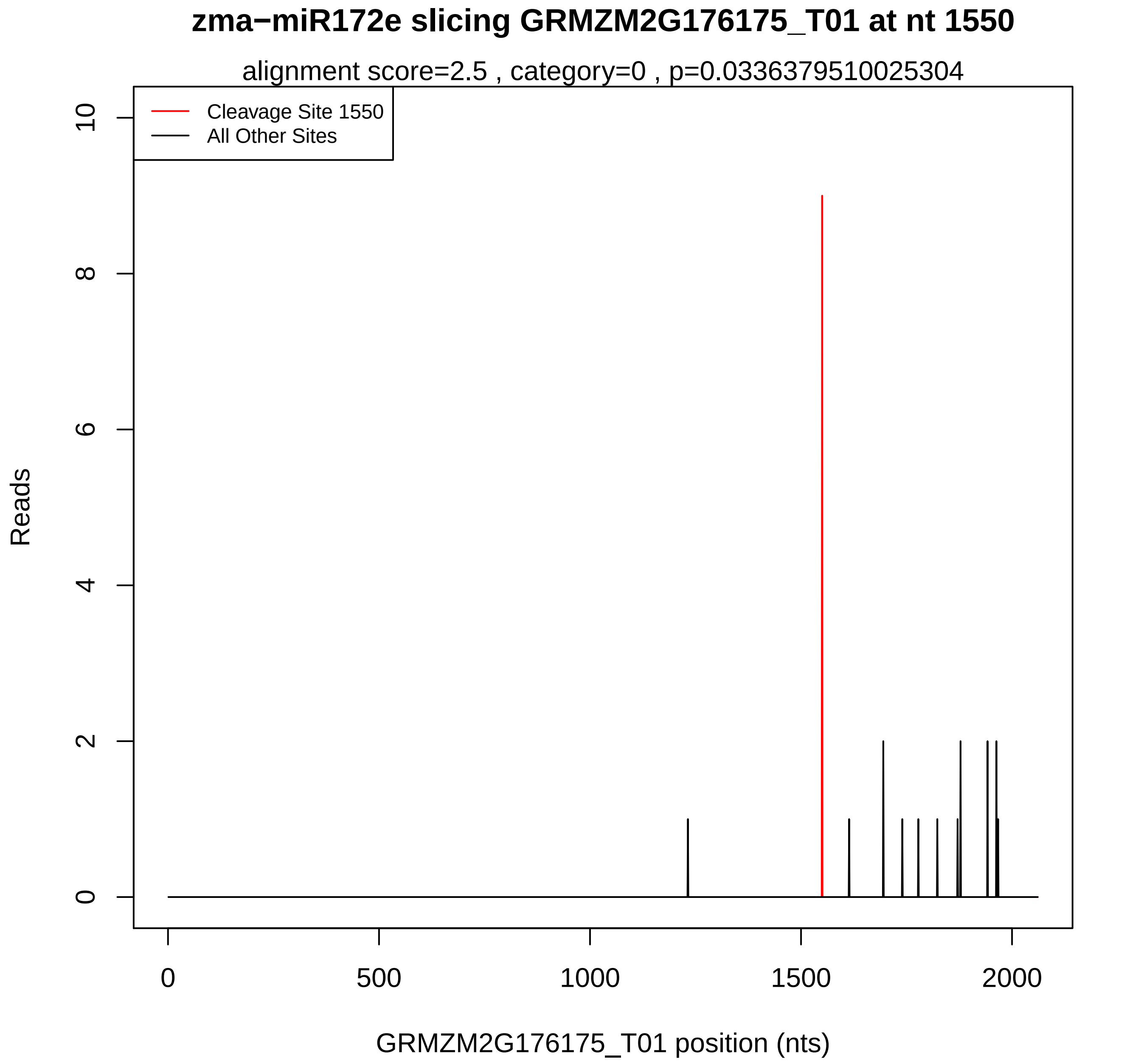
zma−miR172e slicing GRMZM2G176175_T01 at nt 1550
alignment score=2.5 , category=0 , p=0.0336379510025304
Cleavage Site 1550
All Other Sites
Reads
0
2
4
6
8
10
0
500
1000
1500
2000
GRMZM2G176175_T01 position (nts)

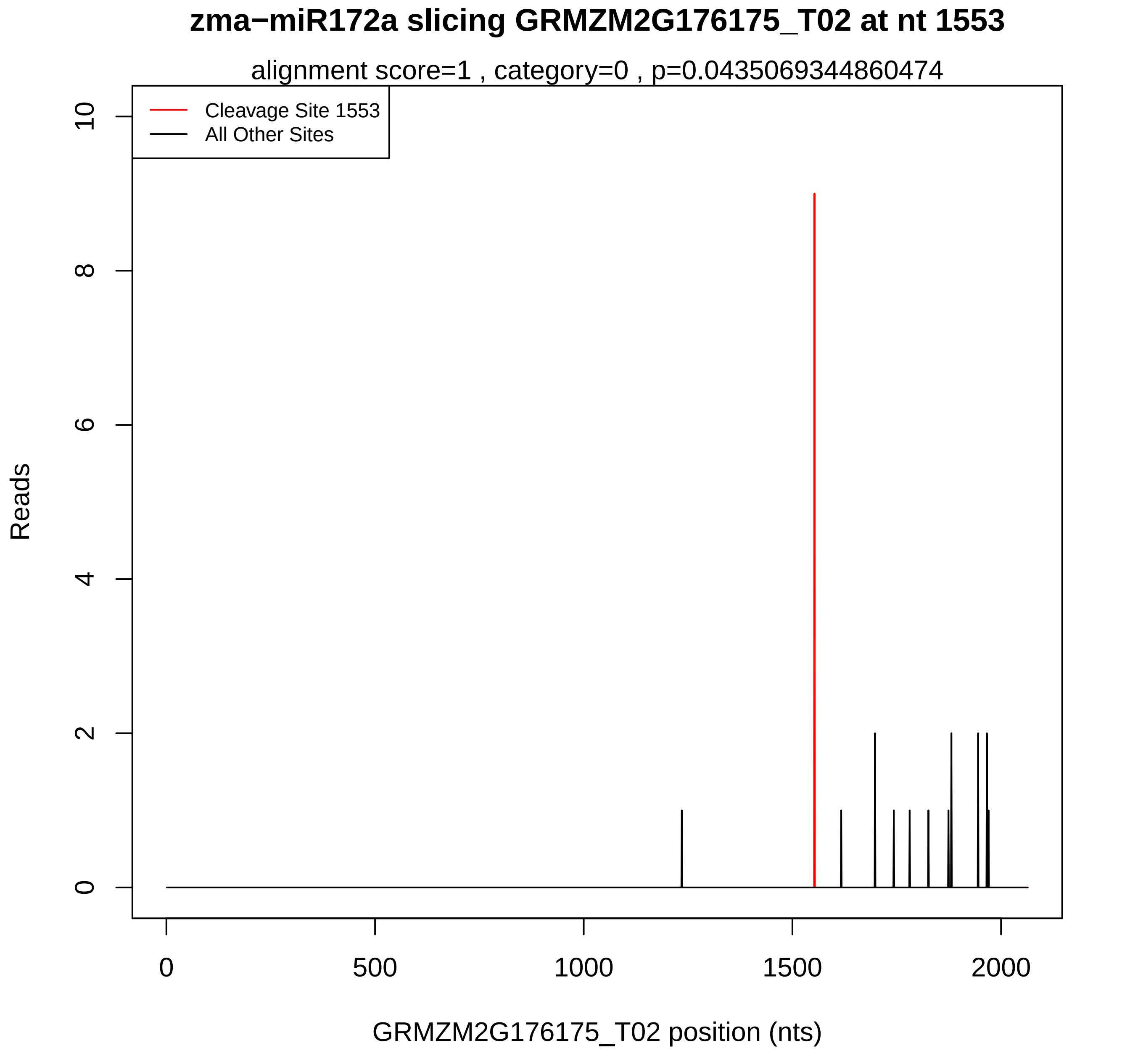

zma−miR172a slicing GRMZM2G176175_T02 at nt 1553
alignment score=1 , category=0 , p=0.0435069344860474
Cleavage Site 1553
All Other Sites
Reads
0
2
4
6
8
10
0
500
1000
1500
2000
GRMZM2G176175_T02 position (nts)

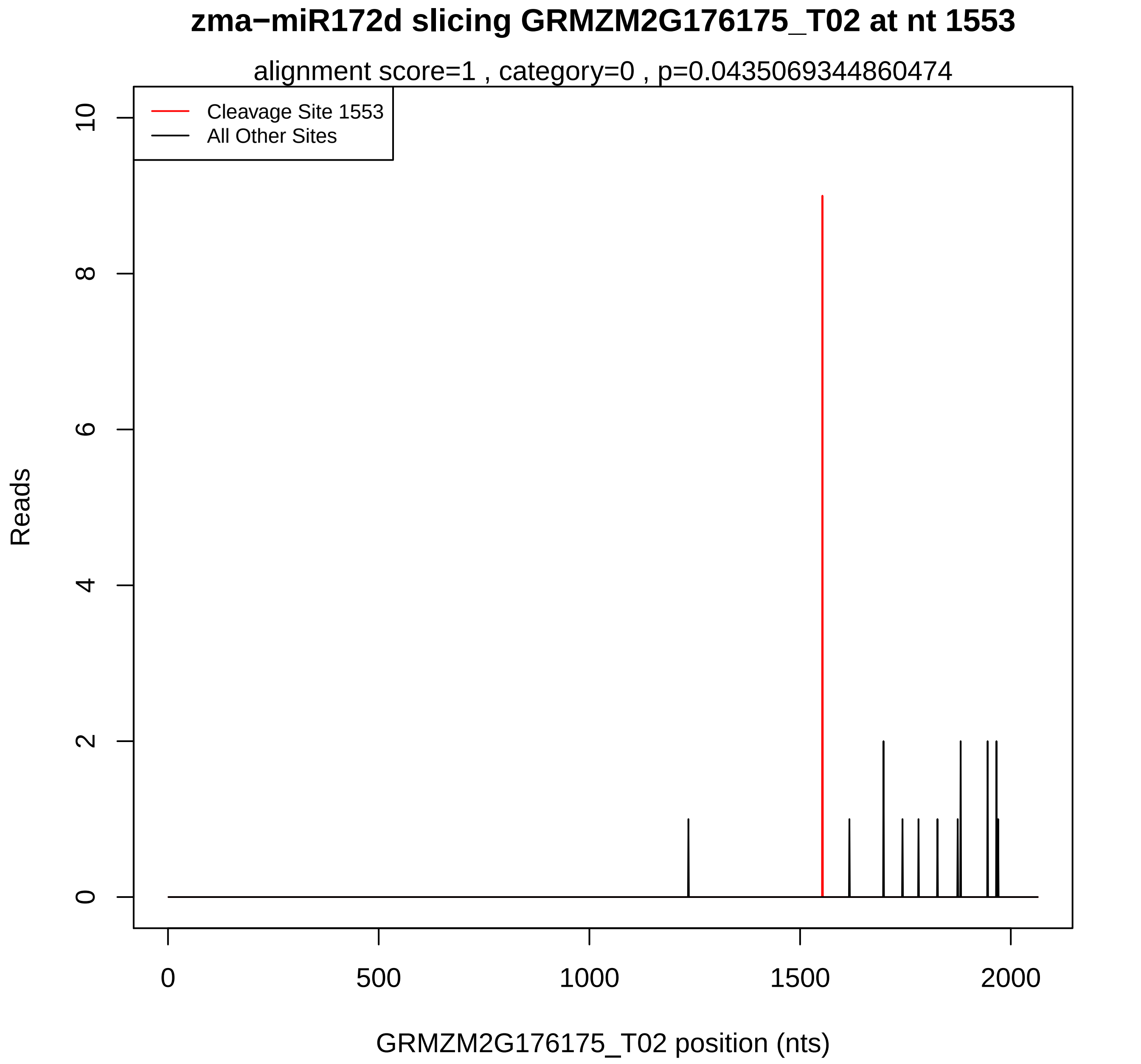

zma−miR172d slicing GRMZM2G176175_T02 at nt 1553
alignment score=1 , category=0 , p=0.0435069344860474
Cleavage Site 1553
All Other Sites
Reads
0
2
4
6
8
10
0
500
1000
1500
2000
GRMZM2G176175_T02 position (nts)

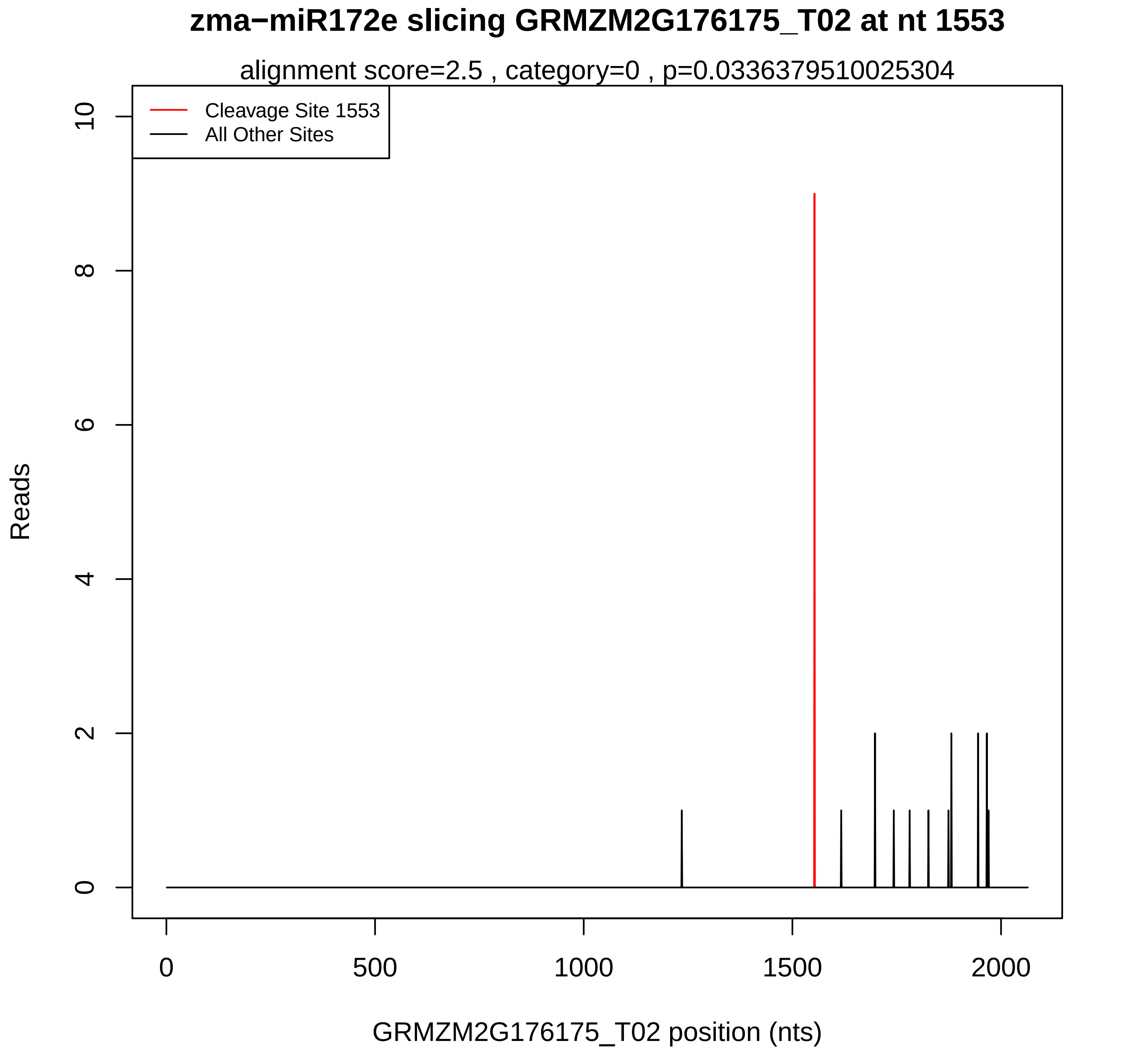
zma−miR172e slicing GRMZM2G176175_T02 at nt 1553
alignment score=2.5 , category=0 , p=0.0336379510025304
Cleavage Site 1553
All Other Sites
Reads
0
2
4
6
8
10
0
500
1000
1500
2000
GRMZM2G176175_T02 position (nts)

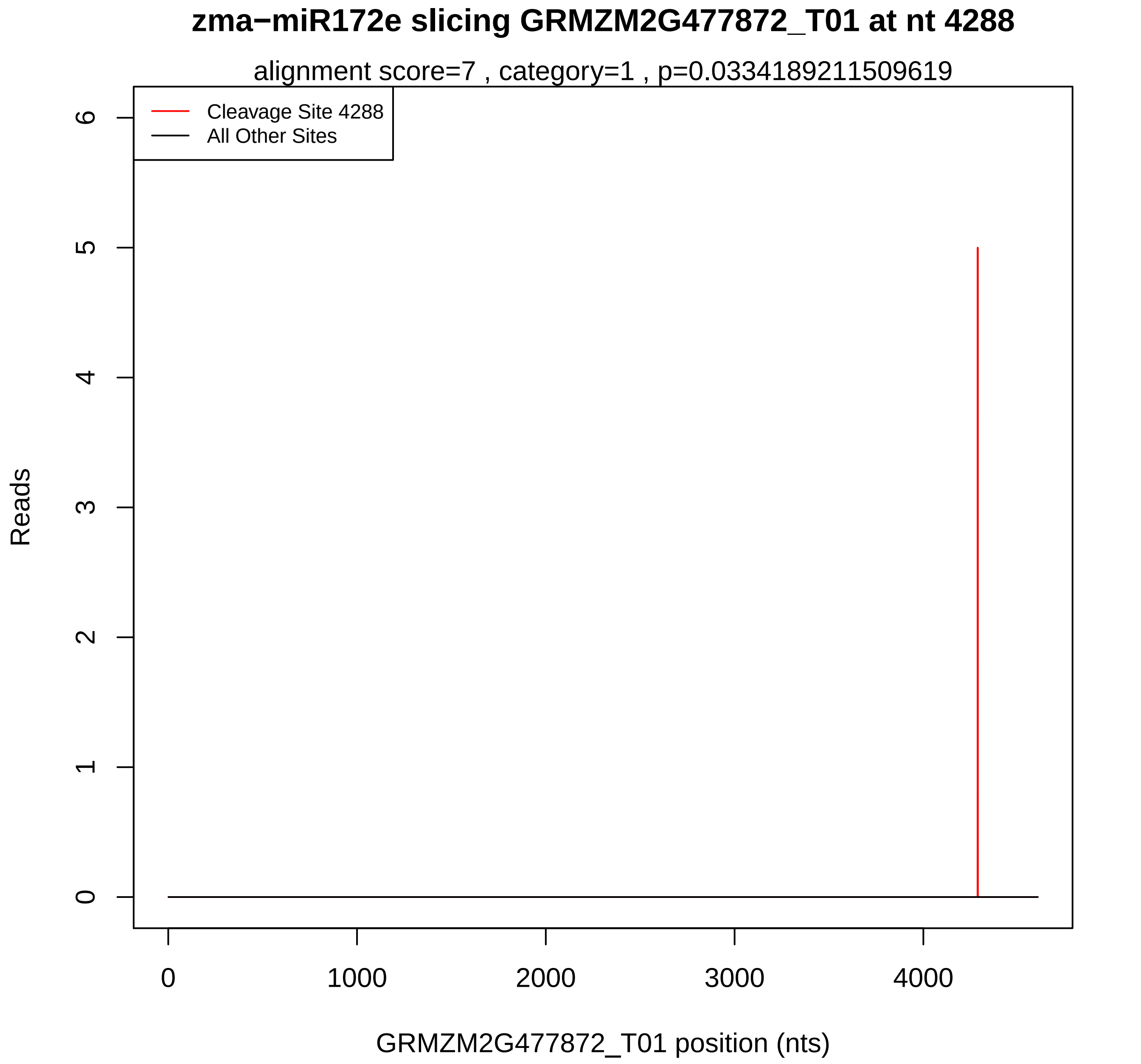

zma−miR172e slicing GRMZM2G477872_T01 at nt 4288
alignment score=7 , category=1 , p=0.0334189211509619
Cleavage Site 4288
All Other Sites
Reads
0
1
2
3
4
5
6
0
1000
2000
3000
4000
GRMZM2G477872_T01 position (nts)

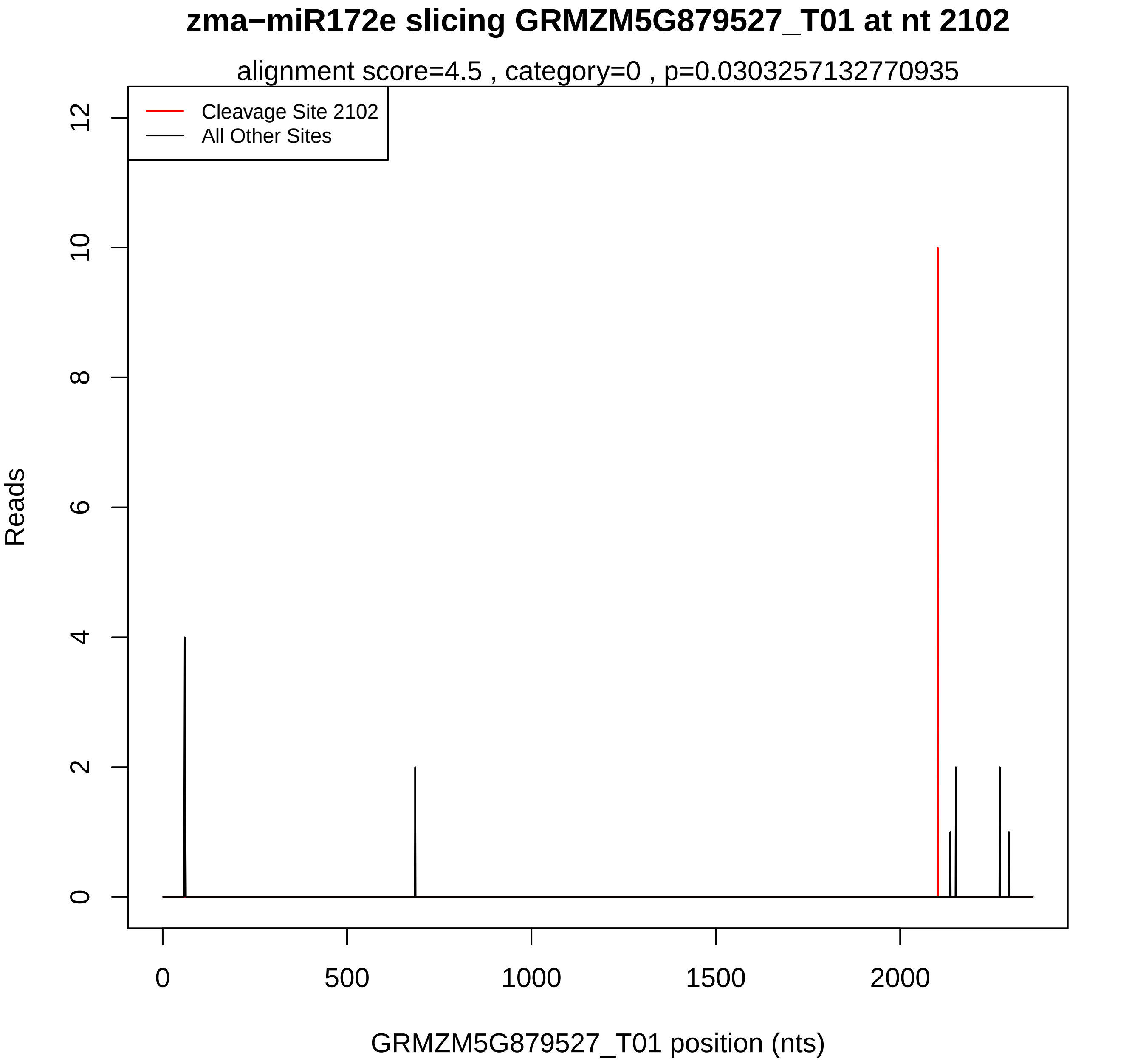

zma−miR172e slicing GRMZM5G879527_T01 at nt 2102
alignment score=4.5 , category=0 , p=0.0303257132770935
Cleavage Site 2102
All Other Sites
Reads
0
2
4
6
8
10
12
0
500
1000
1500
2000
GRMZM5G879527_T01 position (nts)

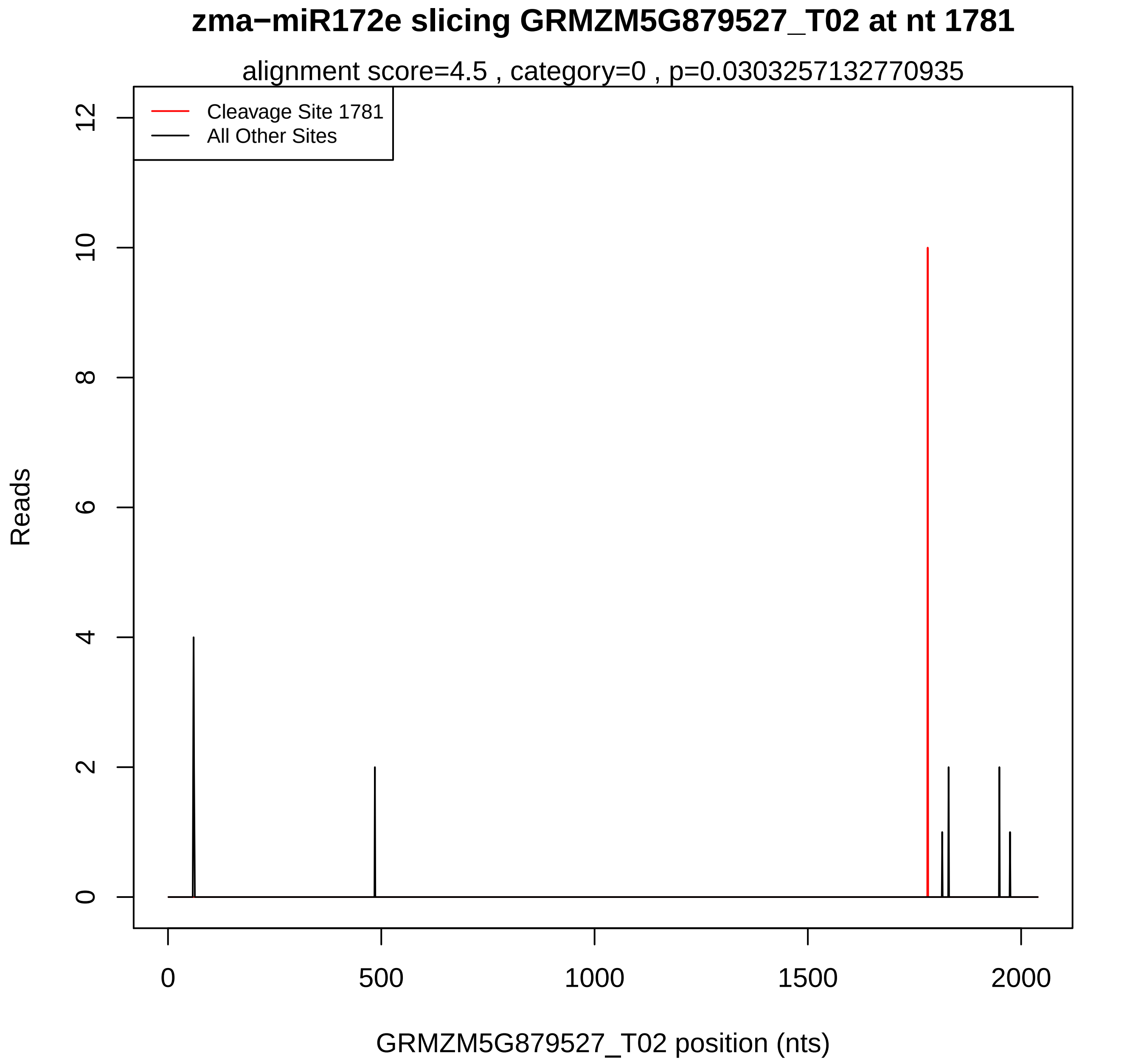
zma−miR172e slicing GRMZM5G879527_T02 at nt 1781
alignment score=4.5 , category=0 , p=0.0303257132770935
Cleavage Site 1781
All Other Sites
Reads
0
2
4
6
8
10
12
0
500
1000
1500
2000
GRMZM5G879527_T02 position (nts)

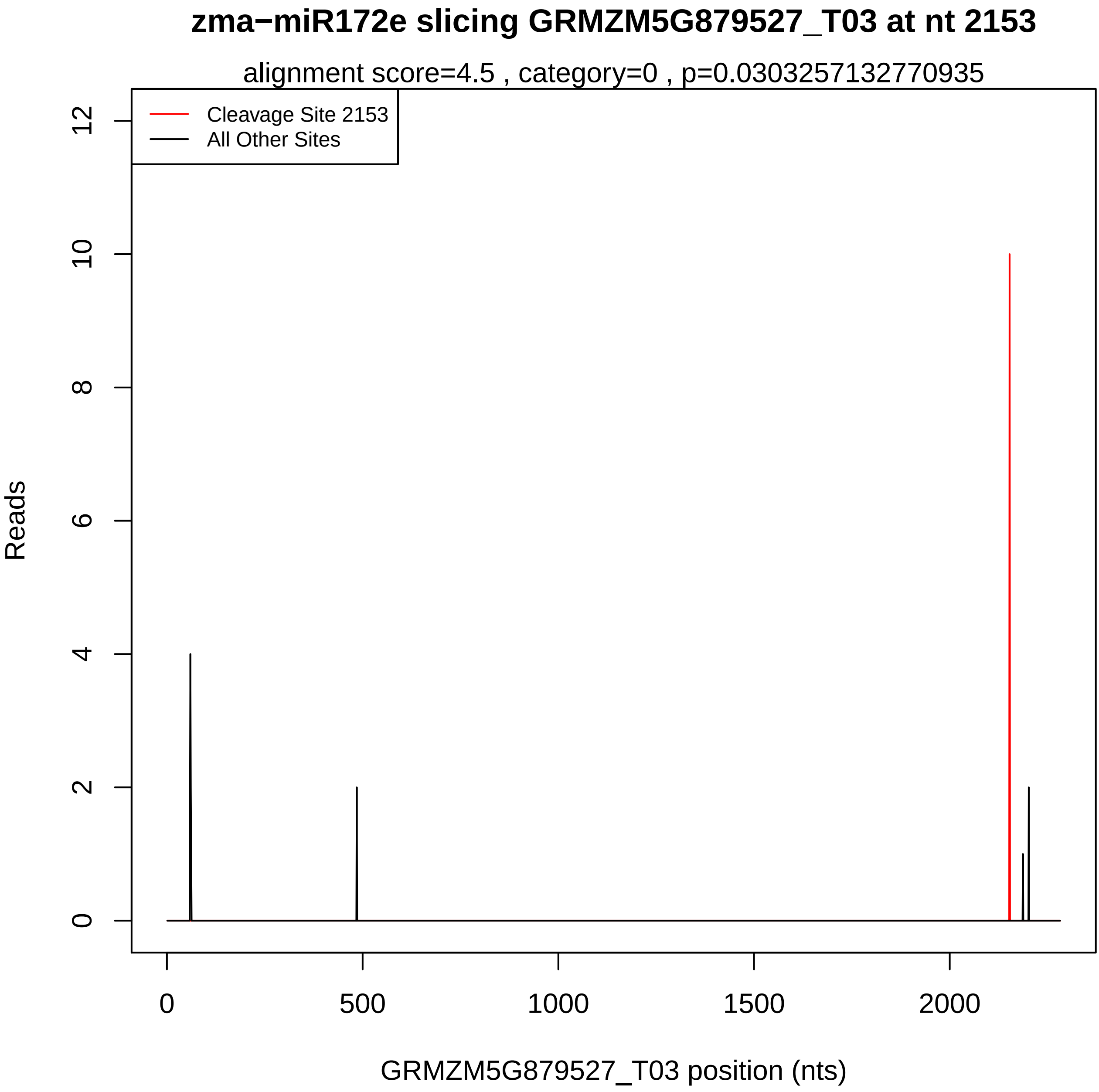

zma−miR172e slicing GRMZM5G879527_T03 at nt 2153
alignment score=4.5 , category=0 , p=0.0303257132770935
Cleavage Site 2153
All Other Sites
Reads
0
2
4
6
8
10
12
0
500
1000
1500
2000
GRMZM5G879527_T03 position (nts)

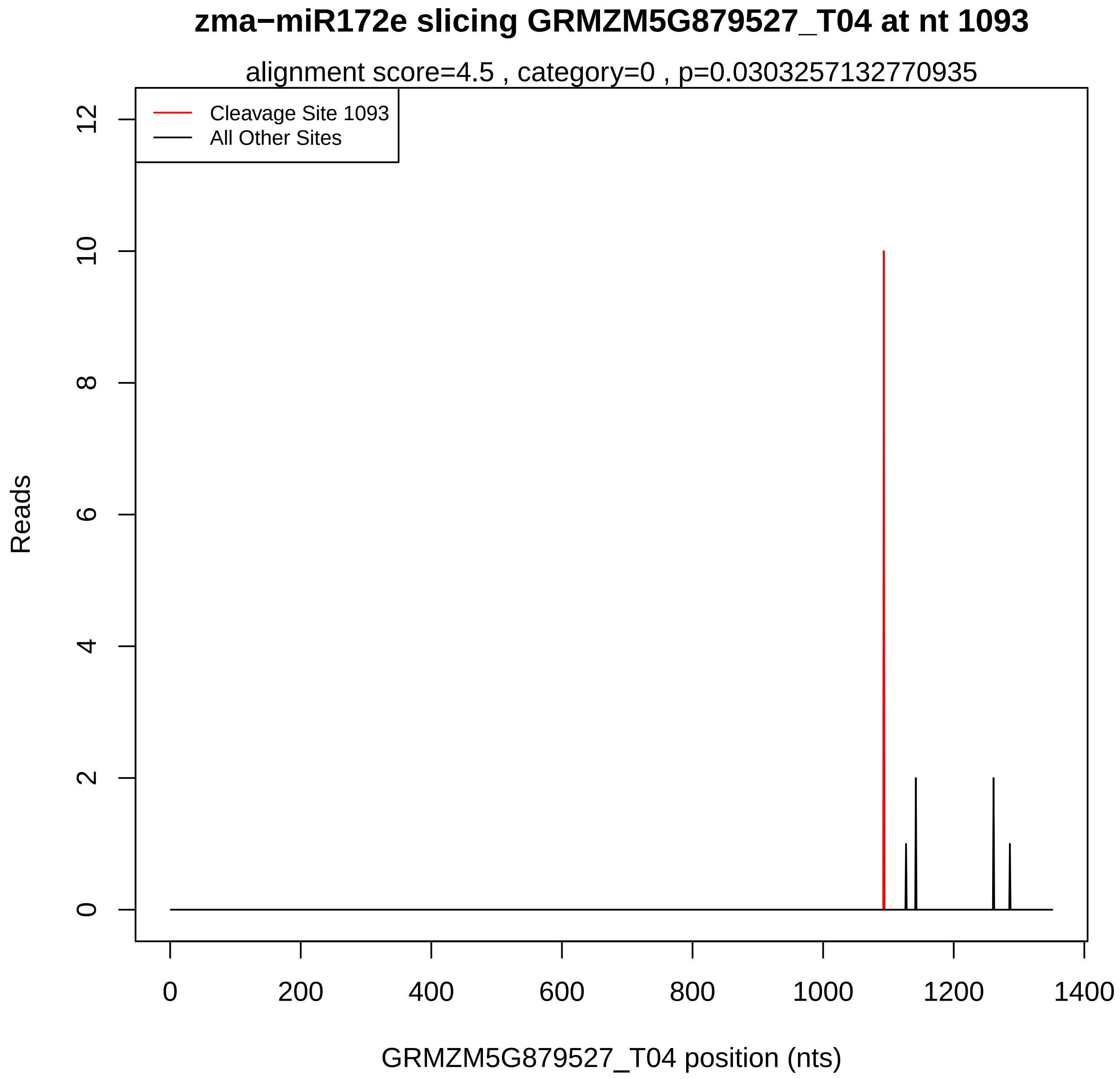

zma−miR172e slicing GRMZM5G879527_T04 at nt 1093
alignment score=4.5 , category=0 , p=0.0303257132770935
Cleavage Site 1093
All Other Sites
Reads
0
2
4
6
8
10
12
0
200
400
600
800
1000
1200
1400
GRMZM5G879527_T04 position (nts)

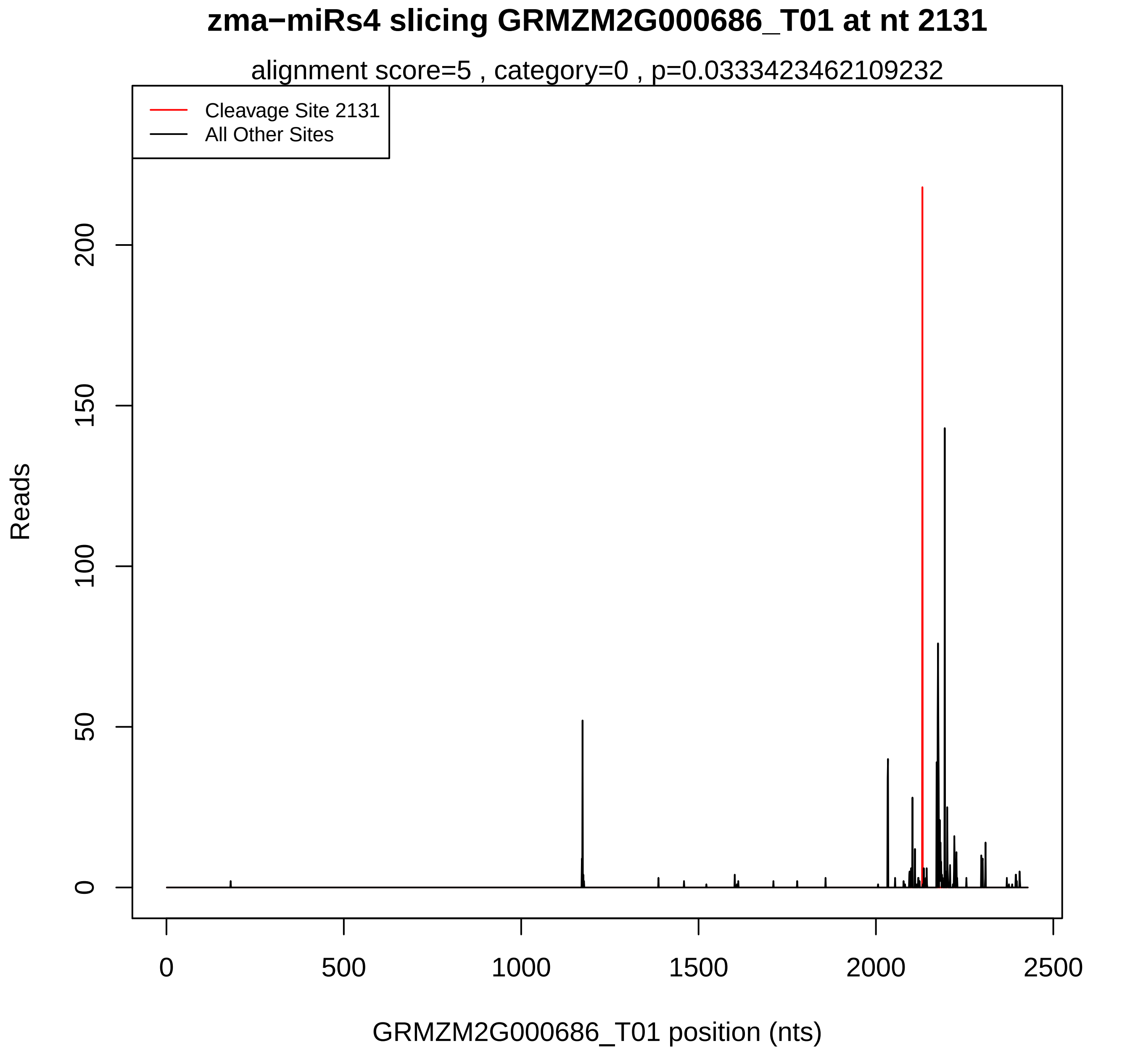
zma−miRs4 slicing GRMZM2G000686_T01 at nt 2131
alignment score=5 , category=0 , p=0.0333423462109232
Cleavage Site 2131
All Other Sites
Reads
0
50
100
150
200
0
500
1000
1500
2000
2500
GRMZM2G000686_T01 position (nts)

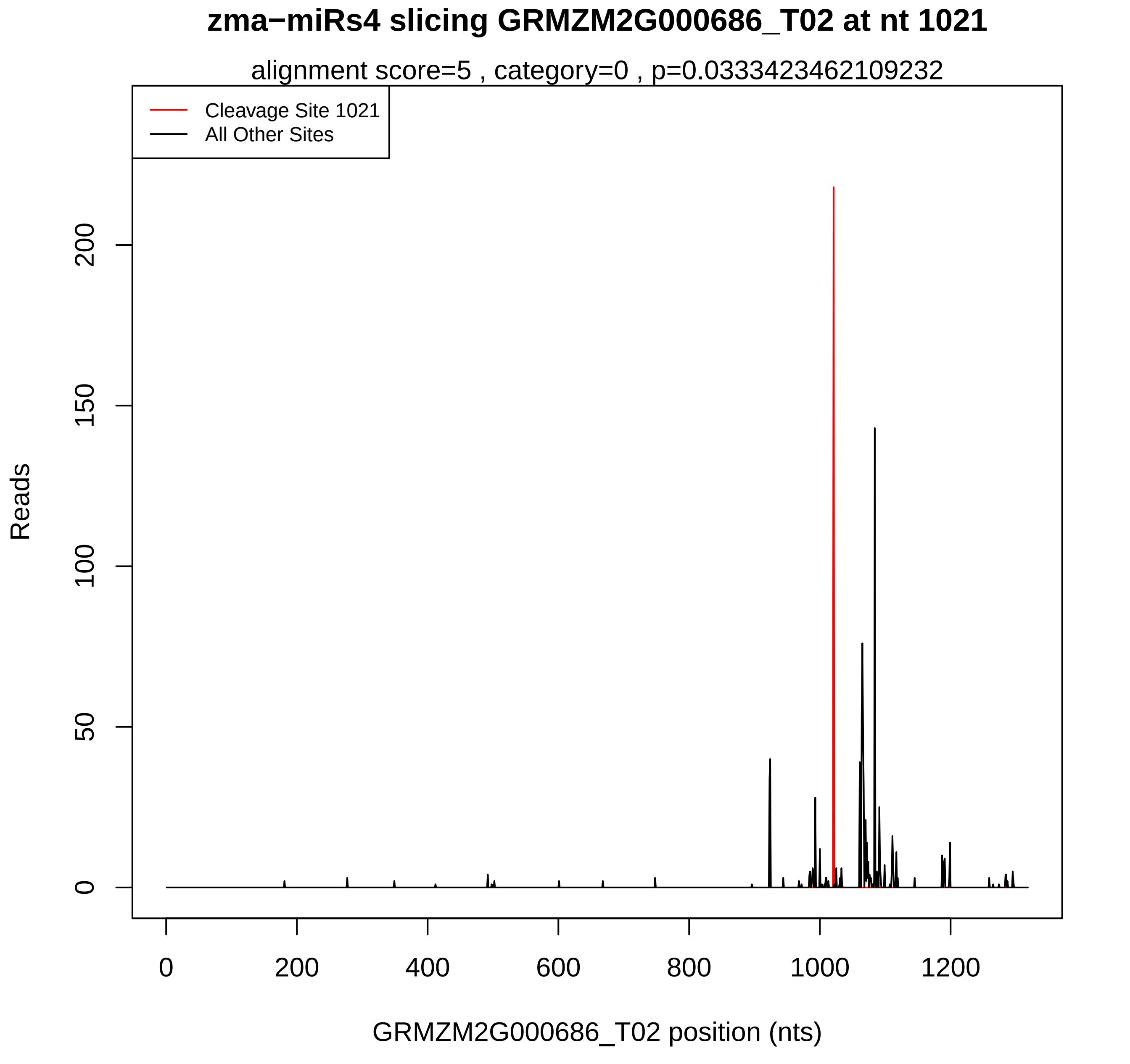
zma−miRs4 slicing GRMZM2G000686_T02 at nt 1021
alignment score=5 , category=0 , p=0.0333423462109232
Cleavage Site 1021
All Other Sites
Reads
0
50
100
150
200
0
200
400
600
800
1000
1200
GRMZM2G000686_T02 position (nts)

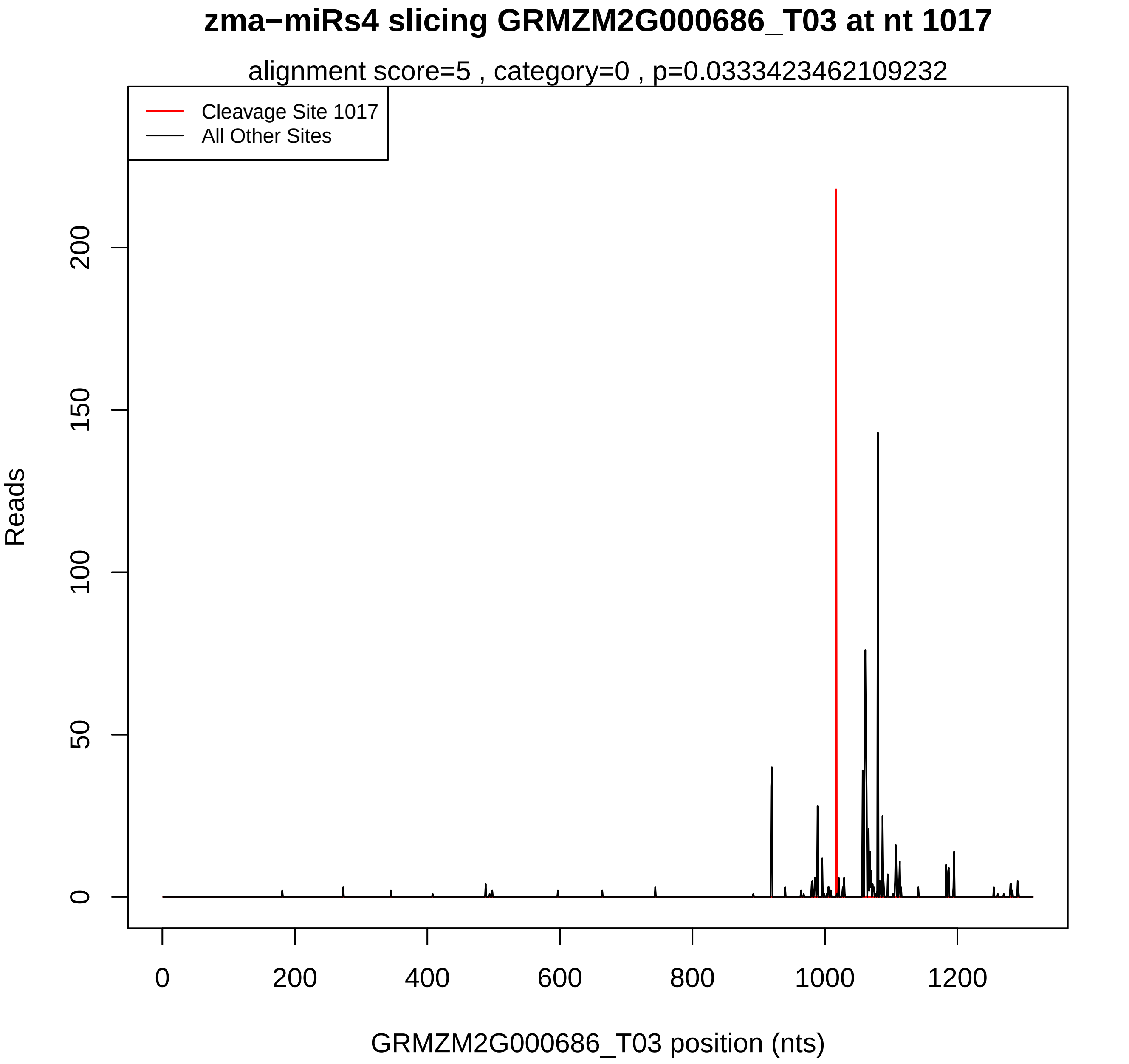
zma−miRs4 slicing GRMZM2G000686_T03 at nt 1017
alignment score=5 , category=0 , p=0.0333423462109232
Cleavage Site 1017
All Other Sites
Reads
0
50
100
150
200
0
200
400
600
800
1000
1200
GRMZM2G000686_T03 position (nts)

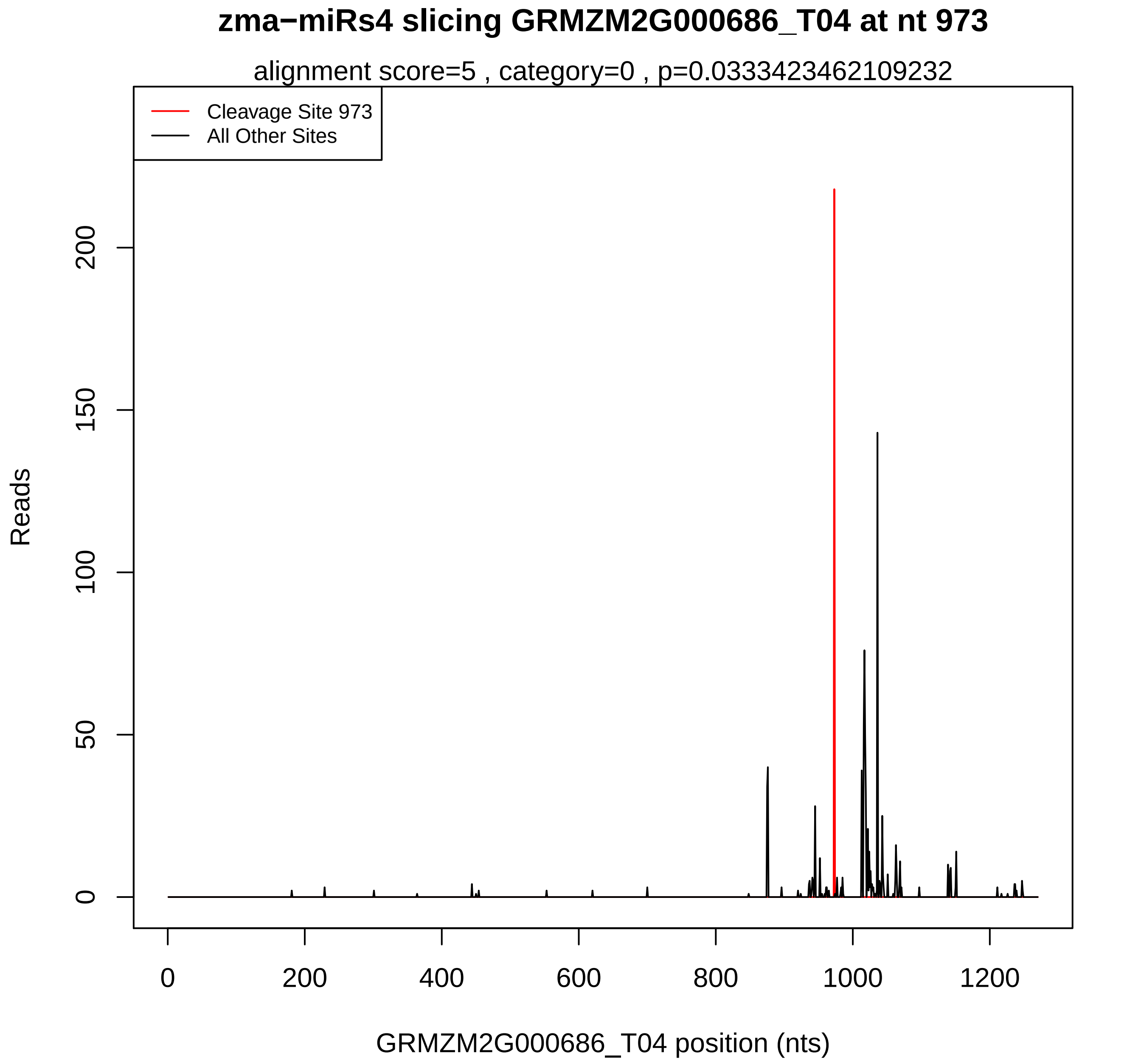
zma−miRs4 slicing GRMZM2G000686_T04 at nt 973
alignment score=5 , category=0 , p=0.0333423462109232
Cleavage Site 973
All Other Sites
Reads
0
50
100
150
200
0
200
400
600
800
1000
1200
GRMZM2G000686_T04 position (nts)

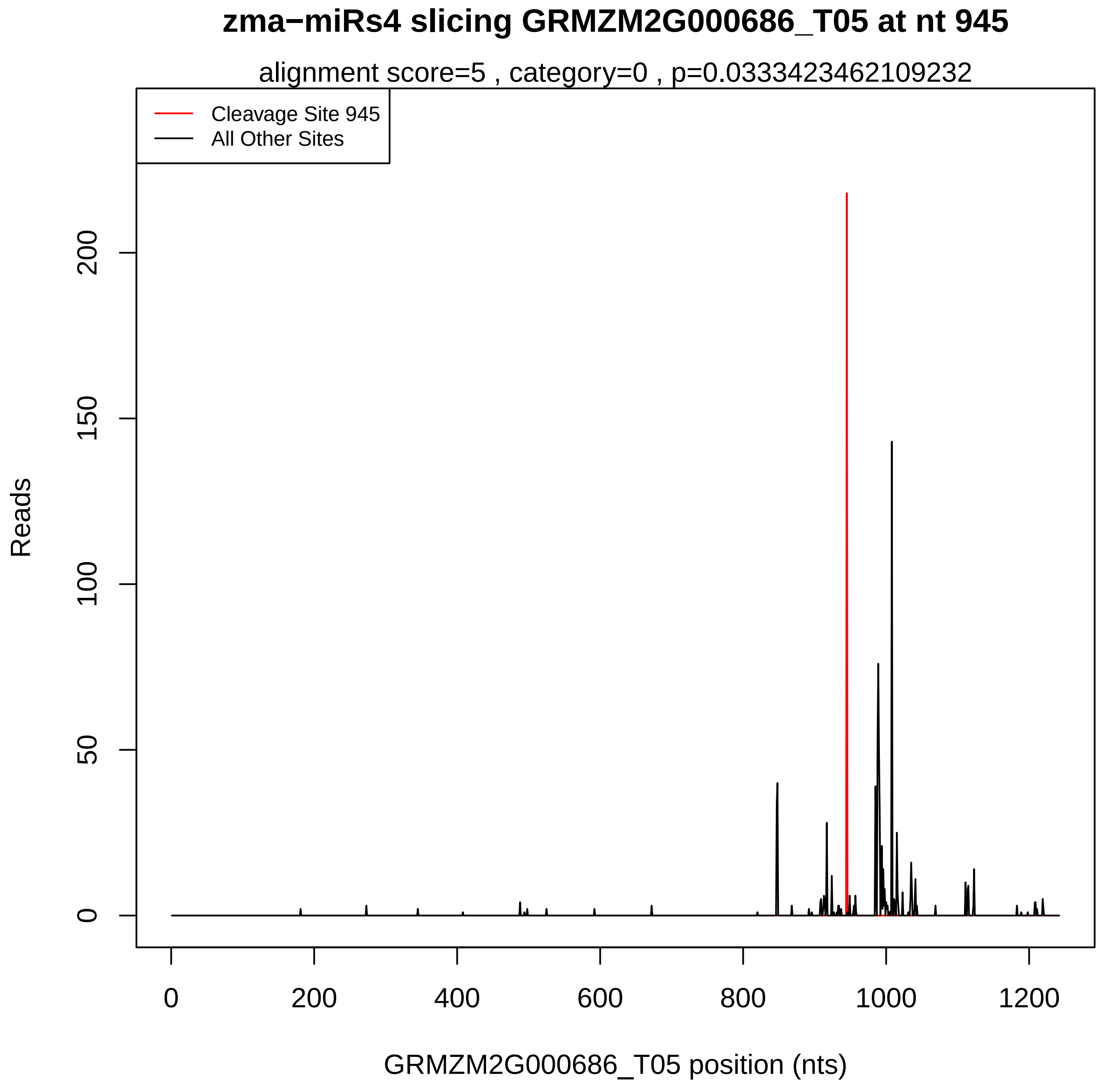
zma−miRs4 slicing GRMZM2G000686_T05 at nt 945
alignment score=5 , category=0 , p=0.0333423462109232
Cleavage Site 945
All Other Sites
Reads
0
50
100
150
200
0
200
400
600
800
1000
1200
GRMZM2G000686_T05 position (nts)

zma−miRs4 slicing GRMZM2G000686_T06 at nt 901

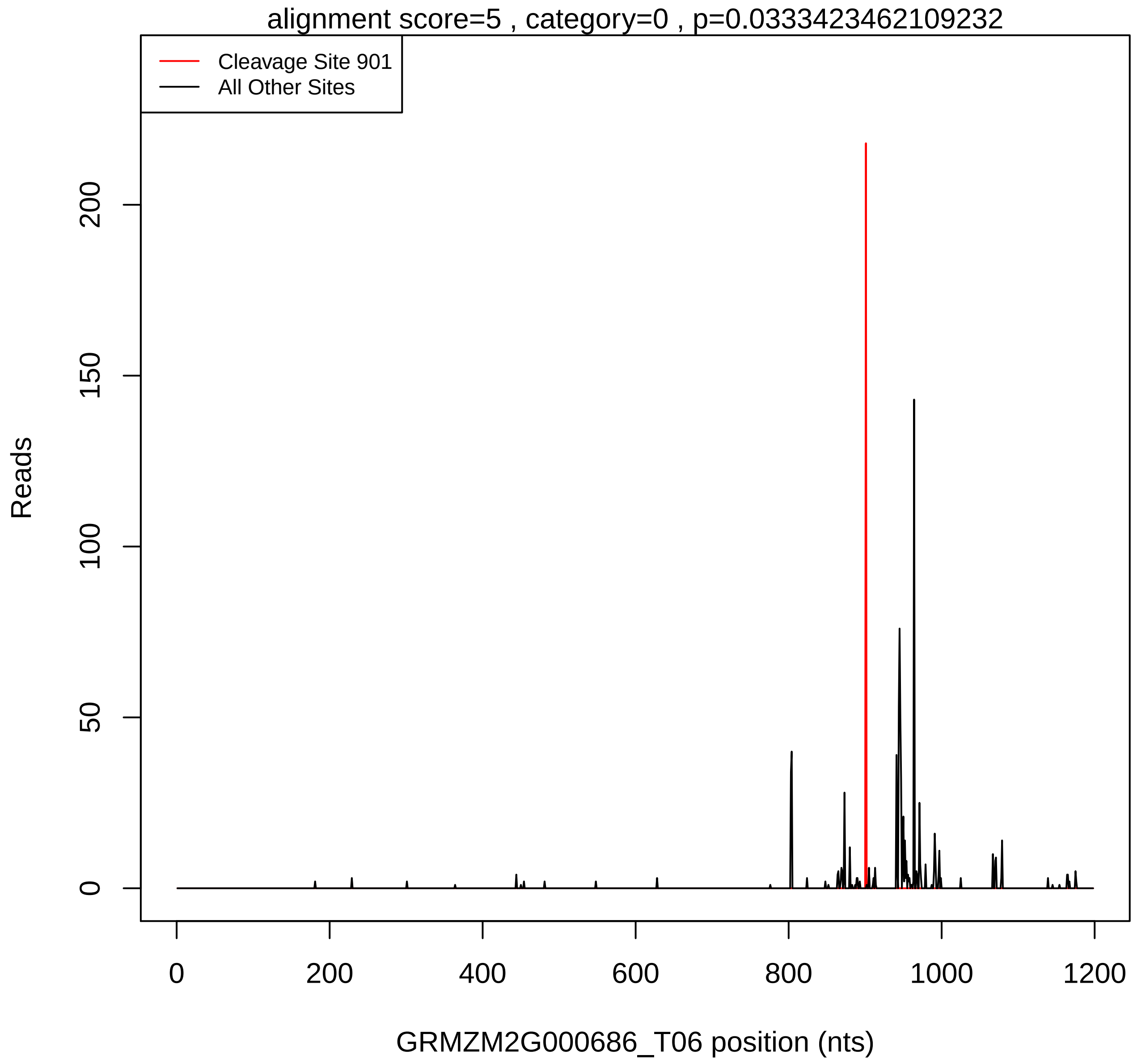

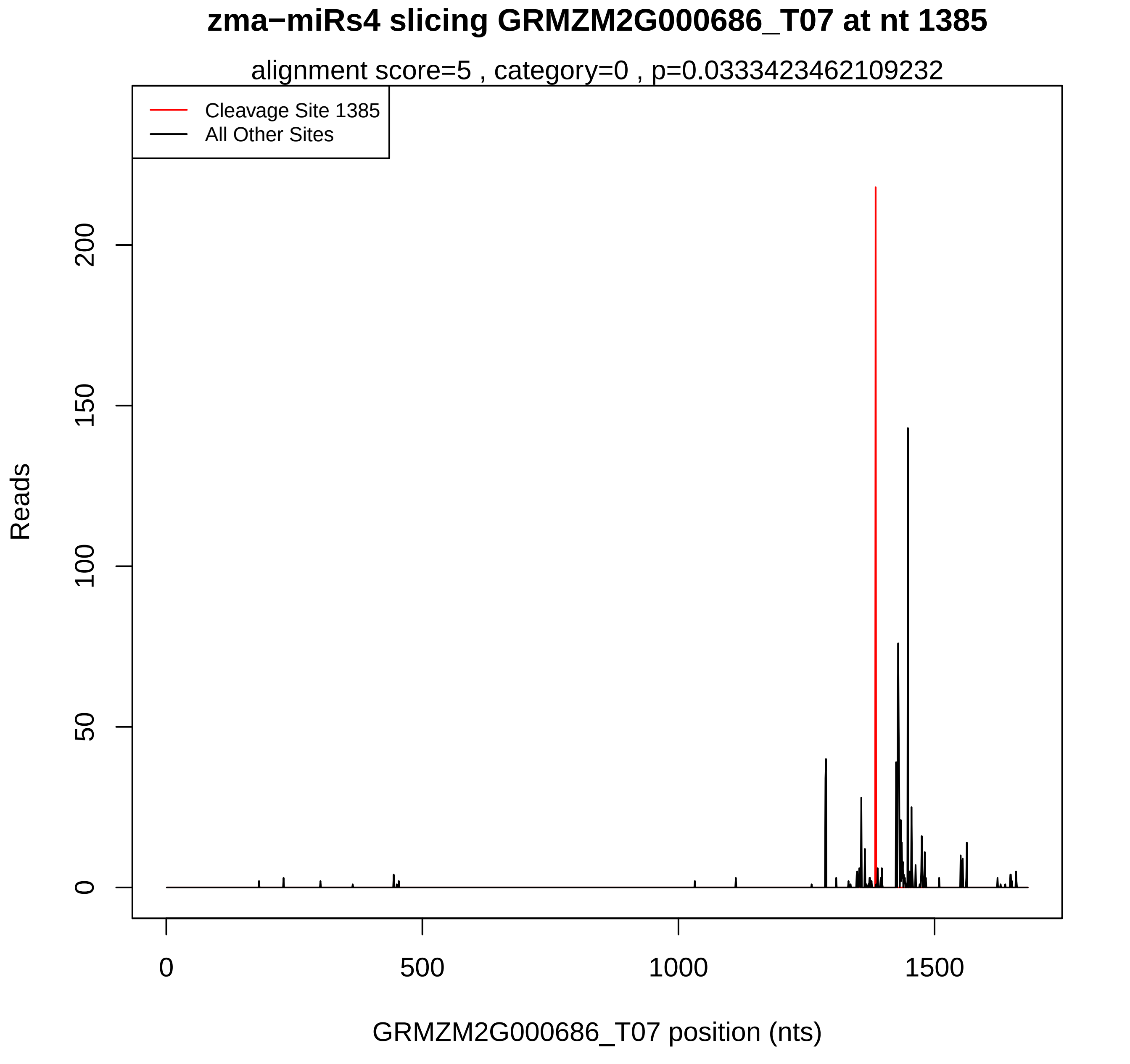
zma−miRs4 slicing GRMZM2G000686_T07 at nt 1385
alignment score=5 , category=0 , p=0.0333423462109232
Cleavage Site 1385
All Other Sites
Reads
0
50
100
150
200
0
500
1000
1500
GRMZM2G000686_T07 position (nts)

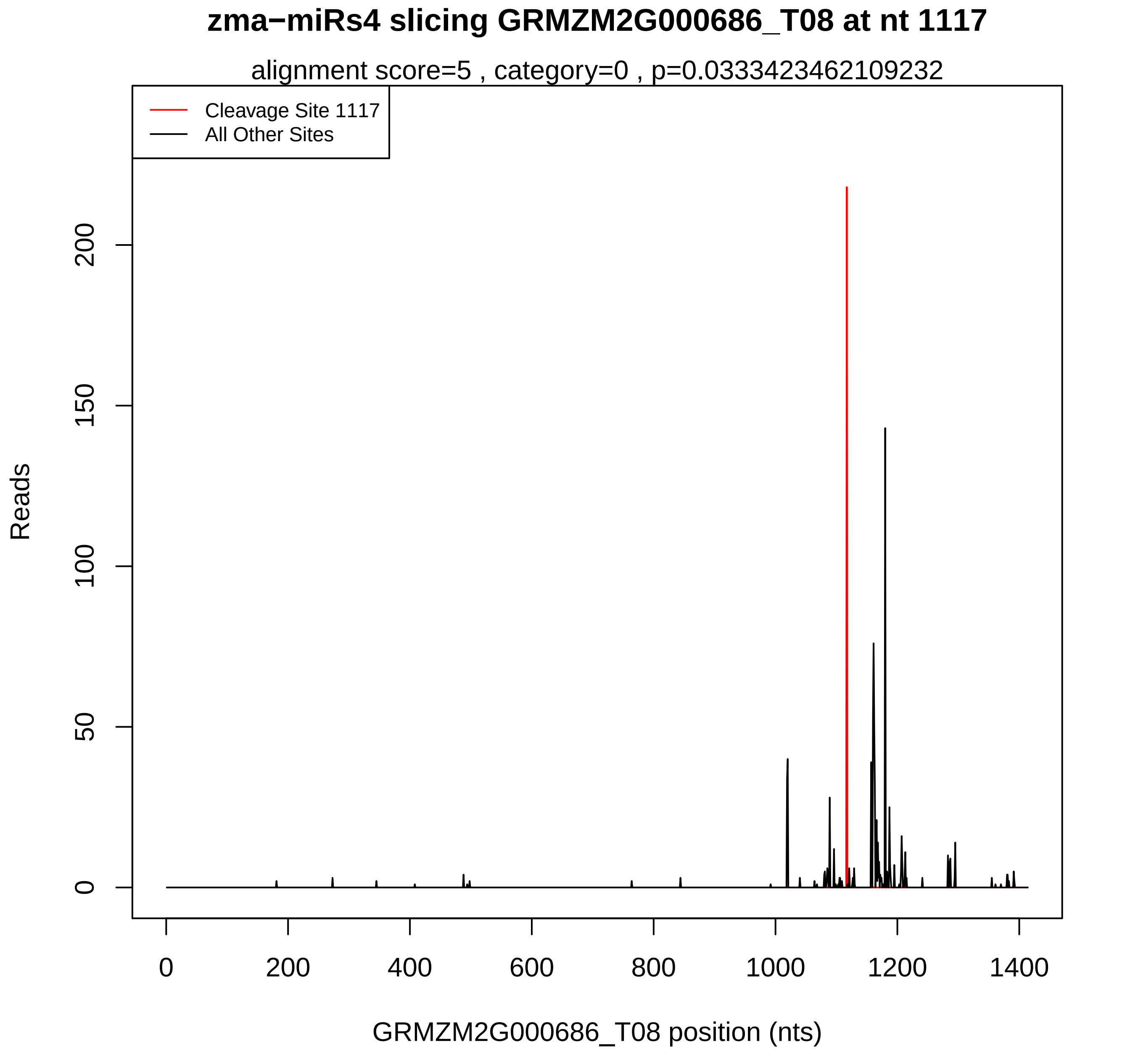
zma−miRs4 slicing GRMZM2G000686_T08 at nt 1117
alignment score=5 , category=0 , p=0.0333423462109232
Cleavage Site 1117
All Other Sites
Reads
0
50
100
150
200
0
200
400
600
800
1000
1200
1400
GRMZM2G000686_T08 position (nts)

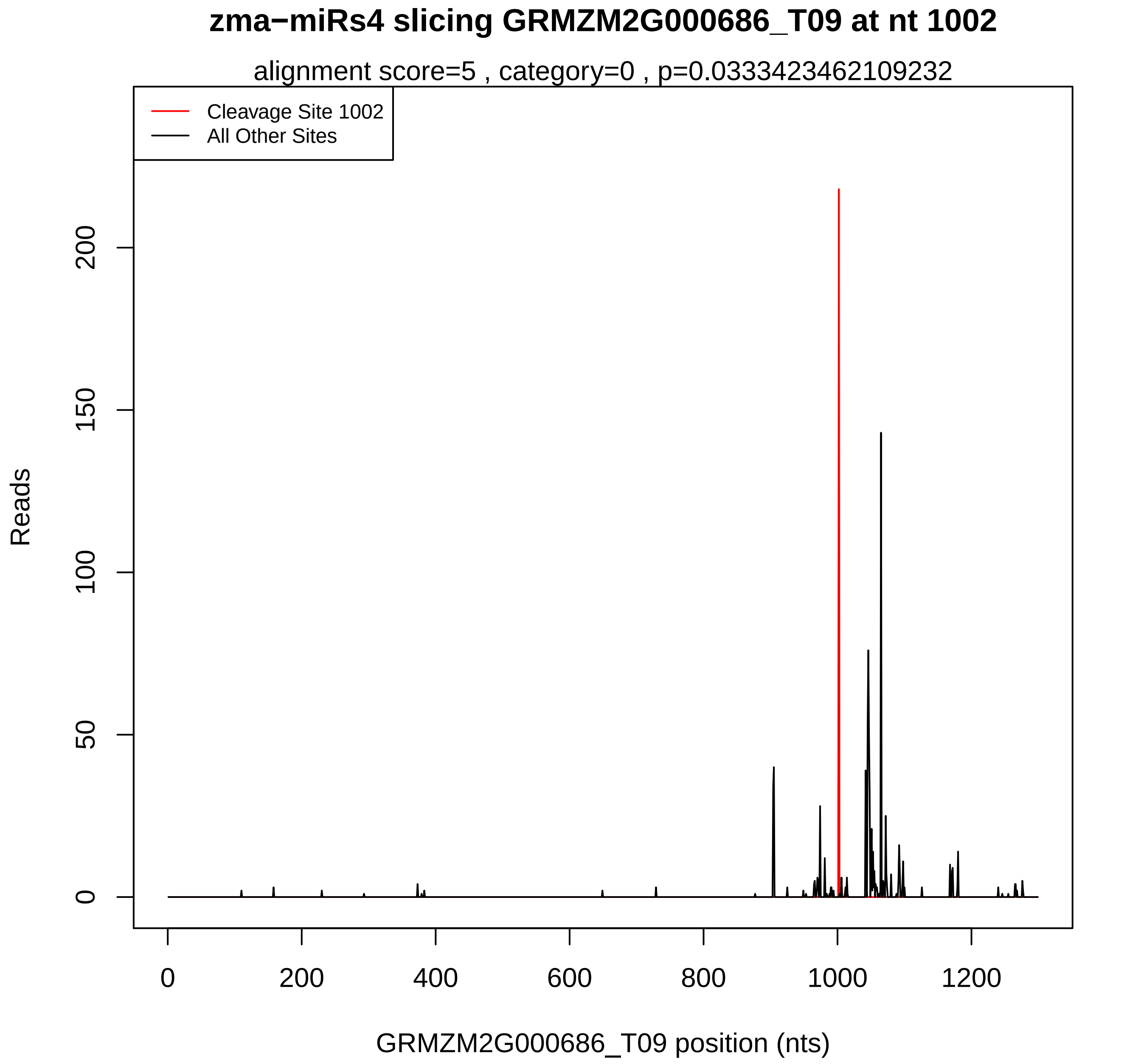
zma−miRs4 slicing GRMZM2G000686_T09 at nt 1002
alignment score=5 , category=0 , p=0.0333423462109232
Cleavage Site 1002
All Other Sites
Reads
0
50
100
150
200
0
200
400
600
800
1000
1200
GRMZM2G000686_T09 position (nts)

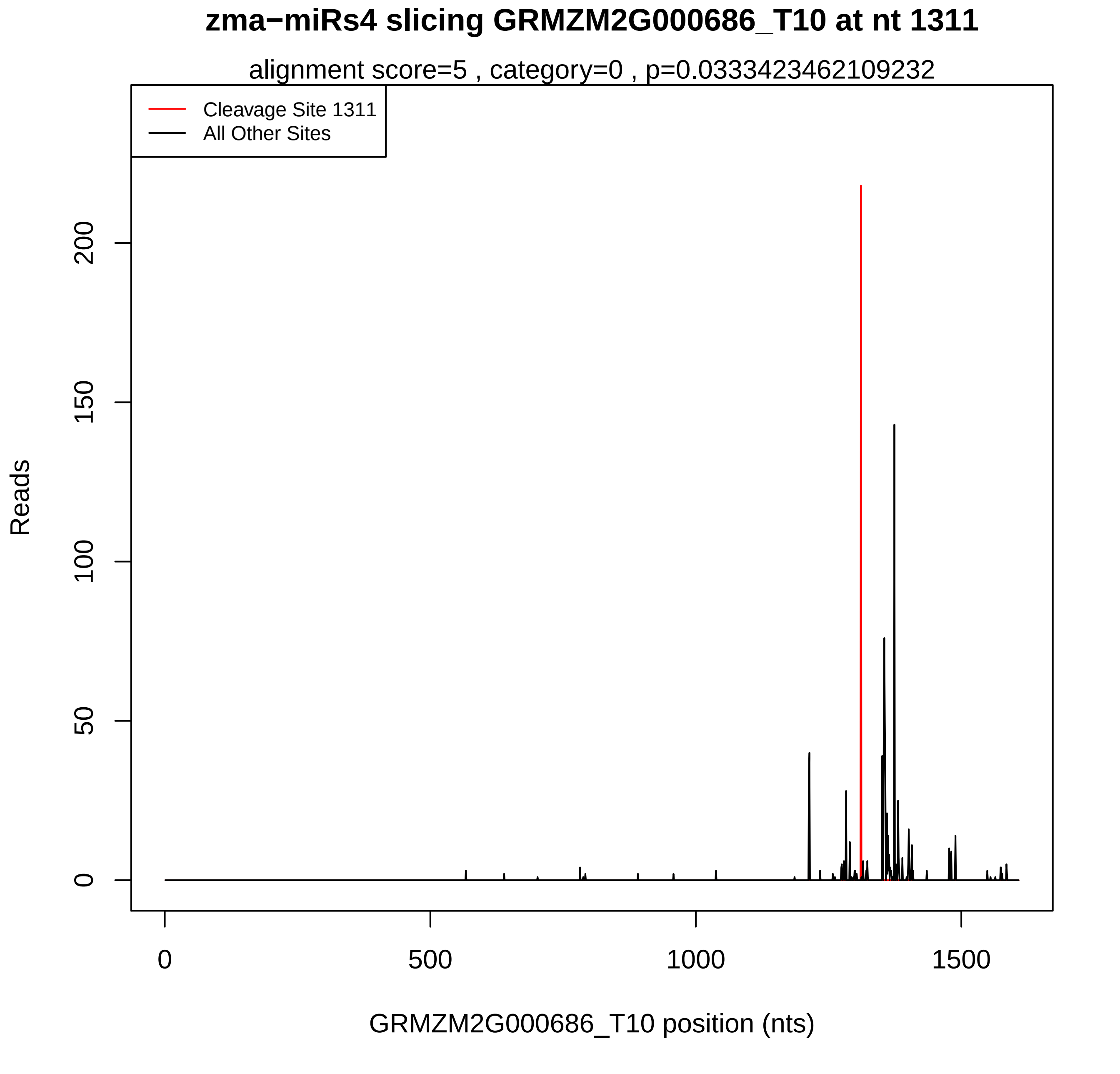
zma−miRs4 slicing GRMZM2G000686_T10 at nt 1311
alignment score=5 , category=0 , p=0.0333423462109232
Cleavage Site 1311
All Other Sites
Reads
0
50
100
150
200
0
500
1000
1500
GRMZM2G000686_T10 position (nts)

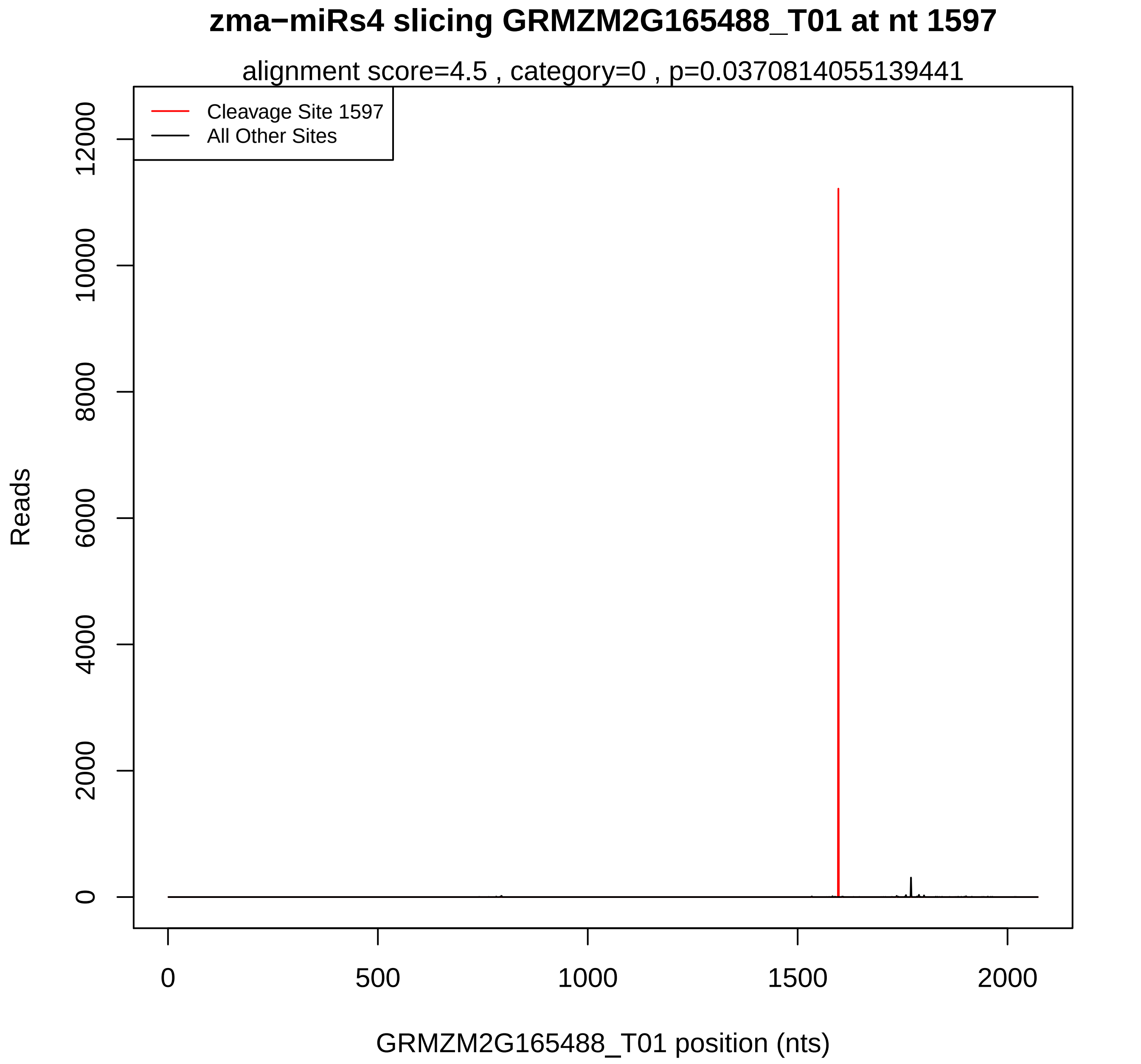

zma−miRs4 slicing GRMZM2G165488_T01 at nt 1597
alignment score=4.5 , category=0 , p=0.0370814055139441
Cleavage Site 1597
All Other Sites
Reads
0
2000
4000
6000
8000
10000
12000
0
500
1000
1500
2000
GRMZM2G165488_T01 position (nts)

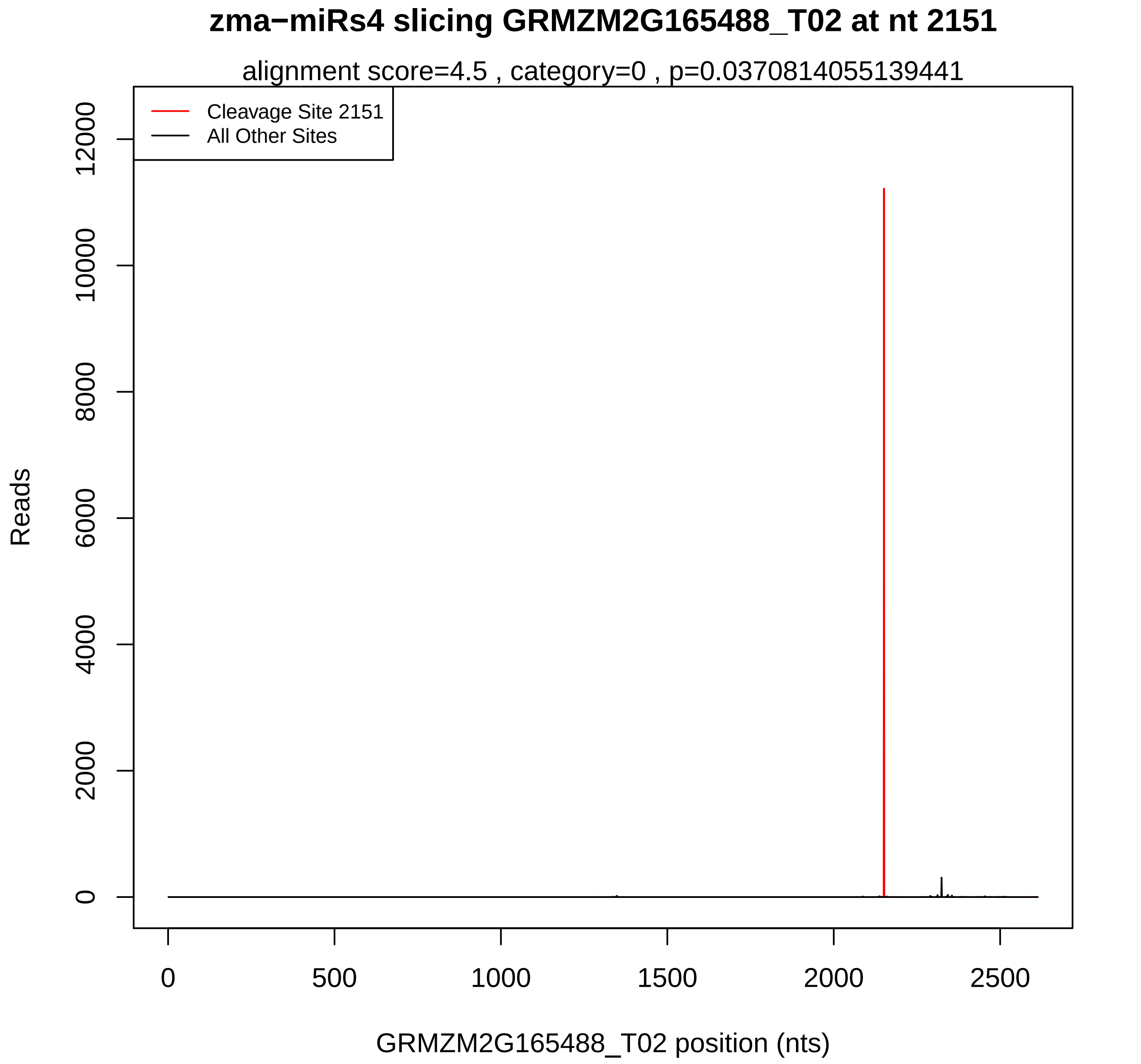
zma−miRs4 slicing GRMZM2G165488_T02 at nt 2151
alignment score=4.5 , category=0 , p=0.0370814055139441
Cleavage Site 2151
All Other Sites
Reads
0
2000
4000
6000
8000
10000
12000
0
500
1000
1500
2000
2500
GRMZM2G165488_T02 position (nts)

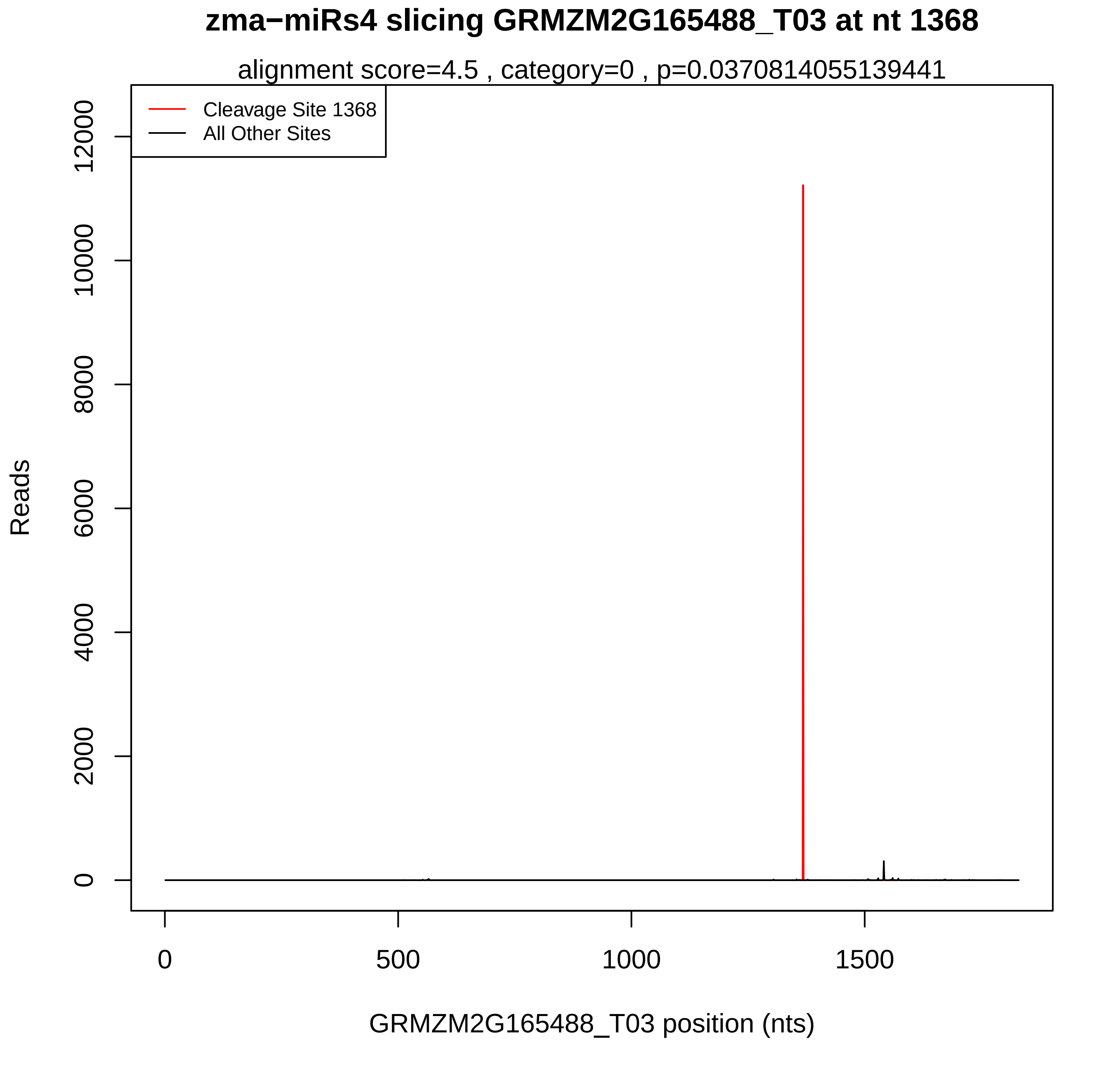

zma−miRs4 slicing GRMZM2G165488_T03 at nt 1368
alignment score=4.5 , category=0 , p=0.0370814055139441
Cleavage Site 1368
All Other Sites
Reads
0
2000
4000
6000
8000
10000
12000
0
500
1000
1500
GRMZM2G165488_T03 position (nts)

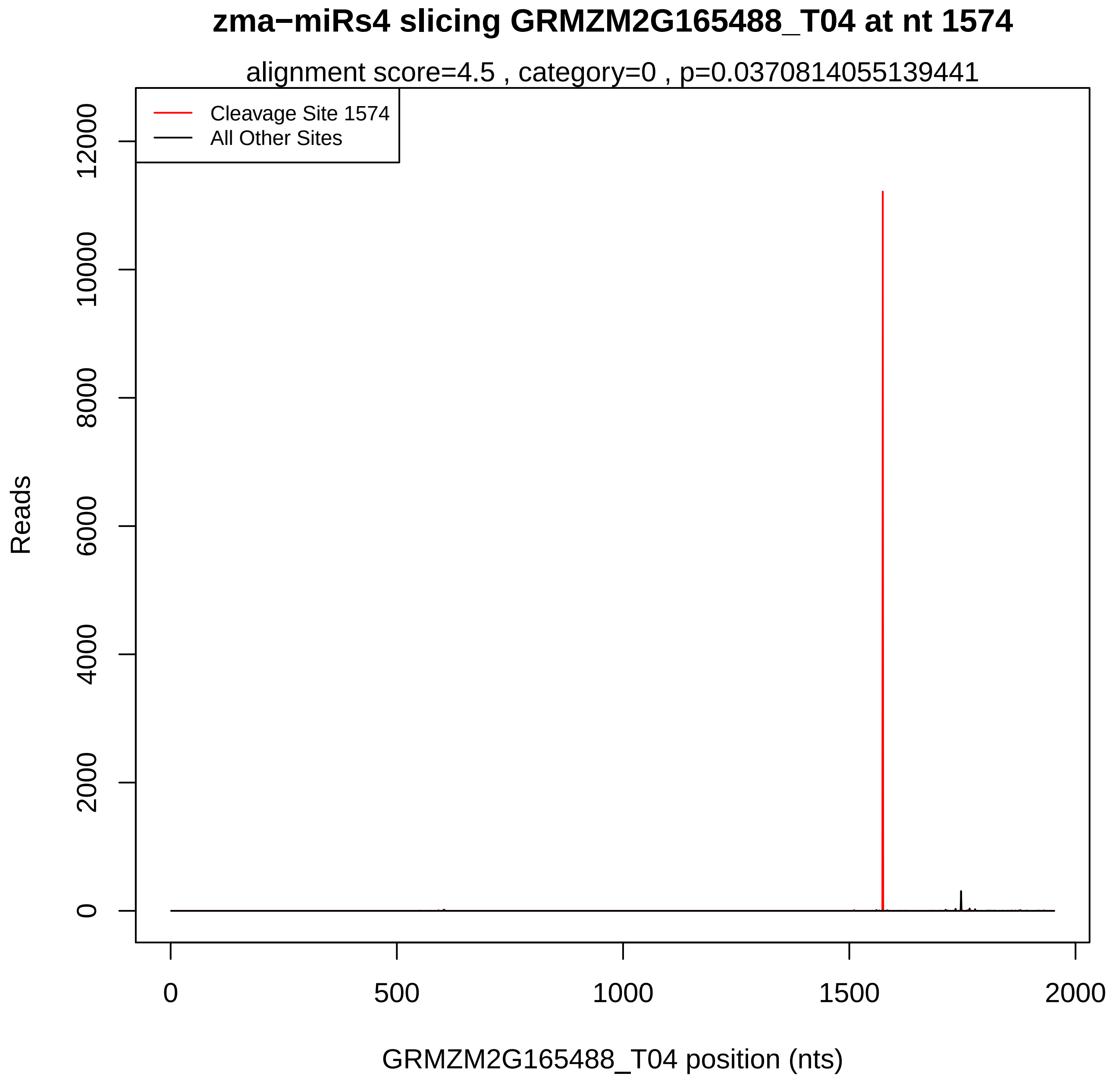
zma−miRs4 slicing GRMZM2G165488_T04 at nt 1574
alignment score=4.5 , category=0 , p=0.0370814055139441
Cleavage Site 1574
All Other Sites
Reads
0
2000
4000
6000
8000
10000
12000
0
500
1000
1500
2000
GRMZM2G165488_T04 position (nts)

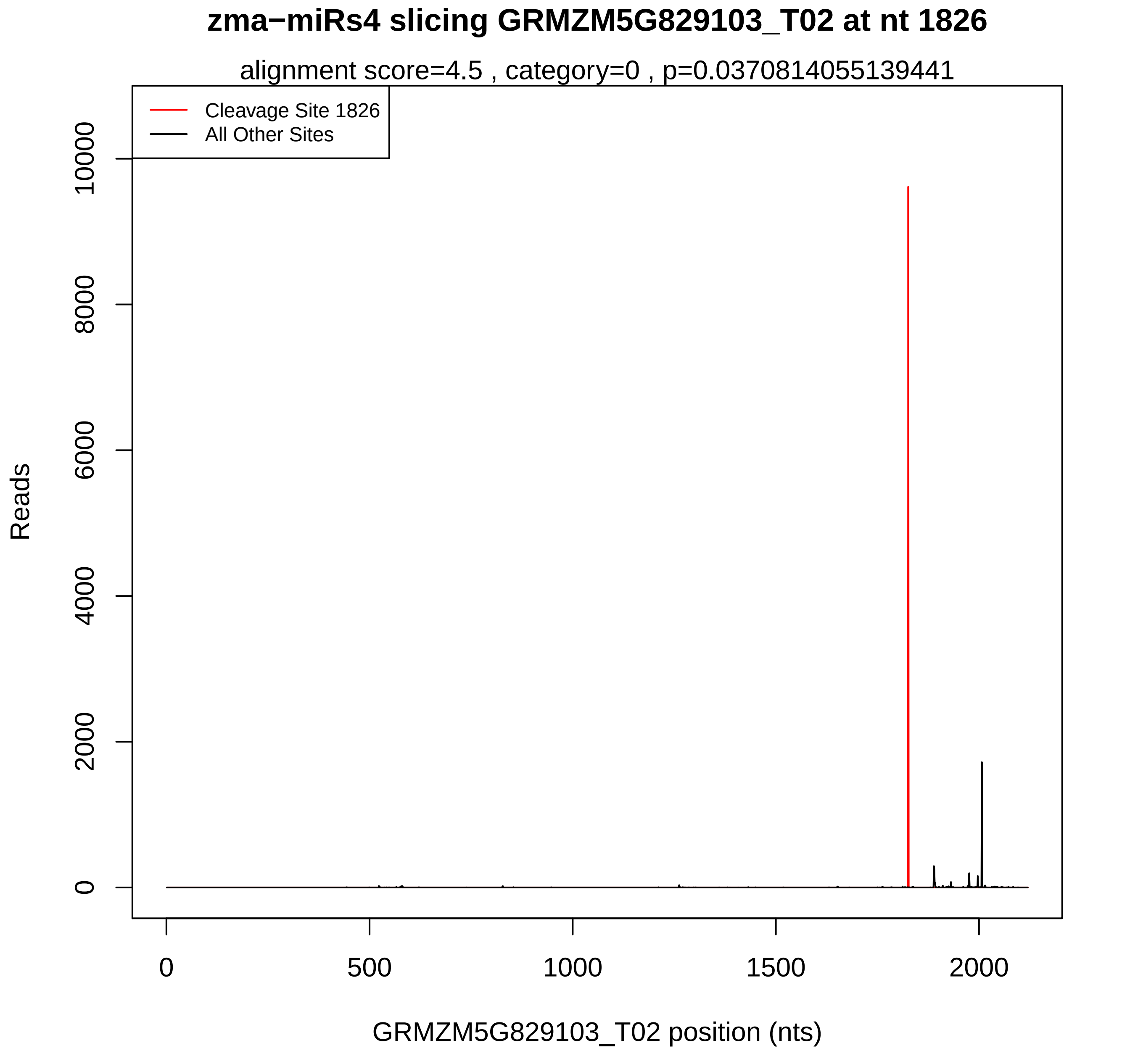
zma−miRs4 slicing GRMZM5G829103_T02 at nt 1826
alignment score=4.5 , category=0 , p=0.0370814055139441
Cleavage Site 1826
All Other Sites
Reads
0
2000
4000
6000
8000
10000
0
500
1000
1500
2000
GRMZM5G829103_T02 position (nts)

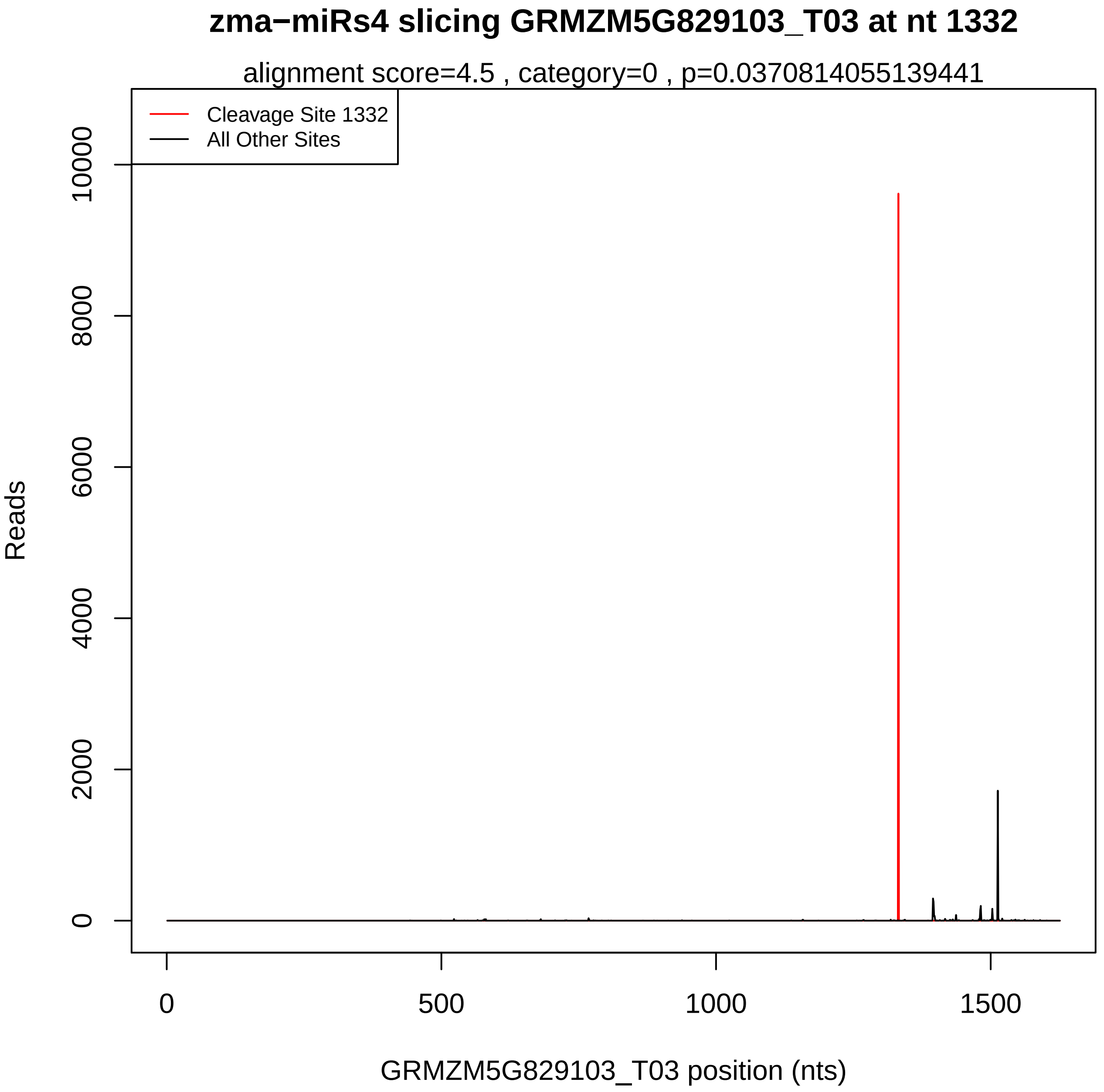
zma−miRs4 slicing GRMZM5G829103_T03 at nt 1332
alignment score=4.5 , category=0 , p=0.0370814055139441
Cleavage Site 1332
All Other Sites
Reads
0
2000
4000
6000
8000
10000
0
500
1000
1500
GRMZM5G829103_T03 position (nts)

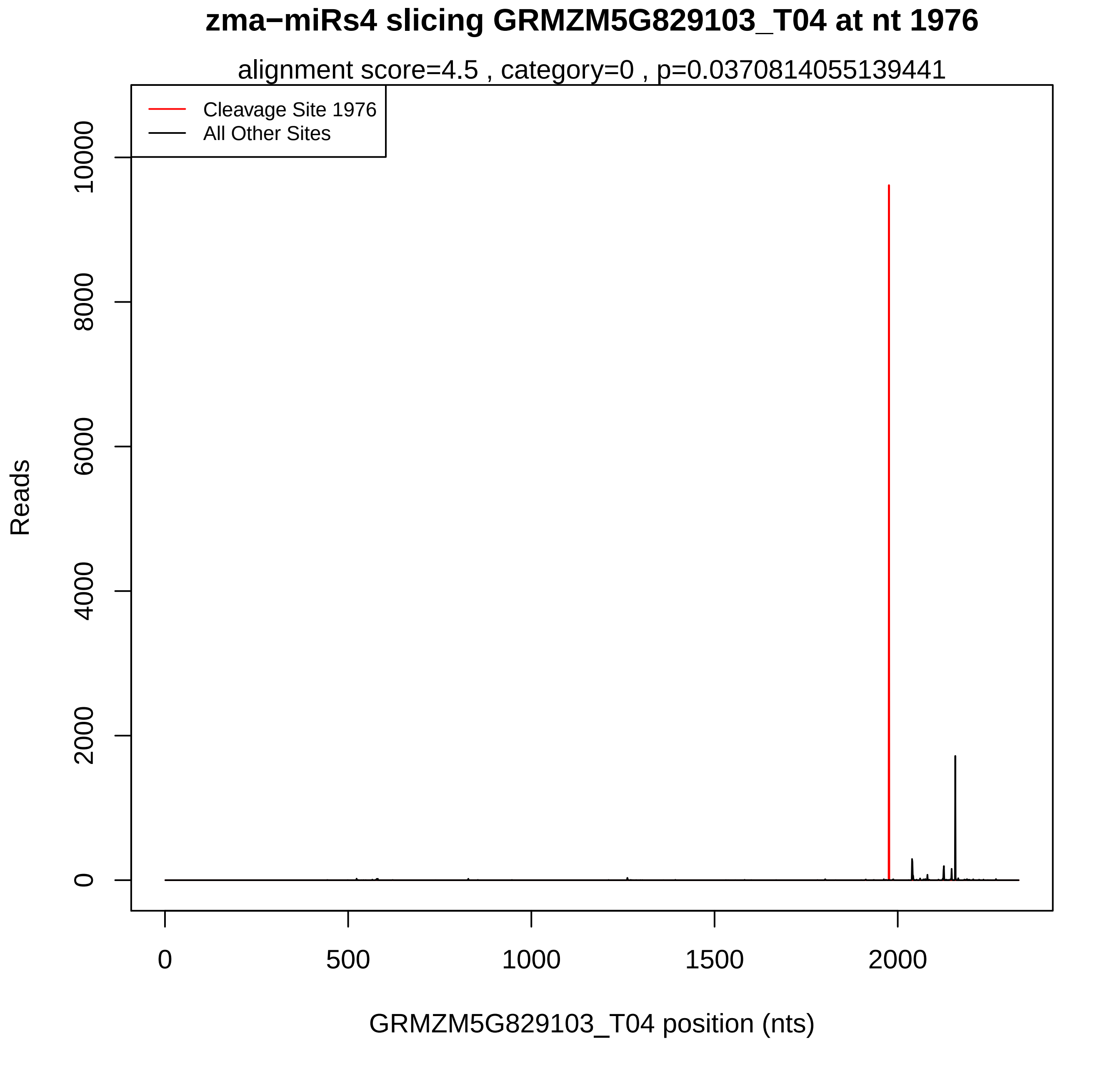

zma−miRs4 slicing GRMZM5G829103_T04 at nt 1976
alignment score=4.5 , category=0 , p=0.0370814055139441
Cleavage Site 1976
All Other Sites
Reads
0
2000
4000
6000
8000
10000
0
500
1000
1500
2000
GRMZM5G829103_T04 position (nts)

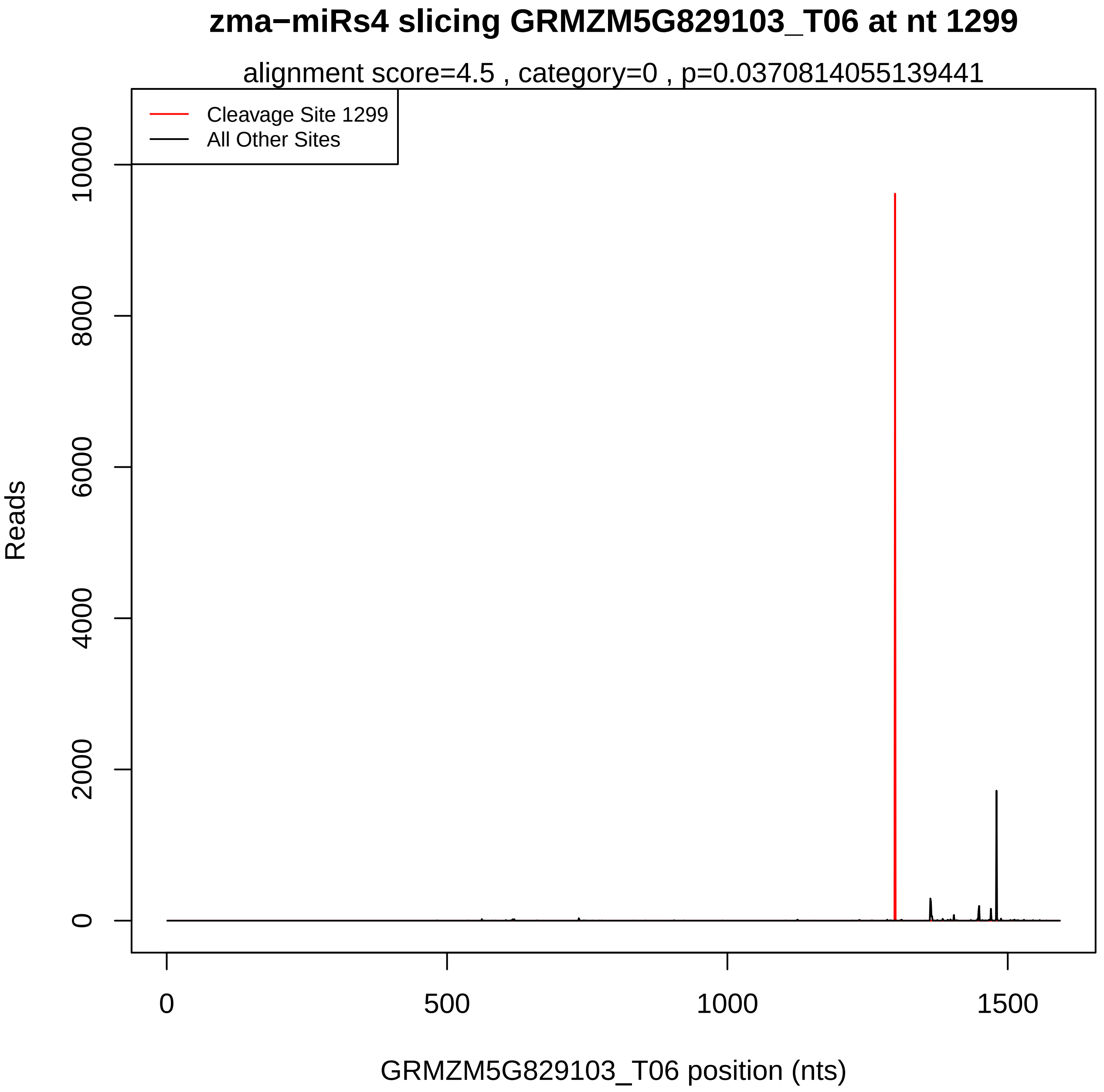
zma−miRs4 slicing GRMZM5G829103_T06 at nt 1299
alignment score=4.5 , category=0 , p=0.0370814055139441
Cleavage Site 1299
All Other Sites
Reads
0
2000
4000
6000
8000
10000
0
500
1000
1500
GRMZM5G829103_T06 position (nts)

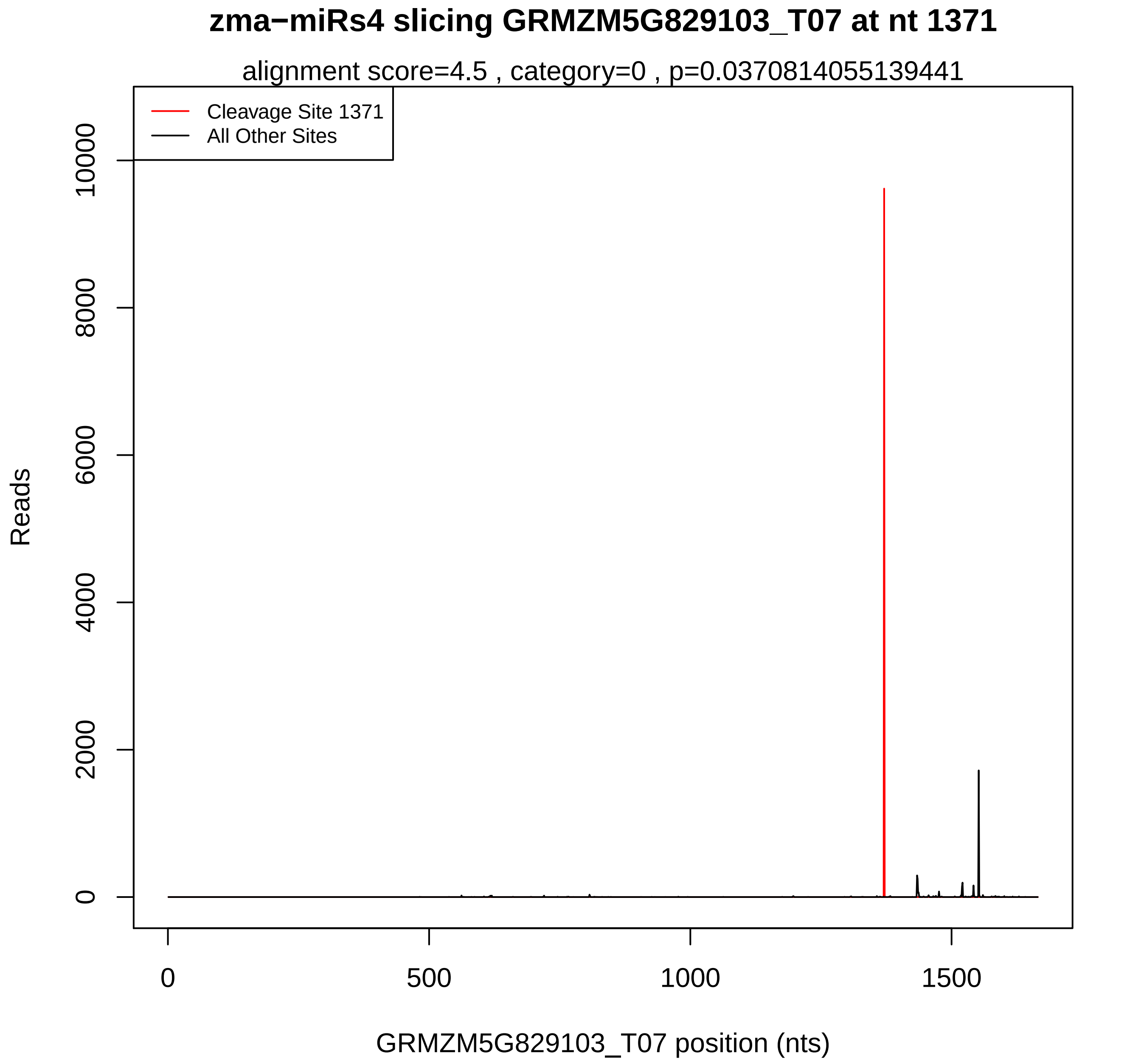

zma−miRs4 slicing GRMZM5G829103_T07 at nt 1371
alignment score=4.5 , category=0 , p=0.0370814055139441
Cleavage Site 1371
All Other Sites
Reads
0
2000
4000
6000
8000
10000
0
500
1000
1500
GRMZM5G829103_T07 position (nts)

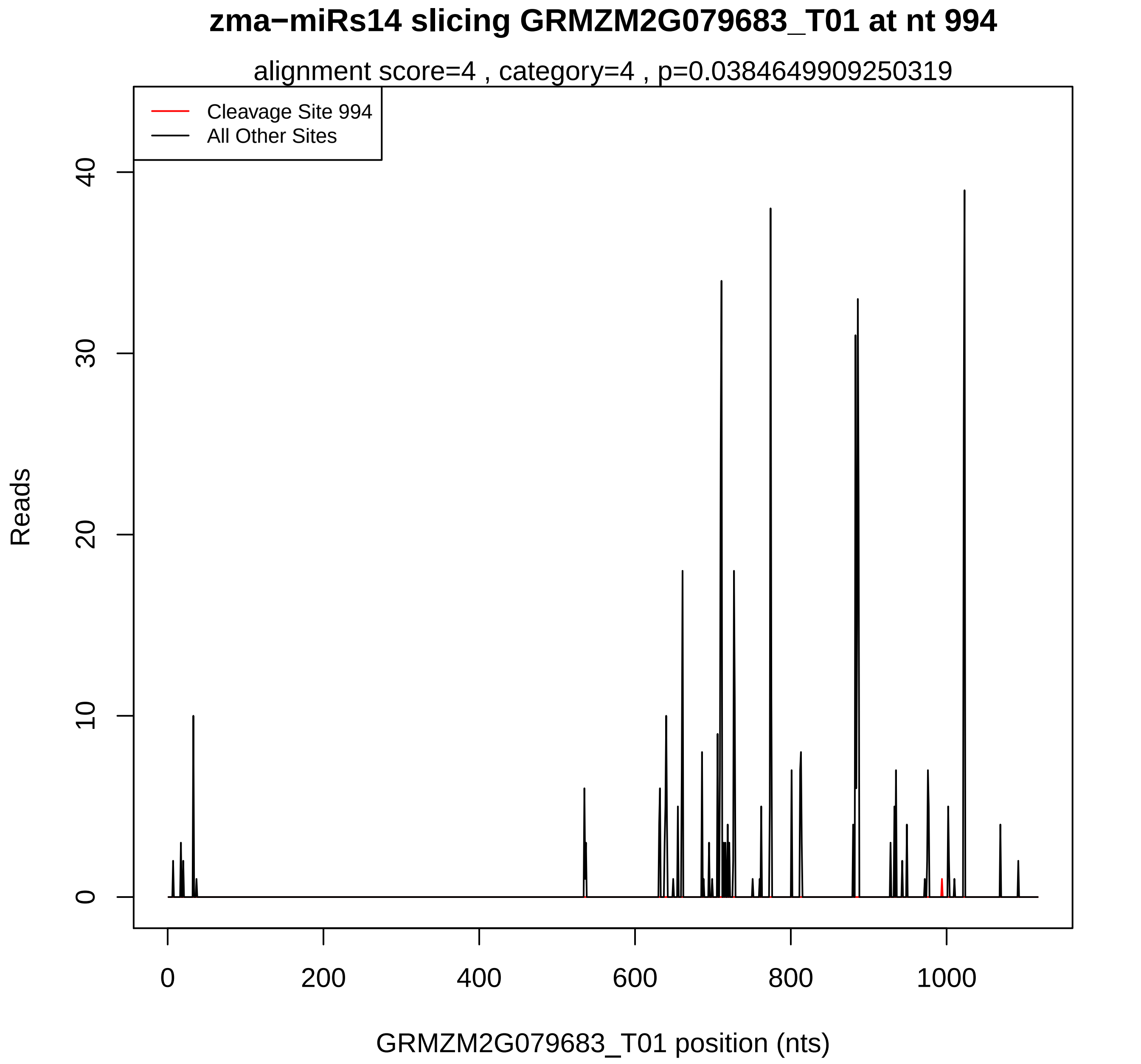
zma−miRs14 slicing GRMZM2G079683_T01 at nt 994
alignment score=4 , category=4 , p=0.0384649909250319
Cleavage Site 994
All Other Sites
Reads
0
10
20
30
40
0
200
400
600
800
1000
GRMZM2G079683_T01 position (nts)

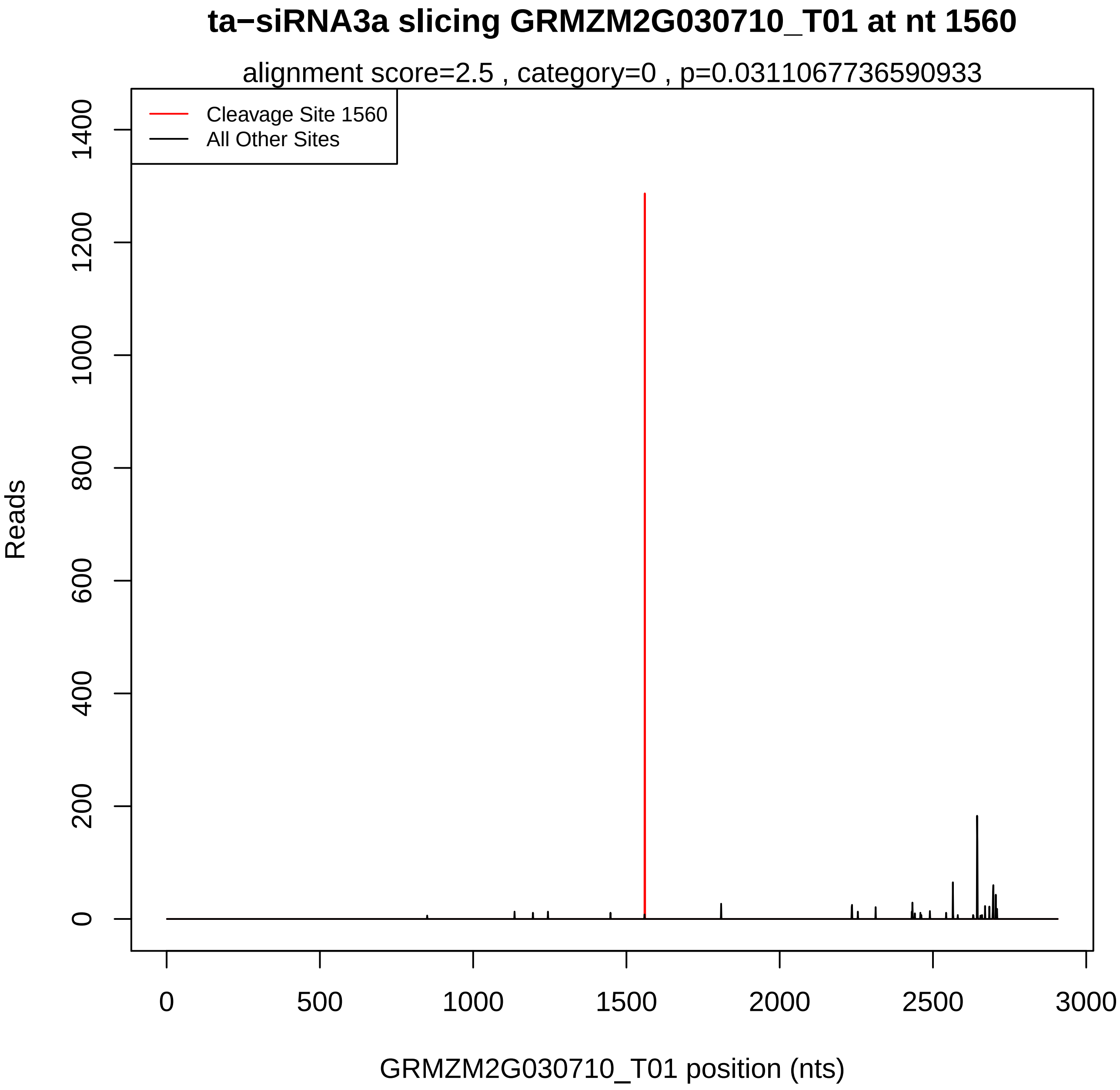

ta−siRNA3a slicing GRMZM2G030710_T01 at nt 1560
alignment score=2.5 , category=0 , p=0.0311067736590933
Cleavage Site 1560
All Other Sites
Reads
0
200
400
600
800
1000
1200
1400
0
500
1000
1500
2000
2500
3000
GRMZM2G030710_T01 position (nts)

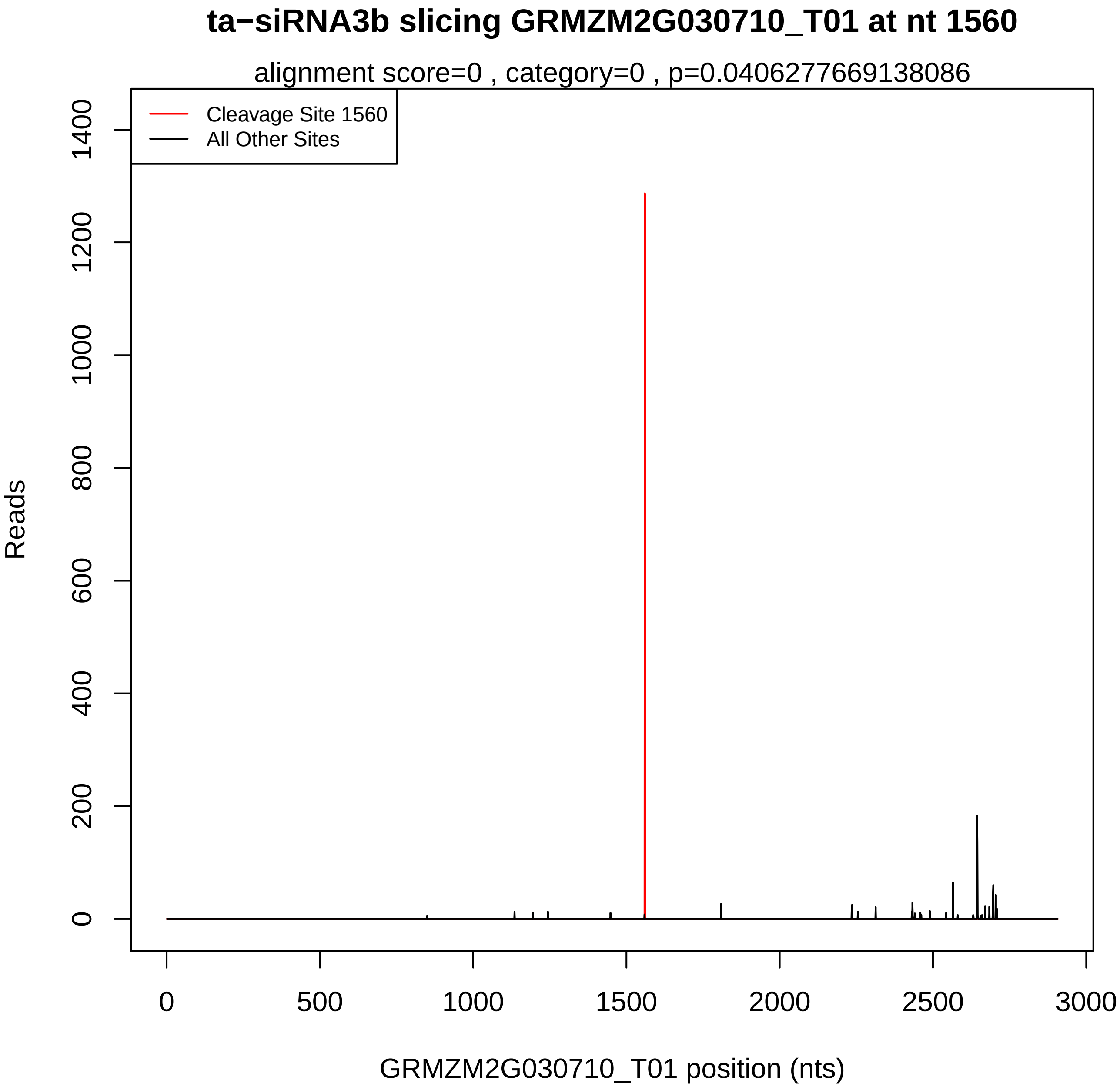
ta−siRNA3b slicing GRMZM2G030710_T01 at nt 1560
alignment score=0 , category=0 , p=0.0406277669138086
Cleavage Site 1560
All Other Sites
Reads
0
200
400
600
800
1000
1200
1400
0
500
1000
1500
2000
2500
3000
GRMZM2G030710_T01 position (nts)

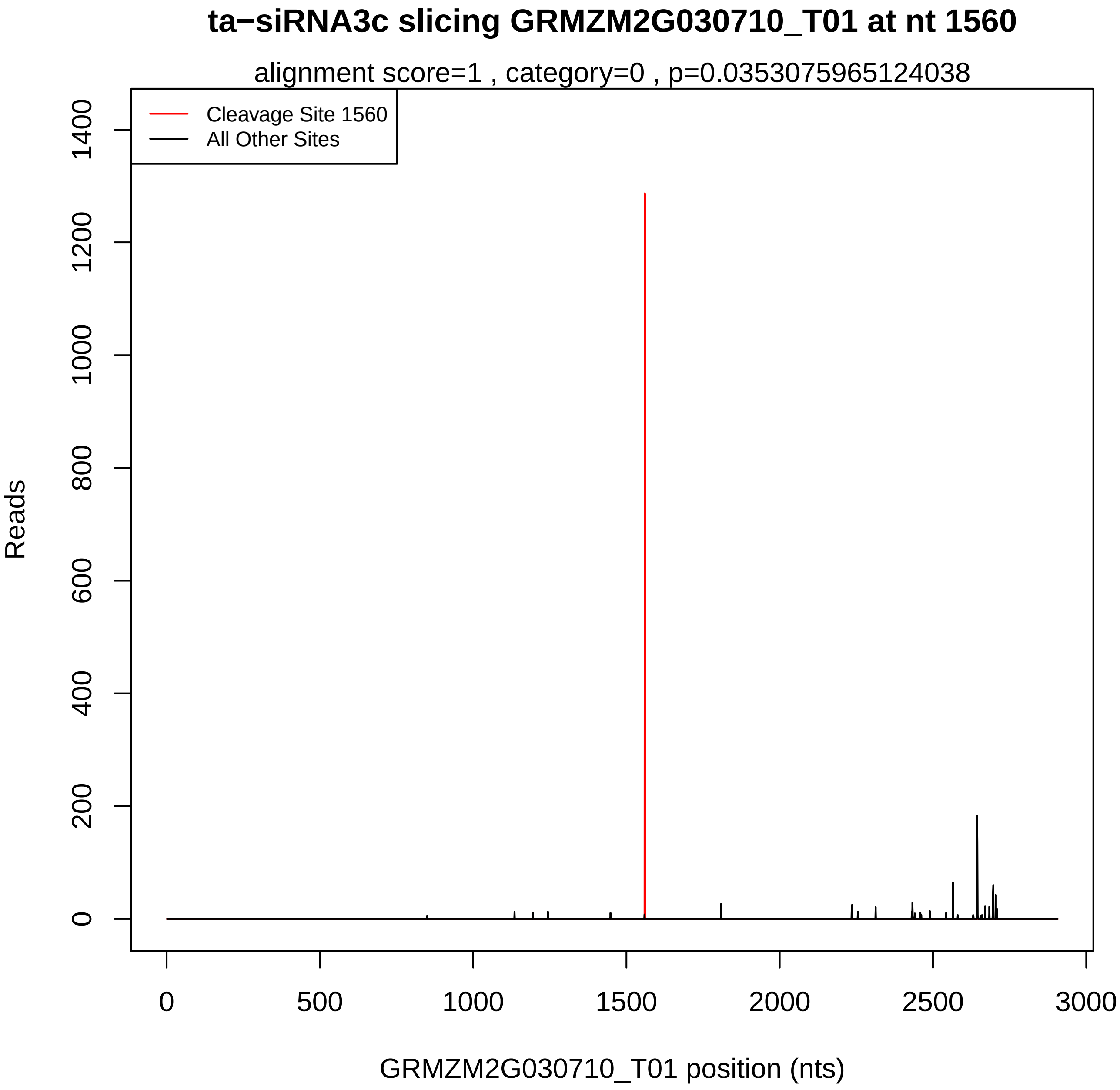
ta−siRNA3c slicing GRMZM2G030710_T01 at nt 1560
alignment score=1 , category=0 , p=0.0353075965124038
Cleavage Site 1560
All Other Sites
Reads
0
200
400
600
800
1000
1200
1400
0
500
1000
1500
2000
2500
3000
GRMZM2G030710_T01 position (nts)

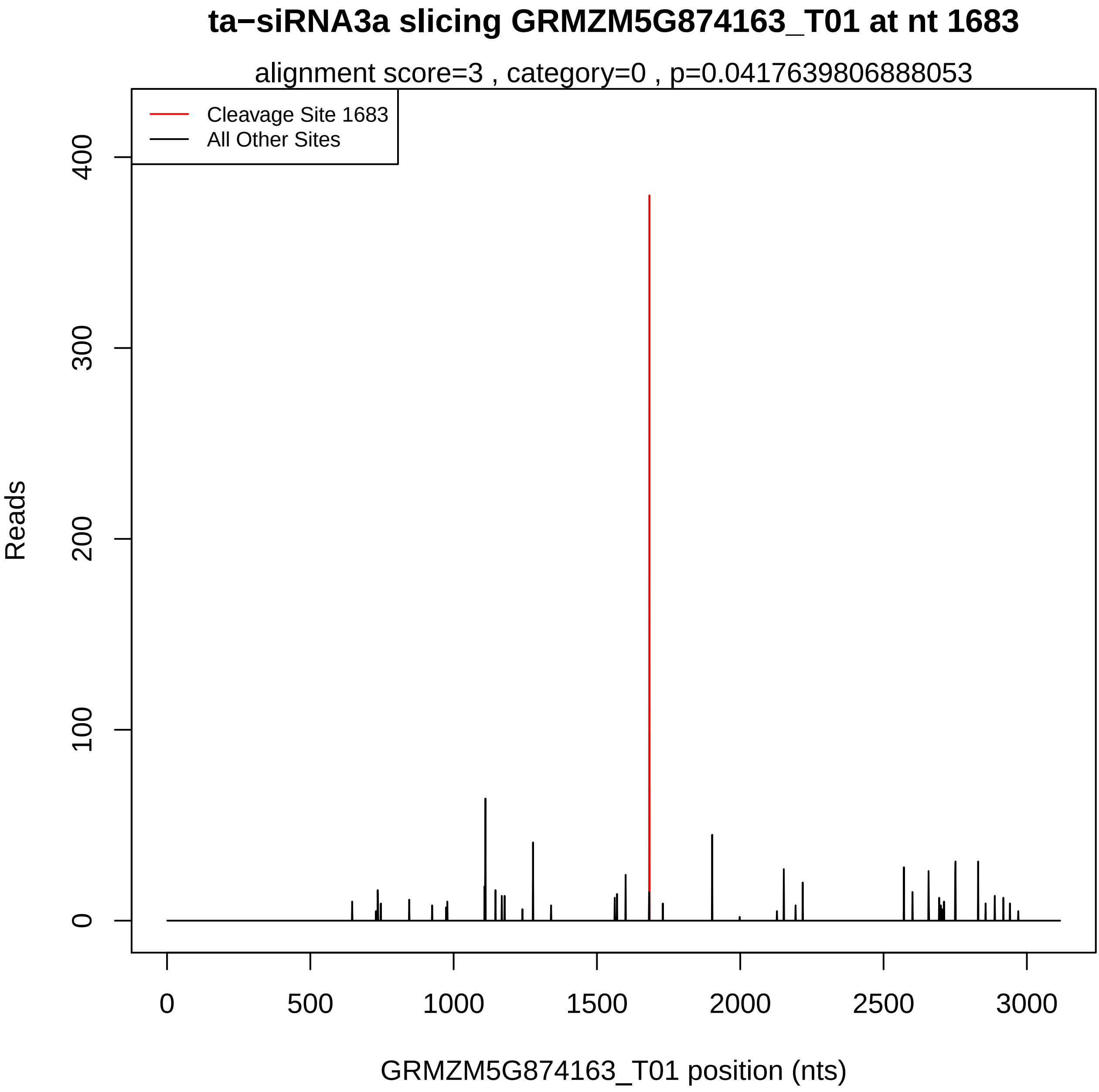
ta−siRNA3a slicing GRMZM5G874163_T01 at nt 1683
alignment score=3 , category=0 , p=0.0417639806888053
Cleavage Site 1683
All Other Sites
Reads
0
100
200
300
400
0
500
1000
1500
2000
2500
3000
GRMZM5G874163_T01 position (nts)

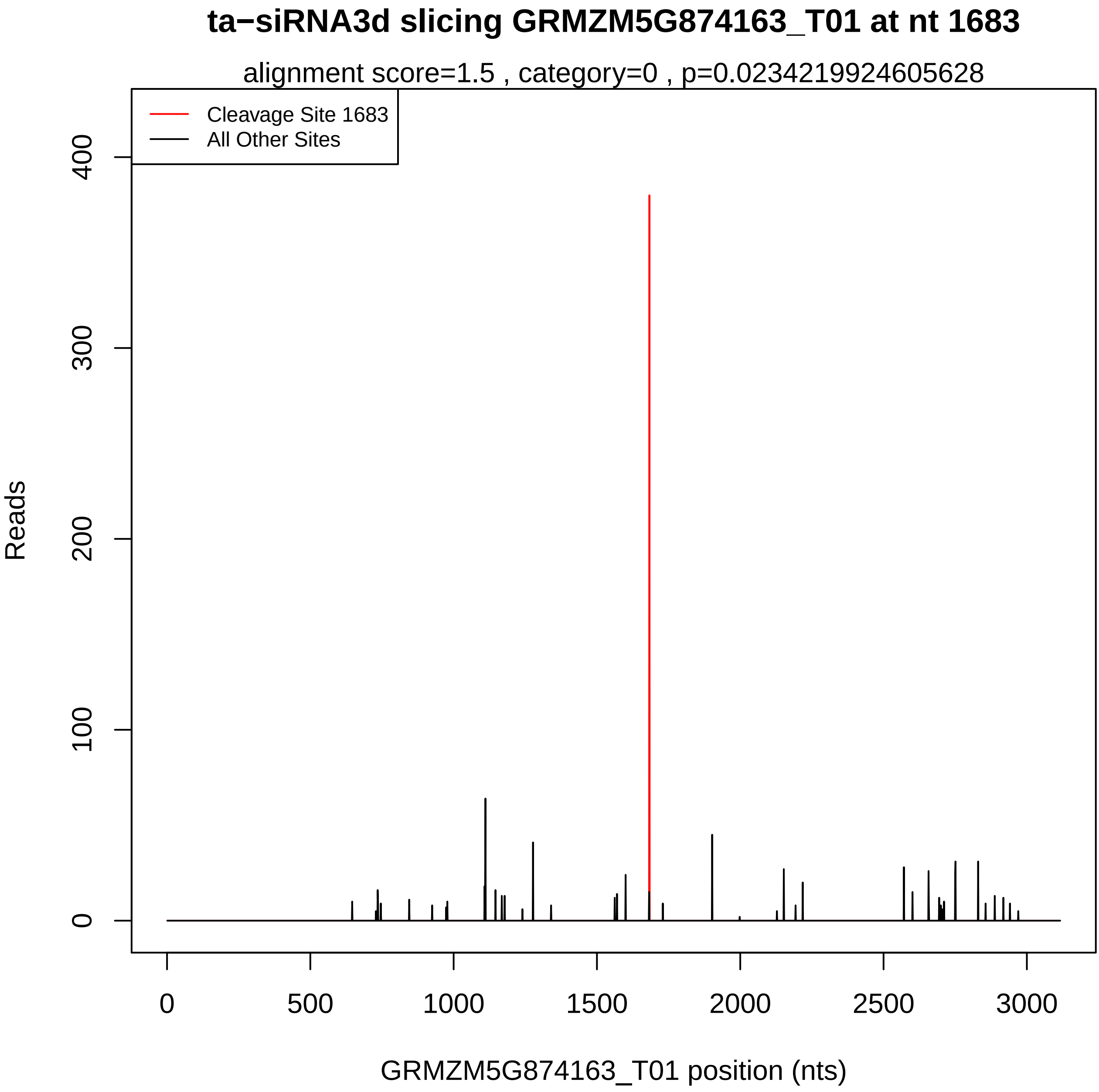

ta−siRNA3d slicing GRMZM5G874163_T01 at nt 1683
alignment score=1.5 , category=0 , p=0.0234219924605628
Cleavage Site 1683
All Other Sites
Reads
0
100
200
300
400
0
500
1000
1500
2000
2500
3000
GRMZM5G874163_T01 position (nts)

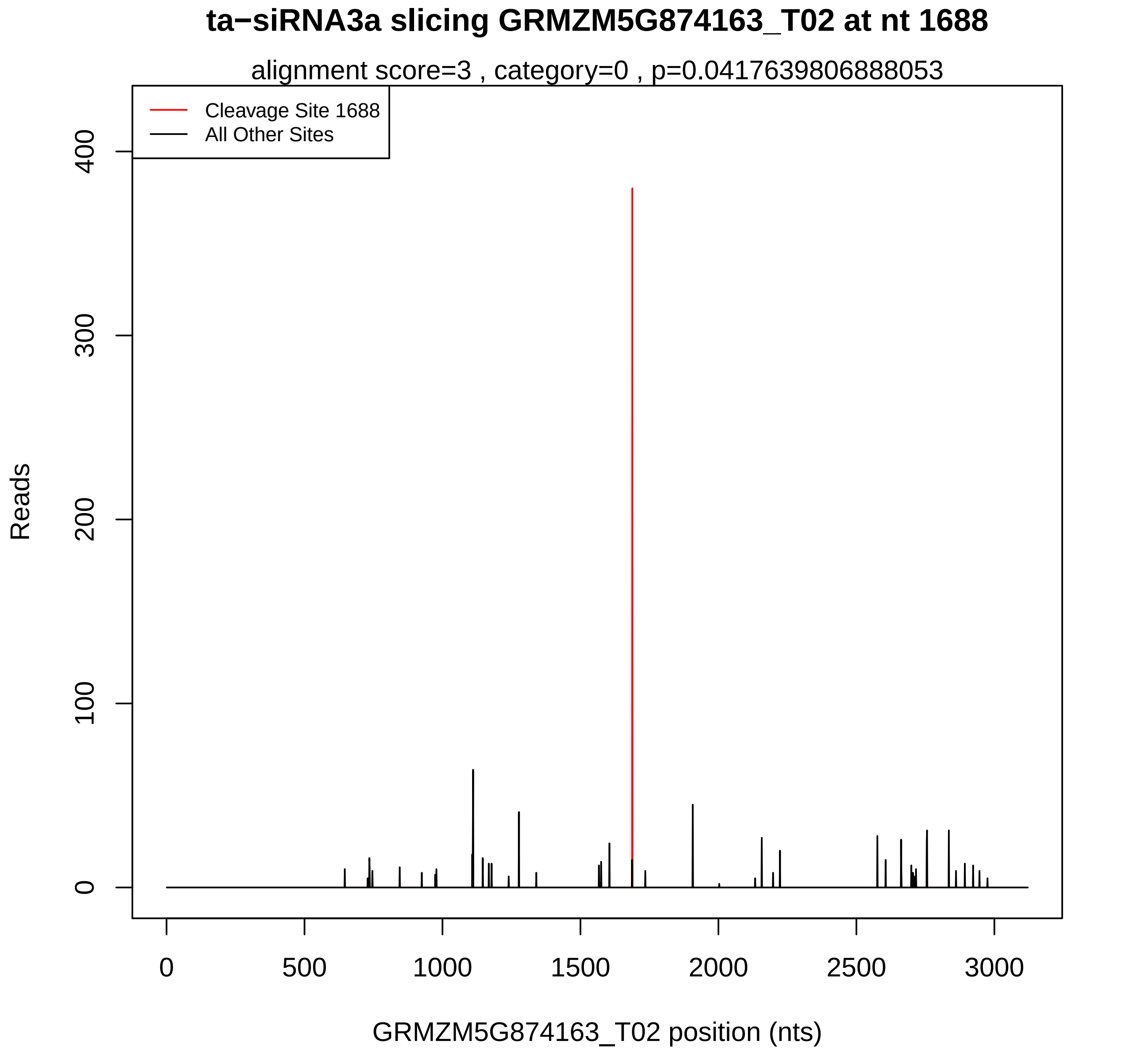
ta−siRNA3a slicing GRMZM5G874163_T02 at nt 1688
alignment score=3 , category=0 , p=0.0417639806888053
Cleavage Site 1688
All Other Sites
Reads
0
100
200
300
400
0
500
1000
1500
2000
2500
3000
GRMZM5G874163_T02 position (nts)

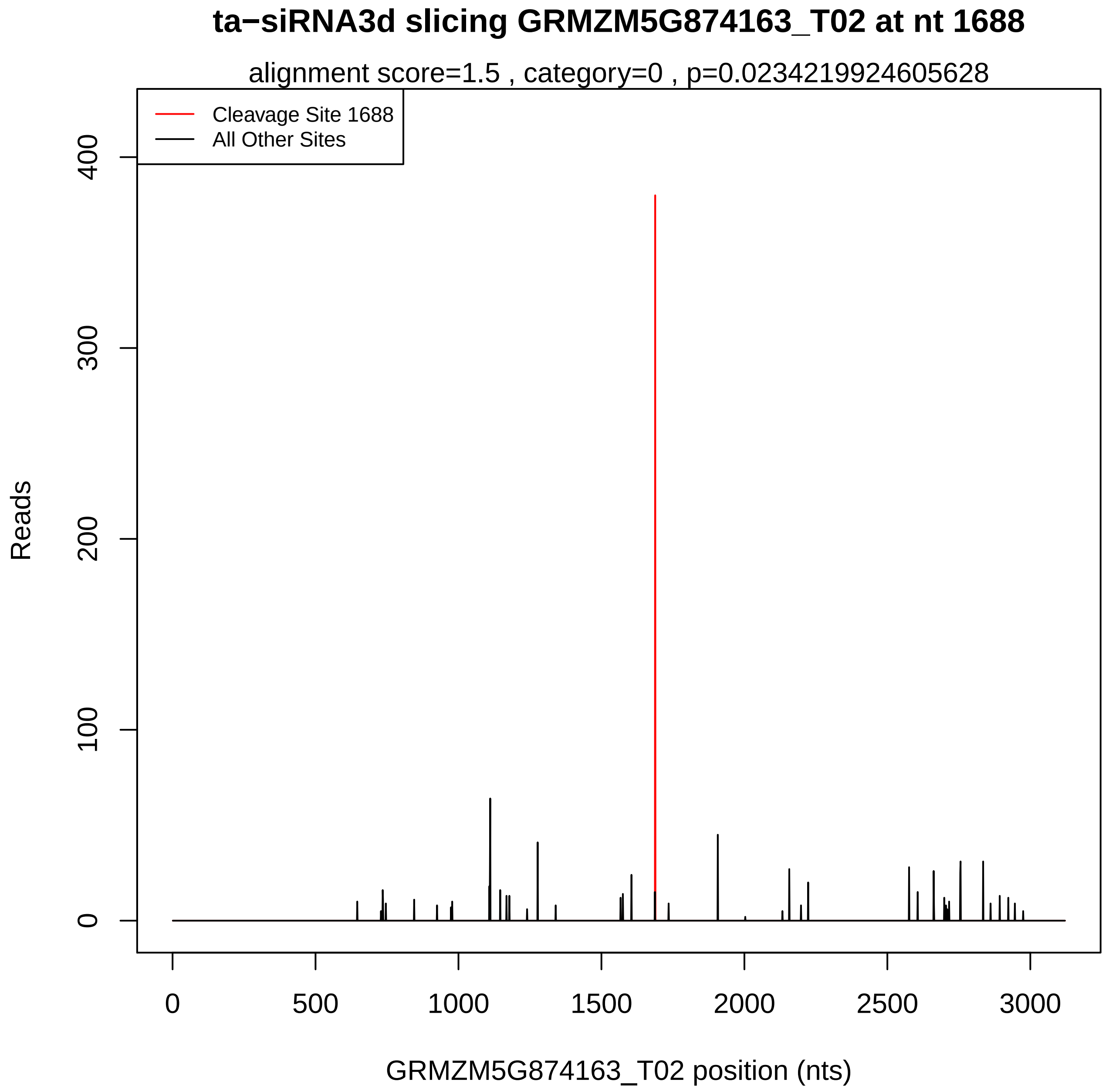

ta−siRNA3d slicing GRMZM5G874163_T02 at nt 1688
alignment score=1.5 , category=0 , p=0.0234219924605628
Cleavage Site 1688
All Other Sites
Reads
0
100
200
300
400
0
500
1000
1500
2000
2500
3000
GRMZM5G874163_T02 position (nts)

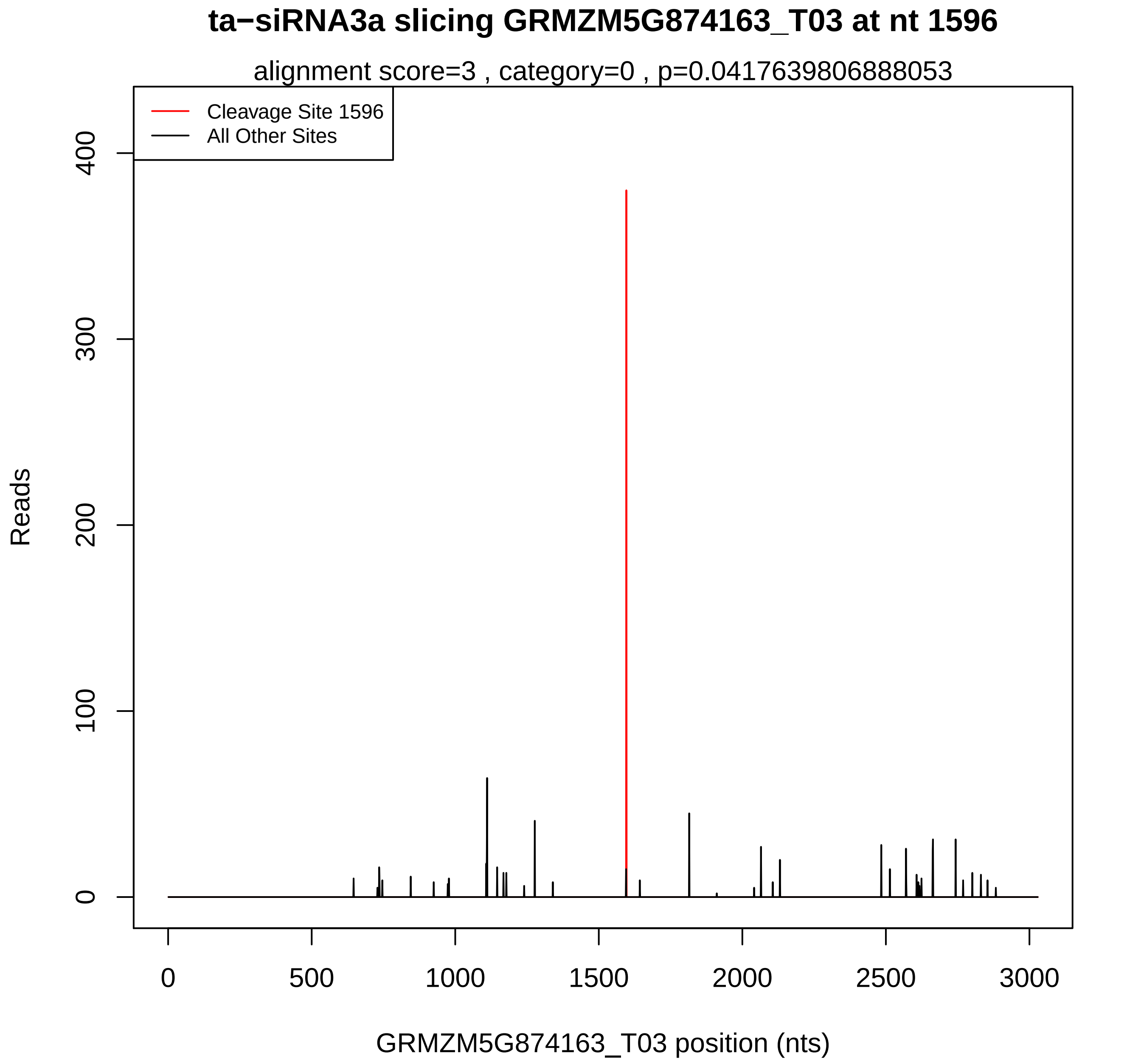
ta−siRNA3a slicing GRMZM5G874163_T03 at nt 1596
alignment score=3 , category=0 , p=0.0417639806888053
Cleavage Site 1596
All Other Sites
Reads
0
100
200
300
400
0
500
1000
1500
2000
2500
3000
GRMZM5G874163_T03 position (nts)

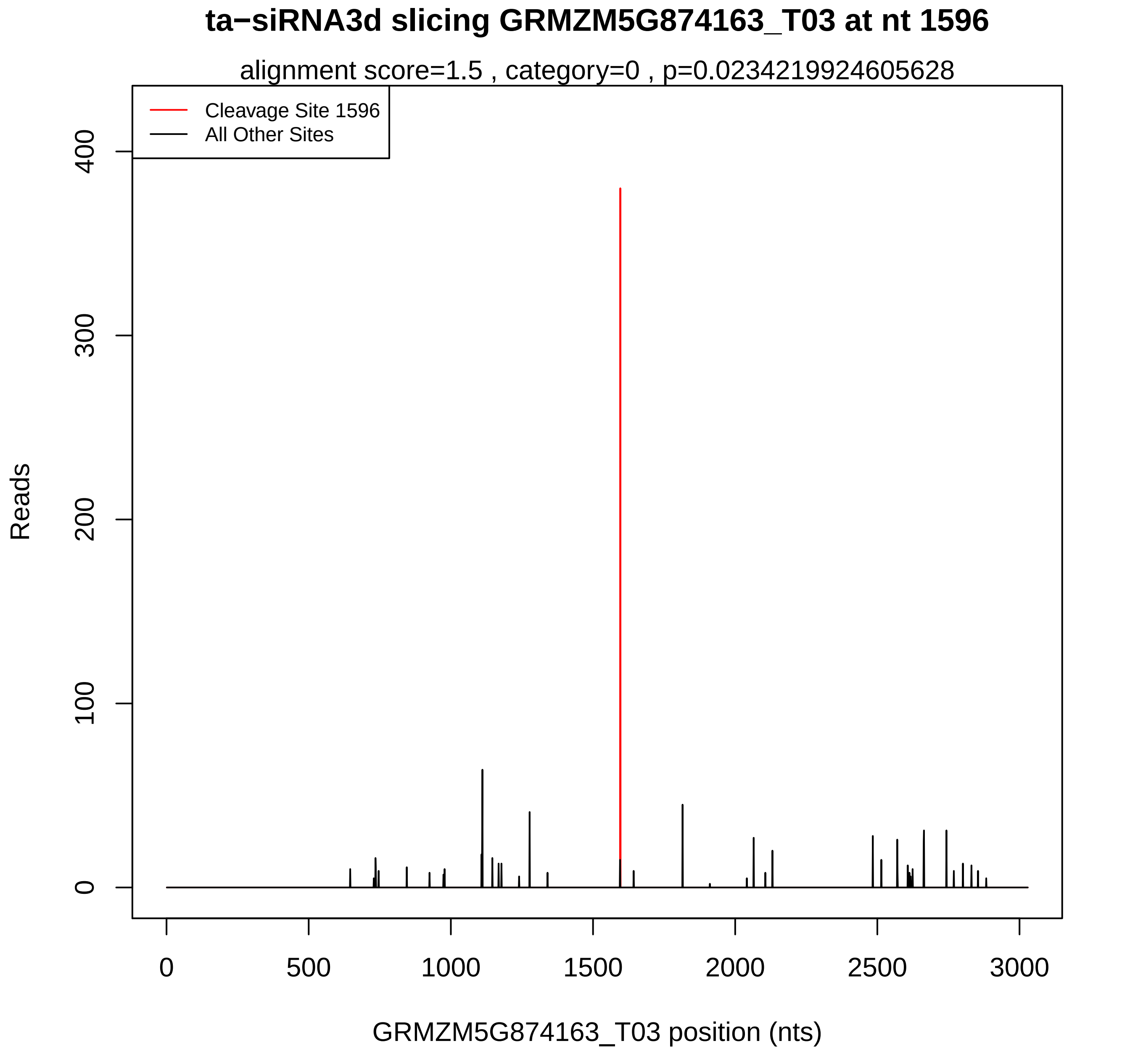
ta−siRNA3d slicing GRMZM5G874163_T03 at nt 1596
alignment score=1.5 , category=0 , p=0.0234219924605628
Cleavage Site 1596
All Other Sites
Reads
0
100
200
300
400
0
500
1000
1500
2000
2500
3000
GRMZM5G874163_T03 position (nts)
